# Plant genetic and root‐associated microbial diversity modulate *Lactuca sativa* responsiveness to a soil inoculum under phosphate deficiency

**DOI:** 10.1111/nph.71529

**Published:** 2026-09-02

**Authors:** Arianna Capparotto, Alessandro Ciampanelli, Paolo Salvucci, Guillaume Chesneau, Johannes Herpell, Simone Sello, Cristina Sudiro, Pieter Clauw, Adriano Altissimo, Stéphane Hacquard, Francesco Vuolo, Marco Giovannetti

**Affiliations:** ^1^ Department of Biology University of Padova 35131 Padova Italy; ^2^ Department of Life Sciences and Systems Biology University of Torino 10125 Torino Italy; ^3^ Department of Plant–Microbe Interactions Max Planck Institute for Plant Breeding Research 50829 Cologne Germany; ^4^ Landlab Srl Via Quintarello 12/A 36050 Quinto Vicentino Italy; ^5^ Gregor Mendel Institute (GMI), Austrian Academy of Sciences 1030 Vienna Austria; ^6^ Agro R&D Sacco System 22071 Cadorago Italy

**Keywords:** 16S and ITS metabarcoding, genetic diversity, GWAS, lettuce, phosphate, plant phenotype, root microbiome, synthetic soil microbial inoculum

## Abstract

- Microbial‐based approaches offer a promising strategy to decrease the use of chemical fertilizers in agriculture. Among them, arbuscular mycorrhizal fungi (AMF), which extend root surface area and enhance phosphate uptake, and phosphate‐solubilizing bacteria (PSB) are particularly relevant. However, their effectiveness depends strongly on plant genetic diversity.
- To identify genetic markers underlying plant responses to beneficial soil microbes, we studied a panel of 128 fully sequenced *Lactuca sativa* varieties under controlled phosphate‐starvation conditions and treated with AMF and PSB.
- Lettuce genetic variation showed a strong effect on physiological and morphological responses to microbial inoculation. Genome‐wide association studies identified specific genomic regions associated with changes in leaf phosphate content and shoot biomass following treatment. Beyond genetic factors, we observed shifts in fungal β‐diversity and increased bacterial α‐diversity associated with phenotypic variation. We also identified 44 amplicon sequence variants associated with agriculturally relevant traits. Among these, six bacterial strains were experimentally validated through *in vitro* and pot experiments for their effects on leaf phosphate concentration and shoot biomass.
- Overall, we highlighted key genetic, microbial, and physiological mechanisms that may enhance microbial treatments for improved plant phosphate management in lettuce.

## Graphical abstract

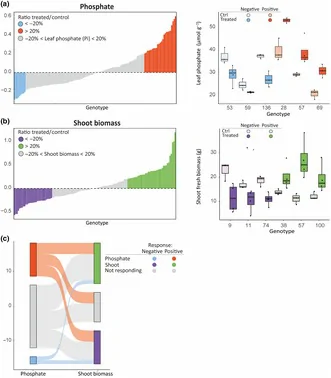

## Abstract (it)

- Gli approcci basati sui microrganismi rappresentano una strategia promettente per ridurre l'uso di fertilizzanti chimici in agricoltura. Tra questi, i funghi micorrizici arbuscolari (AMF), che aumentano la superficie radicale e migliorano l'assorbimento del fosfato, e i batteri solubilizzatori di fosfato (PSB) risultano particolarmente rilevanti. Tuttavia, la loro efficacia dipende fortemente dalla diversità genetica della pianta.
- Per identificare i marcatori genetici alla base delle risposte delle piante ai microrganismi benefici del suolo, abbiamo studiato un pannello di 128 varietà di *Lactuca sativa* completamente sequenziate, in condizioni controllate di carenza di fosfato e trattate con AMF e PSB.
- La variazione genetica della lattuga ha mostrato un effetto marcato sulle risposte fisiologiche e morfologiche all'inoculazione microbica. Studi di associazione genome‐wide (GWAS) hanno identificato specifiche regioni genomiche associate a variazioni nel contenuto di fosfato fogliare e nella biomassa dei germogli in seguito al trattamento. Oltre ai fattori genetici, abbiamo osservato cambiamenti nella β‐diversità fungina e un aumento dell'α‐diversità batterica associati alla variazione fenotipica. Abbiamo inoltre identificato 44 varianti di sequenza amplicone (ASV) associate a caratteri di rilevanza agricola. Tra queste, sei ceppi batterici sono stati validati sperimentalmente attraverso esperimenti *in vitro* e in vaso per i loro effetti sulla concentrazione di fosfato fogliare e sulla biomassa dei germogli.
- Nel complesso, abbiamo evidenziato i principali meccanismi genetici, microbici e fisiologici che potrebbero migliorare gli inoculi a base di microrganismi benefici per una gestione ottimale del fosfato in lattuga.

## Introduction

Phosphate is one of the essential macronutrients for plants, as it plays a role in many enzymatic reactions and metabolic pathways and is a key component of macromolecules like nucleic acids, ATP, and phospholipids (Chiu & Paszkowski, 2019). Although soil contains *c*. 0.05% (w/w) of phosphate, only *c*. 0.1% of this is readily available to plants due to its limited diffusion rate and tendency to be bound in inaccessible organic forms (Zhu *et al*., 2011; Sharma *et al*., 2013; Iftikhar *et al*., 2024; Kaur *et al*., 2024). As a result, phosphate is the second most common limiting macronutrient for plant growth, after nitrogen.

To address this limitation, modern agriculture still heavily depends on chemical fertilizers derived from mined rock phosphate reserves (Cordell *et al*., 2009). However, while synthetic phosphate fertilizers have helped increase crop yields, their overuse has had a negative impact on both plants and the environment. These effects include soil eutrophication, a decline in soil biodiversity, and a reduced ability of certain plants to establish symbiotic interactions, such as those with arbuscular mycorrhizal fungi (AMF) (Martín‐Robles *et al*., 2018) and nitrogen‐fixing bacteria (Kiers *et al*., 2007; Liu *et al*., 2020). Additionally, the gradual depletion of finite phosphate reserves has driven up fertilizer costs. Together, these issues highlight an urgent need for sustainable agricultural strategies that reduce reliance on chemical fertilizers.

An eco‐friendly approach gaining attention since the start of this century shifts the focus from nutrient inputs to the role of microorganisms (Capparotto & Giovannetti, 2023; Giovannetti *et al*., 2023). Throughout evolution, plants have developed beneficial associations with bacteria and fungi, particularly AMF and phosphate‐solubilizing bacteria (PSB), as their primary strategy to cope with phosphate starvation in soils (Javot *et al*., 2007; Kaur *et al*., 2024). These associations are essential for plant health and provide key ecosystem services that sustain soil health. Nevertheless, modern breeding has often prioritized traits like biomass and yield, reducing plants' responsiveness to forming symbiotic relationships (Alaux *et al*., 2024).

Thus, developing synthetic microbial communities capable of persisting in soil and functioning effectively in open‐field conditions, alongside cultivating plant genotypes that respond efficiently to interactions with these microorganisms, remains a critical focus in research and agriculture (dos Reis *et al*., 2024). Indeed, while the effectiveness of nitrogen‐fixing bacteria is well consolidated and widely applied in agriculture (Liu‐Xu *et al*., 2024; Thakur *et al*., 2024), much research is still needed to develop effective microbial inoculants that facilitate phosphate assimilation, especially in open‐field conditions (O'Callaghan *et al*., 2022). For PSB, although various studies have shown a positive impact on nutrient uptake and crop yield, this effect is often achieved only when PSB are supplemented alongside reduced doses of chemical fertilizers (Suleman *et al*., 2018; Javeed *et al*., 2019).

Additionally, there are concerns about whether they can be used across a broad range of plants or if their effectiveness is limited by species‐specific or genotype‐specific affinities. Indeed, it is well established that plant genotypes can influence responses to phosphate levels (Giovannetti *et al*., 2019) and bacterial recruitment in various plant compartments (Bulgarelli *et al*., 2013; Capparotto *et al*., 2026). Moreover, numerous studies have highlighted the impact of intraspecific plant variation on associations with plant growth‐promoting bacteria (PGPB), both in the model plant *Arabidopsis thaliana* (Plucani do Amaral *et al*., 2023) and in crops such as maize (Cuna *et al*., 2016; Vidotti *et al*., 2019; Meier *et al*., 2022) and rice (Edwards *et al*., 2015; Maghboli Balasjin *et al*., 2022). However, a clear understanding of the underlying genetic factors driving variation in plant–beneficial microbe interactions remains elusive.

In addition to the plant's ability to assimilate phosphate, the integration of other morphological parameters is essential for gaining a comprehensive understanding of the plant response to microbial inoculation. Therefore, understanding whether different phenotypic responses are linked to distinct bacterial communities in the roots is crucial for identifying advantageous combinations of genetic regions, microbial consortia, and phenotypic traits that contribute to the environmentally sustainable development of resilient plants in agriculture (Escudero‐Martinez & Bulgarelli, 2023).

In this study, we explored the role of intraspecific variation among 128 *Lactuca sativa* genotypes in determining their responses to a soil microbial inoculum. To gain a comprehensive understanding of the plant's reaction to this treatment, we integrated 15 plant morphological traits. Operating under the hypothesis that plant genotype influences the outcome of plant–microbial associations, we identified putative genomic loci associated with the observed diversity in plant responses to the microbial inoculum. Additionally, we observed that plant responses to the inoculum were associated with changes in root fungal and bacterial diversity and identified bacterial amplicon sequence variants (ASVs), likely originating from the inoculum, linked to these beneficial effects. Among the identified ASVs, we isolated six strains and validated their causal effects on variation in leaf phosphate concentration and plant shoot biomass under two different experimental conditions. Apart from the direct effects of each of these communities, we observed a potential correlation between the fungal phylum Glomeromycota and five bacterial ASVs involved in regulating key nutritional parameters. This suggests that fungi, through their intraradical and extraradical hyphal network, may represent a niche for bacteria with significant effects on plants.

Our findings provide a foundation for understanding the genetic and phenotypic basis of lettuce–soil microbe interactions, particularly in facilitating nutrient acquisition, and guide the identification and isolation of new effective microbes.

## Materials and Methods

### Experimental design

In this study, 131 fully sequenced *Lactuca sativa* L. (lettuce) genotypes were provided by the Centre for Genetic Resources (CGN, Wageningen, Netherlands; Wei *et al*., 2021). Plant germination and growth took place in glasshouse conditions at LandLab S.r.l. in Quinto Vicentino, Vicenza (Italy). Before starting the main experiment, a pilot study was conducted to determine the optimal phosphate concentration that would induce a state of starvation without compromising the plants' viability (Supporting Information Table S1). 131 lettuce genotypes were sown in seed trays in 12 replicates each and germinated in a growth chamber for *c*. 1 month. During this initial growth phase, the plants were fertigated with a nutritional solution containing NPK 18/18/18 to support their development. To simulate phosphate starvation, plants were grown in sand as a medium and supplemented with triple superphosphate (TSP) at a rate of 32.61 kg ha^−1^ (or 0.33 g per plant), equivalent to 15 kg ha^−1^ of P_2_O_5_.

The procedure involved filling each pot (15 cm × 15 cm × 20 cm) with 3 l of nonsterilized soil–sand mixture, adding the pre‐weighed TSP, and then topping it up with an additional 1 l of soil–sand to complete the pot‐filling process. A mesh pad (30 g m^−2^) with holes was placed at the base of each pot to prevent water spillage. Five weeks after germination, a total of 866 young seedlings were transplanted into the previously prepared pots in the glasshouse and randomized positions as outlined in Table S2. Three genotypes (TKI‐93, TKI‐23, TKI‐121) that did not germinate sufficiently to achieve the required number of replicates for the experiments were excluded from the analysis, resulting in a total of 128 genotypes being considered. Four replicates per genotype were treated with 50 mg of microbial inoculum, applied as a 50 ml aqueous solution directly to the plant rootlet, at a concentration comparable to that used under field conditions. The synthetic microbial community used was formulated by Sacco System, located in Cadorago, Como (Italy). This community comprised two strains of AMF (*Rhizophagus irregularis* and *Funneliformis mosseae*) in a concentration of 1000 propagules g^−1^ and two strains of PSB (*Bacillus subtilis* and *Chryseobacterium indoltheticum*) in a concentration of 1 × 10^9^ CFU g^−1^ each. These microorganisms were isolated for their capacity to enhance phosphorous availability and promote plant growth. Because arbuscular mycorrhizal (AM) fungi are obligate symbionts, their propagules were introduced in the form of root fragments enriched with the desired fungal species, which may also contain additional fungal and bacterial taxa. The microbial inoculum had a shelf life of 2 yr, as stated by the company, thereby covering the entire duration of the experiment (3 months). To support plant growth, periodic fertigation was implemented at a concentration of N‐K_2_O 250, equivalent to 76.92 kg ha^−1^. Detailed information regarding dates and concentrations of fertigation are included in Table S3.

### Plant phenotyping

Throughout the entire experiment, plant growth, phenotypic appearance and physiology of each plant were recorded twice daily using a noninvasive, high‐throughput phenotyping platform, the PlantEye (PlantEye F600; Phenospex, Heerlen, the Netherlands), provided by the LandLab company. The analysis combined 3D imaging with multispectral imaging. In this study, the following parameters were analyzed:

1. Greenness/Green Leaf Index (no units; Eqn 1): The ratio of green reflectance to red and blue reflectance. It indicates the combined effects of leaf physiology and canopy structure.
(Eqn 1) $$\text{Greenness}=\left(2\times \text{Green}–\mathrm{Red}–\text{Blue}\right)/\left(\mathrm{Red}+\text{Green}+\text{Blue}\right)$$
2. Hue (°): Represents the visible color determined by the wavelength of reflected light, independent of saturation and brightness. It indicates photosynthetic pigment levels and nutrient concentration in leaves.
3. Digital Biomass (cm^3^; Eqn 2): The volume of the plant's aboveground biomass, indicating plant productivity.
(Eqn 2) Digital Biomass=Height×3D Leaf Area
4. Height (mm): The average height of the community's plants, from the ground to the top 10% of plants. It is an indicator of plant productivity, nutrient availability, light competition, and growth strategy.
5. Leaf area (cm^2^): The three‐dimensional leaf area, corrected for leaf inclination, indicating heat load, water retention, and gas exchange.
6. Leaf area index (cm^2^/cm^2^; Eqn 3): The leaf area per unit ground area, related to photosynthetic activity and respiration.
(Eqn 3) Leaf Area Index=Leaf Area/Sector Size
7. Light penetration depth (mm): The deepest point the laser can penetrate through the canopy, indicating vegetation density and resource availability.
8. Normalized Digital Vegetation Index (NDVI, no units; Eqn 4): The ratio of near‐infrared (NIR) to red reflectance, indicating vegetation type, productivity, and growth parameters like leaf area index and biomass.
(Eqn 4) $$\text{NDVI}=\left(\mathrm{NIR}–\mathrm{Red}\right)/\left(\mathrm{NIR}+\mathrm{Red}\right)$$
9. Normalized Pigments Chlorophyll Ratio Index (NPCI, no units; Eqn 5): The ratio between the blue and red channels, associated with Chl content and indicating the plant's physiological state.
(Eqn 5) $$\text{NPCI}=\left(\mathrm{Red}–\text{Blue}\right)/\left(\mathrm{Red}+\text{Blue}\right)$$
10. Plant Senescence Reflectance Index (PSRI, no units; Eqn 6): The ratio of red and green to near‐infrared reflectance, associated with carotenoid and Chl content, indicating leaf senescence.
(Eqn 6) $$\text{PSRI}=\left(\mathrm{Red}–\text{Green}\right)/\mathrm{NIR}$$

Since daytime measurements could be influenced by various confounding factors, only the nighttime recordings were used for the analysis presented in this paper. The scanning sequence for PlantEye is detailed in Table S4, and measurements for each plant replicate are provided in Table S5.

### Plant sampling

Leaf sample collection took place 5 wk after transplanting (December 14 and 15, 2022). The procedure involved weighing the aerial part of the plant before leaf sampling with a portable balance (sensitivity = 0.01 g). Each leaf was sampled using a single‐hole punch to obtain three leaf disks with a diameter of 3 mm from each leaf. These leaf disks were then combined with those obtained from two additional leaves of the same plant, resulting in a total of 9 leaf disks per replicate. Simultaneously, roots were washed to eliminate soil residues, dried using absorbent paper, and weighed with the same portable balance to obtain their fresh weight. The root and shoot fresh weights are reported in Table S6. Subsequently, all treated roots and a few control ones were sampled for metabarcoding analysis. Specifically, a bundle of *c*. 2 cm in length roots (excluding the primary roots) was collected. Both leaf and root samples were gathered in racked 96 collection microtubes (Qiagen), following the order outlined in Table S7. In addition to these, the soil used for plant germination and the sand used in the experiment mixed with the water used for watering the plants were also collected and used as controls. The tools used for sampling were washed with 70% ethanol and distilled water between each accession. All the collected materials, except for the soils, were stored at −80°C before undergoing lyophilization for long‐term storage.

### Inorganic phosphate quantification

Lyophilized leaf disks were weighed using an ultra‐analytical balance (sensitivity = 0.01 mg) to achieve a final sample weight between 0.25 and 0.80 mg. The disks were then freeze‐dried and ground into a fine powder using a TissueLyzer (Qiagen) before being resuspended in NanoPure water and incubated at 98°C for 1 h. The final suspension, obtained after centrifugation, was used for sample processing with the phosphate assay kit (MAK308; Sigma‐Aldrich), following the instruction manual with some modifications. Briefly, 16 μl of the sample suspension (and separately 16 μl of the standard solution) was mixed in replicates with 32 μl of Malachite Green Reagent. After a 30‐min incubation period, absorbance was measured at 620 nm using a Tecan spectrophotometer (Trading AG, Männedorf, Switzerland). Using the absorbance and intercept values, we calculated the inorganic soluble phosphate concentration, expressed as μmol g^−1^. The phosphate quantification table is detailed in Table S8.

### Plant phenotypic response and metabolites quantification

For each parameter measured by Phenospex, the overall plant response to the inoculum was determined by calculating the ratio between the median values of the treated and control samples for each genotype. The data were then visualized in R by applying *z*‐scaling and creating a heatmap using the pheatmap package (v.1.0.12) as well as through PCA with the factominer (v.2.11) and factoextra (v.1.0.7) packages. From the representation of the first and second principal components (PCs), we identified four groups of genotypes that differently responded based on their positions at the extremes of the phenotypic space and their locations within the four quadrants around the analysis vectors. This process led to the selection of 28 genotypes showing contrasting responses, which were subsequently confirmed through metabolite quantification using the rainbow protocol, with minor modifications (López‐Hidalgo *et al*., 2021). In brief, lyophilized leaf disks were weighed using an ultra‐analytical balance (sensitivity = 0.01 mg) to ensure a final sample weight of at least 1.30 mg. The disks were then freeze‐dried, ground into a fine powder using a TissueLyzer, and suspended in 600 μl of 80% ethanol. For photosynthetic pigments (PHP) quantification, 200 μl of the supernatant was diluted 1 : 2. All subsequent steps for free amino acids (FAA), total soluble sugars (TSS), and starch (STA) analyses were performed according to the published protocol. Their final concentrations were expressed in μl ml^−1^. The metabolite quantification table is detailed in Table S9.

### Genome‐wide association mapping

To associate plant physiological variation in lettuce, we performed a genome‐wide association study (GWAS) using the *Lactuca sativa* single nucleotide polymorphism (SNP) matrix. Analyses were conducted in Visual Studio Code (v.1.94.2) with the GEMMA software package (v.0.98.5; Zhou & Stephens, 2012), employing a linear mixed model. Phenotypic traits were defined as the plant response to microbial treatment, calculated as the ratio between the median trait values of treated and control plants for each genotype. SNPs with a minor allele frequency below 0.05 were excluded. To account for population structure, the first five PCs, obtained via PCA using PLINK2, were included as covariates in the model. A centered relatedness (kinship) matrix was computed in GEMMA using the standardized relatedness estimator and incorporated as a random effect to correct for genetic relatedness among genotypes. Association analysis was conducted with GEMMA's linear mixed model applying restricted maximum likelihood estimation.

The full analysis script is available on Github (https://github.com/picla/salad_marco/tree/master/003.gwas/002.scripts). Broad‐sense heritability for each trait was estimated in R using the ANOVA method.

### DNA extraction and sequencing

Ninety‐one root samples, including six controls and 85 treated samples from 22 genotypes, were selected for 16S and ITS sequencing based on their metabolite clustering and phenotypic responses to the inoculum (Table S10). In addition, four samples each from the inoculum, germination soil, and growth soil (sand + water) were included as environmental matrix controls. DNA extractions were performed using the CTAB protocol with some modifications. Briefly, *c*. 3.5 mg of lyophilized root sample was ground using a TissueLyzer (Qiagen) and then resuspended in 600 μl of CTAB, followed by incubation at 60°C for 1 h. After this step, 600 μl of phenol/chloroform/isoamyl alcohol (25 : 24 : 1) was added twice, mixing and removing impurities each time. The remaining supernatant was treated with ice‐cold isopropanol and incubated at −20°C for 2 h. DNA was then precipitated by centrifugation, washed with 70% ethanol, and resuspended in NanoPure water. Inoculum and sand with water samples were extracted using the same method, whereas the germination soil samples were extracted using the DNeasy® PowerSoil® kit (Qiagen) according to the manufacturer's instructions. To further purify the samples and prepare them for PCR amplification, some were processed using the OneStep PCR Inhibitor Removal Kit (Zymo Research, Irvine, CA, USA). DNA from all 103 samples was quantified using Qubit™ dsDNA HS (High Sensitivity) Assay Kits from Thermo Fisher Scientific. Sample information and quantification values are detailed in Table S11. Subsequently, the DNA samples underwent amplicon sequencing of the 16S V5–V7 regions for bacteria and the ITS2 region for fungi using the MiSeq Illumina platform at NOVOGENE (UK). For bacteria, the V5–V7 region was amplified using the primer pair 799F (5′‐AACMGGATTAGATACCCKG‐3′) and 1193R (5′‐ACGTCATCCCCACCTTCC‐3′). For fungi, the ITS2 region was amplified with the primer pair ITS3F (5′‐GCATCGATGAAGAACGCAGC‐3′) and ITS4R (5′‐TCCTCCGCTTATTGATATGC‐3′).

### Raw reads processing

Raw sequencing reads were processed using Dada2 (v.1.30.0) in R (v.4.3.3). For ITS analysis, only the forward reads were used as described by Pauvert *et al*. (2019). Barcodes and primer sequences were removed before filtering, trimming, and merging the forward and reverse reads. The merged sequences then underwent chimera removal and taxonomic assignment using the Silva database (v.138; Callahan *et al*., 2016) for bacteria and the UNITE dynamic database (v.18.07.2023) for fungi. The dataset was further refined to remove chloroplast, mitochondrial, eukaryotic, and archaeal contaminants. For bacteria, only ASVs with a relative abundance > 0.01% were retained, reducing the number of ASVs from 45 316 to 35 043, while keeping all 103 samples. For fungi, samples with fewer than 100 counts and with a relative abundance lower than 0.01% were excluded, reducing the number of ASVs from 7092 to 7067 and removing five samples. After applying the same abundance threshold used for bacteria, the fungal ASVs were further reduced to 6022. All bacterial samples underwent size‐based rarefaction, set at 41 736 reads, matching the minimum read count among the samples. This process was performed in R with the use of vegan package (v.2.6.4). For fungi, due to large differences between samples, rarefaction was performed per compartment: sand + water samples were rarefied at 42 893 reads, germination soil at 29 357, inoculum at 70 255, and root samples at 1000 reads. This process reduced the number of root samples from 86 to 50, with a final total of 62 samples. The number of samples and ASVs lost at each step reported above is detailed in Table S12. Finally, to check for correlations between the fungal and bacterial communities, both root datasets were rarefied to the minimum sampling depth, corresponding to 1000 reads.

### Evaluation of AM colonization

The entire root systems of seven different genotypes (totaling 46 samples), chosen randomly during sampling, were collected, and processed to assess the degree of AM colonization using the Trouvelot method (Trouvelot, 1986). Roots were first cleared by incubation in 10% KOH at 96°C in a water bath for 10 min. Following incubation, the KOH solution was discarded, and roots were washed twice with 5% lactic acid. Subsequently, roots were stained in a solution containing 5% black ink and 5% lactic acid at 96°C for 11 min. After staining, roots were washed repeatedly in 5% lactic acid until the solution became clear. From each biological replicate, five microscope slides were prepared, each containing 20 root fragments of *c*. 1 cm in length. Therefore, *c*. 100 cm of root tissue was analyzed per sample.

### Bacterial tests on lettuce plant growth

Six bacterial strains were selected from a representative collection of 105 lettuce root‐associated isolates (doi: 10.5281/zenodo.19664961) based on their genetic similarity to ASVs significantly associated with leaf phosphate content in lettuce roots (see the Positively responding plants show enrichment of differentially abundant ASVs from Burkholderiales and Flavobacteriales section). Briefly, the V5–V7 hypervariable regions of the 16S rRNA gene (see the DNA extraction and sequencing section) of the collection isolates were assembled into a single‐fragment contig and aligned against the 22 responsive ASV sequences using VSEARCH v.2.17.0 (Rognes *et al*., 2016), applying a minimum sequence identity threshold of 97%. Full alignment results, including percentage identity, query sequences, CIGAR notation, and taxonomic assignment, are reported in Table S13.

These strains were used to inoculate three lettuce genotypes (TKI‐24, TKI‐38, and TKI‐100) in two experimental setups: a plate assay and a pot assay in a growth chamber.

Each strain was grown individually in 50% Tryptic Soy Broth (cat: 1010717; MP Biomedicals, Santa Ana, CA, USA) for 48 h at 28°C, after which cultures were adjusted to a final OD_600_ of 0.05. A synthetic community (SynCom) was generated by combining the six strains in equimolar proportions. As a mock control, a 10 mM MgCl_2_·6H_2_O solution (cat: 63033; Merck, Darmstadt, Germany) was used.

For the plate assay, lettuce seeds were surface‐sterilized by immersion in 0.5% NaOCl (1 ml per 100 seeds) for 11 min, followed by five 1‐min rinses with sterile water. Seeds were then pregerminated in 12 × 12 cm^2^ plates (cat: 688161; Greiner Bio‐One, Kremsmünster, Austria) containing Long‐Ashton nutrient solution solidified with agar (Table S14). After 2 d of incubation in the dark, germinated seeds were transferred to fresh square plates. At the time of transfer, four seedlings per plate were inoculated by applying 0.5 ml of bacterial suspension or mock solution directly to the roots. Plates were allowed to dry for 30 min before sealing. For each condition, four replicate plates were prepared, each containing four plants. Fresh shoot biomass and phosphate content were assessed 9 d after transfer and inoculation.

For the growth‐chamber pot assay, 0.3 l pots (7 cm × 7 cm × 7 cm) were fitted with a mesh at the bottom and filled with fine quartz sand sterilized by dry heat at 120°C for 3 h. Three surface‐sterilized seeds were placed equidistantly in each pot, and 1 ml of the bacterial suspension or mock solution was dispensed directly onto the seeds, which were then covered with *c*. 1 cm of sand. Plants were subirrigated with Long‐Ashton nutrient solution containing 32 μM phosphorus (Table S14). Four days after sowing, seedlings were thinned to one plant per pot. Ten pots were used for each treatment. Fresh shoot biomass was assessed 40 d after thinning.

### Statistical analysis

Each genotype's response to the inoculum was calculated as follows (Eqn 7):

(Eqn 7) $$\text{median}\;\left(\text{treated replicates}\right)÷\text{median}\;\left(\text{control replicates}\right).$$

The response of individual replicates to the inoculum was calculated as follows (Eqn 8):

(Eqn 8) $$\text{Treated sample}÷\text{median}\;\left(\text{control replicates}\right).$$

Differences between treated and control genotypes were assessed using the Kruskal–Wallis test, followed by Dunn's *post hoc* test (fdr adjustment for multiple corrections). Plants were categorized as either ‘positive’ or ‘negative’ responders to the inoculum for each phenotypic trait analyzed. Specifically, if the ratio of the treated plants to the median of control plants for a given genotype exceeded 1.20, the genotype/plant was classified as a positive responder. Conversely, if the ratio fell below 0.80, it was categorized as a negative responder. Genotypes/plants with a ratio between 0.80 and 1.20 were considered ‘non‐responding’. This classification allowed us to identify three distinct conditions for phosphate accumulation and shoot biomass, which were used to account for differences in bacterial composition and diversity. The phyloseq (v.1.48.0), vegan, reshape2 (v.1.4.4), and ggplot2 (v.3.5.1) packages in R were utilized to generate stacked bar plots of bacterial communities and to perform both β‐diversity analysis (using the Bray–Curtis distance matrix) and α‐diversity analysis (Shannon and Observed indexes). Statistical differences in α‐diversity between phenotypic response groups were assessed through the Kruskal–Wallis test, followed by Dunn's *post hoc* test (fdr adjustment for multiple corrections). The Log_2_foldchange function from the deseq2 package in R (v.1.44.0) with correction for multiple comparisons was used to examine differences in bacterial ASV presence between conditions of positive and negative phosphate and root biomass responses. Broad sense heritability for differentially abundant ASVs in leaf phosphate and root biomass variation was calculated in R with the Imer method from the ime4 package (v.1.1.37). Pearson correlations from the *cor.test* function in the ggpubr package in R (v.0.6.0) were used to study correlations between the taxonomic ranks of fungal and bacterial communities.

## Results

### Physiological and nutritional variability across 128 *Lactuca sativa* accessions depend on genotypes

To broadly assess the impact of an inoculum containing arbuscular mycorrhizal fungi (AMF) and PSB, 128 fully sequenced *Lactuca sativa* accessions were cultivated in pots under low‐phosphate conditions, either with or without the inoculum treatment. Although all the plants were grown in a nonsterile soil–sand mixture, those inoculated showed a tendency toward higher mycorrhization rates compared with untreated plants (Fig. S1; Table S15). Plant responses were first evaluated across two key agricultural parameters: leaf‐soluble phosphate concentration and shoot biomass variation. Results showed substantial variation in responses across genotypes, with many genotypes demonstrating positive responses – considered 20% greater than nonresponders in both leaf soluble phosphate accumulation (25 genotypes; Fig. 1a) and shoot biomass variation (28 genotypes; Fig. 1b; Tables S16, S17) consistently with growth curves obtained with phenospex data (Fig. S2). Only nine genotypes exhibited a decrease in leaf phosphate, whereas 30 genotypes showed a reduction in shoot biomass due to the treatment (Fig. 1a,b). These findings highlight the strong intraspecific variation driving divergent responses among genotypes within the same plant species, an observation that is further reinforced by the high consistency in variation between treated vs mock replicates (Fig. 1a,b, right side).

**Fig. 1:**
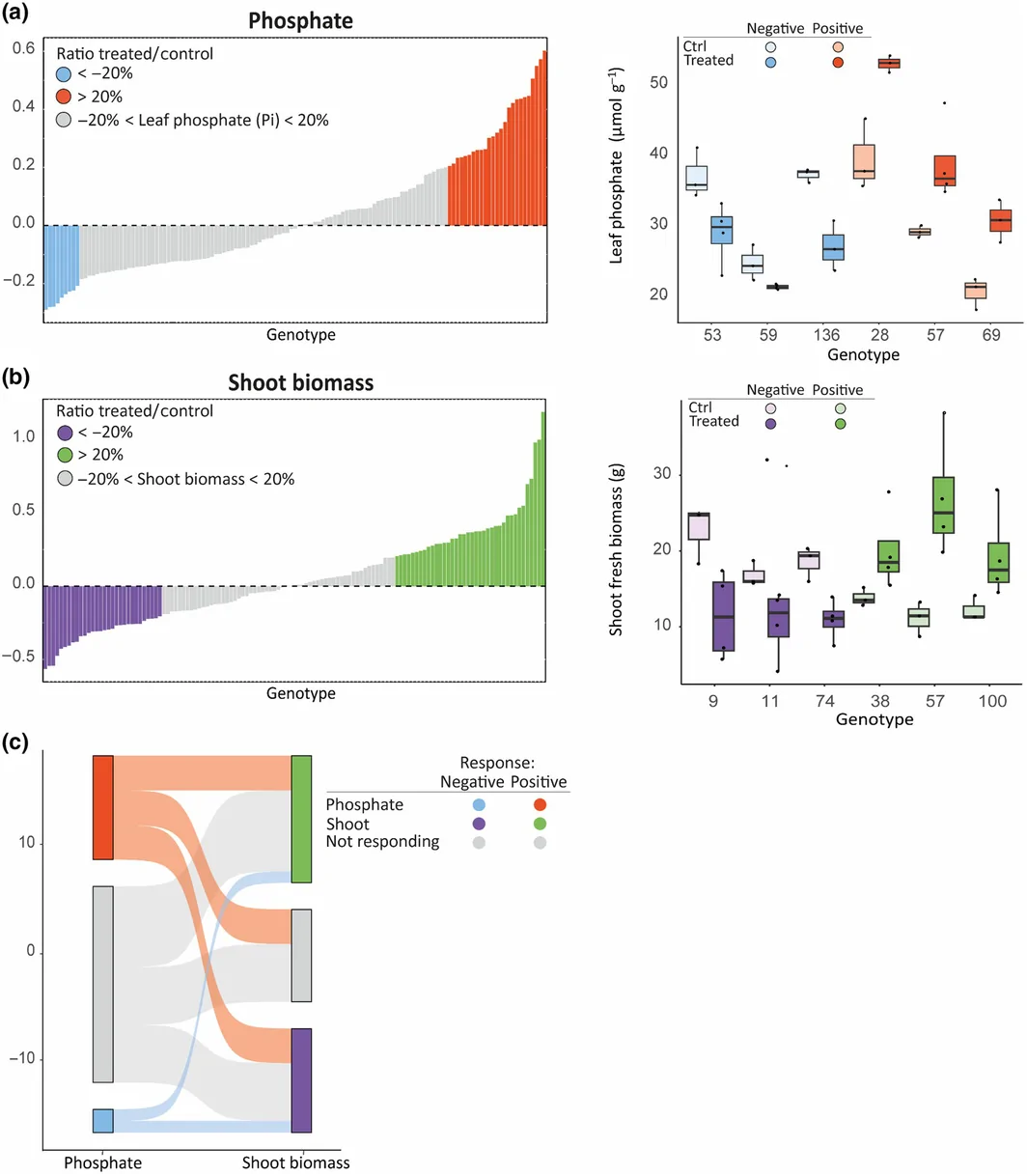
Lettuce leaf phosphate and shoot biomass variation in response to the microbial consortium. (a, b) Bar plots depicting genotype responses in terms of leaf phosphate accumulation (a, left) and shoot biomass variation (b, left). In panel (a), *y*‐axis values are expressed as median (Pi)treated/median (Pi)control, whereas in panel (b), they represent median treated shoot biomass (g)/median control biomass (g). Genotypes with a 20% or greater increase in response are highlighted in red (a) and green (b), whereas those with a 20% or greater decrease are shown in blue (a) and purple (b), genotypes within the middle range are represented in grey. The boxplots on the right side of panels (a) and (b) display representative genotype responses for phosphate (a, right) and shoot biomass (b, right) using the same color scheme for positive (red/green) and negative (blue/purple) responses. In the boxplots, the horizontal line represents the median, box edges indicate the 25^th^ and 75^th^ percentiles, whiskers extend to 1.5× the interquartile range (IQR), and individual data points represent biological replicates. (c) A Sankey diagram illustrates genotype response shifts due to the inoculum for phosphate accumulation (left) and shoot biomass variation (right), following the same color coding. Flow width is proportional to the number of genotypes transitioning between response categories.

Interestingly, the fluctuations of these two parameters appeared independent of each other (Fig. 1c). Specifically, among the 25 plants that accumulated more phosphate, six showed an increase in shoot biomass, six showed a decrease, and 13 exhibited no change. Similarly, among the nine plants that accumulated less phosphate, four displayed an increase in biomass, whereas two showed a decrease (Fig. 2a). By considering all measured morphological and physiological plant parameters in response to the treatment, we identified two main groups of response (Fig. 2a). The analysis revealed a strong correlation between spectral parameters, such as hue and greenness, and various physiological indicators, including NCPI, PSRI, and NDVI. By contrast, morphology‐related traits, such as digital biomass, shoot and root fresh biomass, and leaf area, formed a distinct cluster characterized by a more uniform response pattern. Interestingly, while some physiological traits (spectral parameters like NCPI, PSRI, and NDVI) were strongly correlated, the morphological response was highly variable and often uncoupled, as for the case of shoot biomass and phosphate accumulation mentioned above. Along the *y*‐axis, three distinct response clusters emerged, independent of plant variety.

**Fig. 2:**
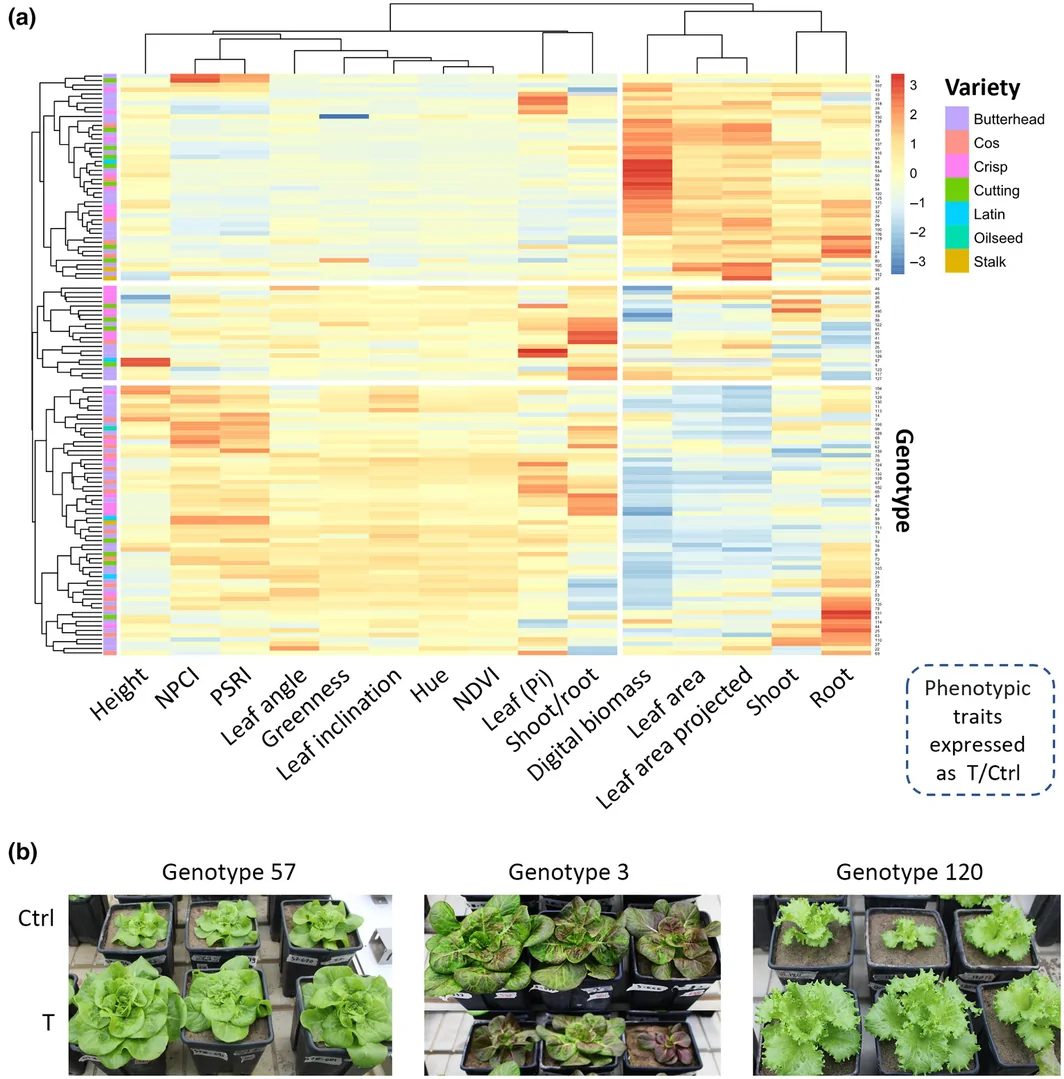
Overall phenotypic and physiological lettuce responses to the soil microbial inoculum. (a) Hierarchical clustered heatmap of *z*‐scaled genotype responses, expressed as median (treated)/median (control) for each trait. Traits included are plant height (cm), NPCI (Normalized Pigment Chlorophyll Index), PSRI (Plant Senescence Reflectance Index), leaf angle (°), greenness, leaf inclination (°), hue, NDVI (Normalized Difference Vegetation Index), leaf phosphate content ((Pi), μmol g^−1^), shoot/root (fresh biomass ratio), digital biomass (mm^3^), leaf area (cm^2^), projected leaf area (cm^2^), shoot fresh biomass (g), and root fresh biomass (g). High values are shown in red, low values in blue, and intermediate values in white. A filled rectangular shape on the left indicates the varieties to which the genotypes belong. (b) Images of three representative genotypes, with control (Ctrl) plants above and treated (T) plants below.

For some genotypes, phenotypic variation resulting from inoculation was visibly apparent by the end of the experiment, as shown in Figs 2(b) and S2. Notably, genotype 57 stood out as a positive responder due to the pronounced phenotypic differences between mock and treated plants, coupled with a significant increase in leaf‐soluble phosphate (Dunn *post hoc*, *P*‐value = 0.034), as highlighted in Fig. 1(a).

Together, these results suggest that lettuce genetic variation plays a key role in shaping the outcomes of beneficial plant–microbe interactions at the soil–root interface.

### Phosphate and shoot biomass responses to the microbial inoculum are linked to three SNPs in the lettuce genome

To better understand how genetic variation influences plant responses to inoculation, we conducted a GWAS to explore potential associations between lettuce SNPs and various phenotypic traits (Figs 3, S3, S4). Due to high correlations among some traits, four phenotypic parameters (leaf area index, leaf area projected, light penetration depth, and maximum height) were excluded to streamline the dataset (Fig. S3a,b).

**Fig. 3:**
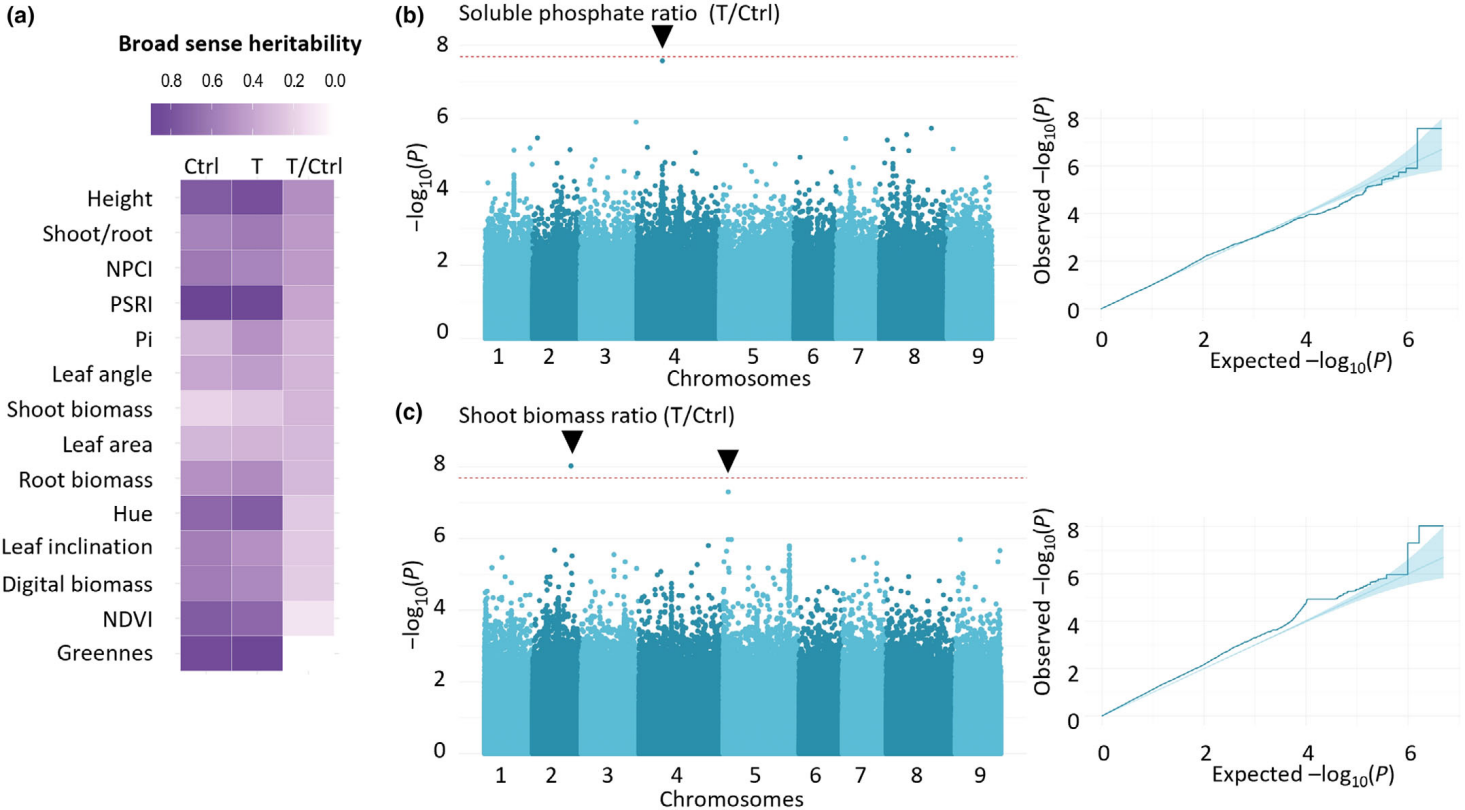
Genetic basis of the nutritional and physiological response of 128 *Lactuca sativa* genotypes to a microbial consortium. (a) Heatmap displaying broad‐sense heritability (*H* ^2^) values calculated for control (Ctrl) and treated (T) plants, as well as their ratio (T/Ctrl), across the measured traits. Color intensity indicates the magnitude of the values, as shown in the scale bar. (b, c) On the left hand, Manhattan plots show the genome‐wide association between lettuce SNPs (single nucleotide polymorphisms) and variations in (b) leaf‐soluble phosphate ratio and (c) shoot biomass ratio. On the right, quantile–quantile (QQ) plots illustrate expected vs observed −log_10_(*P*) values.

We calculated broad‐sense heritability (*H* ^2^) for traits of treated plants, control plants, and their ratio (reflecting the response to inoculation; Fig. 3a; Table S18). PSRI had the highest heritability for both control (*H* ^2^ = 89.62%) and treated (*H* ^2^ = 87.18%) plants, while shoot biomass had the lowest heritability for control (*H* ^2^ = 19.05%) and treated (*H* ^2^ = 24.67%) plants. Overall, heritability decreased for plant response (ratio) compared to the control and treated conditions. The highest *H* ^2^ for plant response was observed for head height (*H* ^2^ = 50.97%), whereas the lowest was for greenness (*H* ^2^ = *c*. 0). Notably, shoot biomass variation (T/Ctrl, *H* ^2^ = 32.56%) and soluble leaf phosphate variation (T/Ctrl, *H* ^2^ = 33%) were among the few traits where *H* ^2^ increased compared to both control and treated plants (Fig. 3a).

For soluble leaf phosphate, we identified a highly significant SNP on chromosome 4 (position: 117030769, allele frequency = 32.3%, *P*.wald = 6.79e‐08; Fig. 3b). Additionally, two SNPs, one on chromosome 2 (position: 22753367, allele frequency = 8.3%) and another on chromosome 5 (position: 172536404, allele frequency = 49.2%), were significantly associated with shoot biomass variation (*P*.wald = 2.56e‐08 and 1.43e‐08, respectively; Fig. 3c; Table 1).

**Table 1:** List of single nucleotide polymorphisms significantly associated with phenotypic and physiological traits.

Phenotypic trait	Heritability	Chromosome position	Chromosome	Allelic frequency (%)	*P*‐wald
Pi T/Ctrl	0.323	117030769	4	32.3	6.79E‐08
Shoot biomass T/Ctrl	0.326	22753367	2	8.3	2.56E‐08
Shoot biomass T/Ctrl	0.326	172536404	5	49.2	1.43E‐08
Head height T/Ctrl	0.51	22753358	5	49.6	1.08E‐24
Head height T/Ctrl	0.51	36408738	5	49.6	1.08E‐24
Head height T/Ctrl	0.51	36408739	5	49.6	1.08E‐24
Head height T/Ctrl	0.51	21725555	9	49.6	1.08E‐24
Head height T/Ctrl	0.51	295749109	5	50.8	4.76E‐13
Head height T/Ctrl	0.51	66640473	1	49.2	6.19E‐12
Head height T/Ctrl	0.51	209962003	2	49.2	6.19E‐12
Head height T/Ctrl	0.51	209962010	2	49.2	6.19E‐12
Head height T/Ctrl	0.51	209962035	2	49.2	1.73E‐11
Head height T/Ctrl	0.51	22753367	5	49.2	6.67E‐11
Head height T/Ctrl	0.51	85252515	3	49.2	1.65E‐11
NPCI	0.455	74322621	3	5.5	2.44E‐08
NPCI	0.455	115636353	8	5.1	2.72E‐08
NPCI	0.455	20535263	9	5.1	3.84E‐08
NPCI	0.455	20602606	9	5.5	4.55E‐08
PSRI	0.404	49514559	8	11.2	2.79E‐08

For other traits, GWAS was performed on the response (T/Ctrl) parameters with *H* ^2^ > 20% (Fig. S4). This analysis showed no significant SNP associations for the shoot/root ratio (Fig. S4a), leaf angle ratio (Fig. S4e), and root ratio (Fig. S4f). However, several significant SNPs associated with NPCI (Fig. S4c) and PSRI (Fig. S4d) were identified. On the other hand, head height (Fig. S4b) displayed highly polygenic patterns. Table 1 provides a comprehensive list of all significant SNPs identified in this analysis, with further details and raw data reported in Table S19 available at Zenodo (zenodo.org/records/14224765).

These findings highlight the complex genetic architecture underlying lettuce's response to inoculation.

### Integrating plant phenotypic and metabolic traits allows a detailed clustering of inoculum effects on plants

To identify clusters of genotypes that showed an overall similar plant phenotypic response to the inoculum, we conducted PCA on the trait ratios for each genotype (T/Ctrl; Figs 4, S5). Given the strong correlations among certain phenotypic traits (Fig. S5a), we used only a few independent and highly descriptive ones to define the phenotypic space of the genotypes (Fig. 4). This approach effectively separated physiological parameters (NPCI, PSRI, Hue, NDVI) from morphological ones (shoot and root biomass and their ratio). Notably, the leaf phosphate vector is orthogonal to shoot biomass, nearly overlapping with the shoot/root ratio, while the phosphate and root vectors point in opposite directions, reflecting plant physiological responses to phosphorus availability or deficiency.

**Fig. 4:**
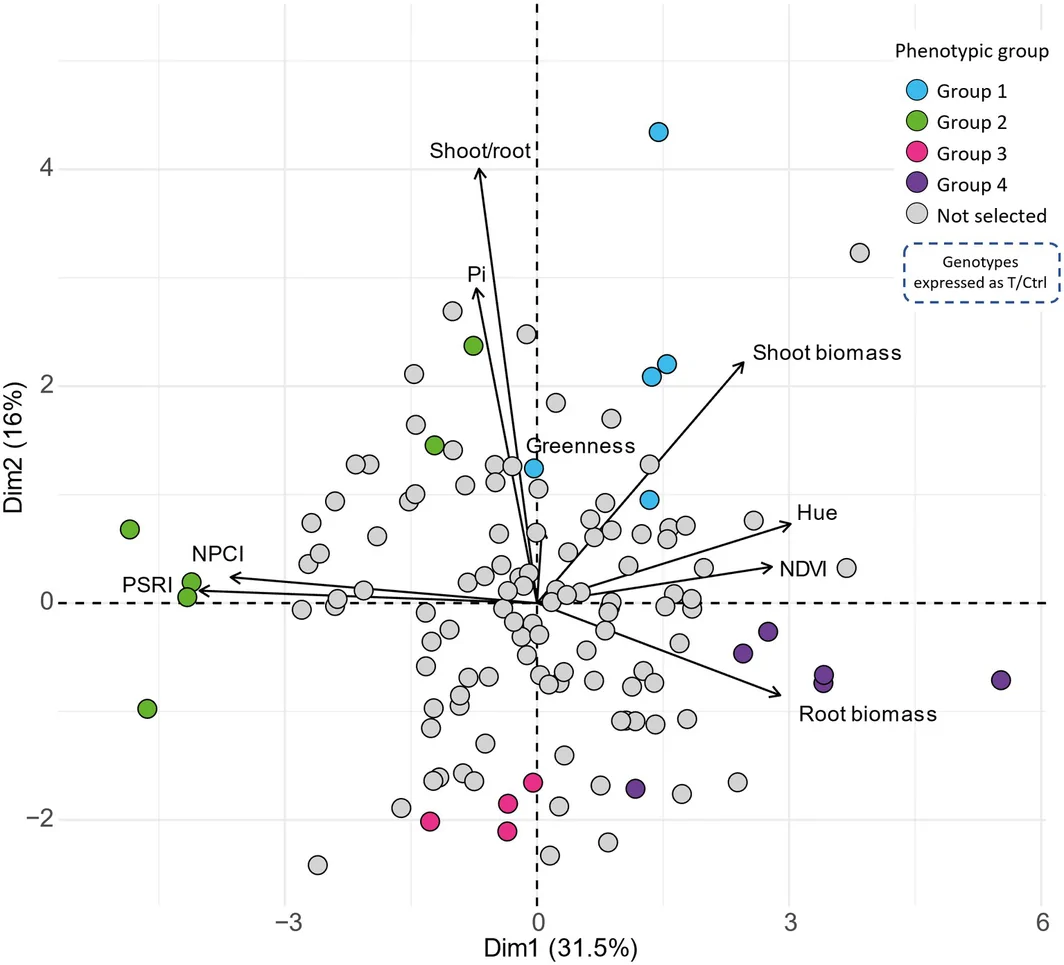
Multiple phenotypes integration disentangles lettuce response to the microbial inoculum. PCA of genotype responses to inoculum treatment, expressed as median (treated values)/median (control values). Genotypes chosen for metabolite quantification, representing the four distinct phenotypic groups, are color‐coded as blue, green, pink, and purple. Non‐selected genotypes are shown in grey. Arrows indicate the loading vectors of the traits contributing to the principal components, with direction and length representing the strength and orientation of each trait's contribution to the two dimensions shown; trait labels are as follows: PSRI (Plant Senescence Reflectance Index), NPCI (Normalized Pigment Chlorophyll ratio Index), NDVI (Normalized Difference Vegetation Index), Pi (leaf phosphate content, μmol g^−1^), Greenness, Shoot/root (fresh biomass ratio), Shoot biomass (g), Root biomass (g), and Hue.

In summary, this method identified four distinct response groups across the lettuce population capturing the plant phenotypic variation in response to microbial inoculation. Among these clusters, we selected 28 genotypes to further analyze their leaf metabolites (Fig. S5b), which confirmed the initial classification and led to the exclusion of six genotypes that did not fit into any group. The remaining 22 genotypes, represented by 91 plants occupying diverse plant phenotypic space, were used for downstream analysis, with the hypothesis that plants with similar responses to the inoculum exhibit analogous microbial interactions.

### The four phenotypic response groups are associated with diverse root‐associated microbial communities

To explore the relationship between plant phenotypic responses and root microbial communities, we analyzed the fungal and bacterial community composition across all substrates that were used in our experimental setup. The growing medium (sand and water) exhibited the highest bacterial Shannon and Observed diversity, while roots displayed the lowest diversity, followed by germination soil and microbial inoculum (Figs S6a–c, S7a–c). Although the inoculum's richness was similar to or lower than that of the soils, it enhanced the Shannon diversity index in treated plants compared to controls (Figs S6a,b, S7a–c). Additionally, each compartment had a distinct bacterial composition (Figs S6d, S7d). The dominant bacterial phyla across all compartments were Proteobacteria and Actinobacteriota, while for fungi Ascomycota predominated (Table S20a,b). The inoculum showed enrichment in Firmicutes (12% in inoculum compared to 5% in roots, 2% in growth soil, and 0% in germination soil) and Bacteroidota (6% in inoculum, 5% in roots, 4% in growth soil, and 3% in germination soil) (Table S20a). Notably, an amplicon sequence variant corresponding to *Chryseobacterium indoltheticum* was detected in plant roots and assigned to ASV1713 (Table S21). The Glomeromycota phylum, to which *Rhizophagus irregularis* and *Funneliformis mosseae* belong, was present in the roots and in the inoculum but not in the other compartments (Table S20b).

To achieve greater resolution in individual plant responses, we examined root‐associated bacterial diversity at a single‐replicate level, as described in the Materials and Methods section (Eqn 8) and Table S10. This analysis showed statistically significant differences in fungal β‐diversity (PERMANOVA, *P*‐value = 0.018, Fig. 5a) among the four previously identified phenotypic groups, but not in their α‐diversity (Fig. S8a). For bacteria, instead, no significant differences were observed in the bacterial Shannon and Observed α‐diversity indices among the four previously identified phenotypic groups (Fig. S8b).

**Fig. 5:**
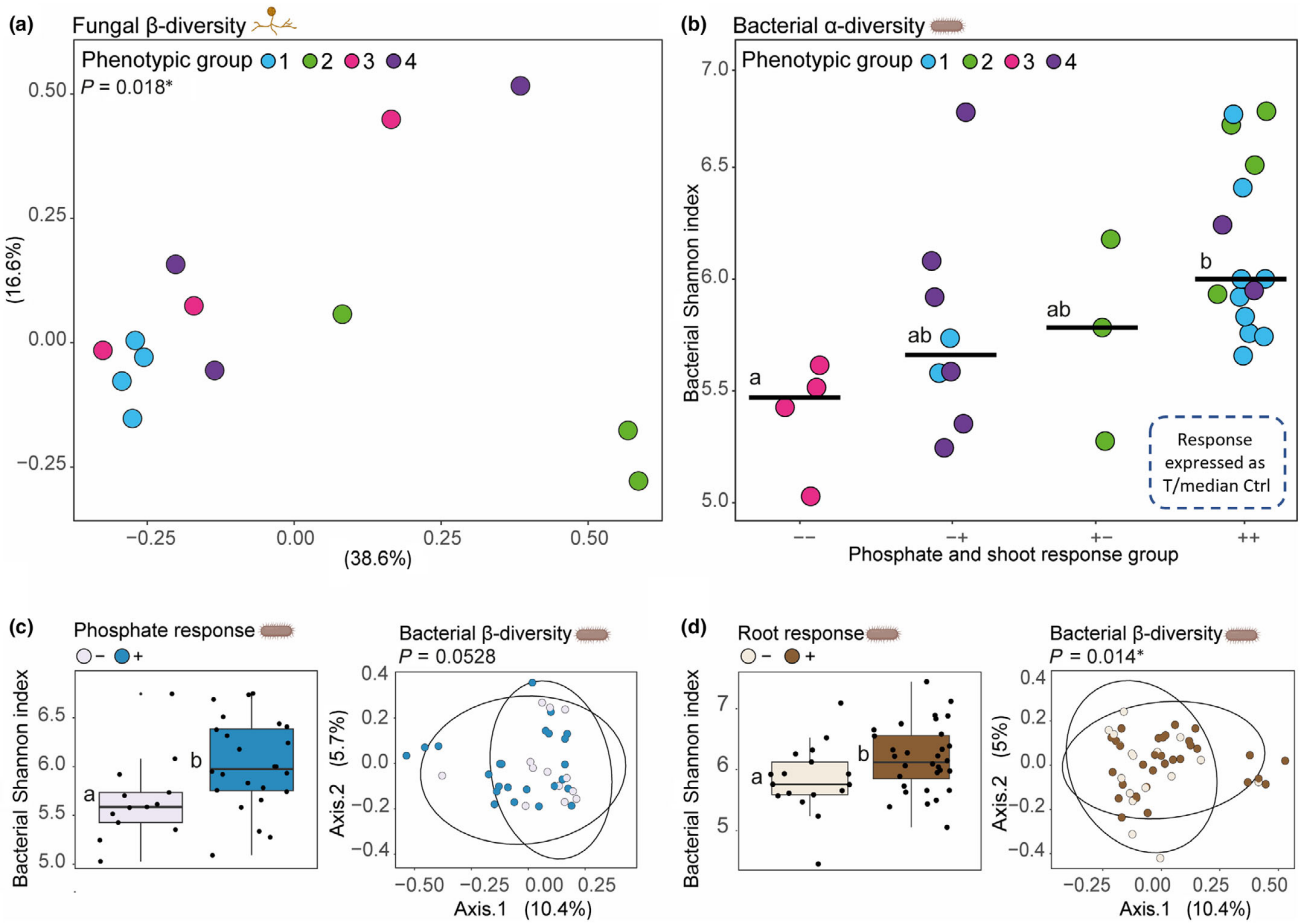
Root‐associated microbial variation at the base of divergent lettuce phosphate and shoot responses. (a) PCoA plot based on fungal Bray–Curtis dissimilarity distance matrix between phenotypic groups (highlighted by colors). Group separation was assessed by PERMANOVA (*P* = 0.018; *, *P* < 0.05). (b) Bacterial Shannon diversity index variation across the four response intervals: ‘−−’ denotes negative responders, ‘++’ positive responders, with ‘−+’ and ‘+−’ representing intermediate responses for phosphate and shoot biomass variation, respectively. Colored dots represent individual biological replicates, colored according to phenotypic group as shown in the color legend in the figure; the black horizontal line indicates the median for each group. Letters above each group indicate statistically homogeneous groups based on Dunn's *post hoc* test with false discovery rate (FDR)‐adjusted *P*‐values. (c) Bacterial Shannon index and Bray–Curtis distance matrix variation comparing plants with low (−, in gray) and high (+, in blue) leaf phosphate accumulation. In the boxplots, the horizontal line represents the median, box edges indicate the 25^th^ and 75^th^ percentiles, whiskers extend to 1.5× the interquartile range (IQR), and individual points represent single biological samples. Letters above boxplots indicate statistically homogeneous groups based on Dunn's *post hoc* test with FDR‐adjusted *P*‐values. (d) Shannon index and Bray–Curtis distance matrix variation comparing plants with negative (−, in light brown) and positive (+, in brown) root biomass response. Letters above boxplots indicate statistically homogeneous groups based on Dunn's *post hoc* test with FDR‐adjusted *P*‐values (*P*.adj = 0.036).

Building on this result, we narrowed the analysis to phosphate and shoot response and observed that bacterial α‐diversity indices increased with progressively higher leaf phosphate and shoot biomass response (Fig. 5b). This trend was most pronounced between plants exhibiting completely negative (−−) vs completely positive (++) responses (Dunn *post hoc*, adjusted *P*‐value = 0.014). By contrast, no comparable differences were observed for fungi (Fig. S9a,b). To determine the main driver of this correlation, we further separated the response into phosphate and shoot components. Fungal diversity did not consistently vary with leaf phosphate (Fig. S9c), shoot biomass (Fig. S9d), root biomass (Fig. S9e, right part), and shoot/root biomass responses (Fig. S9f). The only notable exception was a larger variation in root biomass, which correlated with a significantly higher fungal Shannon index (Dunn *post hoc*, adjusted *P*‐value = 0.0098, Fig. S9e).

In bacteria, leaf phosphate variation was significantly associated with a distinctively richer bacterial community (Dunn *post hoc*, adjusted *P*‐value = 0.012; Figs 5c, S10a,b) and showed a near‐significant effect on bacterial β‐diversity (PERMANOVA, *P*‐value = 0.0528; Fig. 5c). By contrast, shoot biomass variation was not associated with changes in α‐ or β‐diversity (Fig. S10c). Among the other morphological parameters, only root variation was linked to changes in both bacterial α‐ and β‐diversity (Fig. 5d). Specifically, plants with larger root systems hosted significantly richer bacterial communities for both Shannon (Dunn *post hoc*, adjusted *P*‐value = 0.036; Fig. 5d) and observed indices (Dunn *post hoc*, adjusted *P*‐value = 0.025, Fig. S10d) compared to those with smaller roots. The bacterial diversity between the two conditions was also validated by β‐diversity (PERMANOVA, *P*‐value = 0.014; Fig. 5d). Conversely, the shoot/root biomass ratio was not linked to shifts in bacterial β‐ and α‐diversity (Fig. S10e). The positive association between bacterial α‐diversity and leaf phosphate variation was further confirmed in a subsequent experiment, in which plants were treated with the same synthetic community, in controlled growth chamber condition, and with the AM fungus alone (Fig. S11; Table S22; Methods S1).

Together, these findings underscore the tight regulation of root‐associated fungal and bacterial diversity in relation to key plant nutritional parameters, such as leaf phosphate accumulation, while also highlighting its associations with morphological changes, particularly in root system development.

### Positively responding plants show enrichment of differentially abundant ASVs from Burkholderiales and Flavobacteriales

To identify the ASVs that are differentially abundant between plants with positive and negative responses in terms of phosphate accumulation and root biomass, at the base of the observed differences in diversity, we performed a log_2_ fold‐change analysis. This approach revealed 22 bacterial ASVs that were significantly more abundant (adjusted *P*‐value < 0.05) in plants accumulating higher phosphate levels in response to the inoculum, with no ASVs significantly enriched in the opposite condition (Fig. 6a). In addition, six bacterial ASVs showing a base mean above 10, and 16 ASVs with a base mean below 10, were enriched in plants exhibiting a positive root biomass response to the treatment (Figs 6b, S12a).

**Fig. 6:**
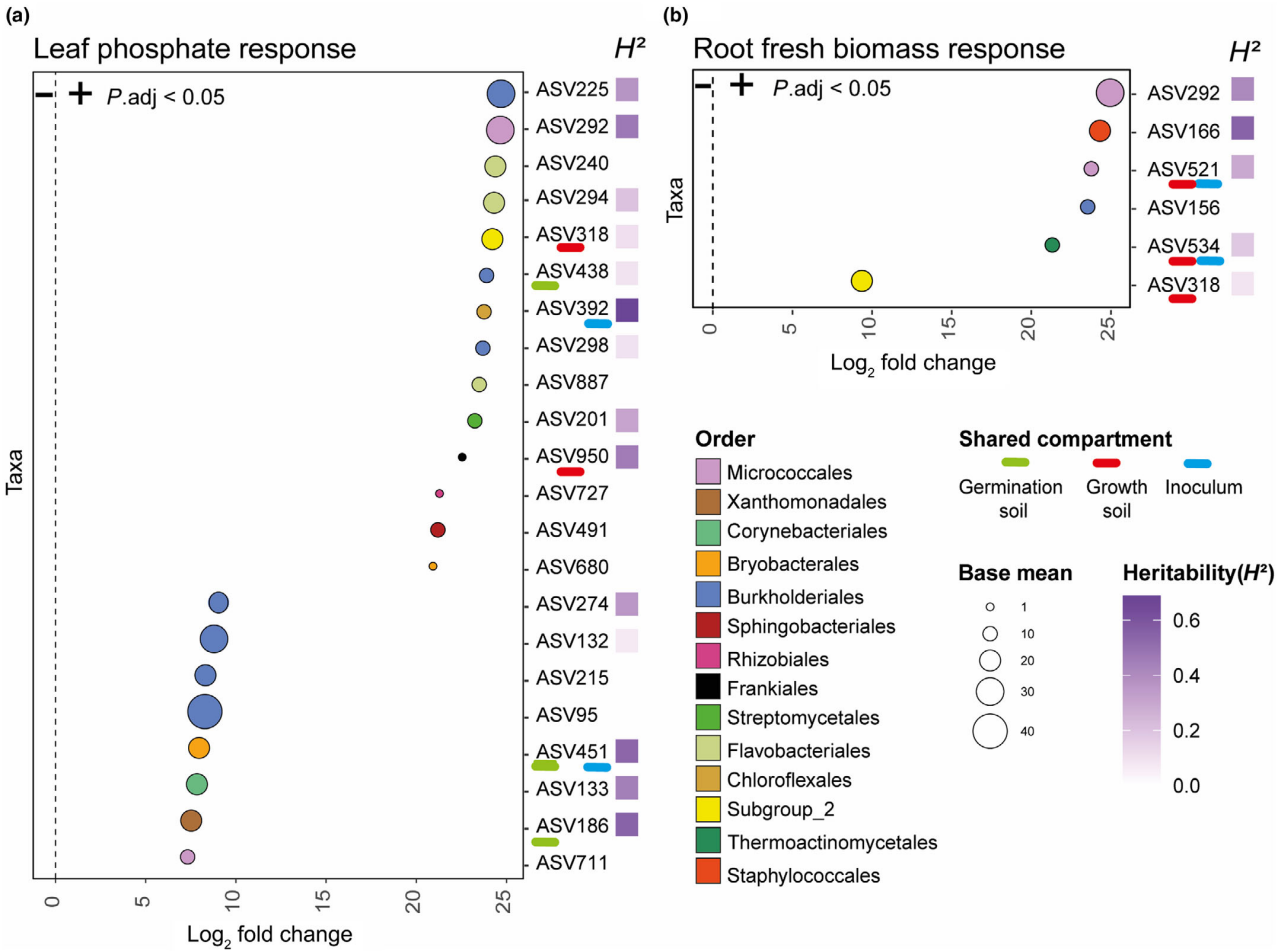
Differential abundance of amplicon sequence variants (ASVs) in lettuce plants with positive and negative leaf phosphate accumulation and root fresh biomass response. (a) Log_2_ fold‐change values representing ASVs that are significantly (*P*.adj < 0.05) more abundant in plants with high (+) and low (−) phosphate accumulation in response to the inoculum. (b) Log_2_ fold‐change values representing ASVs with a base mean above 10 that are significantly (*P*.adj < 0.05) more abundant in plants with a positive (+) and negative (−) root fresh biomass variation in response to the inoculum. Each dot represents an ASV, with colors indicating its taxonomic order and size representing the base mean effect. ASVs shared with the germination soil are underlined in green, in the growth soil in red and in the inoculum in blue. Broad‐sense heritability (*H* ^2^) is shown using a purple color scale, with higher *H* ^2^ values in dark purple and lower values in lighter shades.

To determine if these ASVs were shared with other compartments and speculate a possible origin, we found that, for phosphate response, ASVs 438 and 186 were uniquely associated with the germination soil, ASVs 950 and 318 were specific to the growth soil, and ASV 392 was uniquely shared with the inoculum. In the roots, we identified two ASVs (2775 and 1037) that were uniquely shared with the germination soil and the inoculum, respectively. Additionally, two other ASVs were shared between both the growth soil and the inoculum.

Almost half of the ASVs enriched in the positively responding plants belong to Burkholderiales (7 out of 22) and Flavobacteriales order (3 out of 22; Fig. 6a). In the root biomass response, the Burkholderiales represent 3 out of the 22 total ASVs, while only one Flavobacteriales ASV is present (Figs 6b, S12a).

To evaluate how plant genotype contributes to variation in the presence of differentially abundant ASVs under contrasting conditions, we calculated broad‐sense heritability (Table S23). Eight ASVs, including two from the order Burkholderiales (ASVs 225 and 274), exhibited *H* ^2^ values > 0.30 in response to phosphate variation. Among these, ASVs 392 and 451 showed the highest heritability (*H* ^2^ = 0.69 and 0.54, respectively), and were also detected in the microbial inoculum (Fig. 6a). For root biomass variation, five ASVs exhibited *H* ^2^ values above 0.30, with ASV 521 notably shared with the microbial inoculum (Figs 6b, S12a).

To explore any possible link between the fungal community (with a particular focus on the AMF used in this experiment) and the bacterial ASVs enriched under conditions of increased leaf phosphate and root biomass, we performed correlation analyses between the two communities with a particular focus on Burkholderiales that represented the most enriched order. We found a positive correlation (Pearson's correlation, *R* = 0.39, *P*‐value = 0.0051) between the root‐associated Burkholderiales and Glomeromycota relative abundances (Fig. S12b). After analyzing each differentially enriched ASV in the two conditions described above, ASV292 (Micrococcales order) showed a positive correlation with the relative abundance of the Glomeromycota phylum in both conditions of positive phosphate and root biomass response (Fig. S12c,d). Interestingly, ASV292 had among the highest heritability values in both conditions (*H* ^2^ = 0.46). Other significant correlations were detected between ASV294 (Flavobacteriales order), ASV186 (Xanthomonadales order), and ASV438 (Burkholderiales order) and the Glomeromycota relative abundance in positively responding plants for leaf phosphate (Fig. S12c). Additionally, another member of Micrococcales order, ASV521, was correlated with the fungal community in plants with a positive root biomass response (Fig. S12d).

In summary, we identified 44 bacterial ASVs associated with increased phosphate accumulation and improved root biomass response. Among these, five ASVs were positively correlated with the Glomeromycota fungal phylum, suggesting a possible effect of fungal presence on the bacterial community and, consequently, on plant nutrient acquisition.

### Effects of six selected ASV‐corresponding isolates on leaf phosphate content and shoot biomass under plate and pot conditions

To test causal relationships between the identified ASVs, plant biomass, and leaf phosphate concentration, we selected six isolates from the lettuce‐root culture collection whose sequences were highly similar (> 97% identity) to ASVs that were significantly enriched in our dataset. These isolates were used in both plate assays and pot experiments, as described in the Materials and Methods section (the Bacterial tests on lettuce plant growth section), to assess phosphate‐related phenotypes with and without bacterial inoculation in three lettuce genotypes that showed differential responses in the initial screening (Fig. 1; Table S10).

In the plate experiment, the isolate corresponding to ASV438 (Comamonadaceae_1) significantly increased leaf phosphate content in genotype TKI‐38. Similarly, the isolate corresponding to ASV225 (Comamonadaceae_2) increased leaf phosphate content in genotype TKI‐100 compared with both the mock treatment and the synthetic community composed of all six isolates (Fig. 7a). None of the tested isolates, either individually or in combination, significantly affected shoot biomass, measured as fresh weight, under plate conditions, except for the Xanthomonadaceae isolate, corresponding to ASV186, which reduced shoot biomass in TKI‐100 compared with all other treatments (Fig. 7a). Under pot conditions, the synthetic community composed of all six isolates significantly increased fresh plant biomass in two of the three genotypes tested, namely genotypes TKI‐24 and TKI‐38 (Fig. 7b).

**Fig. 7:**
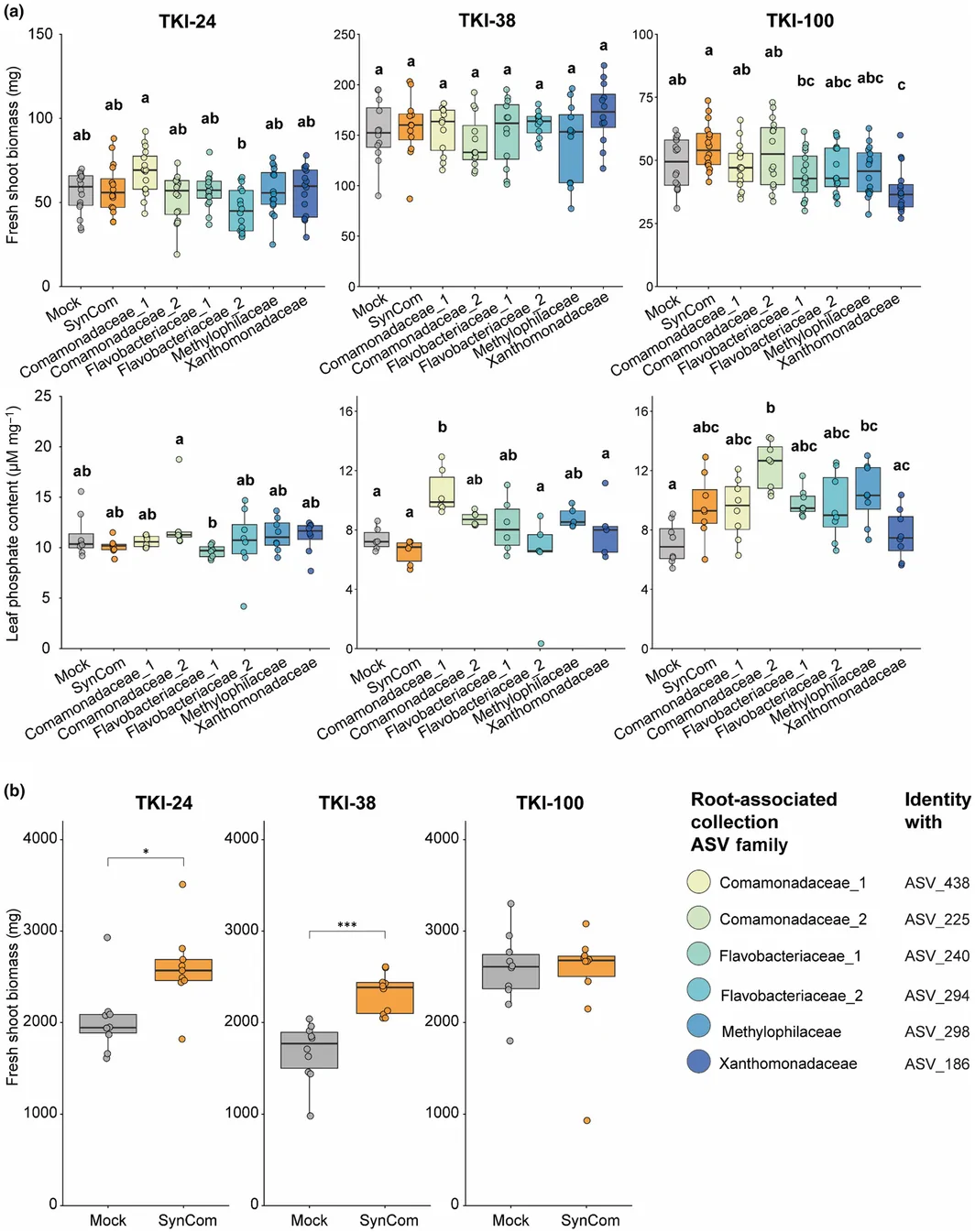
Effects of beneficial amplicon sequence variants (ASVs) and their combination on lettuce shoot biomass and leaf phosphate concentration under plate and pot conditions. (a) Effects of individual bacterial ASVs (blue‐shaded boxplots, grouped at the family level) and their combined synthetic community (SynCom, orange) compared with mock‐treated plants (gray) on shoot fresh biomass (mg, upper panels) and leaf phosphate content (μM mg^−1^, lower panels) in genotypes 24, 38, and 100 under plate conditions. Mock‐treated plants are shown in gray, SynCom in orange, and individual ASVs in blue‐toned colors grouped by bacterial family. In the boxplots, the horizontal line represents the median, box edges indicate the 25^th^ and 75^th^ percentiles, and whiskers extend to 1.5× the interquartile range (IQR); each dot represents a biological replicate. Different letters indicate statistically homogeneous groups based on Dunn's *post hoc* test with Benjamini–Hochberg (BH)‐adjusted *P*‐values. (b) Effect of the SynCom on shoot fresh biomass (mg) in genotypes 24, 38, and 100 under pot conditions. Asterisks indicate statistically significant differences between mock and SynCom treatments, as assessed by Wilcoxon rank‐sum test (*, *P* < 0.005; ***, *P* < 0.001). ASV family and identity are shown in the legend.

Together, these experiments provide partial support for a link between ASVs enriched in plants showing positive responses to the treatment and plant phenotypes following inoculation with corresponding bacterial isolates. At the single‐isolate level, *in vitro*, two of the six tested strains increased leaf phosphate content, with each effect observed in one genotype, indicating genotype‐dependent but not broadly consistent effects. By contrast, when applied together as a synthetic community under pot conditions, the isolates significantly increased plant biomass in two of the three tested genotypes. Thus, the validation experiments suggest that selected members of the enriched bacterial community can influence phosphate accumulation and, in combination, promote biomass under specific plant genotype and growth‐condition contexts.

## Discussion

It is increasingly evident that plant intraspecific variation is crucial in shaping the plant microbiota and plant responses to it. However, the specific genetic regions responsible remain largely unknown and it is not clear if the same genomic variants only govern interactions with specific microorganisms or a broad spectrum of them (Peiffer *et al*., 2013; Bulgarelli *et al*., 2013; Hassani *et al*., 2018; Deng *et al*., 2021).

In this study, we explored the connection between genetic diversity and plant responses to a soil microbial inoculum by conducting a large‐scale glasshouse experiment on a population of 128 *Lactuca sativa* genotypes. Our findings revealed a strong influence of genetic diversity on both the phenotypic and physiological responses of plants. Specifically, we observed that different genotypes exhibit varied responses to the treatment both in morphological traits, such as root and shoot biomass, leaf angle, and in physiological traits, such as phosphate accumulation.

Supporting these findings, a GWAS analysis identified specific genetic regions in the lettuce genome associated with responses to microbial inoculation. For key agronomic traits such as leaf phosphate concentration and shoot biomass, we identified one SNP on chromosome 4 and two SNPs on chromosomes 2 and 5, respectively, that are linked to variations resulting from the treatment. Overall, heritability values decreased when evaluating the plant's response to microbial inoculation compared to the median values observed in treated and untreated plants. However, for the two traits mentioned above, leaf phosphate concentration and shoot biomass, heritability increased in response to the inoculation. This underscores the genetic control exerted by plants on key parameters that are influenced by interactions with beneficial microorganisms. Identifying the putative genes involved in establishing interactions that determine these beneficial fitness phenotypes in the host will be crucial and will require further investigation using transcriptomic and metabolomics approaches, taking into account the large Linkage Disequilibrium in *Lactuca sativa*, where the pairwise correlation coefficient dropped to half of its maximum value at 177.7 kb (Wei *et al*., 2021).

Beyond investigating the plant genetic base of differential plant responses to soil microorganisms, our results revealed a strong connection between the four identified plant phenotypic response groups and the root‐associated fungal β‐diversity. Conversely, root‐associated bacterial communities were associated with the plant response through their α‐diversity, showing increased Shannon and Observed indexes with progressively more positive shoot biomass and leaf phosphate accumulation. These findings suggest a potential link between plant phenotypic responses to microbial inoculum, phosphate accumulation, and the richness and diversity of root‐associated microbial communities.

Furthermore, we identified 22 bacterial ASVs differentially enriched in plants with a positive leaf phosphate response to the inoculum and 22 ASVs enriched in plants with increased root biomass as a result of the treatment. Two of them, ASV392 (belonging to the Chloroflexales order) and ASV1037 (belonging to the Corynebacteriales order) for leaf phosphate and root biomass, respectively, were uniquely shared with the inoculum. Given that 7 out of 22 ASVs associated with improved leaf phosphate assimilation belong to the Burkholderiales order, known to be obligate endosymbionts of the cytoplasm or intracellular structures of AMF (Basiru *et al*., 2023; Zhang *et al*., 2024), we investigated their potential association with the AMF present in our inoculum, which belongs to the phylum Glomeromycota. Our findings revealed a positive correlation between root‐associated Burkholderiales order and Glomeromycota phylum. Among Burkholderiales, ASV438 was positively correlated with Glomeromycota relative abundance in positively responding plants for phosphate accumulation. To establish causality between differentially enriched ASVs and their effects on plant phenotypes, as hypothesized on the basis of the bioinformatic analyses, we performed experimental validation using six bacterial strains isolated from lettuce roots. These strains were tested individually and in combination (SynCom) under both *in vitro* plate conditions and in sterile sand in growth chamber experiments, across three lettuce genotypes. Overall, these experiments demonstrated that the selected bacterial strains can significantly influence plant growth and leaf phosphate content; however, their effects are strongly genotype‐dependent.

Overall, these findings highlight three key considerations for improving the efficacy of microbial inoculants when applied to soil. First, plant genotype is crucial and should be considered in the breeding process to select plants capable of establishing beneficial interactions with soil microorganisms, which can enhance agronomic parameters such as leaf phosphate content and shoot biomass (Escudero‐Martinez & Bulgarelli, 2023). Second, selecting plant morphological traits, such as root biomass and leaf phosphate concentration, that promote the association with richer soil microbial communities can further enhance the functional benefits these microorganisms provide to plants. Third, integrating genetic and phenotypic variation can facilitate the identification of bacterial strains linked to variation in agriculturally important phenotypic traits under different conditions.

Further research should target fungal communities using tailored experimental designs and increased sequencing depth to better understand their interactions with bacterial assemblages. In addition, given the challenges in tracking inoculated bacteria throughout the experiment (only *Chryseobacterium* was recovered), future efforts should prioritize methods to improve the detection and persistence assessment of introduced strains.

The results of this research not only clarify some of the mechanisms shaping interactions between soil microorganisms and *Lactuca sativa* roots but also provide a foundation for future breeding efforts. These should focus on selecting optimal combinations of genotypes, morphological traits, and microbial communities to develop resilient plants that sustainably meet the needs of a growing global population without compromising environmental health.

## Competing interests

None declared.

## Author contributions

A Capparotto, MG and FV planned and designed the research. A Capparotto, A Ciampanelli, MG, CS, SS, FV and AA performed experiments and conducted fieldwork. A Capparotto and PS conducted the phosphate and metabolite quantification. A Capparotto performed DNA extraction. A Capparotto, MG and PC analyzed data. CS, SS, AA, FV, SH and MG supervised the work. A Ciampanelli, JH and GC created the culture collection. A Capparotto, A Ciampanelli and MG wrote the manuscript. A Capparotto and A Ciampanelli contributed equally to this work.

## Disclaimer

The New Phytologist Foundation remains neutral with regard to jurisdictional claims in maps and in any institutional affiliations.

## Supporting information


**Fig. S1** Frequency of mycorrhization (F%) in control and treated samples.
**Fig. S2** Digital biomass trends of genotypes with differential responses (9‐11‐74‐38‐57‐100) over the course of the experiment.
**Fig. S3** Correlation matrix among all parameters measured by the Phenospex system.
**Fig. S4** GWAS of phenospex parameters.
**Fig. S5** Metabolite quantification to validate genotype phenotypic categorization.
**Fig. S6** Fungal community richness and composition across different compartments.
**Fig. S7** Bacterial community richness and composition across different compartments.
**Fig. S8** Root‐associated microbial variation at the base of phenotypic plant response.
**Fig. S9** Root‐associated fungal community variation at the base of divergent responses.
**Fig. S10** Root‐associated bacterial community variation at the base of divergent responses.
**Fig. S11** Leaf Pi response to fungal (Myc) and synthetic microbial community (SynCom) treatments under Pi deficiency.
**Fig. S12** Correlation analysis between the root‐associated fungal community and the bacterial ASVs enriched in the plants with a positive leaf phosphate and root biomass response.

**Methods S1** Supplementary materials and methods.

**Notes S1** Supplementary script.

**Table S1** Preliminary lettuce phosphate starvation trial conducted before the large‐scale experiment.
**Table S2** Glasshouse experimental design.
**Table S3** Fertirrigation and inoculum administration plan.
**Table S4** PlantEye scanning order.
**Table S5** PlantEye measurements per genotype.
**Table S6** Fresh shoot and root biomass per genotype.
**Table S7** Sampling design.
**Table S8** Soluble leaf phosphate (Pi) quantification.
**Table S9** Leaf metabolite quantification.
**Table S10** Genotypes selected for DNA extraction.
**Table S11** Sample information and quantification values for the genotypes selected for 16S metagenomic analysis.
**Table S12** Reads remaining at each step of sample contamination removal.
**Table S13** Percentage identity between the root‐associated collection and the ASVs identified in this study.
**Table S14** Long Ashton recipe.
**Table S15** Trouvelot assay data.
**Table S16** Dunn *post hoc* test for leaf phosphate concentration between control and treated plants.
**Table S17** Dunn *post hoc* test for shoot biomass between control and treated plants.
**Table S18** Broad sense heritability values for control (c), treated (T), and their ratio (T/Ctrl).
**Table S19** Genome‐wide association study outcomes.
**Table S20** Bacterial and fungal phylum relative abundance across the different compartments.
**Table S21** Comparison between the reference sequences used to prepare the inoculum and the sequences detected in the final dataset.
**Table S22** Experimental design and leaf phosphate quantification from the additional experiment conducted to validate alpha diversity results.
**Table S23** Broad‐sense heritability values for ASVs that are differentially abundant in plants responding positively vs negatively to leaf phosphorus (Pi) and plants with positive vs negative responses in root biomass.
Please note: Wiley is not responsible for the content or functionality of any Supporting Information supplied by the authors. Any queries (other than missing material) should be directed to the *New Phytologist* Central Office.

## Data Availability

The datasets generated during the current study are available in the NCBI SRA repository as Bioproject PRJNA1215347. The R script used to obtain the results and the figures presented in this paper is reported as Notes S1.
