# Supplementary material for: Plant genetic and root‐associated microbial diversity modulate Lactuca sativa responsiveness to a soil inoculum under phosphate deficiency
Source: New Phytol. 2026 Sep 2;252(4):1584–601. doi: 10.1111/nph.71529 (PMC13669811; doi:10.1111/nph.71529)
Supplement: Supplementary file 1 [file NPH-252-1584-s001.pdf]

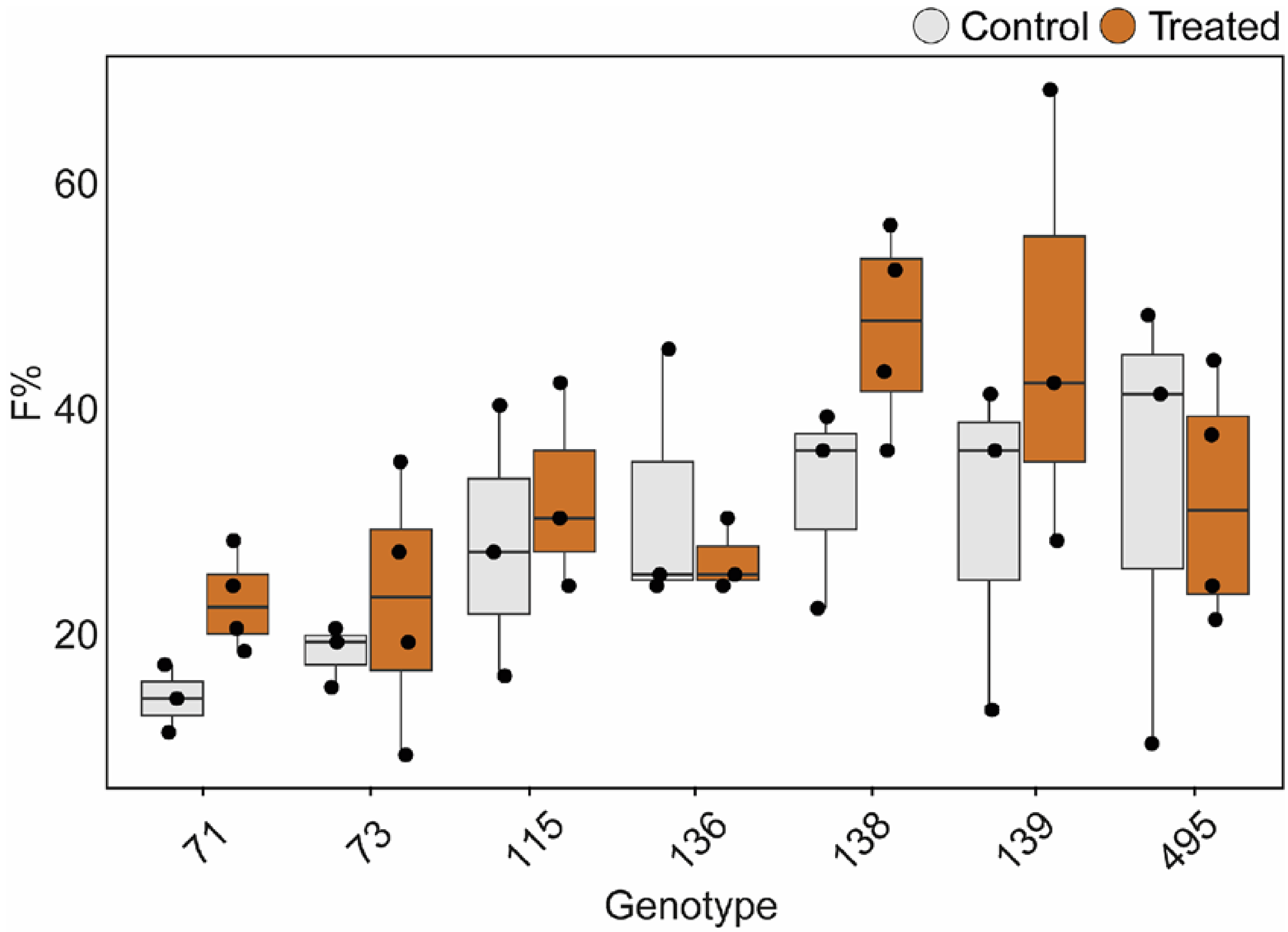

Fig. S1. Frequency of mycorrhization (F%) in control and treated samples.
Boxplots show the distribution of F% values for each genotype, comparing Control (gray) and Treated (brown) plants, as indicated in the color legend above the plot. In the boxplots, the horizontal line represents the median, box edges indicate the 25th and 75th percentiles, whiskers extend to 1.5× the interquartile range (IQR), and individual points represent single biological samples.

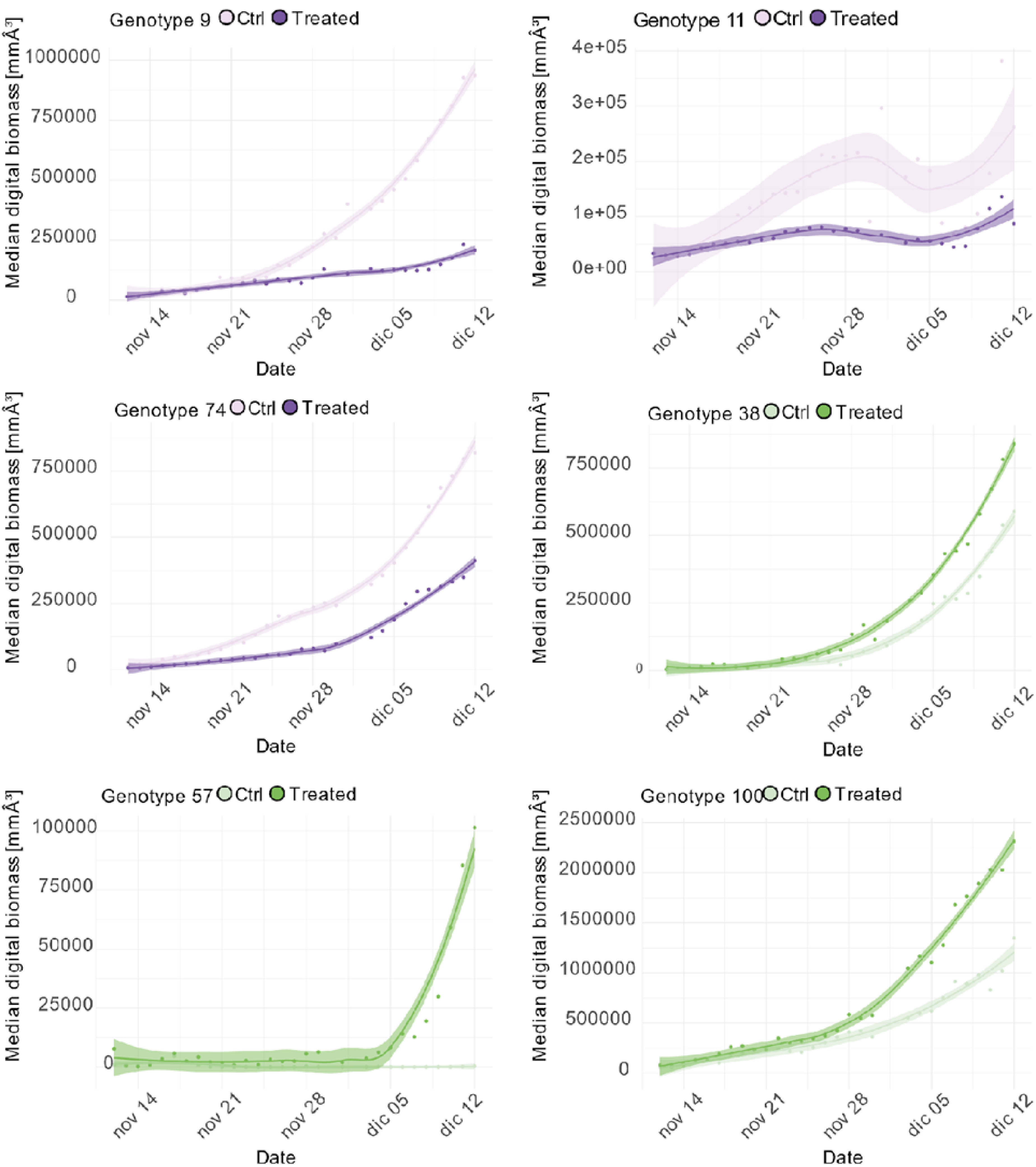

Fig. S2. Digital biomass trends of genotypes with differential responses (9-11-74-38-57-100) over the course of the experiment. Temporal trends in digital biomass are shown for six genotypes with differential responses to the microbial inoculum, grouped according to their overall response direction. Each panel shows median digital biomass (mm³) over time for one genotype, measured at five time points spaced one week apart. Genotypes that show a decrease in biomass in response to the treatment are highlighted in purple, while those that experience an increase in biomass due to inoculation are shown in green. For each genotype, two series are shown: Treated (dark purple/dark green) and Control (light purple/light green), following the same color scheme. Solid lines represent the median trend for each treatment group, and shaded areas represent the 95% confidence interval.

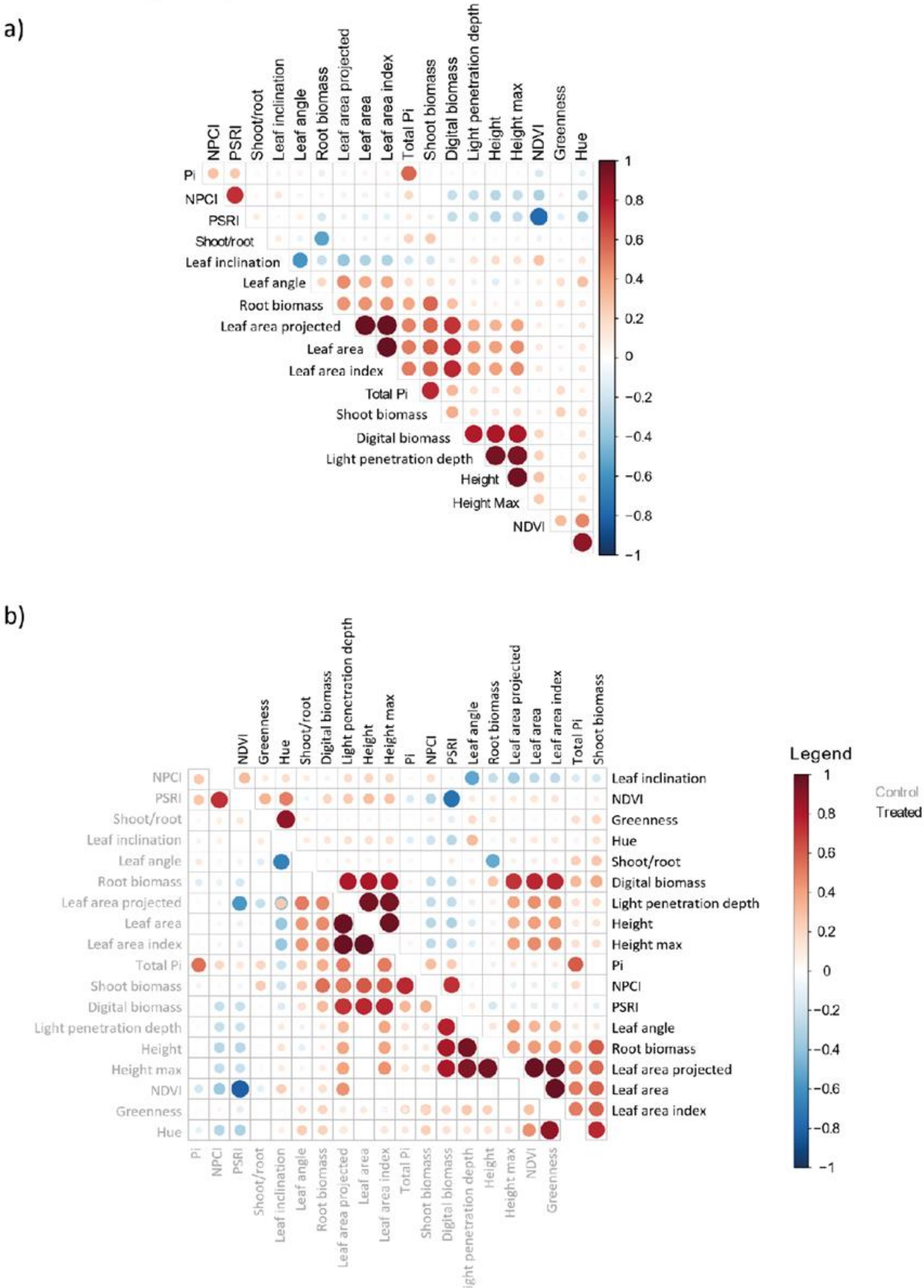
a)
NPCI
PSRI
Shoot/root
Leaf inclination
Leaf angle
Root biomass
Leaf area projected
Leaf area
Leaf area index
Total Pi
Shoot biomass
Digital biomass
Light penetration depth
Height
Height max
NDVI
Greenness
Hue
Pi
Height Max
1
0.8
0.6
0.4
0.2
0
−0.2
−0.4
−0.6
−0.8
−1
b)
Legend
Control
Treated
NDVI
Greenness
Hue
Shoot/root
Digital biomass
Light penetration depth
Height
Height max
Pi
NPCI
PSRI
Leaf angle
Root biomass
Leaf area projected
Leaf area
Leaf area index
Total Pi
Shoot biomass
Leaf inclination

Fig. S3. Correlation matrix among all parameters measured by the Phenospex system.
Pairwise correlations among all phenotypic and physiological parameters measured by the Plant Eye (Phenospex) system are shown as **(a)** a correlation matrix including all samples, and **(b)** a correlation matrix highlighting differences between treated and control samples.
In both panels, color indicates the direction and strength of the correlation along a continuous gradient, with positive correlations shown in red, negative correlations in blue, and no correlation in white. Dot size is proportional to the strength of the correlation, with larger dots representing stronger correlations (positive or negative). The parameters included are: Pi, NPCI (Normalized Pigment Chlorophyll ratio Index), PSRI (Plant Senescence Reflectance Index), Shoot/root, leaf inclination, leaf angle, root biomass, projected leaf area, leaf area, leaf area index, total Pi, shoot biomass, digital biomass, light penetration depth, height, maximum height, and NDVI (Normalized Difference Vegetation Index).
(b) Variable labels shown in black refer to correlations calculated in treated plants, while labels shown in gray refer to correlations calculated in control plants.

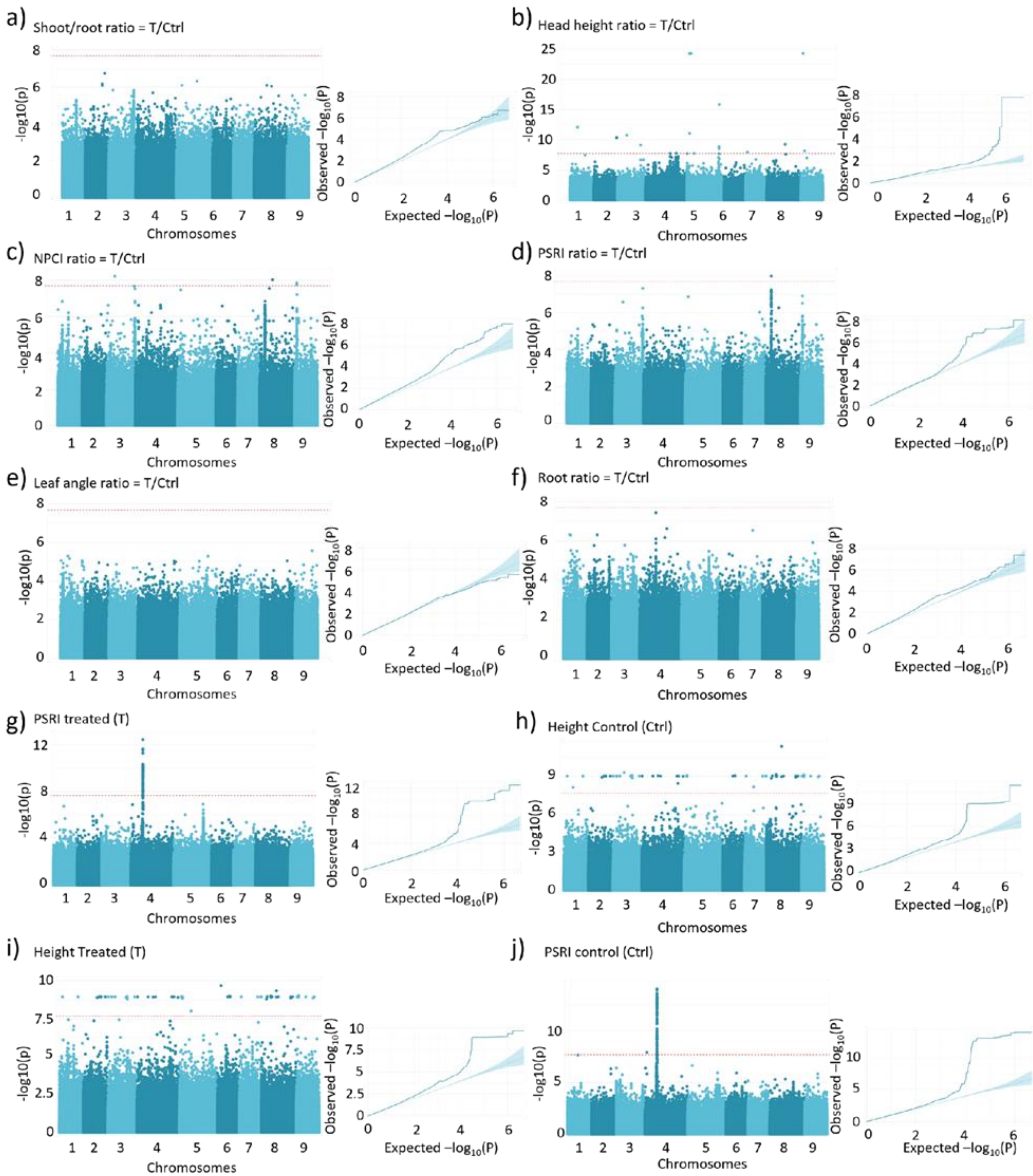

Fig. S4. GWAS of phenospex parameters. **(a-j)** Manhattan plots (left) show the association between SNPs (single nucleotide polymorphisms) in the *Lactuca sativa* genome and the following traits, plotted as −log10(p) values against SNP genomic position: (a) shoot/root ratio, (b) head height ratio, (c) NPCI (Normalized Pigment Chlorophyll ratio Index) ratio, (d) PSRI (Plant Senescence Reflectance Index) ratio, (e) leaf angle ratio, (f) root ratio, (g) PSRI treated, (h) height control, (i) height treated, and (j) PSRI control. The dashed horizontal line indicates the genome-wide significance threshold (−log10(p) = 7.5). QQ (quantile-quantile) plots (right) illustrate expected versus observed −log10 (p) values under the null hypothesis of no association; the solid line represents the expected distribution under the null hypothesis, and the shaded area represents the 95% confidence interval, which widens toward the upper tail of the distribution.

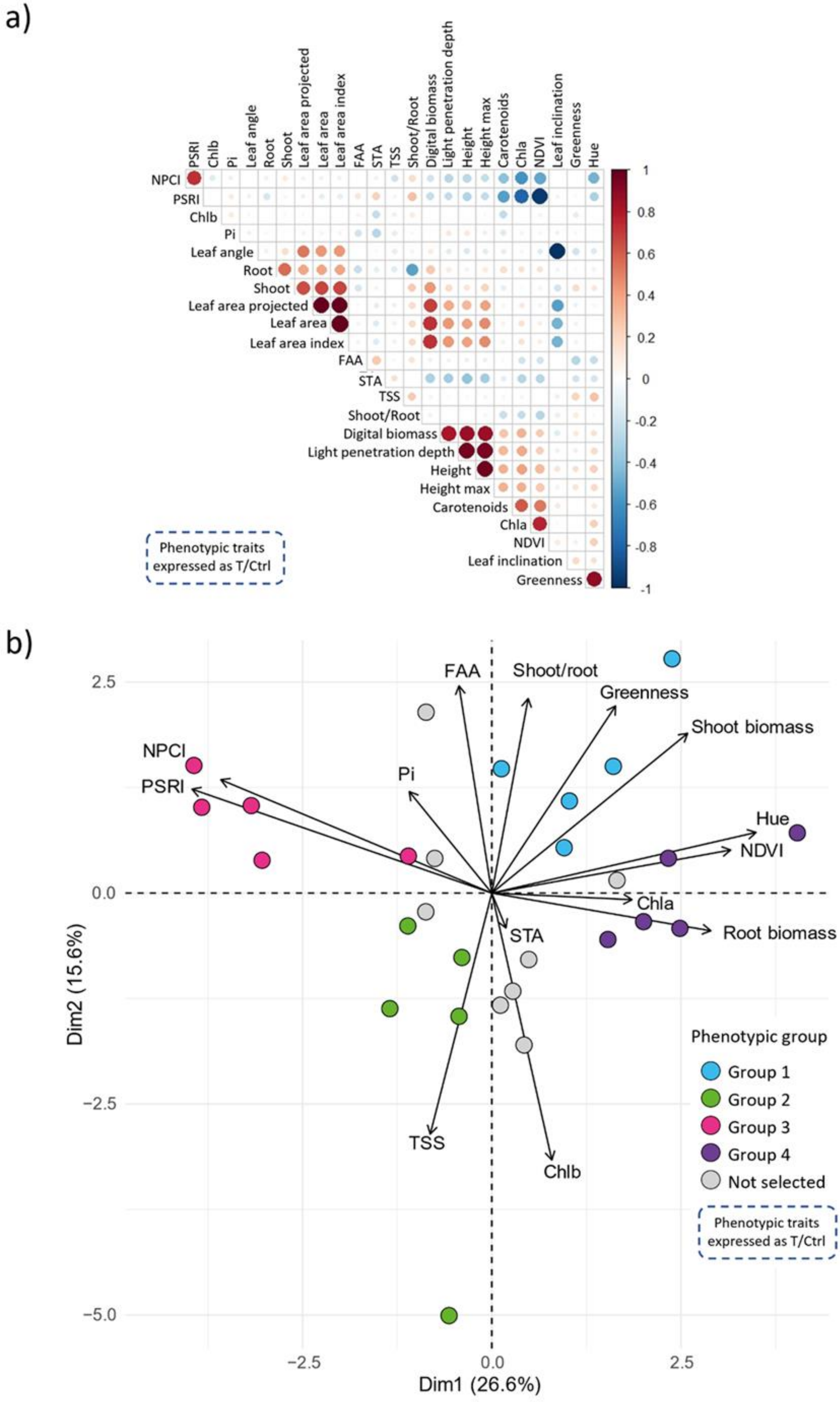
a)
NPCI
PSRI
Chlb
Pi
Leaf angle
Root
Shoot
Leaf area projected
Leaf area
Leaf area index
FAA
STA
TSS
Shoot/Root
Digital biomass
Light penetration depth
Height
Height max
Carotenoids
Chla
NDVI
Leaf inclination
Greenness
Hue
1
0.8
0.6
0.4
0.2
0
-0.2
-0.4
-0.6
-0.8
-1
Phenotypic traits expressed as T/Ctrl
b)
FAA
Shoot/root
Greenness
Shoot biomass
NPCI
PSRI
Pi
Hue
NDVI
Chla
Root biomass
STA
TSS
Chlb
Dim1 (26.6%)
Dim2 (15.6%)
2.5
0.0
-2.5
-5.0
Phenotypic group
Group 1
Group 2
Group 3
Group 4
Not selected
Phenotypic traits expressed as T/Ctrl

Fig. S5. Metabolite quantification to validate genotype phenotypic categorization. **(a)** Correlation matrix among all parameters measured by the Phenospex system and the quantified leaf metabolites. Color indicates the direction and strength of the correlation along a continuous gradient, with positive correlations shown in red, negative correlations in blue, and no correlation in white. Dot size is proportional to the strength of the correlation, with larger dots representing stronger correlations. The parameters included are: NPCI (Normalized Pigment Chlorophyll ratio Index), PSRI (Plant Senescence Reflectance Index), Chlb (chlorophyll b), Pi, leaf angle, root biomass, shoot biomass, projected leaf area, leaf area, leaf area index, FAA (free amino acids), STA (starch), TSS (total soluble sugars), Shoot/root, digital biomass, light penetration depth, height, maximum height, Carotenoids, Chla (chlorophyll a), NDVI (Normalized Difference Vegetation Index), leaf inclination, and Greenness.

(b) Principal component analysis (PCA) of responses to the inoculum in 28 selected genotypes, based on plant phenotypes and leaf metabolites. Genotypes chosen for root-DNA extraction, representing four distinct phenotypic groups, are color-coded in blue, green, pink, and purple. Non-selected genotypes are displayed in gray. Arrows indicate the loading vectors of the traits contributing to the principal components, with direction and length representing the strength and orientation of each trait's contribution to the two dimensions shown; trait labels are as follows: NPCI, PSRI, Pi, FAA, Shoot/root, Greenness, shoot biomass, Hue, NDVI, Chla, STA, root biomass, Chlb, and TSS.

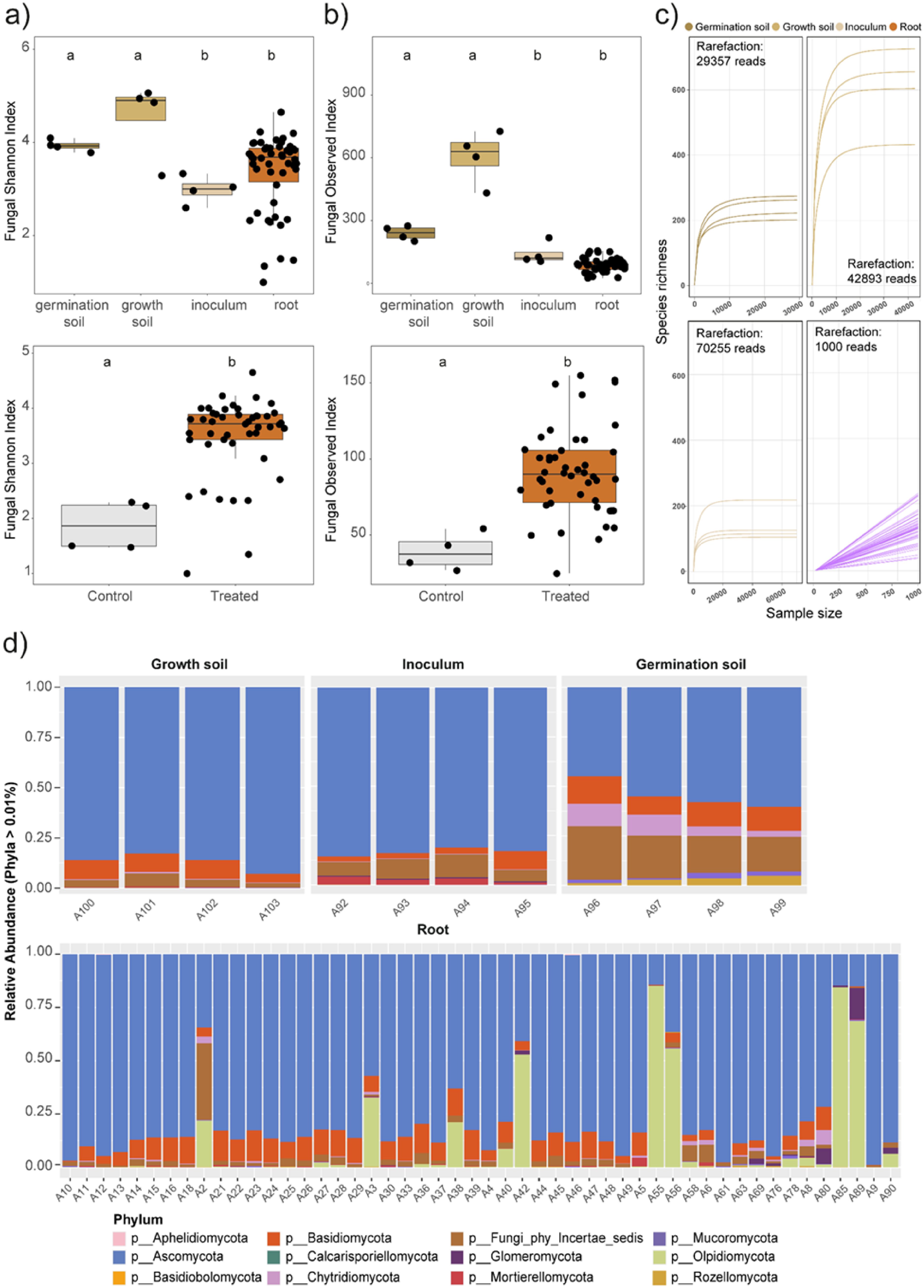
a)
Fungal Shannon Index
germination soil
growth soil
inoculum
root
Control
Treated
b)
Fungal Observed Index
c)
Germination soil
Growth soil
Inoculum
Root
Rarefaction: 29357 reads
Rarefaction: 42893 reads
Rarefaction: 70255 reads
Rarefaction: 1000 reads
Species richness
Sample size
d)
Growth soil
Inoculum
Germination soil
Root
Relative Abundance (Phyla > 0.01%)
Phylum
p__Aphelidiomycota
p__Ascomycota
p__Basidiobolomycota
p__Basidiomycota
p__Calcarisporiellomycota
p__Chytridiomycota
p__Fungi_phy_Incertae_sedis
p__Glomeromycota
p__Mortierellomycota
p__Mucoromycota
p__Olpidiomycota
p__Rozellomycota

Fig. S6. Fungal community richness and composition across different compartments. (a,b) Shannon (a) and Observed (b) α-diversity indices across germination soil, growth soil, inoculum, and root samples, color-coded according to compartment using a brown/sand color scale, consistent with panel (c). The lower section of each panel compares Shannon (a) and Observed (b) α-diversity between control (gray) and treated (brown) root samples. In the boxplots, the horizontal line represents the median, box edges indicate the 25th and 75th percentiles, whiskers extend to 1.5× the interquartile range (IQR), and individual points represent single biological samples. Letters above boxplots indicate statistically homogeneous groups based on Dunn's post-hoc test with FDR-adjusted p-values.
(c) Rarefaction curves following size-based rarefaction across germination soil, growth soil, inoculum, and root samples, shown at four sequencing depths: 29,357 reads (top left), 42,893 reads (top right), 70,255 reads (bottom left), and 1,000 reads (bottom right). All curves are shown in purple; the shallowest depth (1,000 reads) is included to illustrate that rarefaction has not yet reached a plateau at this sequencing depth.
(d) Fungal community composition at the phylum level across compartments, shown as stacked bar plots. Only phyla with relative abundance above 0.01% are shown; phylum colors are indicated in the legend in panel (d).

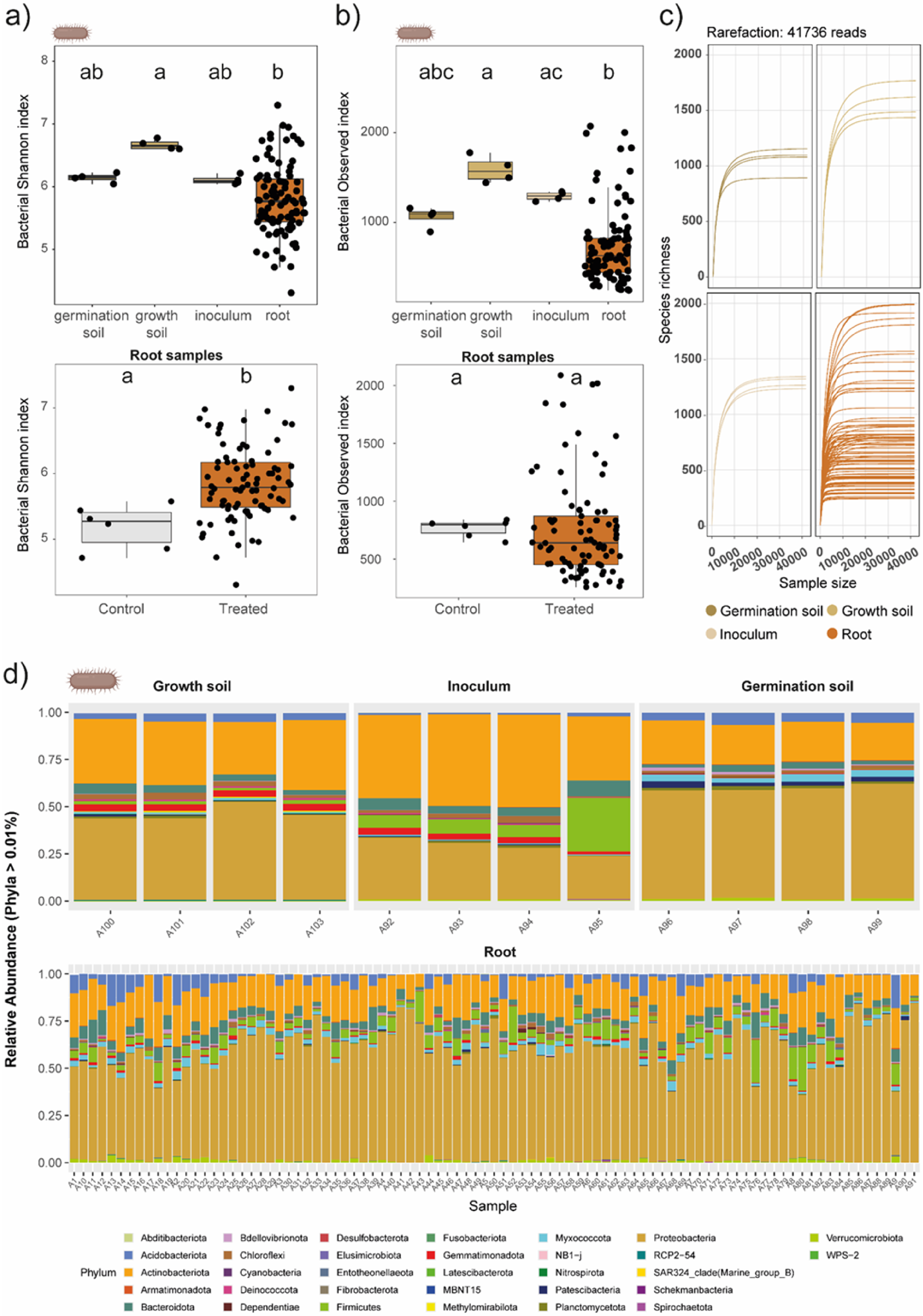
a)
ab
a
ab
b
Bacterial Shannon index
germination soil
growth soil
inoculum
root
Root samples
a
b
Control
Treated
b)
abc
a
ac
b
Bacterial Observed index
germination soil
growth soil
inoculum
root
Root samples
a
a
Control
Treated
c)
Rarefaction: 41736 reads
Species richness
Sample size
Germination soil
Growth soil
Inoculum
Root
d)
Growth soil
Inoculum
Germination soil
A100
A101
A102
A103
A92
A93
A94
A95
A96
A97
A98
A99
Root
Relative Abundance (Phyla > 0.01%)
Sample
Phylum
Abditibacteriota
Acidobacteriota
Actinobacteriota
Armatimonadota
Bacteroidota
Bdellovibrionota
Chloroflexi
Cyanobacteria
Deinococcota
Dependentiae
Desulfobacterota
Elusimicrobiota
Entotheonellaeota
Fibrobacterota
Firmicutes
Fusobacteriota
Gemmatimonadota
Latescibacterota
MBNT15
Methylomirabilota
Myxococcota
NB1−j
Nitrospirota
Patescibacteria
Planctomycetota
Proteobacteria
RCP2−54
SAR324_clade(Marine_group_B)
Schekmanbacteria
Spirochaetota
Verrucomicrobiota
WPS−2

Fig. S7. Bacterial community richness and composition across different compartments.

(a,b) Shannon (a) and Observed (b) α-diversity indices across germination soil, growth soil, inoculum, and root samples, color-coded according to compartment using a brown/sand color scale, consistent with panel (c). The lower section of each panel compares Shannon (a) and Observed (b) α-diversity between control (gray) and treated (brown) root samples. In the boxplots, the horizontal line represents the median, box edges indicate the 25th and 75th percentiles, whiskers extend to 1.5× the interquartile range (IQR), and individual points represent single biological samples. Letters above boxplots indicate statistically homogeneous groups based on Dunn's post-hoc test with FDR-adjusted p-values.

(c) Rarefaction curves following size-based rarefaction across germination soil, growth soil, inoculum, and root samples, shown at a single sequencing depth of 41,736 reads.

(d) Bacterial community composition at the phylum level across compartments, shown as stacked bar plots. Only phyla with relative abundance above 0.01% are shown; phylum colors are indicated in the legend in panel (d).

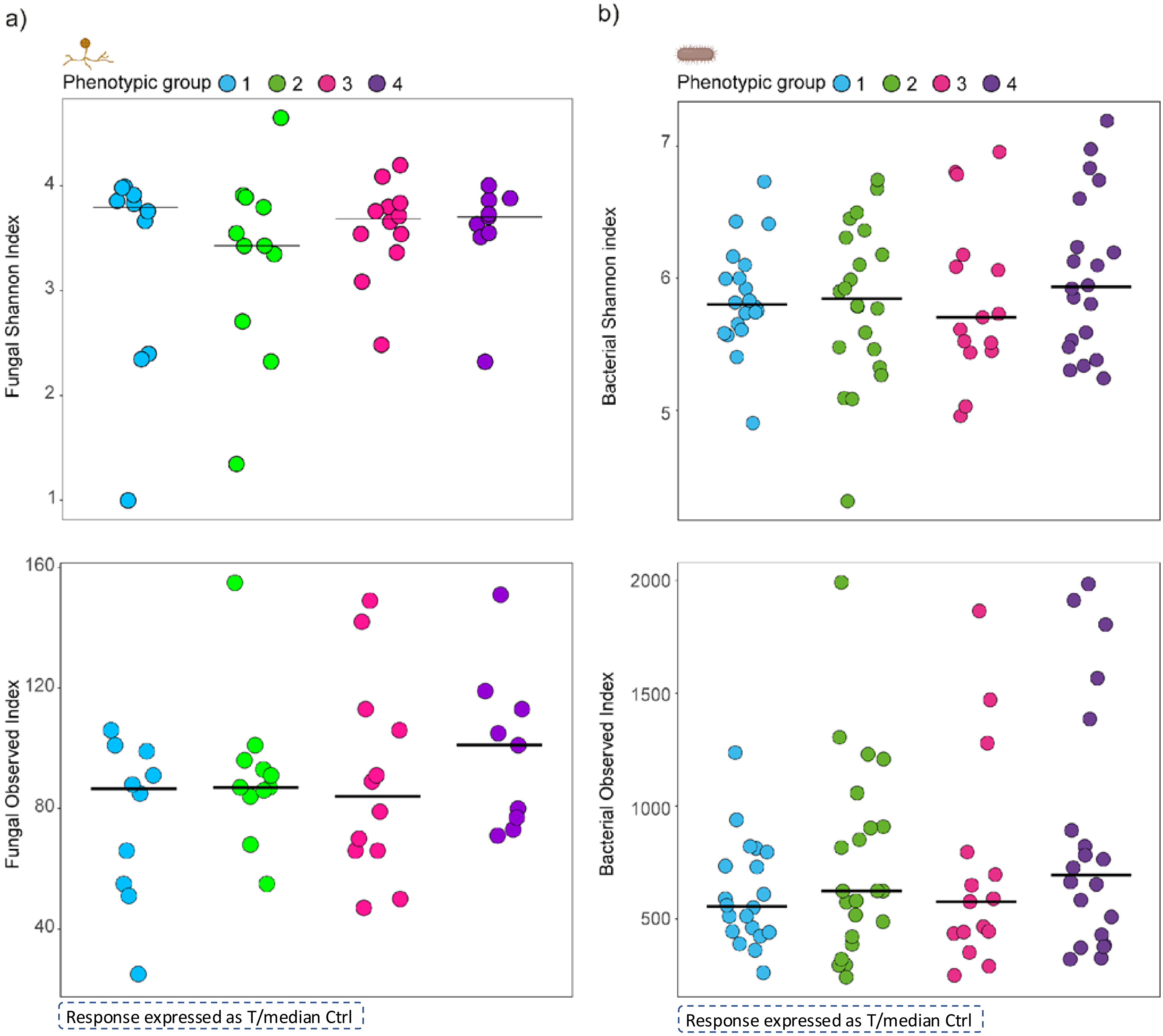

Fig. S8. Root-associated microbial variation at the base of phenotypic plant response. Fungal (a) and bacterial (b) α-diversity indices are shown across the four phenotypic groups of plant response to the microbial inoculum.

(a,b) Shannon (above) and Observed (below) α-diversity indices for fungal (a) and bacterial (b) communities across the four phenotypic groups, indicated by different colors (group 1, light blue; group 2, green; group 3, pink; group 4, purple). Colored dots represent individual biological replicates, and the horizontal line indicates the median for each group. Response is expressed as treated/median control (T/median Ctrl).

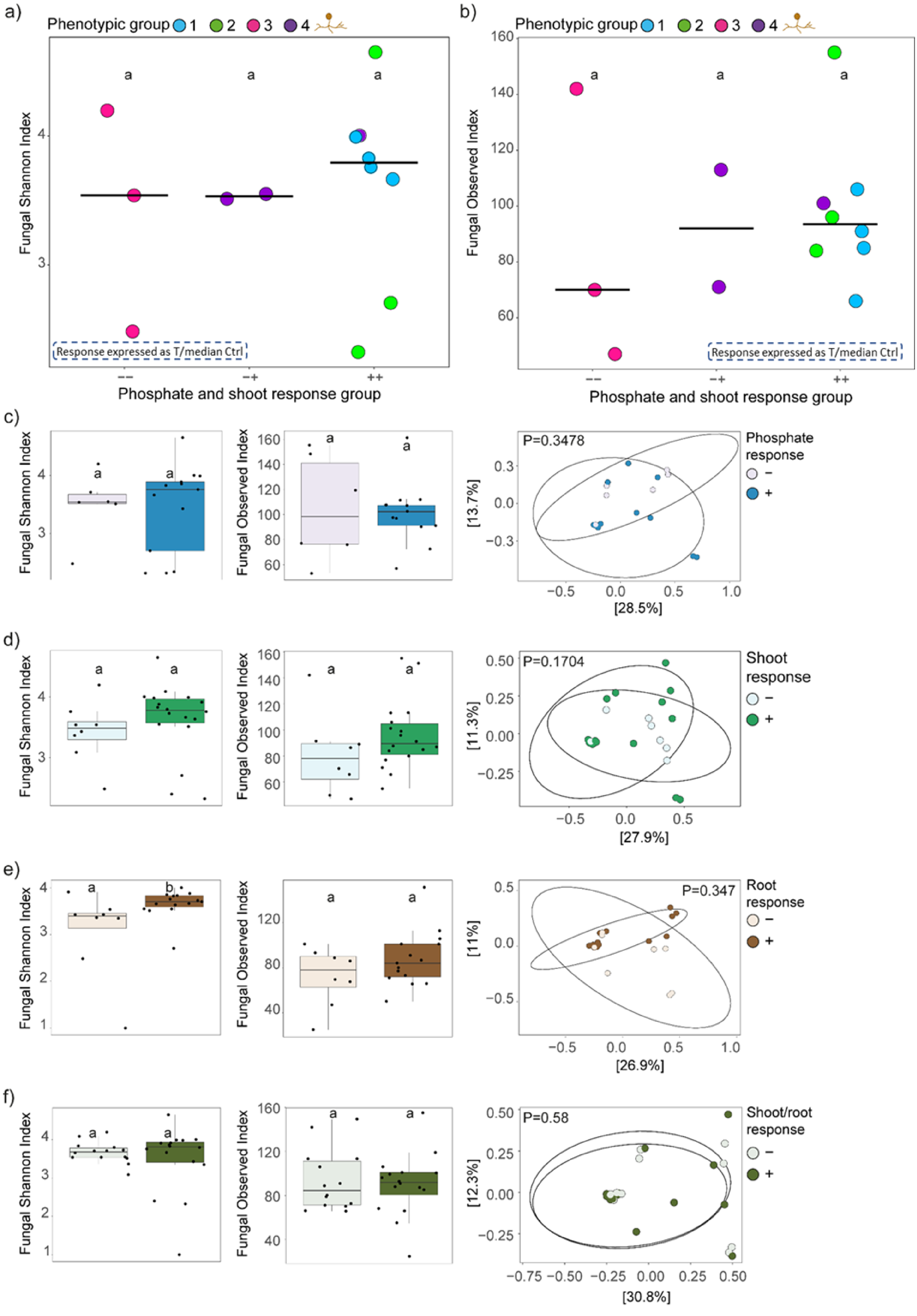

a)
Phenotypic group 1 2 3 4
Fungal Shannon Index
Response expressed as T/median Ctrl
Phosphate and shoot response group
b)
Phenotypic group 1 2 3 4
Fungal Observed Index
Response expressed as T/median Ctrl
Phosphate and shoot response group
c)
P=0.3478
Phosphate response
[28.5%]
[13.7%]
d)
P=0.1704
Shoot response
[27.9%]
[11.3%]
e)
P=0.347
Root response
[26.9%]
[11%]
f)
P=0.58
Shoot/root response
[30.8%]
[12.3%]

Fig. S9. Root-associated fungal community variation at the base of divergent responses. **(a,b)** Fungal Shannon (a) and Observed (b) α-diversity indices across the three response groups (--, +, ++) for leaf phosphate and shoot biomass variation, with each replicate color-coded by phenotypic group. In the boxplots, the horizontal line represents the median, box edges indicate the 25th and 75th percentiles, whiskers extend to 1.5× the interquartile range (IQR), and individual points represent single biological samples. Letters above boxplots indicate statistically homogeneous groups based on Dunn's post-hoc test with FDR-adjusted p-values.

(c-f) Fungal α-diversity (Shannon and Observed indices, left/center) and β-diversity (right) comparing positive and negative responses in leaf phosphate accumulation (c), shoot biomass (d), root biomass (e), and shoot/root biomass variation (f). Boxplot elements are as described in (a,b). For the beta diversity plots, Axis 1 and Axis 2 explain, respectively: 28.5% and 13.7% (c); 27.9% and 11.3% (d); 26.9% and 11% (e); and 30.8% and 12.3% (f) of the total variance. Group separation was assessed by PERMANOVA; none of the comparisons reached statistical significance ($P > 0.05$ in all panels).

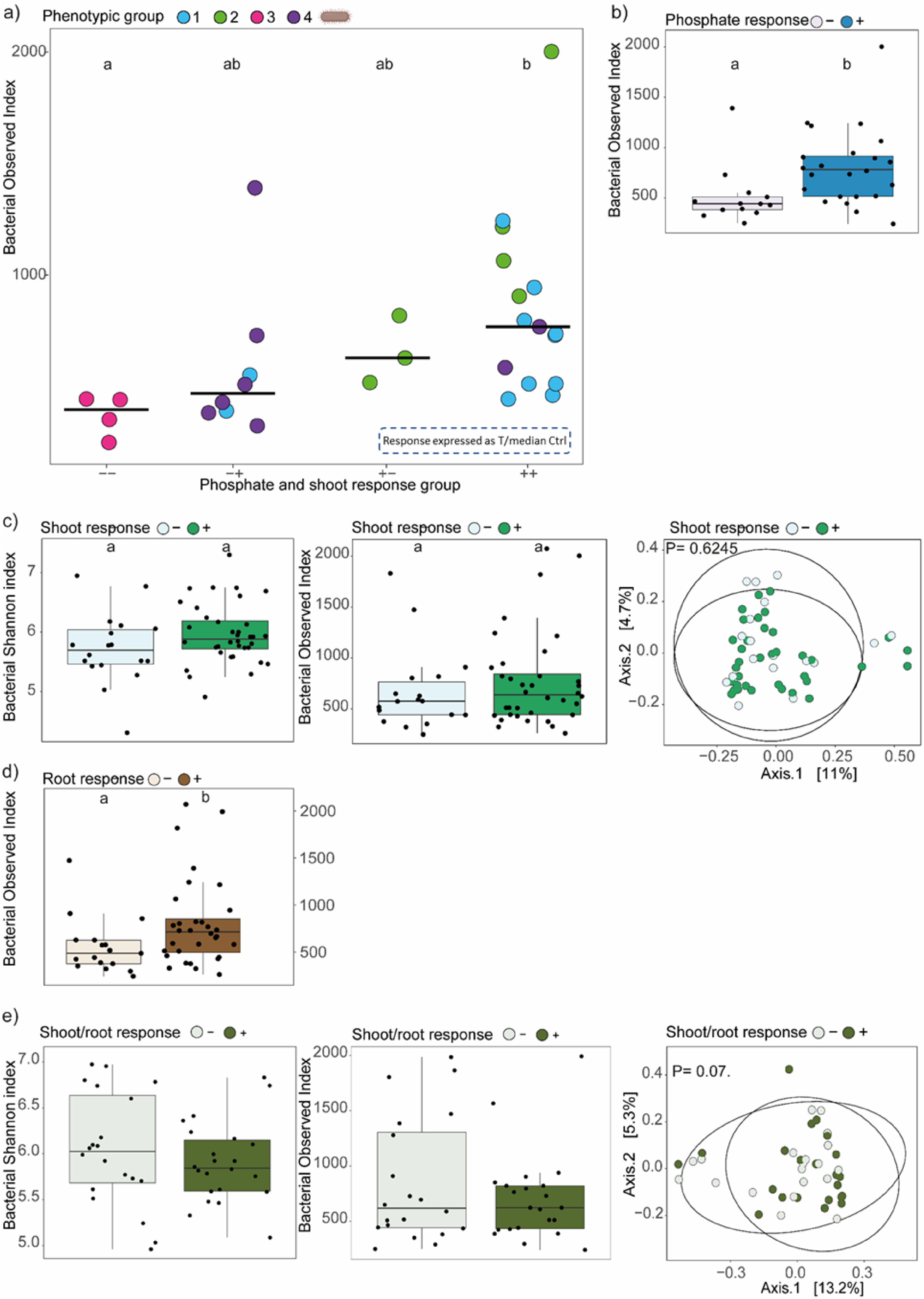

a)
Phenotypic group 1 2 3 4
Bacterial Observed Index
2000
1000
a
ab
ab
b
Response expressed as T/median Ctrl
-- -+ +- ++
Phosphate and shoot response group
b)
Phosphate response − +
Bacterial Observed Index
2000
1500
1000
500
a
b
c)
Shoot response − +
Bacterial Shannon index
7
6
5
a
a
Shoot response − +
Bacterial Observed Index
2000
1500
1000
500
a
a
Shoot response − +
P= 0.6245
Axis.2 [4.7%]
0.4
0.2
0.0
-0.2
-0.25
0.00
0.25
0.50
Axis.1 [11%]
d)
Root response − +
Bacterial Observed Index
a
b
2000
1500
1000
500
e)
Shoot/root response − +
Bacterial Shannon index
7.0
6.5
6.0
5.5
5.0
Shoot/root response − +
Bacterial Observed Index
2000
1500
1000
500
Shoot/root response − +
P= 0.07.
Axis.2 [5.3%]
0.4
0.2
0.0
-0.2
-0.3
0.0
0.3
Axis.1 [13.2%]

Fig. S10. Root-associated bacterial community variation at the base of divergent responses. **(a)** Bacterial Observed α-diversity indices across the four response groups (--, -+, +, ++) for leaf phosphate and shoot biomass variation, with each replicate color-coded by phenotypic group. Colored dots represent individual biological replicates, and the horizontal line indicates the median for each group. Response is expressed as treated/median control (T/median Ctrl). Letters above each group indicate statistically homogeneous groups based on Dunn's post-hoc test with FDR-adjusted p-values.

(b) Bacterial Observed α-diversity index comparing plants with positive (+, blue) and negative (–, gray) responses to leaf phosphate accumulation. In the boxplots, the horizontal line represents the median, box edges indicate the 25th and 75th percentiles, whiskers extend to 1.5× the interquartile range (IQR), and individual points represent single biological samples. Letters above boxplots indicate statistically homogeneous groups based on Dunn's post-hoc test with FDR-adjusted p-values.

(c) Bacterial Shannon and Observed α-diversity indices (left, center) and β-diversity (right) comparing plants with positive (+, green) and negative (–, gray) shoot biomass responses. Boxplot elements are as described in (b). Letters above boxplots indicate statistically homogeneous groups based on Dunn's post-hoc test with FDR-adjusted p-values. For the beta diversity plot, Axis 1 and Axis 2 explain 11% and 4.7% of the total variance, respectively; group separation was assessed by PERMANOVA ($P > 0.05$, not statistically significant).

(d) Bacterial Observed α-diversity index comparing plants with positive (+, brown) and negative (–, gray) root biomass responses. Boxplot elements are as described in (b). Letters above boxplots indicate statistically homogeneous groups based on Dunn's post-hoc test with FDR-adjusted p-values.

(e) Bacterial Shannon and Observed α-diversity indices (left, center) and β-diversity (right) comparing plants with positive (+, olive green) and negative (–, gray) shoot/root biomass responses. Boxplot elements are as described in (b); no significant differences were found between groups (Dunn's post-hoc test, not significant). For the beta diversity plot, Axis 1 and Axis 2 explain 13.2% and 5.3% of the total variance, respectively; group separation was assessed by PERMANOVA ($P > 0.05$, not statistically significant).

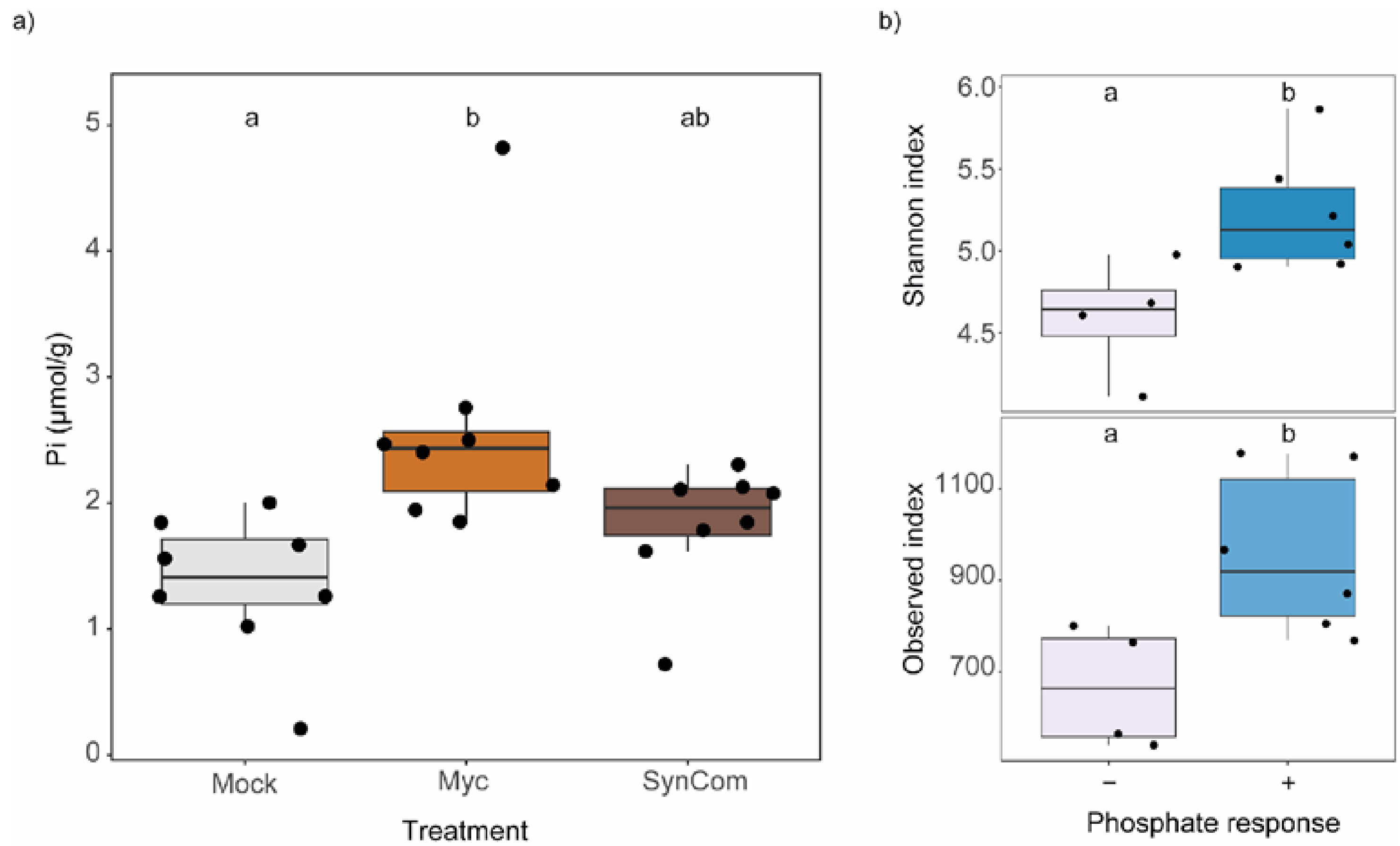

Fig. S11. Leaf Pi response to fungal (Myc) and synthetic microbial community (SynCom) treatments under Pi deficiency. (a) Difference in leaf soluble phosphate (Pi) levels between mock-treated plants (gray), plants treated with the arbuscular mycorrhizal fungus alone (Myc, light brown), and plants treated with the synthetic microbial community (SynCom, dark brown). In the boxplots, the horizontal line represents the median, box edges indicate the 25th and 75th percentiles, whiskers extend to 1.5× the interquartile range (IQR), and individual points represent single biological samples. Letters above boxplots indicate statistically homogeneous groups based on Dunn's post-hoc test with FDR-adjusted p-values.

(b) Shannon (top) and Observed (bottom) bacterial α-diversity indices comparing plants with strong (blue) or low (gray) leaf phosphate accumulation relative to mock-treated plants. Boxplot elements are as described in (a). Letters above boxplots indicate statistically homogeneous groups based on Dunn's post-hoc test with FDR-adjusted p-values.

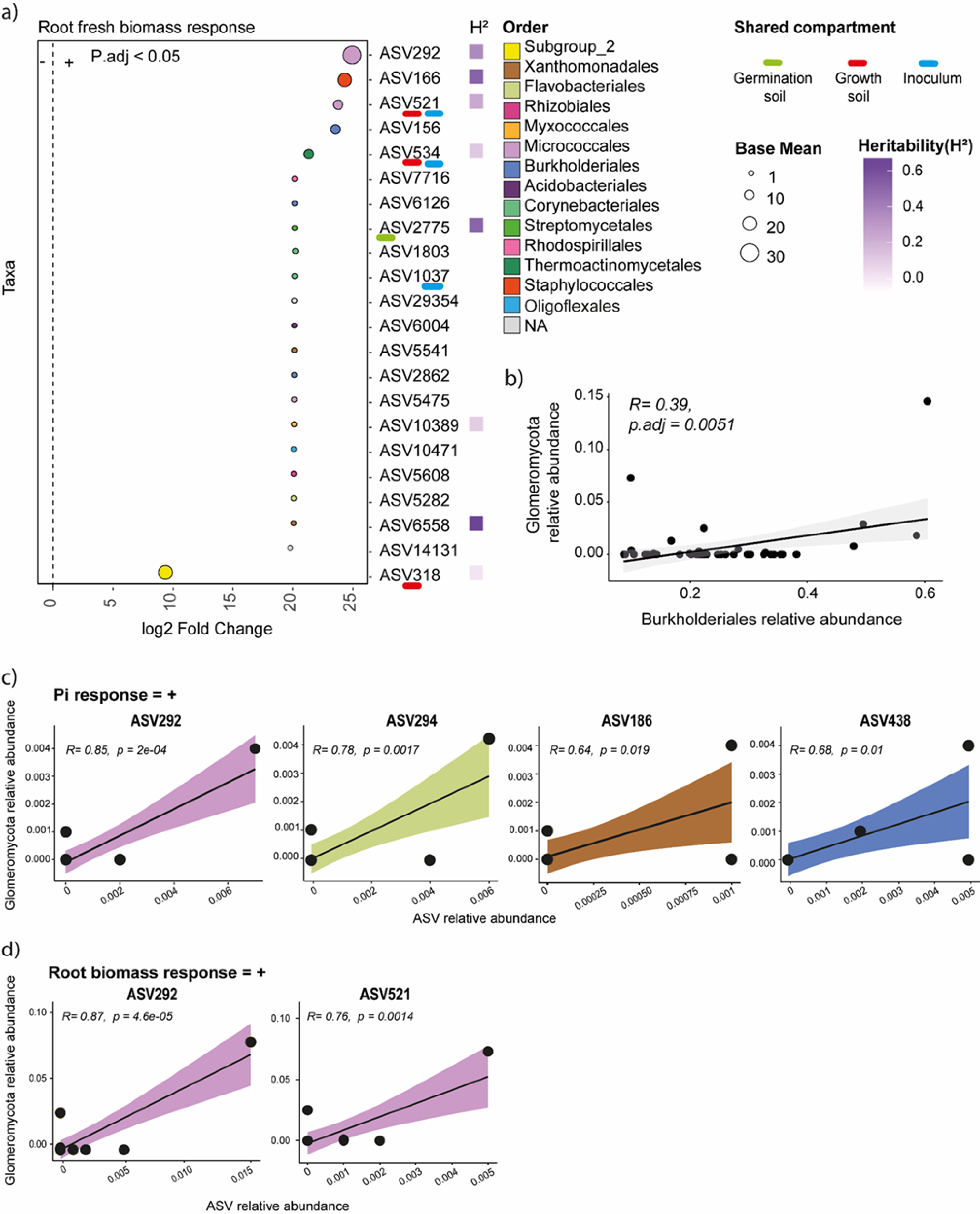

a)
Root fresh biomass response
P.adj < 0.05
- +
Taxa
log2 Fold Change
ASV292
ASV166
ASV521
ASV156
ASV534
ASV7716
ASV6126
ASV2775
ASV1803
ASV1037
ASV29354
ASV6004
ASV5541
ASV2862
ASV5475
ASV10389
ASV10471
ASV5608
ASV5282
ASV6558
ASV14131
ASV318
H²
Order
Subgroup_2
Xanthomonadales
Flavobacteriales
Rhizobiales
Myxococcales
Micrococcales
Burkholderiales
Acidobacteriales
Corynebacteriales
Streptomycetales
Rhodospirillales
Thermoactinomycetales
Staphylococcales
Oligoflexales
NA
Shared compartment
Germination soil
Growth soil
Inoculum
Base Mean
1
10
20
30
Heritability(H²)
0.6
0.4
0.2
0.0
b)
R= 0.39,
p.adj = 0.0051
Glomeromycota relative abundance
Burkholderiales relative abundance
c)
Pi response = +
ASV292
R= 0.85, p = 2e-04
ASV294
R= 0.78, p = 0.0017
ASV186
R= 0.64, p = 0.019
ASV438
R= 0.68, p = 0.01
Glomeromycota relative abundance
ASV relative abundance
d)
Root biomass response = +
ASV292
R= 0.87, p = 4.6e-05
ASV521
R= 0.76, p = 0.0014
Glomeromycota relative abundance
ASV relative abundance

Fig. S12. Correlation analysis between the root-associated fungal community and the bacterial ASVs enriched in the plants with a positive leaf phosphate and root biomass response. (a) Log2 fold-change values for ASVs (amplicon sequence variants), including those with a base mean below 10, showing their abundance in plants with positive (+) and negative (–) variation in root fresh biomass in response to the inoculum. Each dot represents an ASV, with colors indicating its taxonomic order and dot size representing the base mean (average normalized read abundance across samples, as calculated by DESeq2). ASVs shared with the germination soil are highlighted in green, those shared with the growth soil in red, and those shared with the inoculum in blue. Broad-sense heritability (H^2) is shown using a purple color scale, with higher H^2 values in dark purple and lower values in lighter shades.

(b) Pearson's correlation between the relative abundance of Burkholderiales (x-axis) and the relative abundance of Glomeromycota (y-axis); each dot represents a biological replicates. The black line represents the linear regression fit, and the gray shaded area represents the 95% confidence interval ($R = 0.39$, $p.adj = 0.0051$).

(c,d) Pearson's correlation between the relative abundance of ASVs 292, 294, 186, 438, and 521 (x-axis) and the relative abundance of Glomeromycota (y-axis); each dot represents a biological sample. For each panel, the black line represents the linear regression fit, and the shaded confidence interval is color-coded according to the taxonomic order of the corresponding ASV, as shown in the legend in (a). R and p values are indicated in each panel. Panel (c) shows ASVs enriched in plants with a positive phosphate uptake response, while panel (d) shows those enriched in plants with increased root biomass. Only statistically significant correlations are shown.
