# Supplementary material for: Plant genetic and root‐associated microbial diversity modulate Lactuca sativa responsiveness to a soil inoculum under phosphate deficiency
Source: New Phytol. 2026 Sep 2;252(4):1584–601. doi: 10.1111/nph.71529 (PMC13669811; doi:10.1111/nph.71529)
Supplement: Supplementary file 2 [file NPH-252-1584-s003.pdf]

SupplementaryScript

Capparotto et al., 2025

2025-03-20

```
knitr::kable(head(mtcars), "latex", longtable = TRUE)
```

1 - import and manage data from Supplementary Table 5

```
###install the following packages and/or load them

library(ggExtra)
library(tidyverse)
```

```
## ── Attaching core tidyverse packages ──────────────────────── tidyverse 2.0.0 ──
## ✔ dplyr     1.1.4     ✔ readr     2.1.5
## ✔ forcats   1.0.0     ✔ stringr   1.5.2
## ✔ ggplot2   4.0.0     ✔ tibble    3.3.0
## ✔ lubridate 1.9.4     ✔ tidyr     1.3.1
## ✔ purrr     1.1.0
## ── Conflicts ────────────────────────────────────────── tidyverse_conflicts() ──
## ✖ dplyr::filter() masks stats::filter()
## ✖ dplyr::lag()    masks stats::lag()
## ℹ Use the conflicted package (<http://conflicted.r-lib.org/>) to force all conflic
ts to become errors
```

```
library(multcomp)
```

```
## Caricamento del pacchetto richiesto: mvtnorm
## Caricamento del pacchetto richiesto: survival
## Caricamento del pacchetto richiesto: TH.data
## Caricamento del pacchetto richiesto: MASS
##
## Caricamento pacchetto: 'MASS'
##
## Il seguente oggetto è mascherato da 'package:dplyr':
##
##     select
##
##
## Caricamento pacchetto: 'TH.data'
##
## Il seguente oggetto è mascherato da 'package:MASS':
##
##     geyser
```

```
library(ggpubr)
library(rstatix)
```

```
##
## Caricamento pacchetto: 'rstatix'
##
## Il seguente oggetto è mascherato da 'package:MASS':
##
##     select
##
## Il seguente oggetto è mascherato da 'package:stats':
##
##     filter
```

```
library(patchwork)
```

```
##
## Caricamento pacchetto: 'patchwork'
##
## Il seguente oggetto è mascherato da 'package:MASS':
##
##     area
```

```
library(dplyr)

###your path
#use Suppelementary_table_5
data <- read.csv("C:/Users/Alber/Desktop/DOTTORATO/LATTUGA/LANDLAB/P/Phosphate.csv",
                 header=T,sep = ";", na.string=c("na", "NA", "null")) #replace with y
our path
str(data)
```

```
## 'data.frame':    897 obs. of  29 variables:
##  $ position                   : int  570 592 640 514 579 624 636 525 593 608 ...
##  $ genotype                   : chr  "1" "1" "1" "1" ...
##  $ treatment                  : chr  "K" "K" "K" "T" ...
##  $ nord.sud                   : chr  "sud" "sud" "sud" "centro" ...
##  $ pmol.in.16.ul              : num  196 335 296 284 266 ...
##  $ plate                      : int  4 4 4 4 4 4 4 4 4 4 ...
##  $ date                       : chr  "14/03/2023" "14/03/2023" "14/03/2023" "14/0
3/2023" ...
##  $ rack                       : int  1 1 1 1 1 1 1 1 1 1 ...
##  $ variety                    : chr  "Butterhead" "Butterhead" "Butterhead" "Butt
erhead" ...
##  $ leaf.disk.weight           : num  0.32 0.46 0.49 0.39 0.43 0.53 0.38 0.42 0.48
0.44 ...
##  $ Pi                         : num  38.4 45.5 37.8 45.5 38.7 ...
##  $ total.Pi                   : chr  "674.3578125" "1020.173913" "699.2244898" "7
46.4102564" ...
##  $ S.R                        : chr  "7.032" "7.943262411" "9.168316832" "11.7142
8571" ...
##  $ shoot                      : num  17.6 22.4 18.5 16.4 11.1 ...
##  $ root                       : num  2.5 2.82 2.02 1.4 1.16 1.94 1.56 1.72 2.1 2.
44 ...
##  $ Digital.biomass..mm..      : num  200668 375358 101679 106289 134570 ...
##  $ greenness.average          : num  0.53 0.55 0.543 0.531 0.508 ...
##  $ Height..mm.                : num  20 23.8 19.7 14.9 17.3 ...
##  $ Height.Max..mm.            : num  31.7 35.5 28.2 23.7 30.8 ...
##  $ hue.average....            : num  97 102.3 100.5 100.7 96.2 ...
##  $ Leaf.angle....             : num  36.4 38.7 39.6 42.1 40 ...
##  $ Leaf.area..mm..            : num  10021 15758 5152 7144 7758 ...
##  $ Leaf.area.index..mm..mm..  : num  0.148 0.233 0.076 0.106 0.115 0.012 0.159 0.
275 0.245 0.307 ...
##  $ Leaf.area..projected...mm.. : num  5939 9833 3275 4785 4991 ...
##  $ Leaf.inclination..mm..mm..  : num  1.69 1.6 1.57 1.49 1.55 ...
##  $ Light.penetration.depth..mm.: num  17.4 20.9 18.3 13.1 15.6 ...
##  $ NDVI.average               : num  0.643 0.705 0.678 0.659 0.615 ...
##  $ NPCI.average               : num  0.555 0.435 0.471 0.455 0.537 ...
##  $ PSRI.average               : num  0.148 0.101 0.118 0.123 0.157 ...
```

```
#Define the two levels of the treatment

data %>%
  filter (treatment == "K" | treatment== "T") %>%
  filter(genotype!="xxx") %>% ###remove the xxx plants (present in the space)
  droplevels()  -> data


#transform into the right factor

data$position <- as.factor(data$position)
data$genotype <- as.factor(data$genotype)
data$treatment <- as.factor(data$treatment)
data$plate <-as.factor(data$plate)
data$date<-as.Date(data$date)
data$rack <-as.factor(data$rack)
data$shoot<- as.numeric(data$shoot)
data$root<- as.numeric(data$root)
data$variety<- as.factor(data$variety)
data$leaf.disk.weight<-as.numeric(data$leaf.disk.weight)
data$S.R<- as.numeric(data$S.R)
```

```
## Warning: NA introdotti per coercizione
```

```
data$total.Pi<- as.numeric(data$total.Pi)
```

```
## Warning: NA introdotti per coercizione
```

2 - Stat tables

```
#1. file with statistics

data_stats  <- data %>%
  group_by(genotype, treatment) %>%
 summarise(mediaPi = mean(Pi),
            stdevPi = sd(Pi),
            sterrPi = sd(Pi)/sqrt(length(Pi)),
           mediaPi.tot = mean(total.Pi),
            stdevPi.tot = sd(total.Pi),
            sterrPi.tot = sd(total.Pi)/sqrt(length(total.Pi)),
           mediaS.R = mean(S.R),
            stdevS.R = sd(S.R),
            sterrS.R = sd(S.R)/sqrt(length(S.R)),
           mediashoot = mean(shoot),
            stdevshoot = sd(shoot),
            sterrshoot = sd(shoot)/sqrt(length(shoot)),
           mediaroot = mean(root),
            stdevroot = sd(root),
            sterrroot = sd(root)/sqrt(length(root)),
           mediaDigital.biomass..mm.. = mean(Digital.biomass..mm..),
            stdevDigital.biomass..mm.. = sd(Digital.biomass..mm..),
            sterrDigital.biomass..mm.. = sd(Digital.biomass..mm..)/sqrt(length(Digita
l.biomass..mm..)),
           mediaHeight..mm. = mean(Height..mm.),
            stdevHeight..mm. = sd(Height..mm.),
            sterrHeight..mm. = sd(Height..mm.)/sqrt(length(Height..mm.)),
           mediahue.average.... = mean(hue.average....),
            stdevhue.average.... = sd(hue.average....),
            sterrhue.average.... = sd(hue.average....)/sqrt(length(hue.average....)),
           mediaLeaf.angle.... = mean(Leaf.angle....),
            stdevLeaf.angle.... = sd(Leaf.angle....),
            sterrLeaf.angle.... = sd(Leaf.angle....)/sqrt(length(Leaf.angle....)),
           mediaLeaf.area..mm.. = mean(Leaf.area..mm..),
            stdevLeaf.area..mm.. = sd(Leaf.area..mm..),
            sterrLeaf.area..mm.. = sd(Leaf.area..mm..)/sqrt(length(Leaf.area..mm..)),
           mediaLeaf.inclination..mm..mm.. = mean(Leaf.inclination..mm..mm..),
            stdevLeaf.inclination..mm..mm.. = sd(Leaf.inclination..mm..mm..),
            sterrLeaf.inclination..mm..mm.. = sd(Leaf.inclination..mm..mm..)/sqrt(len
gth(Leaf.inclination..mm..mm..)),
           mediaLeaf.area..projected...mm.. = mean(Leaf.area..projected...mm..),
            stdevLeaf.area..projected...mm.. = sd(Leaf.area..projected...mm..),
            sterrLeaf.area..projected...mm.. = sd(Leaf.area..projected...mm..)/sqrt(l
ength(Leaf.area..projected...mm..)),
           mediaNDVI.average = mean(NDVI.average),
            stdevNDVI.average = sd(NDVI.average),
            sterrNDVI.average = sd(NDVI.average)/sqrt(length(NDVI.average)),
           mediaNPCI.average = mean(NPCI.average),
            stdevNPCI.average = sd(NPCI.average),
            sterrNPCI.average = sd(NPCI.average)/sqrt(length(NPCI.average)),
           mediaPSRI.average = mean(PSRI.average),
            stdevPSRI.average = sd(PSRI.average),
            sterrPSRI.average = sd(PSRI.average)/sqrt(length(PSRI.average)),
           medialeaf.disk.weight = mean(leaf.disk.weight),
            stdevleaf.disk.weight = sd(leaf.disk.weight),
            sterrleaf.disk.weight = sd(leaf.disk.weight)/sqrt(length(leaf.disk.weigh
```

```
t)),
           )
```

```
## `summarise()` has grouped output by 'genotype'. You can override using the
## `.groups` argument.
```

```
#2. file with medians of Ctrl and Treated genotypes

data_stats_medians  <- data %>%
  group_by(genotype, treatment) %>%
 summarise(medianPi = median(Pi),
           medianS.R = median(S.R),
           medianshoot = median(shoot),
           medianroot = median(root),
           medianDigital.biomass..mm.. = median(Digital.biomass..mm..),
           medianHeight..mm. = median(Height..mm.),
           medianhue.average.... = median(hue.average....),
           medianLeaf.angle.... = median(Leaf.angle....),
           medianLeaf.area..mm.. = median(Leaf.area..mm..),
           medianLeaf.inclination..mm..mm.. = median(Leaf.inclination..mm..mm..),
           medianLeaf.area..projected...mm.. = median(Leaf.area..projected...mm..),
           medianNDVI.average = median(NDVI.average),
           medianNPCI.average = median(NPCI.average),
           medianPSRI.average = median(PSRI.average),
           medianGrenness = median(greenness.average))
```

```
## `summarise()` has grouped output by 'genotype'. You can override using the
## `.groups` argument.
```

```
# 3. pivot table to construct the heat map

data_stats_medians  %>%
  pivot_wider(
    names_from = treatment,
    values_from = c(medianPi, medianS.R,medianshoot,medianroot, medianDigital.biomas
s..mm.., medianHeight..mm.,medianhue.average.... , medianLeaf.angle...., medianLeaf.a
rea..mm.., medianLeaf.inclination..mm..mm.., medianLeaf.area..projected...mm.., media
nNDVI.average, medianNPCI.average,  medianPSRI.average,medianGrenness)
  ) -> data_stat_pivot

# 4. Pivoted table with the median of the ratios of Treated/Ctrls

data_stat_pivot %>%
  group_by(genotype) %>%
      summarise(ratio_Pi = medianPi_T/medianPi_K,
                ratio_S.R = medianS.R_T/medianS.R_K,
                ratio_shoot = medianshoot_T/medianshoot_K,
                ratio_root = medianroot_T/medianroot_K,
                ratio_height = medianHeight..mm._T/medianHeight..mm._K,
                ratio_hue = medianhue.average...._T/medianhue.average...._K,
                ratio_digital_biomass = medianDigital.biomass..mm.._T/medianDigital.b
iomass..mm.._K,
                ratio_leaf_angle =medianLeaf.angle...._T/medianLeaf.angle...._K,
                ratio_leaf_area = medianLeaf.area..mm.._T/medianLeaf.area..mm.._K,
                ratio_leaf_inclination = medianLeaf.inclination..mm..mm.._T/medianLea
f.inclination..mm..mm.._K,
                ratio_leaf_area_projected= medianLeaf.area..projected...mm.._T/median
Leaf.area..projected...mm.._K,
                ratio_NDVI = medianNDVI.average_T/medianNDVI.average_K,
                ratio_NPCI = medianNPCI.average_T/medianNPCI.average_K,
                ratio_PSRI = medianPSRI.average_T/medianPSRI.average_K,
                ratio_grenness = medianGrenness_T/medianGrenness_K) -> data_ratio

# 5. Table with response intervals

# Remove columns 6, and columns 8 to 12

data_ratio_median   <- data_ratio  %>%
  mutate(interval_Pi = case_when(ratio_Pi < 0.80 ~ "negative",
                              ratio_Pi > 1.20 ~ "positive",
                              ratio_Pi >= 0.80 & ratio_Pi<=1.20 ~ "non responding"),
         interval_shoot = case_when(ratio_shoot < 0.80 ~ "negative",
                              ratio_shoot > 1.20 ~ "positive",
                              ratio_shoot >= 0.80 & ratio_shoot<=1.20 ~ "non respondi
ng"))

# 6. Create a new column in the dataset based on conditions from the 'interval_Pi' an
d 'interval_shoot' columns

data_response <-data_ratio_median %>%
   mutate(interval_response = case_when(interval_Pi == "positive" & interval_shoot ==
"positive" ~ "++",
                              interval_Pi == "positive" & interval_shoot == "negativ
e" ~ "+-",
```

```
                                    interval_Pi == "negative" & interval_shoot == "negativ
e" ~ "--",
                                    interval_Pi == "negative" & interval_shoot == "positiv
e" ~ "-+",
                                    interval_Pi == "positive" & interval_shoot == "non resp
onding" ~ "+/",
                                    interval_Pi == "negative" & interval_shoot == "non resp
onding" ~ "-/",
                                    interval_Pi == "non responding" & interval_shoot == "no
n responding" ~ "//",
                                    interval_Pi == "non responding" & interval_shoot == "ne
gative" ~ "/-",
                                    interval_Pi == "non responding" & interval_shoot == "po
sitive" ~ "/+"))
```

3 - leaf [Pi] statistics

```
#1. Check for the best linear model

##model 1
mod1<- lm(Pi ~ genotype*treatment, data = data) -> model
##model 2
mod2<- lm(Pi ~ genotype*treatment + leaf.disk.weight, data = data) -> model
anova(mod1, mod2) #the second one is better
```

	Res.Df<br><dbl>	RSS<br><dbl>	Df<br><dbl>	Sum of Sq<br><dbl>	F<br><dbl>	Pr(>F)<br><dbl>
1	610	42033.34	NA	NA	NA	NA
2	609	39164.40	1	2868.934	44.61145	5.423842e-11

2 rows

```
#2. check normality and homogeneity of variance

## Build the linear model
lmod2<- lm(Pi ~ genotype*treatment + leaf.disk.weight, data = data) -> model

## Create a QQ plot of residuals and check for NORMALITY
ggqqplot(residuals(model))      #plot
```

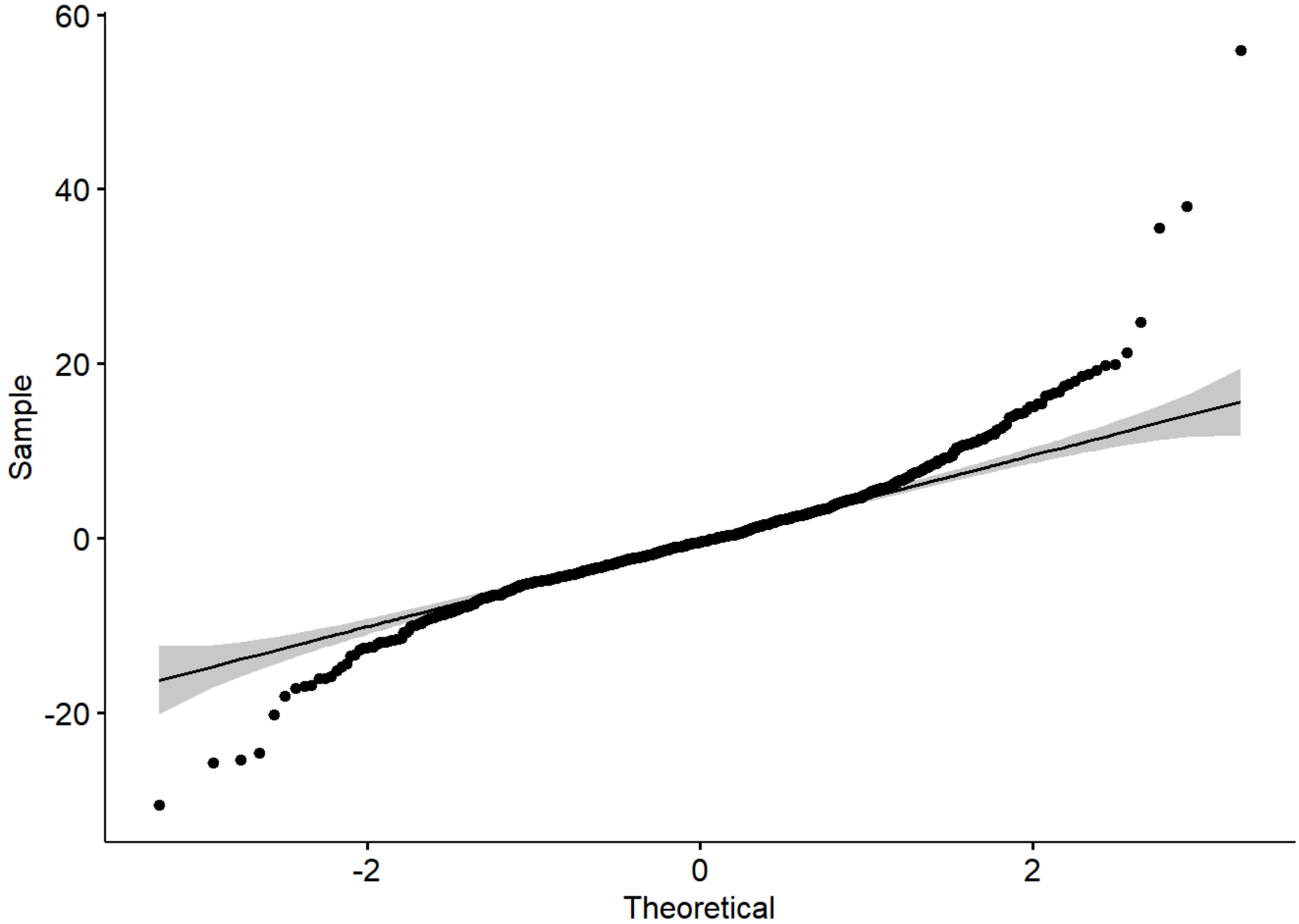


```
shapiro_test(residuals(model))  #significance
```

variable<br><chr>	statistic<br><dbl>	p.value<br><dbl>
residuals(model)	0.9200558	5.676255e-21

1 row

```
##check Homogeneity of VARIANCE
plot(model, 1) ##for Pi, the distribution is not normal
```

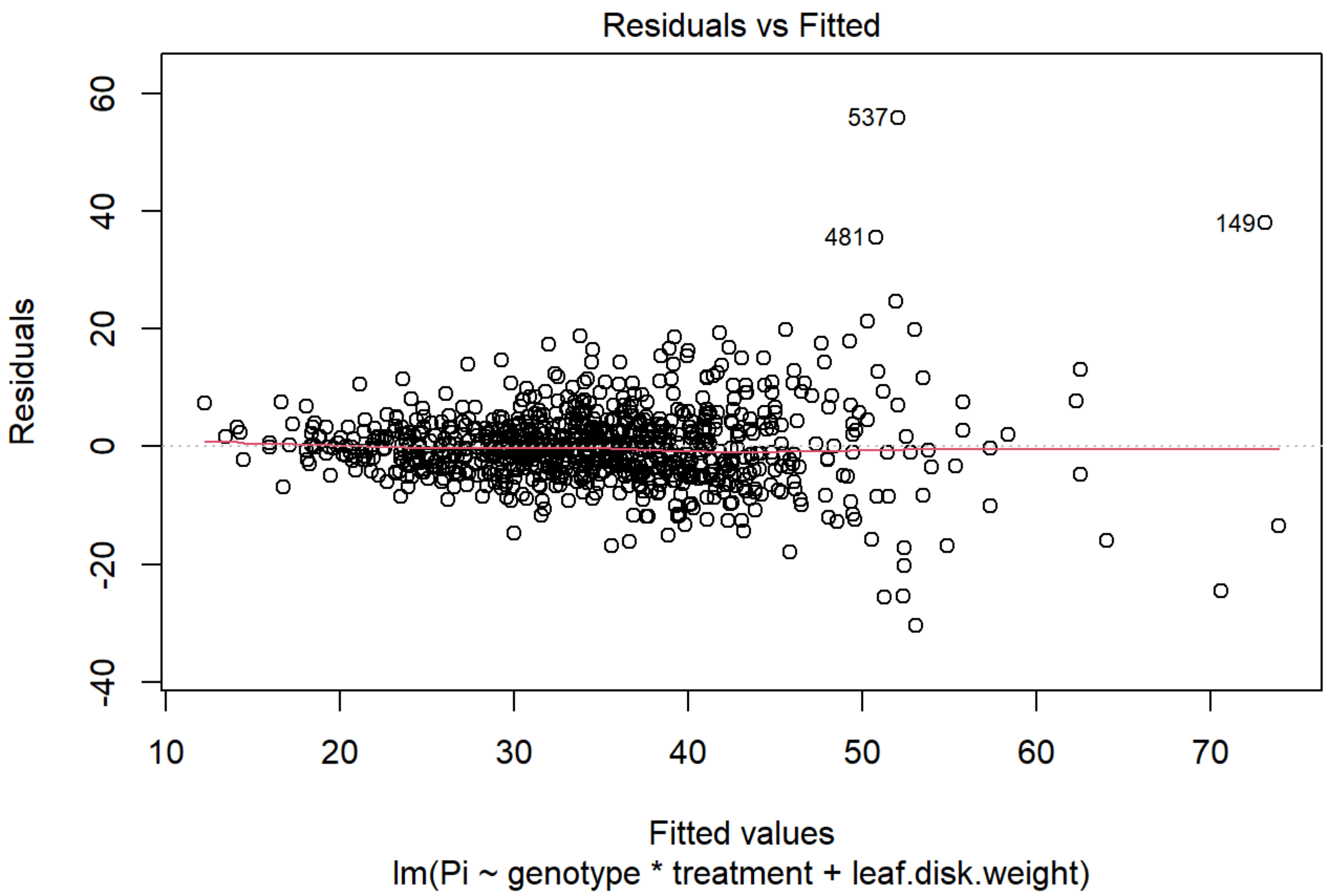


```
#3. Remove the outliers

##Identify the outliers
data%>%
  identify_outliers(Pi)-> data_Pi_outliers

##exclude the outliers
data %>%
  filter(!position %in% data_Pi_outliers$position) -> data_Pi_outliers_F

## Build the linear model, excluding outliers
lm(Pi ~ genotype*treatment + leaf.disk.weight, data = data_Pi_outliers_F) -> model_fi
lt

## Create a QQ plot of residuals and check for normality
ggqqplot(residuals(model_filt ))
```

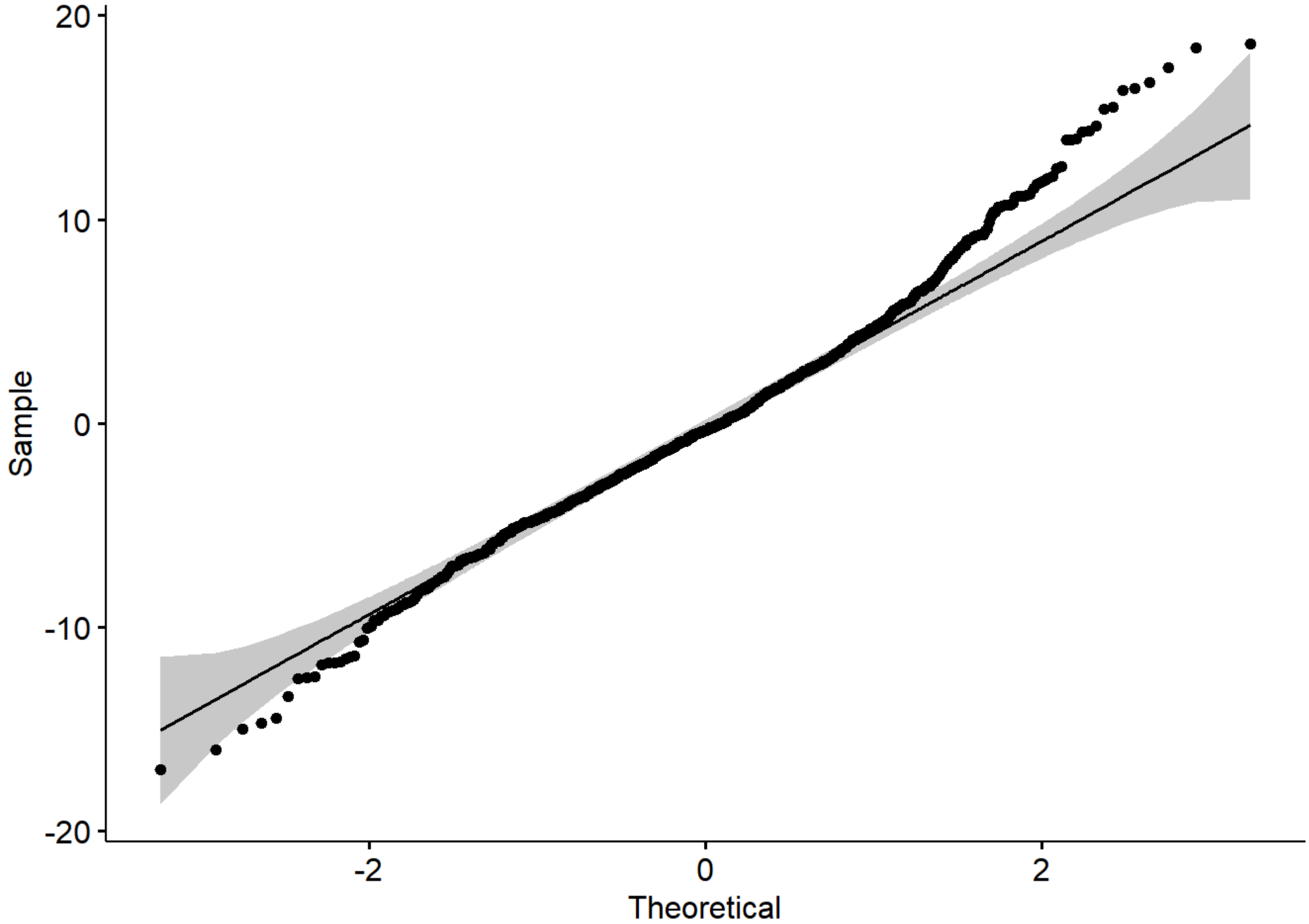


```
shapiro_test(residuals(model_filt ))
```

variable <chr>	statistic <dbl>	p.value <dbl>
residuals(model_filt)	0.9856543	2.594804e-07

1 row

```
##check Homogeneity of variance
plot(model_filt, 1)
```

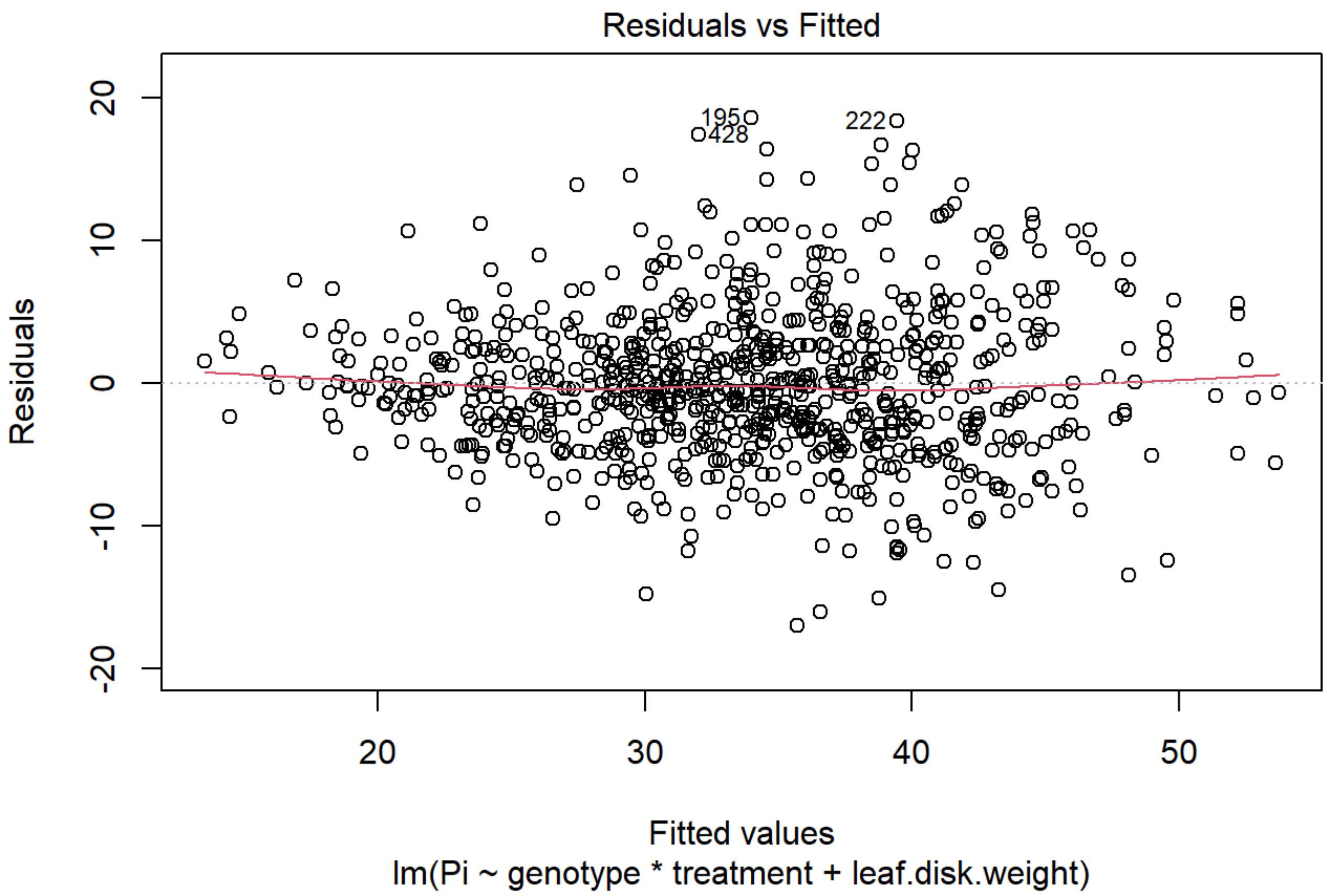


```
##there is not normality homogeneity of variance, then we will apply a non-parametric
test

#4. kruskal wallis
data %>%
  group_by(genotype) %>%
  kruskal_test(Pi ~ treatment)
```

	genotype <fct>	.y. <chr>	n <int>	statistic <dbl>	df <int>	p <dbl>	method <chr>
1	1	Pi	7	0.12500000	1	0.7240	Kruskal-Wallis
2	100	Pi	7	1.12500000	1	0.2890	Kruskal-Wallis
3	101	Pi	7	2.00000000	1	0.1570	Kruskal-Wallis
4	102	Pi	7	3.12500000	1	0.0771	Kruskal-Wallis
5	103	Pi	7	3.12500000	1	0.0771	Kruskal-Wallis
6	104	Pi	7	0.12500000	1	0.7240	Kruskal-Wallis
7	105	Pi	7	0.12500000	1	0.7240	Kruskal-Wallis
8	106	Pi	7	3.12500000	1	0.0771	Kruskal-Wallis
9	107	Pi	7	0.50000000	1	0.4800	Kruskal-Wallis
10	108	Pi	7	0.50000000	1	0.4800	Kruskal-Wallis

1-10 of 127 rows Previous **1** 2 3 4 5 6 ... 13 Next

```
#5. Dunn's post-hoc test : Supplementary table 14

data %>%
   group_by(genotype) %>%
  dunn_test(Pi ~ treatment, p.adjust.method = "fdr")
```

	genotype	.y.	group1	group2	n1	n2	statistic	p	p.adj
	<fct>	<chr>	<chr>	<chr>	<int>	<int>	<dbl>	<dbl>	<dbl>
1	1	Pi	K	T	3	4	0.3535534	0.72367361	0.72367361
2	100	Pi	K	T	3	4	-1.0606602	0.28884437	0.28884437
3	101	Pi	K	T	3	4	1.4142136	0.15729921	0.15729921
4	102	Pi	K	T	3	4	1.7677670	0.07709987	0.07709987
5	103	Pi	K	T	3	4	-1.7677670	0.07709987	0.07709987
6	104	Pi	K	T	3	4	0.3535534	0.72367361	0.72367361
7	105	Pi	K	T	3	4	0.3535534	0.72367361	0.72367361
8	106	Pi	K	T	3	4	-1.7677670	0.07709987	0.07709987
9	107	Pi	K	T	3	4	-0.7071068	0.47950012	0.47950012
10	108	Pi	K	T	3	4	0.7071068	0.47950012	0.47950012

1-10 of 127 rows | 1-10 of 11 columns Previous **1** 2 3 4 5 6 ... 13 Next

4 - Shoot biomass statistics

```
#1. Check for the best linear model

##model 1
mod1<- lm(shoot ~ genotype*treatment, data = data) -> model
##model 2
mod2<- lm(shoot ~ genotype*treatment + nord.sud, data = data) -> model

anova(mod1, mod2) #the second one is better
```

	Res.Df	RSS	Df	Sum of Sq	F	Pr(>F)
	<dbl>	<dbl>	<dbl>	<dbl>	<dbl>	<dbl>
1	610	16578.49	*NA*	*NA*	*NA*	*NA*
2	608	16345.54	2	232.9455	4.3324	0.0135439

2 rows

```
#2. check normality and homogeneity of variance

## Build the linear model
lmod2<- lm(shoot ~ genotype*treatment + nord.sud, data = data) -> model

## Create a QQ plot of residuals and check for NORMALITY
ggqqplot(residuals(model))      #plot
```

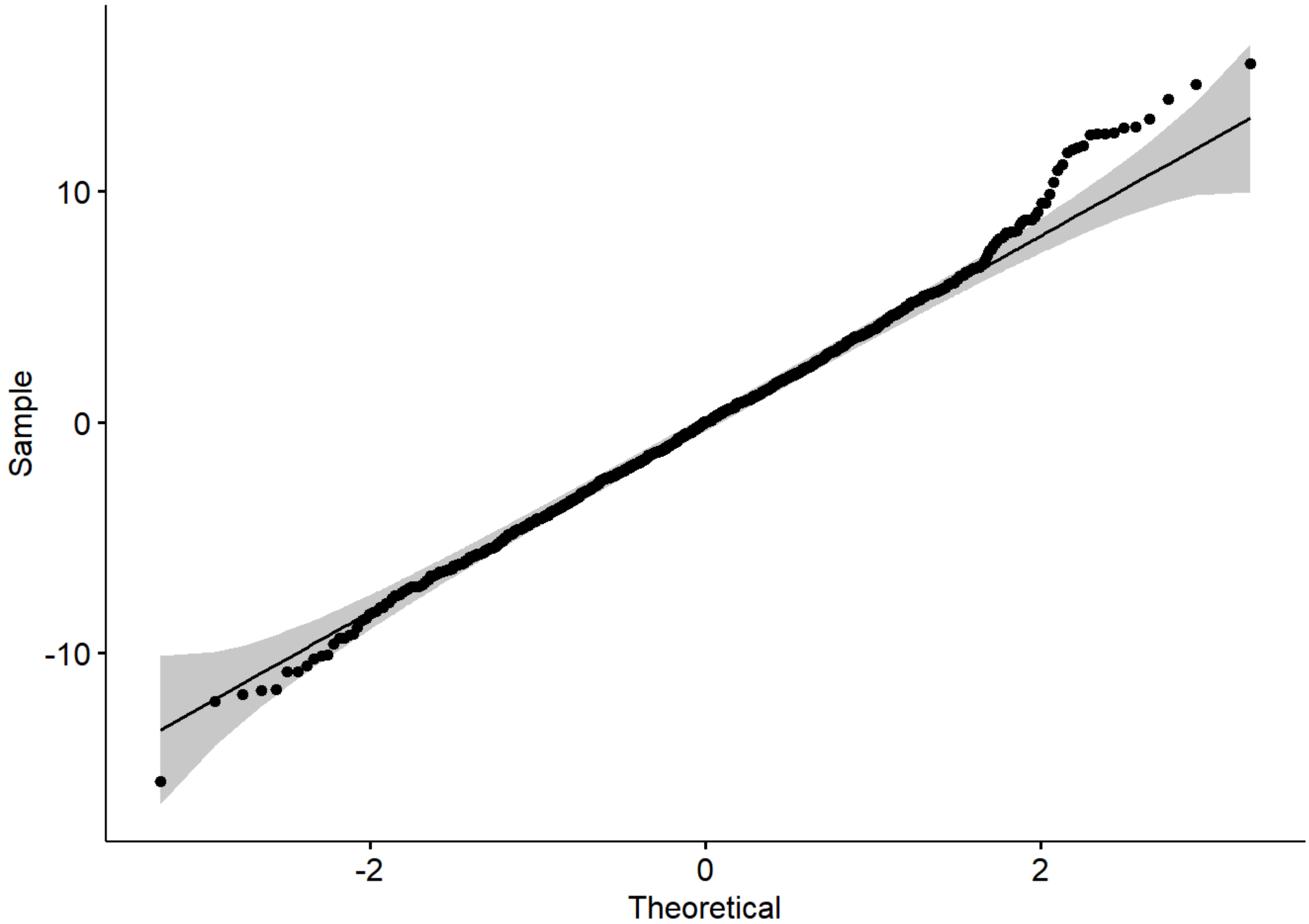


```
shapiro_test(residuals(model))  #significance
```

variable	statistic	p.value
<chr>	<dbl>	<dbl>
residuals(model)	0.9937693	0.001178367

1 row

```
##check Homogeneity of VARIANCE
plot(model, 1)
```

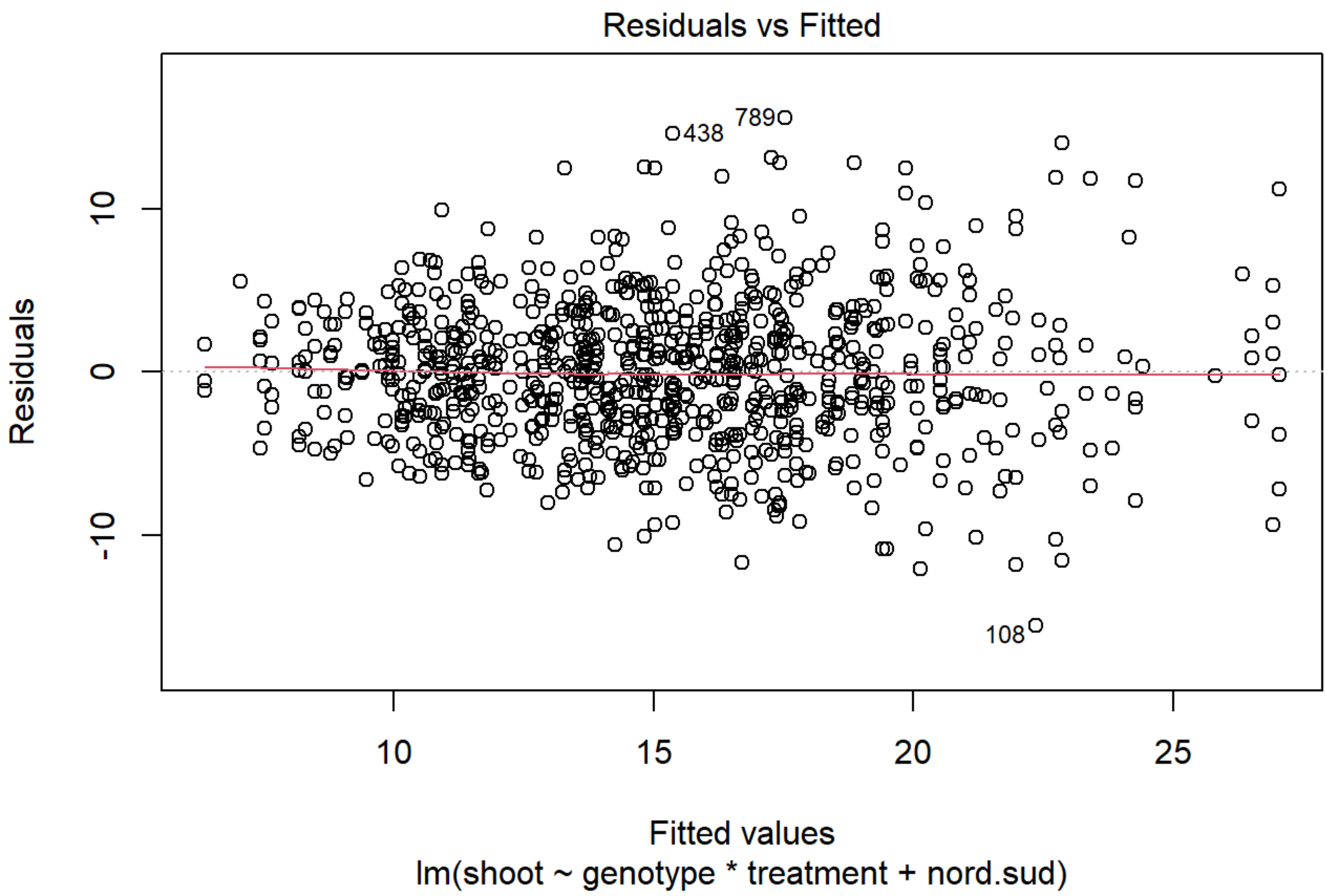


```
##for shoot biomass, the distribution is not normal

#3. Remove the outliers

##identify outliers
data%>%
  identify_outliers(shoot)-> data_shoot_outliers

##remove outliers
data %>%
  filter(!position %in% data_shoot_outliers$position) -> data_shoot_outliers_F

##Build the linear model, excluding outliers
lm(shoot ~ genotype*treatment + nord.sud, data = data_shoot_outliers_F) -> model_filt

##Create a QQ plot of residuals and check for normality
ggqqplot(residuals(model_filt ))
```

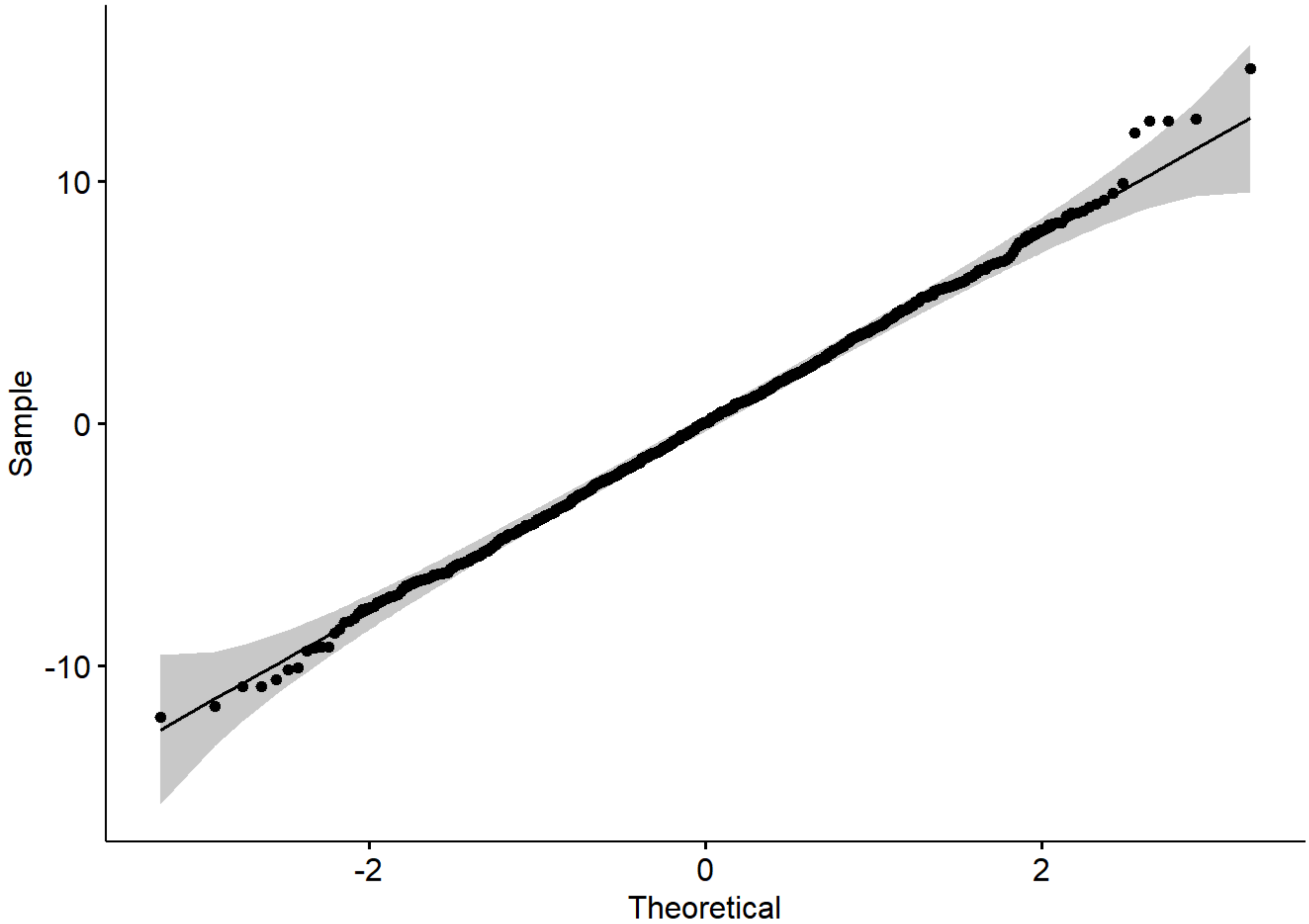


```
shapiro_test(residuals(model_filt ))
```

variable <chr>	statistic <dbl>	p.value <dbl>
residuals(model_filt)	0.9983588	0.6128949

1 row

```
##check Homogeneity of variance
plot(model_filt, 1)
```

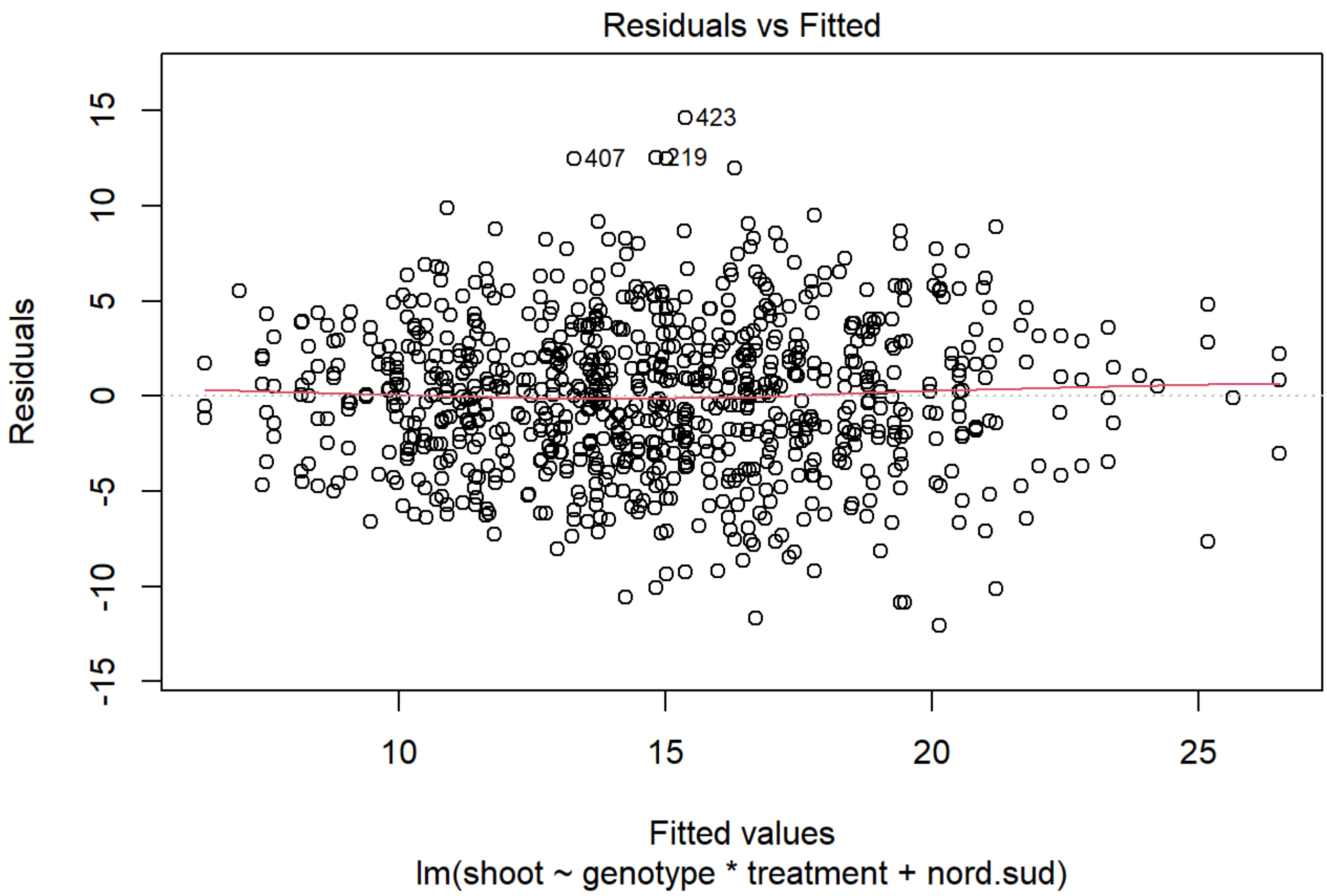


```
##there is not normality and homogeneity of variance, then we will apply a non-parametric test

#4.kruskal wallis

data %>%
  group_by(genotype) %>%
  kruskal_test(shoot ~ treatment)
```

	genotype <fct>	.y. <chr>	n <int>	statistic <dbl>	df <int>	p <dbl>	method <chr>
1	1	shoot	7	2.00000000	1	0.1570	Kruskal-Wallis
2	100	shoot	7	4.50000000	1	0.0339	Kruskal-Wallis
3	101	shoot	7	0.50000000	1	0.4800	Kruskal-Wallis
4	102	shoot	7	0.00000000	1	1.0000	Kruskal-Wallis
5	103	shoot	7	0.50000000	1	0.4800	Kruskal-Wallis
6	104	shoot	7	0.00000000	1	1.0000	Kruskal-Wallis
7	105	shoot	7	0.12500000	1	0.7240	Kruskal-Wallis
8	106	shoot	7	1.12500000	1	0.2890	Kruskal-Wallis
9	107	shoot	7	1.12500000	1	0.2890	Kruskal-Wallis
10	108	shoot	7	2.00000000	1	0.1570	Kruskal-Wallis

1-10 of 127 rows Previous 1 2 3 4 5 6 ... 13 Next

```
#5.Dunn's post-hoc test : Supplementary table 15
data %>%
   group_by(genotype) %>%
  dunn_test(shoot ~ treatment, p.adjust.method = "fdr")
```

	genotype <fct>	.y. <chr>	group1 <chr>	group2 <chr>	n1 <int>	n2 <int>	statistic <dbl>	p <dbl>	p.adj <dbl>
1	1	shoot	K	T	3	4	-1.4142136	0.15729921	0.15729921
2	100	shoot	K	T	3	4	2.1213203	0.03389485	0.03389485
3	101	shoot	K	T	3	4	-0.7071068	0.47950012	0.47950012
4	102	shoot	K	T	3	4	0.0000000	1.00000000	1.00000000
5	103	shoot	K	T	3	4	-0.7071068	0.47950012	0.47950012
6	104	shoot	K	T	3	4	0.0000000	1.00000000	1.00000000
7	105	shoot	K	T	3	4	-0.3535534	0.72367361	0.72367361
8	106	shoot	K	T	3	4	1.0606602	0.28884437	0.28884437
9	107	shoot	K	T	3	4	1.0606602	0.28884437	0.28884437
10	108	shoot	K	T	3	4	1.4142136	0.15729921	0.15729921

1-10 of 127 rows | 1-10 of 11 columns Previous 1 2 3 4 5 6 ... 13 Next

5 - Figure 1A left

```
data_Figure1A<- data %>%
  filter(genotype %in% c("38", "57", "69", "53", "59", "136"))

data_Figure1A$genotype <- factor(data_Figure1A$genotype, levels = c("53", "59", "13
6", "38", "57", "69"))

data_Figure1A %>%
  ggplot(aes(x = genotype, y = Pi, fill = interaction(genotype, treatment))) +
  geom_boxplot(position = position_dodge(0.9), outlier.shape = NA, lwd = 0.5)  +
  theme_classic() +
  xlab("Genotype") +
  ylab("Pi (µmol/g)") +
  theme(axis.title.x = element_text(size = 14, face = "bold"),
        axis.title.y = element_text(size = 14, face = "bold"),
        axis.text.y = element_text(size = 12),
        axis.text.x = element_text(size = 12)) +
  geom_hline(yintercept = mean(data$Pi), linetype = 2) +
  geom_point(position = position_jitterdodge(dodge.width = 0.9), size = 1) +
  stat_compare_means(aes(label = ..p.signif..),
                     method = "kruskal.test",
                     size = 4, angle = 45,
                     hjust = 0.5, vjust = 0.1, label.y = 55) +
  scale_fill_manual(values = c("57.K" = "#ffc4a6",
                               "69.K" = "#ffc4a6",
                               "38.K" = "#ffc4a6",
                               "53.K" = "#e4f1fc",
                               "136.K" = "#e4f1fc",
                               "59.K" = "#e4f1fc",
                               "57.T" = "#fd582f",
                               "69.T" = "#fd582f",
                               "38.T" = "#fd582f",
                               "53.T" = "#82b9e9",
                               "136.T" = "#82b9e9",
                               "59.T" = "#82b9e9")) -> boxplot

boxplot
```

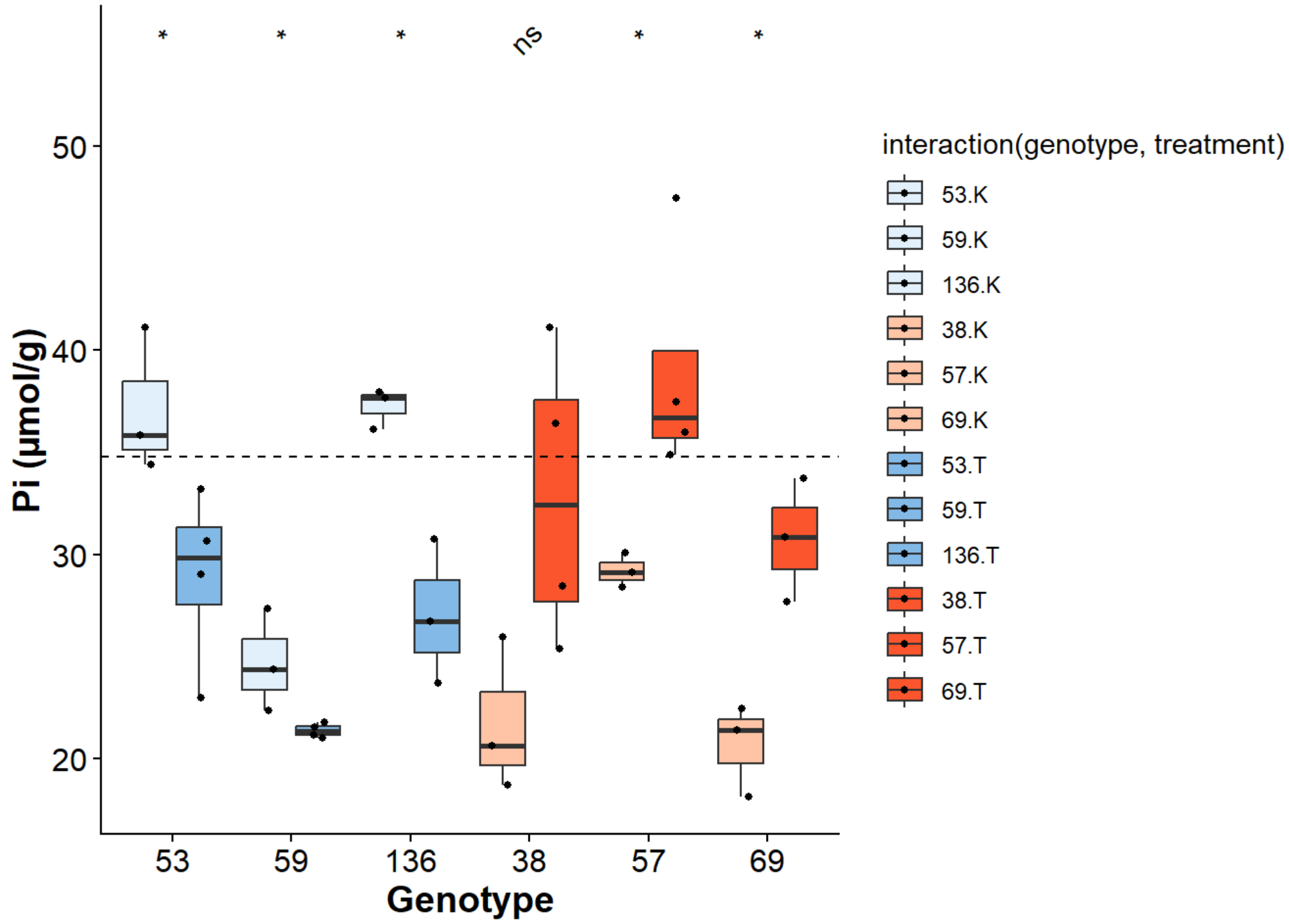

6 - Figure 1A right

```
library(ggplot2)

# Order the dataframe by the descending value of ratio_Pi
data_ratio_median <- data_ratio_median[order(data_ratio_median$ratio_Pi), ]

# Create a column for color based on interval_Pi
data_ratio_median$color <- ifelse(data_ratio_median$interval_Pi == "negative", "#82b9
e9",
                                  ifelse(data_ratio_median$interval_Pi == "positive",
"#e94e23",  # Burgundy Red
                                         "#d3d3d3"))  # Light Grey

# Transform the values of ratio_Pi: those less than 1 should be converted to negative
values relative to 1
data_ratio_median$transformed_ratio_Pi <- ifelse(data_ratio_median$ratio_Pi >= 1,
                                                 data_ratio_median$ratio_Pi - 1,
                                                 -(1 - data_ratio_median$ratio_Pi))

# Create the bar plot
ggplot(data_ratio_median, aes(x = reorder(genotype, ratio_Pi), y = transformed_ratio_
Pi, fill = color)) +
  geom_bar(stat = "identity") +
  geom_hline(yintercept = 0, linetype = "dashed", color = "black") +  # Horizontal li
ne at y = 0
  scale_fill_identity() +
  theme_minimal() +
  theme(panel.grid.major = element_line(color = "white"),  # Major grid lines in whit
e
        panel.grid.minor = element_line(color = "white"),  # Minor grid lines in whit
e
        axis.text.x = element_text(angle = 90, hjust = 1, size = 6),  # Reduce text s
ize
        panel.background = element_rect(fill = "white")) +  # White panel background
  xlab("Genotype") +
  ylab("Transformed Ratio Pi (vs 1)")
```

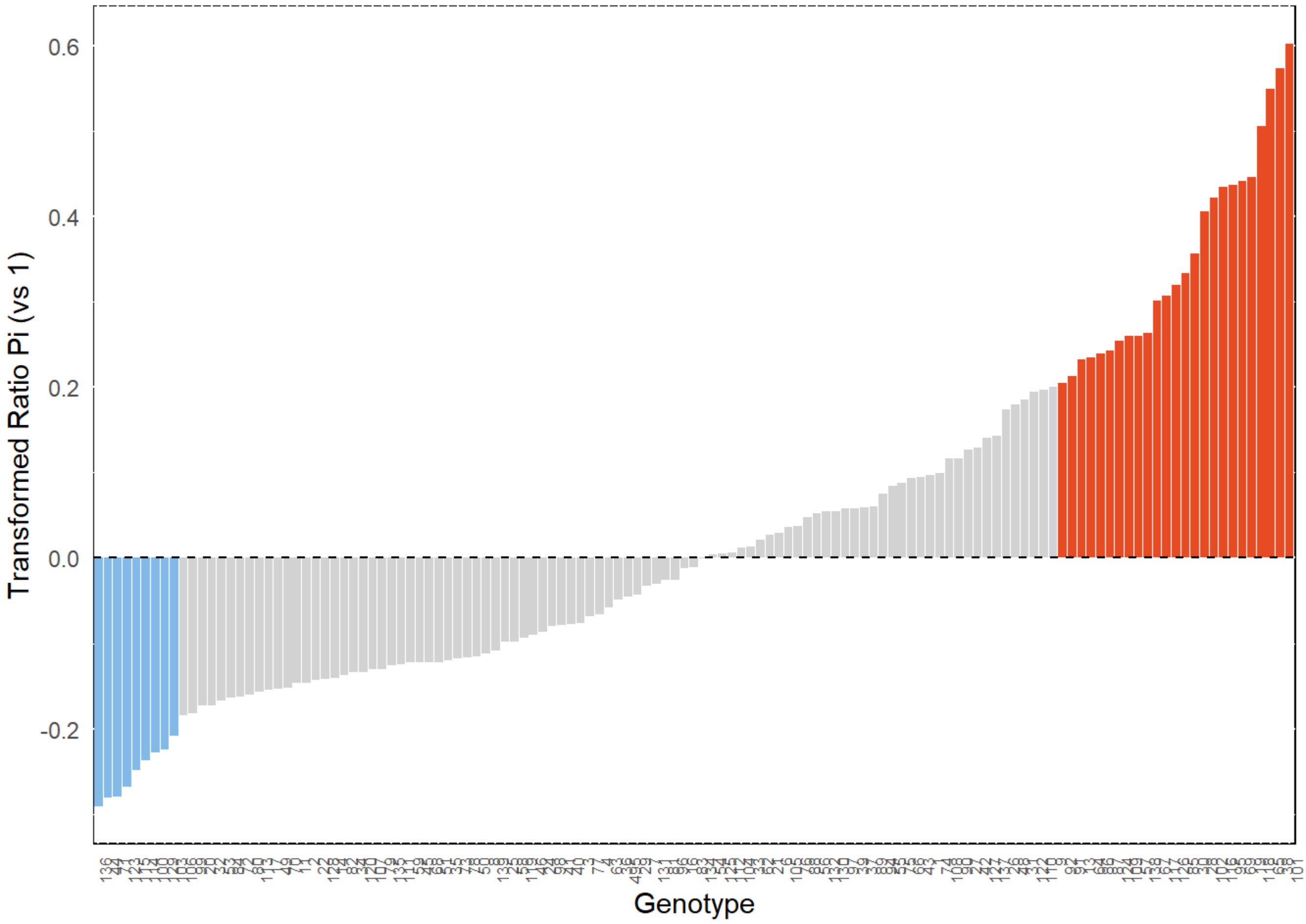

7 - Figure 1B left

```
#Figure 1

data_Figure1B<- data %>%
  filter(genotype %in% c("9", "11","74", "38", "57", "100"))

data_Figure1B$genotype <- factor(data_Figure1B$genotype, levels = c("9", "11","74",
"38", "57", "100"))

data_Figure1B %>%
  ggplot(aes(x = genotype, y = shoot, fill = interaction(genotype, treatment))) +
  geom_boxplot(position = position_dodge(0.9), outlier.shape = NA, lwd = 0.5)  +
  theme_classic() +
  xlab("Genotype") +
  ylab("Pi (µmol/g)") +
  theme(axis.title.x = element_text(size = 14, face = "bold"),
        axis.title.y = element_text(size = 14, face = "bold"),
        axis.text.y = element_text(size = 12),
        axis.text.x = element_text(size = 12)) +
  geom_hline(yintercept = mean(data$shoot), linetype = 2) +
  geom_point(position = position_jitterdodge(dodge.width = 0.9), size = 1) +
 stat_compare_means(aes(label = ..p.signif..),
                    method = "kruskal.test",
                    size = 4, angle = 45,
                    hjust = 0.5, vjust = 0.1, label.y = 55) +
  scale_fill_manual(values = c("9.K" = "#EEDDF9",
                               "11.K" = "#EEDDF9",
                               "74.K" = "#EEDDF9",
                               "38.K" = "#D1F7D0",
                               "57.K" = "#D1F7D0",
                               "100.K" = "#D1F7D0",
                               "9.T" = "#9c53e1",
                               "11.T" = "#9c53e1",
                               "74.T" = "#9c53e1",
                               "38.T" = "#6ce05a",
                               "57.T" = "#6ce05a",
                               "100.T" = "#6ce05a")) -> boxplot

boxplot
```

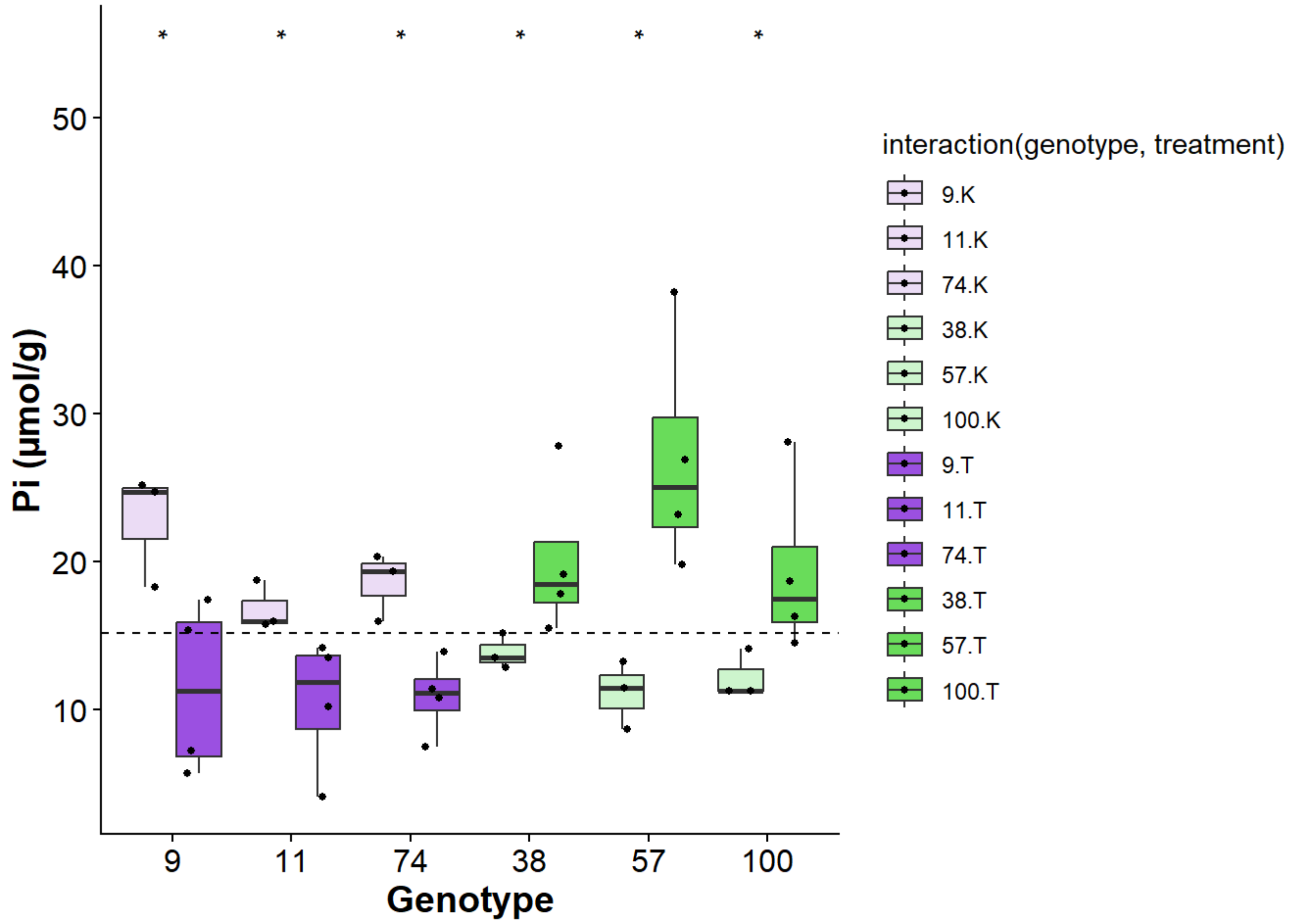

8 - Figure 1B right

```
# Order the dataframe by the descending value of ratio_shoot
data_ratio_median <- data_ratio_median[order(data_ratio_median$ratio_shoot), ]

# Create a column for color based on interval_shoot
data_ratio_median$color <- ifelse(data_ratio_median$interval_shoot == "negative", "#9
c53e1",
                                  ifelse(data_ratio_median$interval_shoot == "positiv
e", "#6ce05a",  # Burgundy Red
                                         "#d3d3d3"))  # Light Grey

# Transform the values of ratio_shoot: those less than 1 should be converted to negat
ive values relative to 1
data_ratio_median$transformed_ratio_shoot <- ifelse(data_ratio_median$ratio_shoot >=
1,
                                                 data_ratio_median$ratio_shoot - 1,
                                                 -(1 - data_ratio_median$ratio_shoo
t))

# Create the bar plot
ggplot(data_ratio_median, aes(x = reorder(genotype, ratio_shoot), y = transformed_rat
io_shoot, fill = color)) +
  geom_bar(stat = "identity") +
  geom_hline(yintercept = 0, linetype = "dashed", color = "black") +  # Horizontal li
ne at y = 0
  scale_fill_identity() +
  theme_minimal() +
  theme(panel.grid.major = element_line(color = "white"),  # Major grid lines in whit
e
        panel.grid.minor = element_line(color = "white"),  # Minor grid lines in whit
e
        axis.text.x = element_text(angle = 90, hjust = 1, size = 6),  # Reduce text s
ize
        panel.background = element_rect(fill = "white")) +  # White panel background
  xlab("Genotype") +
  ylab("Transformed Ratio Shoot (vs 1)")
```

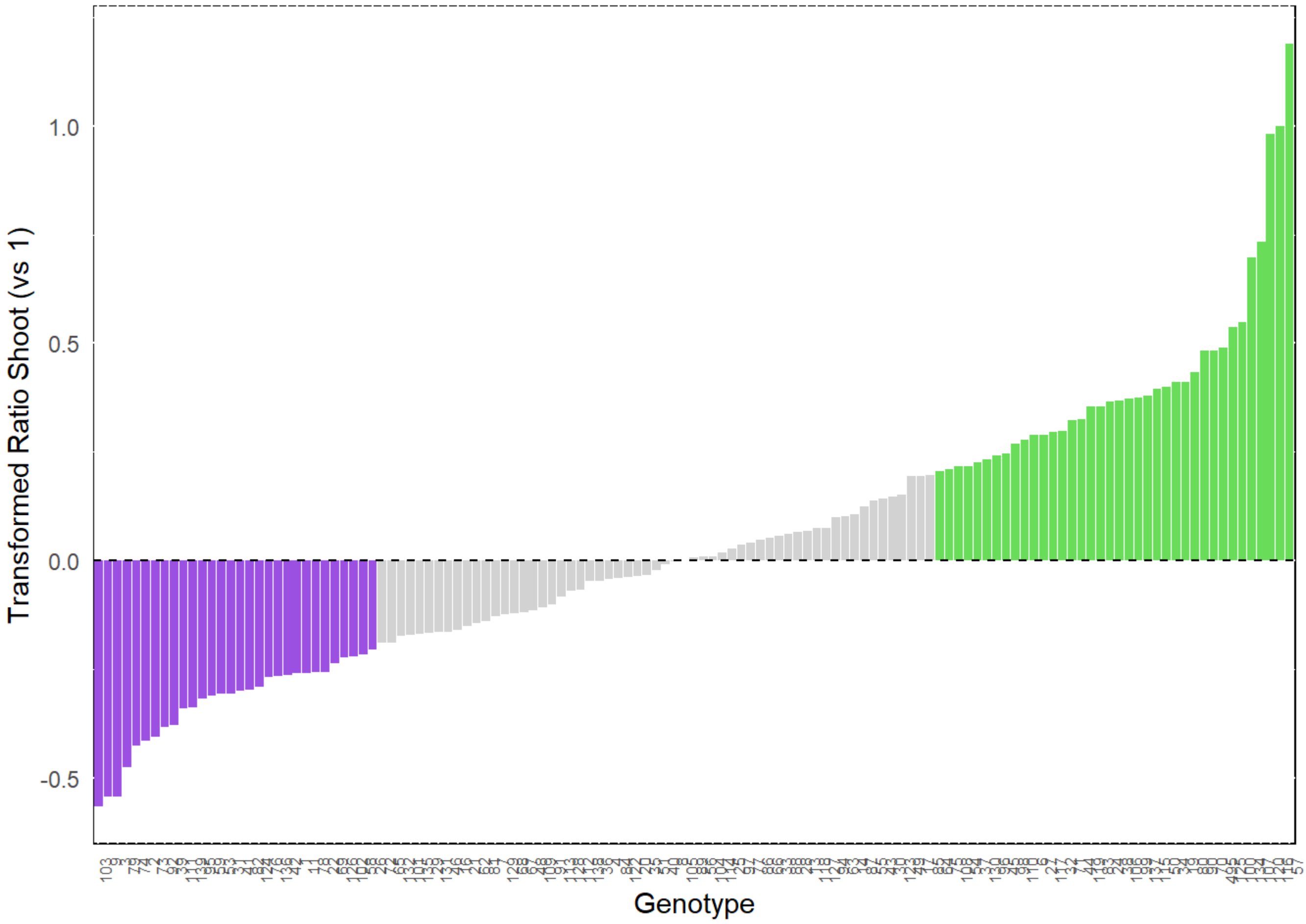

9 - Fugure 1C - Sankey plot

```
library(ggsankey)
library(ggplot2)

# Select relevant columns from the dataset
data_Sankey <- data_response[c(1, 17:18)]

# Convert the data into long format for use with ggsankey
data_Sankey_long <- data_Sankey %>%
  make_long(interval_Pi, interval_shoot)

# Display the transformed data
print(data_Sankey_long)
```

```
## # A tibble: 254 × 4
##    x              node           next_x         next_node
##    <fct>          <chr>          <fct>          <chr>
##  1 interval_Pi    non responding interval_shoot negative
##  2 interval_shoot negative       <NA>           <NA>
##  3 interval_Pi    negative       interval_shoot positive
##  4 interval_shoot positive       <NA>           <NA>
##  5 interval_Pi    positive       interval_shoot non responding
##  6 interval_shoot non responding <NA>           <NA>
##  7 interval_Pi    positive       interval_shoot negative
##  8 interval_shoot negative       <NA>           <NA>
##  9 interval_Pi    negative       interval_shoot negative
## 10 interval_shoot negative       <NA>           <NA>
## # ℹ 244 more rows
```

```
# Modify the data to include appropriate categories for visualization
data_Sankey_long <- data_Sankey_long %>%
  mutate(
    response_category = case_when(
      node == "non responding" ~ "non responding",  # Grey color for non-responding c
ategory
      node == "positive" & x == "interval_shoot" ~ "positive_shoot",  # Specific colo
r for positive in shoot interval
      node == "negative" & x == "interval_shoot" ~ "negative_shoot",  # Specific colo
r for negative in shoot interval
      node == "positive" ~ "positive",   # Default color for positive in Pi interval
      node == "negative" ~ "negative",   # Default color for negative in Pi interval
      TRUE ~ "other"  # Category for unspecified nodes
    )
  )

# Create the Sankey diagram using ggplot2
pl <- ggplot(data_Sankey_long, aes(
  x = x,
  next_x = next_x,
  node = node,
  next_node = next_node,
  fill = factor(response_category)  # Assign colors based on the defined categories
))

# Add the Sankey diagram layer
pl <- pl + geom_sankey(
  flow.alpha = 0.5,          # Set transparency of flow lines
  node.color = "black",      # Set the color for the node borders
  show.legend = TRUE         # Display the legend for the categories
)

# Define the color scheme for each response category
pl <- pl + scale_fill_manual(values = c(
  "non responding" = "#d3d3d3",  # Grey for "non responding"
  "positive_shoot" = "#6ce05a",  # Green for positive in shoot interval
  "negative_shoot" = "#9c53e1",  # Purple for negative in shoot interval
  "negative" = "#82b9e9",        # Blue for negative in Pi interval
  "positive" = "#e94e23",        # Red for positive in Pi interval
  "other" = "#cccccc"            # Light grey for unspecified nodes
)) +
  theme_bw() +  # Use a white background theme
  theme(
    panel.grid.major = element_blank(),  # Remove major grid lines
    panel.grid.minor = element_blank()   # Remove minor grid lines
  )

# Display the Sankey plot
print(pl)
```

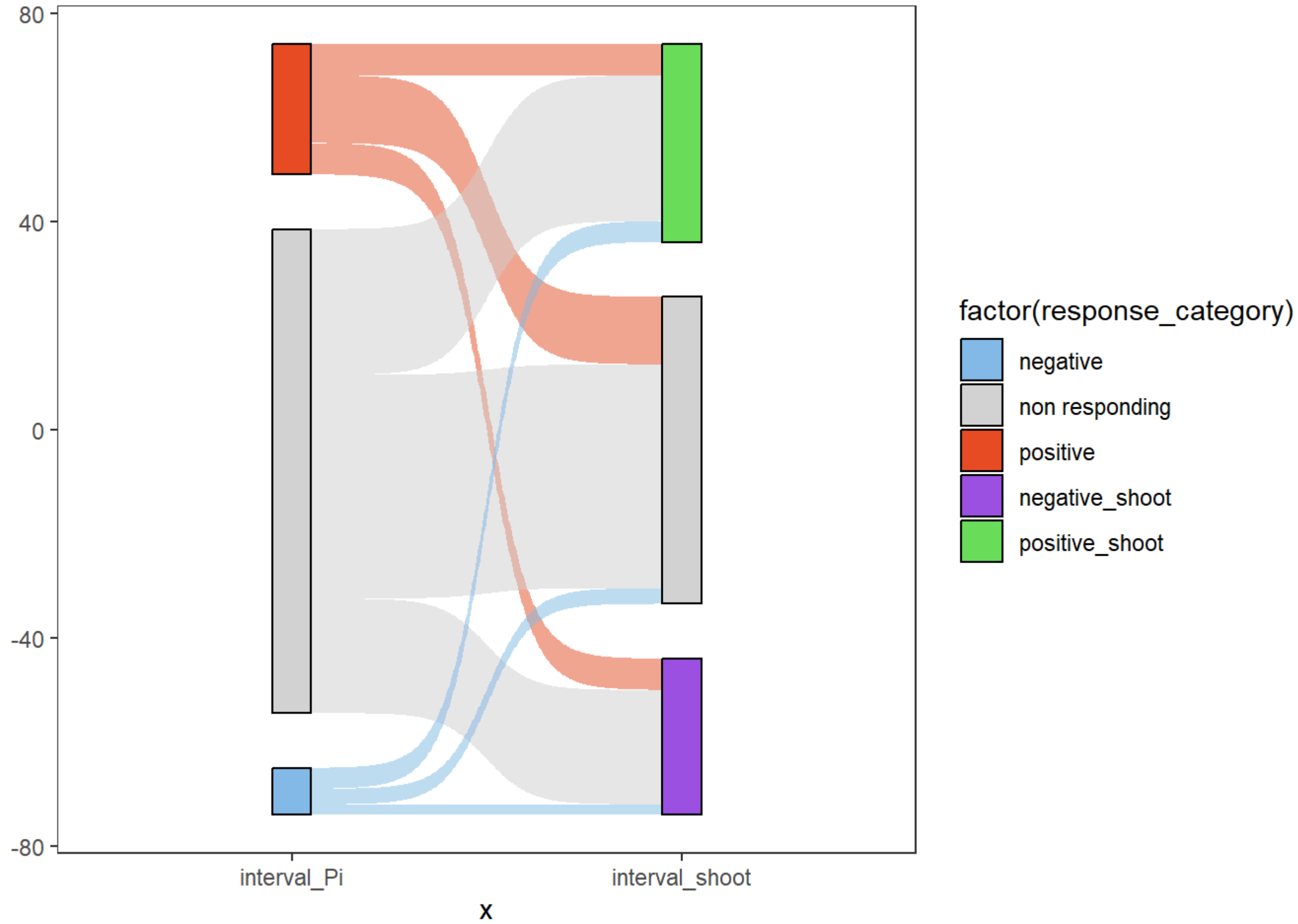

10 - Figure 2A - Heatmap

```
# Load necessary libraries
library(devtools)
```

```
## Caricamento del pacchetto richiesto: usethis
```

```
library(phytools)
```

```
## Caricamento del pacchetto richiesto: ape
```

```
##
## Caricamento pacchetto: 'ape'
```

```
## Il seguente oggetto è mascherato da 'package:ggpubr':
##
##     rotate
```

```
## Il seguente oggetto è mascherato da 'package:dplyr':
##
##     where
```

```
## Caricamento del pacchetto richiesto: maps
```

```
##
## Caricamento pacchetto: 'maps'
```

```
## Il seguente oggetto è mascherato da 'package:purrr':
##
##     map
```

```
library(RColorBrewer)
library(pheatmap)
library(grid)

# Transform the dataset into a matrix
data_ratio <- as.data.frame(data_ratio)
rownames(data_ratio) <- data_ratio[, 1]
data_ratio[, 1] <- NULL
data_ratio <- as.matrix(data_ratio)

# Import the annotation file (ensure the path is correct)
#use a file with a column for sample, another for varieties
data_variety <- read.csv("C:/Users/Alber/Desktop/DOTTORATO/LATTUGA/LANDLAB/P/data _va
rierty.csv",  #change with your path
                 header=T,sep = ";", na.string=c("na", "NA", "null"))

# Create a data frame for variety annotations
var_df <- data.frame("Var" = data_variety$variety)
rownames(var_df) <- rownames(data_ratio)  # Ensure annotations match rownames in data

# Define a color palette for the heatmap annotations
ann_colors <- list(
  Time = c("white", "firebrick"),
  Time2 = c("white", "firebrick"),
  CellType = c(CT1 = "#1B9E77", CT2 = "#D95F02"),
  GeneClass = c(Path1 = "#7570B3", Path2 = "#E7298A", Path3 = "#66A61E")
)

# Remove outliers from a specific column (column 84 in this case)
data_ratio <- t(data_ratio)  # Transpose the matrix so columns become rows for proces
sing

# Select the column to check for outliers
column_data <- data_ratio[, 84]

# Calculate the first and third quartiles and the Interquartile Range (IQR)
Q1 <- quantile(column_data, 0.25)
Q3 <- quantile(column_data, 0.75)
IQR <- Q3 - Q1

# Define the limits for non-outlier values
lower_limit <- Q1 - 1.5 * IQR
upper_limit <- Q3 + 1.5 * IQR

# Replace outlier values with NA
data_ratio[column_data < lower_limit | column_data > upper_limit, 84] <- NA

# Scale the dataset (z-score normalization)
data_scaled <- scale(data_ratio)

# Create the heatmap with the scaled dataset
pheatmap(t(data_scaled),
         annotation_colors = ann_colors,  # Apply color palette for annotations
         show_rownames = TRUE,  # Display row names (genotypes)
         show_colnames = TRUE,  # Display column names (phenotypes)
```

```
        border_color = FALSE,  # Remove borders around cells
        fontsize_col = 7,  # Set font size for column labels
        fontsize_row = 3,  # Set font size for row labels
        angle_col = 45,  # Rotate column labels for better readability
        cutree_cols = 2,  # Cluster columns into two groups
        cutree_rows = 3,  # Cluster rows into three groups
        annotation_row = var_df,  # Add row annotations based on variety
        main = "Phenotypic Variability Across Genotypes")  # Title of the heatmap
```

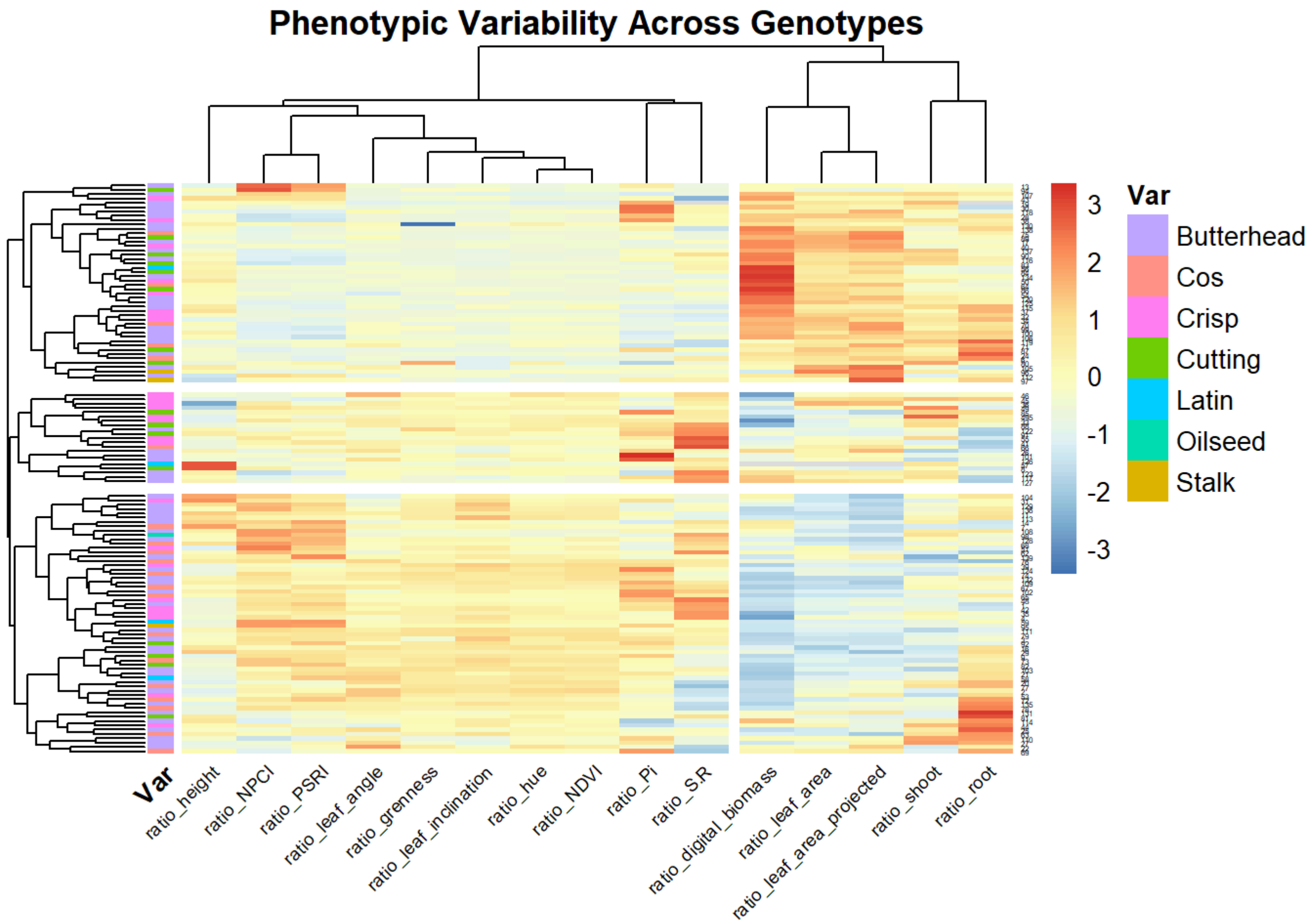

11 - Broad sense heritability

```
#1- Create a table to treat Ctrl
filtered_data <- data[data$treatment == "K", ]

# Create a new table containing only the columns genotype and Pi
Kdata <- filtered_data[, c("genotype", "Pi", "shoot", "S.R", "root", "Digital.biomas
s..mm..", "Height..mm.", "hue.average....", "Leaf.angle....", "Leaf.area..mm..", "Lea
f.inclination..mm..mm..", "NDVI.average", "NPCI.average", "PSRI.average", "greenness.
average")]

# Write the new table into a .txt file
write.table(Kdata, "Kdata.txt", sep = "\t", row.names = FALSE, quote = FALSE)

#2- Create column for the T and ratio T/K
data_K <- data %>% filter(treatment == "K")
data_T <- data %>% filter(treatment == "T")

mediane_K <- data_K %>%
  group_by(genotype) %>%
  summarise(
    mediana_Pi = median(Pi, na.rm = TRUE),
    mediana_shoot = median(shoot, na.rm = TRUE),
    mediana_S.R = median(S.R, na.rm = TRUE),
    mediana_root = median(root, na.rm = TRUE),
    mediana_Digital.biomass..mm.. = median(Digital.biomass..mm.., na.rm = TRUE),
    mediana_Height..mm. = median(Height..mm., na.rm = TRUE),
    mediana_hue.average.... = median(hue.average...., na.rm = TRUE),
    mediana_Leaf.angle.... = median(Leaf.angle...., na.rm = TRUE),
    mediana_Leaf.area..mm.. = median(Leaf.area..mm.., na.rm = TRUE),
    mediana_Leaf.inclination..mm..mm.. = median(Leaf.inclination..mm..mm.., na.rm = T
RUE),
    mediana_NDVI.average = median(NDVI.average, na.rm = TRUE),
    mediana_NPCI.average = median(NPCI.average, na.rm = TRUE),
    mediana_PSRI.average = median(PSRI.average, na.rm = TRUE),
    mediana_greenness.average = median(greenness.average, na.rm = TRUE)
  )

# Merge the medians with the treatment data (T)
data_T <- data_T %>%
  left_join(mediane_K, by = "genotype")

# Calculate the T/K ratio for all variables
data_T <- data_T %>%
  mutate(
    Pi_ratio = Pi / mediana_Pi,
    Shoot_ratio = shoot / mediana_shoot,
    S.R_ratio = S.R / mediana_S.R,
    root_ratio = root / mediana_root,
    Digital.biomass_ratio = Digital.biomass..mm.. / mediana_Digital.biomass..mm..,
    Height_ratio = Height..mm. / mediana_Height..mm.,
    hue_ratio = hue.average.... / mediana_hue.average....,
    Leaf.angle_ratio = Leaf.angle.... / mediana_Leaf.angle....,
    Leaf.area_ratio = Leaf.area..mm.. / mediana_Leaf.area..mm..,
    Leaf.inclination_ratio = Leaf.inclination..mm..mm.. / mediana_Leaf.inclination..m
m..mm..,
    NDVI_ratio = NDVI.average / mediana_NDVI.average,
```

```
    NPCI_ratio = NPCI.average / mediana_NPCI.average,
    PSRI_ratio = PSRI.average / mediana_PSRI.average,
    greenness_ratio = greenness.average / mediana_greenness.average
  )

# Select the columns of interest
new_table <- data_T %>%
  select(
    genotype, Pi, Pi_ratio, shoot, Shoot_ratio, S.R, S.R_ratio, root, root_ratio,
    Digital.biomass..mm.., Digital.biomass_ratio, Height..mm., Height_ratio,
    hue.average...., hue_ratio, Leaf.angle...., Leaf.angle_ratio,
    Leaf.area..mm.., Leaf.area_ratio, Leaf.inclination..mm..mm.., Leaf.inclination_ra
tio,
    NDVI.average, NDVI_ratio,
    NPCI.average, NPCI_ratio, PSRI.average, PSRI_ratio, greenness.average, greenness_
ratio
  )

# Write the new table into a text file
write.table(new_table, "heritability_ratios_T_extended.txt", sep = "\t", row.names =
FALSE, quote = FALSE)

# Specify genotype and range of phenotype column from phenotype file
phenofile = '/Users/Alber/Desktop/DOTTORATO/LATTUGA/LANDLAB/P/Kdata.txt'
col_sep = "\t"
gwas_id_col = 1
start_phenotype_col = 2
end_phenotype_col = 29

##### Function starts, don't modify this block
broad_sense <- function(idcol, phencol) {
  trait <- as.matrix(na.omit(new[, c(idcol, phencol)]))
  ### Phenotype with reps
  Y <- as.matrix(trait[, 2])
  rownames(Y) <- trait[, 1]
  ## Build the trait mean
  trait_mean <- aggregate(trait[, 2], list(trait[, 1]), mean)
  Y_mean <- as.matrix(trait_mean[, 2])
  rownames(Y_mean) <- trait_mean[, 1]

  ## BROAD SENSE H
  data_H <- data.frame('ecot_id' = as.factor(rownames(Y)), value = Y[, 1])

  anv <- anova(lm(value ~ ecot_id, data = data_H))

  mean_nb_rep <- nrow(Y) / nrow(Y_mean)    # There are some assumptions in ANOVA abou
t having the same number of reps, but lm should be robust
  theta_aov <- c((anv$'Mean Sq'[1] - anv$'Mean Sq'[2]) / mean_nb_rep, anv$'Mean S
q'[2])
  H_aov <- theta_aov[1] / (theta_aov[1] + theta_aov[2])
  print(paste(((colnames(new))[phencol]), "Broad sense Heritability:", H_aov, sep = "
"))
}

####### Function ends, execute broad sense here
e <- simpleError("test error")
```

```
new <- read.table(phenofile, header = TRUE, sep = col_sep, na.strings = 'NA')
new <- na.omit(new)

for (col in start_phenotype_col:end_phenotype_col) {
  withRestarts(tryCatch(broad_sense(gwas_id_col, col), error = function(e) e))
}
```

```
## [1] "Pi Broad sense Heritability: 0.327389814124397"
## [1] "shoot Broad sense Heritability: 0.190484091052698"
## [1] "S.R Broad sense Heritability: 0.568169973741243"
## [1] "root Broad sense Heritability: 0.499828672416008"
## [1] "Digital.biomass..mm.. Broad sense Heritability: 0.600137322282273"
## [1] "Height..mm. Broad sense Heritability: 0.752711008109179"
## [1] "hue.average.... Broad sense Heritability: 0.693700919085143"
## [1] "Leaf.angle.... Broad sense Heritability: 0.398559787874543"
## [1] "Leaf.area..mm.. Broad sense Heritability: 0.319820975441951"
## [1] "Leaf.inclination..mm..mm.. Broad sense Heritability: 0.588200408259822"
## [1] "NDVI.average Broad sense Heritability: 0.750011864592086"
## [1] "NPCI.average Broad sense Heritability: 0.615401328109686"
## [1] "PSRI.average Broad sense Heritability: 0.896188191796785"
## [1] "greenness.average Broad sense Heritability: 0.831972395387213"
```

```
# K
"Pi Broad sense Heritability: 0.327389814124397"
```

```
## [1] "Pi Broad sense Heritability: 0.327389814124397"
```

```
"shoot Broad sense Heritability: 0.190484091052698"
```

```
## [1] "shoot Broad sense Heritability: 0.190484091052698"
```

```
"S.R Broad sense Heritability: 0.568169973741243"
```

```
## [1] "S.R Broad sense Heritability: 0.568169973741243"
```

```
"root Broad sense Heritability: 0.499828672416008"
```

```
## [1] "root Broad sense Heritability: 0.499828672416008"
```

```
"Digital.biomass..mm.. Broad sense Heritability: 0.600137322282273"
```

```
## [1] "Digital.biomass..mm.. Broad sense Heritability: 0.600137322282273"
```

```
"Height..mm. Broad sense Heritability: 0.752711008109179"
```

```
## [1] "Height..mm. Broad sense Heritability: 0.752711008109179"
```

```
"hue.average.... Broad sense Heritability: 0.693700919085143"
```

```
## [1] "hue.average.... Broad sense Heritability: 0.693700919085143"
```

```
"Leaf.angle.... Broad sense Heritability: 0.398559787874543"
```

```
## [1] "Leaf.angle.... Broad sense Heritability: 0.398559787874543"
```

```
"Leaf.area..mm.. Broad sense Heritability: 0.319820975441951"
```

```
## [1] "Leaf.area..mm.. Broad sense Heritability: 0.319820975441951"
```

```
"Leaf.inclination..mm..mm.. Broad sense Heritability: 0.588200408259822"
```

```
## [1] "Leaf.inclination..mm..mm.. Broad sense Heritability: 0.588200408259822"
```

```
"NDVI.average Broad sense Heritability: 0.750011864592086"
```

```
## [1] "NDVI.average Broad sense Heritability: 0.750011864592086"
```

```
"NPCI.average Broad sense Heritability: 0.615401328109686"
```

```
## [1] "NPCI.average Broad sense Heritability: 0.615401328109686"
```

```
"PSRI.average Broad sense Heritability: 0.896188191796785"
```

```
## [1] "PSRI.average Broad sense Heritability: 0.896188191796785"
```

```
"greenness.average Broad sense Heritability: 0.831972395387213"
```

```
## [1] "greenness.average Broad sense Heritability: 0.831972395387213"
```

```
# T
"Pi Broad sense Heritability: 0.494459100165354"
```

```
## [1] "Pi Broad sense Heritability: 0.494459100165354"
```

```
"shoot Broad sense Heritability: 0.246693923533641"
```

```
## [1] "shoot Broad sense Heritability: 0.246693923533641"
```

```
"S.R Broad sense Heritability: 0.608397762816039"
```

```
## [1] "S.R Broad sense Heritability: 0.608397762816039"
```

```
"root Broad sense Heritability: 0.526098691704259"
```

```
## [1] "root Broad sense Heritability: 0.526098691704259"
```

```
"Digital.biomass..mm.. Broad sense Heritability: 0.538792933135548"
```

```
## [1] "Digital.biomass..mm.. Broad sense Heritability: 0.538792933135548"
```

```
"Height..mm. Broad sense Heritability: 0.810553906922922"
```

```
## [1] "Height..mm. Broad sense Heritability: 0.810553906922922"
```

```
"hue.average.... Broad sense Heritability: 0.744828669987051"
```

```
## [1] "hue.average.... Broad sense Heritability: 0.744828669987051"
```

```
"Leaf.angle.... Broad sense Heritability: 0.443144994001118"
```

```
## [1] "Leaf.angle.... Broad sense Heritability: 0.443144994001118"
```

```
"Leaf.area..mm.. Broad sense Heritability: 0.333146429951933"
```

```
## [1] "Leaf.area..mm.. Broad sense Heritability: 0.333146429951933"
```

```
"Leaf.inclination..mm..mm.. Broad sense Heritability: 0.498436237419065"
```

```
## [1] "Leaf.inclination..mm..mm.. Broad sense Heritability: 0.498436237419065"
```

```
"NDVI.average Broad sense Heritability: 0.697066634495995"
```

```
## [1] "NDVI.average Broad sense Heritability: 0.697066634495995"
```

```
"NPCI.average Broad sense Heritability: 0.556917930455915"
```

```
## [1] "NPCI.average Broad sense Heritability: 0.556917930455915"
```

```
"PSRI.average Broad sense Heritability: 0.871763694903424"
```

```
## [1] "PSRI.average Broad sense Heritability: 0.871763694903424"
```

```
"greenness.average Broad sense Heritability: 0.878218662458944"
```

```
## [1] "greenness.average Broad sense Heritability: 0.878218662458944"
```

```
# Ratios
"Pi_ratio Broad sense Heritability: 0.329996460621562"
```

```
## [1] "Pi_ratio Broad sense Heritability: 0.329996460621562"
```

```
"Shoot_ratio Broad sense Heritability: 0.325619462105682"
```

```
## [1] "Shoot_ratio Broad sense Heritability: 0.325619462105682"
```

```
"S.R_ratio Broad sense Heritability: 0.458850802669031"
```

```
## [1] "S.R_ratio Broad sense Heritability: 0.458850802669031"
```

```
"root_ratio Broad sense Heritability: 0.306489907973609"
```

```
## [1] "root_ratio Broad sense Heritability: 0.306489907973609"
```

```
"Digital.biomass_ratio Broad sense Heritability: 0.227425193302999"
```

```
## [1] "Digital.biomass_ratio Broad sense Heritability: 0.227425193302999"
```

```
"Height_ratio Broad sense Heritability: 0.509705353876361"
```

```
## [1] "Height_ratio Broad sense Heritability: 0.509705353876361"
```

```
"hue_ratio Broad sense Heritability: 0.23994516827033"
```

```
## [1] "hue_ratio Broad sense Heritability: 0.23994516827033"
```

```
"Leaf.angle_ratio Broad sense Heritability: 0.329397409322106"
```

```
## [1] "Leaf.angle_ratio Broad sense Heritability: 0.329397409322106"
```

```
"Leaf.area_ratio Broad sense Heritability: 0.307188819927735"
```

```
## [1] "Leaf.area_ratio Broad sense Heritability: 0.307188819927735"
```

```
"Leaf.inclination_ratio Broad sense Heritability: 0.237742002757892"
```

```
## [1] "Leaf.inclination_ratio Broad sense Heritability: 0.237742002757892"
```

```
"NDVI_ratio Broad sense Heritability: 0.122405935667105"
```

```
## [1] "NDVI_ratio Broad sense Heritability: 0.122405935667105"
```

```
"NPCI_ratio Broad sense Heritability: 0.455476193109567"
```

```
## [1] "NPCI_ratio Broad sense Heritability: 0.455476193109567"
```

```
"PSRI_ratio Broad sense Heritability: 0.404446728512302"
```

```
## [1] "PSRI_ratio Broad sense Heritability: 0.404446728512302"
```

```
"greenness_ratio Broad sense Heritability: -0.242277953957643"
```

```
## [1] "greenness_ratio Broad sense Heritability: -0.242277953957643"
```

11.1 - Figure 3A

```
# Carica i pacchetti necessari
library(ggplot2)
library(reshape2)
```

```
##
## Caricamento pacchetto: 'reshape2'
```

```
## Il seguente oggetto è mascherato da 'package:tidyr':
##
##     smiths
```

```
library(dplyr)

# Creare il dataframe con i dati forniti
heritability_data <- data.frame(
  Variable = c("Pi", "Shoot biomass", "Shoot/Root", "Root biomass", "Digital biomas
s", "Height",
               "Hue average", "Leaf angle", "Leaf area", "Leaf inclination", "NDVI",
               "NPCI", "PSRI", "Greenness"),
  K = c(0.327389814124397, 0.190484091052698, 0.568169973741243, 0.499828672416008,
        0.600137322282273, 0.752711008109179, 0.693700919085143, 0.398559787874543,
        0.319820975441951, 0.588200408259822, 0.750011864592086, 0.615401328109686,
        0.896188191796785, 0.831972395387213),
  T = c(0.494459100165354, 0.246693923533641, 0.608397762816039, 0.526098691704259,
        0.538792933135548, 0.810553906922922, 0.744828669987051, 0.443144994001118,
        0.333146429951933, 0.498436237419065, 0.697066634495995, 0.556917930455915,
        0.871763694903424, 0.878218662458944),
  ratios = c(0.329996460621562, 0.325619462105682, 0.458850802669031, 0.3064899079736
09,
             0.227425193302999, 0.509705353876361, 0.23994516827033, 0.32939740932210
6,
             0.307188819927735, 0.237742002757892, 0.122405935667105, 0.4554761931095
67,
             0.404446728512302, 0)  # Valore per greenness_ratio considerato 0
)

# Trasforma i dati in formato lungo per ggplot
heritability_melted <- melt(heritability_data, id.vars = "Variable", variable.name =
"Group", value.name = "Heritability")

# Ottieni l'ordinamento dei valori di ratios dalla più alta alla più bassa
ratios_order <- heritability_melted %>%
  filter(Group == "ratios") %>%
  arrange(Heritability) %>%
  pull(Variable)

# Modifica l'ordine delle righe in base ai valori di ratios
heritability_melted$Variable <- factor(heritability_melted$Variable, levels = ratios_
order)

# Crea il grafico
ggplot(heritability_melted, aes(x = Group, y = Variable, fill = Heritability)) +
  geom_tile(color = "white") +
  scale_fill_gradient(low = "white", high = "purple", na.value = "grey") +
  theme_minimal() +
  theme(axis.text.x = element_text(angle = 0, hjust = 1),
        axis.text.y = element_text(size = 10)) +
  labs(title = "Broad Sense Heritability", x = "Group", y = "Variable", fill = "Herit
ability")
```

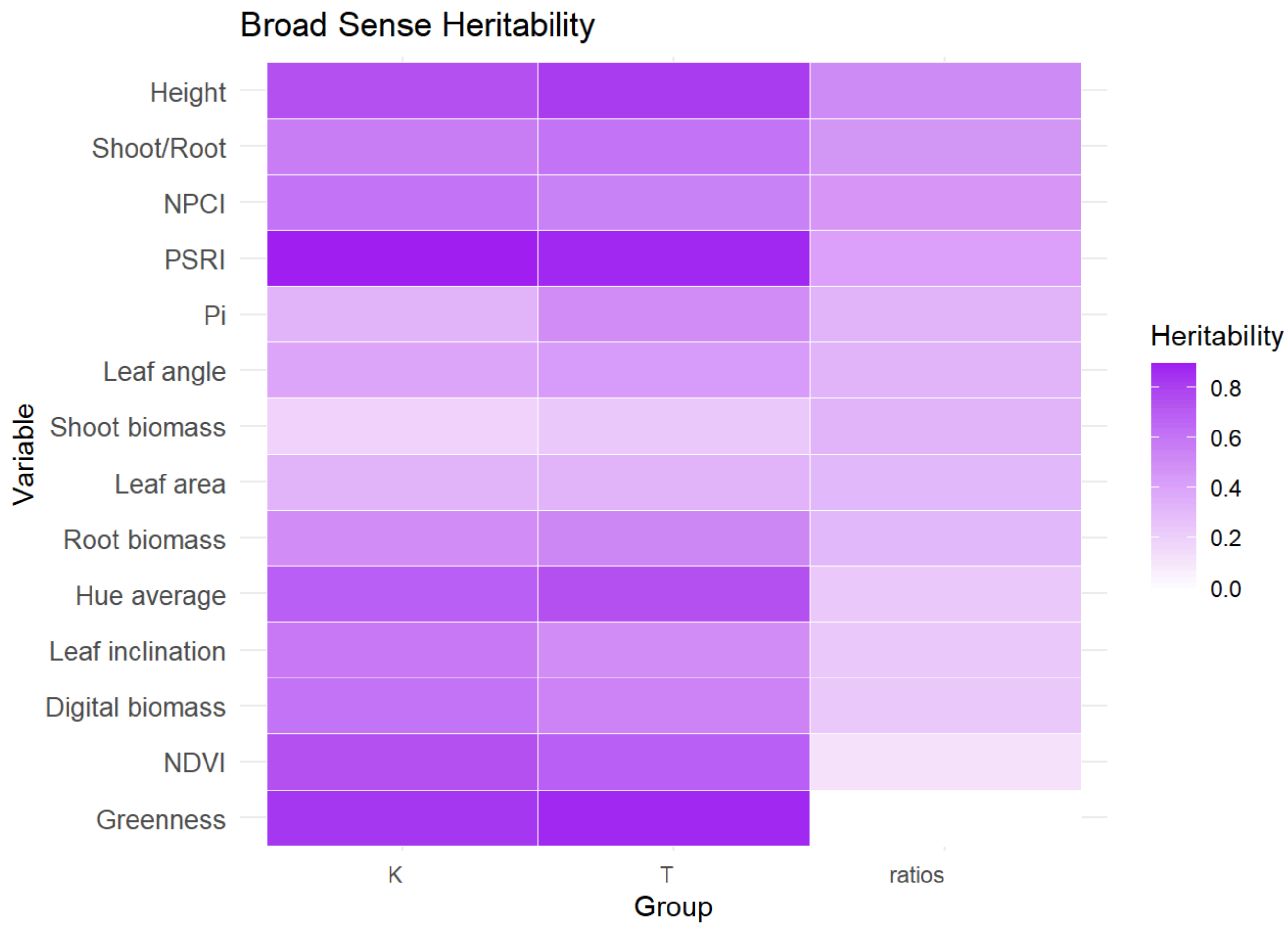

12 - Figure 4 - PCA

```
# Load the required libraries
library(devtools)
library(ggbiplot)
library(FactoMineR)
library(psych)
```

```
##
## Caricamento pacchetto: 'psych'
```

```
## Il seguente oggetto è mascherato da 'package:ggbiplot':
##
##     reflect
```

```
## Il seguente oggetto è mascherato da 'package:phytools':
##
##     rescale
```

```
## I seguenti oggetti sono mascherati da 'package:ggplot2':
##
##     %+%, alpha
```

```
library(factoextra)
```

```
## Welcome! Want to learn more? See two factoextra-related books at https://goo.gl/ve
3WBa
```

```
library(dplyr)
library(ggplot2)
library(ggfortify)
```

```
##
## Caricamento pacchetto: 'ggfortify'
```

```
## Il seguente oggetto è mascherato da 'package:ggbiplot':
##
##     ggbiplot
```

```
#data_response <- data_ratio_median %>% select(-c(6, 11:15))

# Convert genotype to a factor
data_response$genotype <- as.factor(data_response$genotype)

# Perform Principal Component Analysis (PCA) on the selected data columns, excluding
non-numeric variables
res.pca <- PCA(data_response[,-c(1,6,8:12,17:19)], graph = FALSE, scale = TRUE)
```

```
## Warning in PCA(data_response[, -c(1, 6, 8:12, 17:19)], graph = FALSE, scale =
## TRUE): Missing values are imputed by the mean of the variable: you should use
## the imputePCA function of the missMDA package
```

```
# Convert 'interval_response' to a factor for proper ordering
data_response$interval_response <- factor(data_response$interval_response,
                                           levels = c("++", "--", "+-", "-+", "-/",
"/-", "//", "+/", "/+"))

# Define a color palette for each 'interval_response' category
response_colors <- c("++" = "#00BFFF", "--" = "#FF1493", "+-" = "#00FF00",
                     "-+" = "#9400D3", "-/" = "#808080", "/-" = "#808080",
                     "//" = "#808080", "+/" = "#808080", "/+" = "#808080")

# Generate the PCA biplot colored by 'interval_response'
pca_biplot <- fviz_pca_biplot(res.pca,
                               axes = c(1, 2),
                               col.ind = data_response$interval_response,
                               palette = response_colors,
                               legend.title = "[Pi] and shoot biomass response",
                               col.var = "black",
                               geom = "text",
                               labelsize = 3,
                               title = "Plant Physiological Response")
```

```
## Warning: Using `size` aesthetic for lines was deprecated in ggplot2 3.4.0.
## ℹ Please use `linewidth` instead.
## ℹ The deprecated feature was likely used in the ggpubr package.
##   Please report the issue at <https://github.com/kassambara/ggpubr/issues>.
## This warning is displayed once every 8 hours.
## Call `lifecycle::last_lifecycle_warnings()` to see where this warning was
## generated.
```

```
# Display the PCA biplot
pca_biplot
```

```
## Ignoring unknown labels:
## • fill : "[Pi] and shoot biomass response"
## • linetype : "[Pi] and shoot biomass response"
## • shape : "[Pi] and shoot biomass response"
```

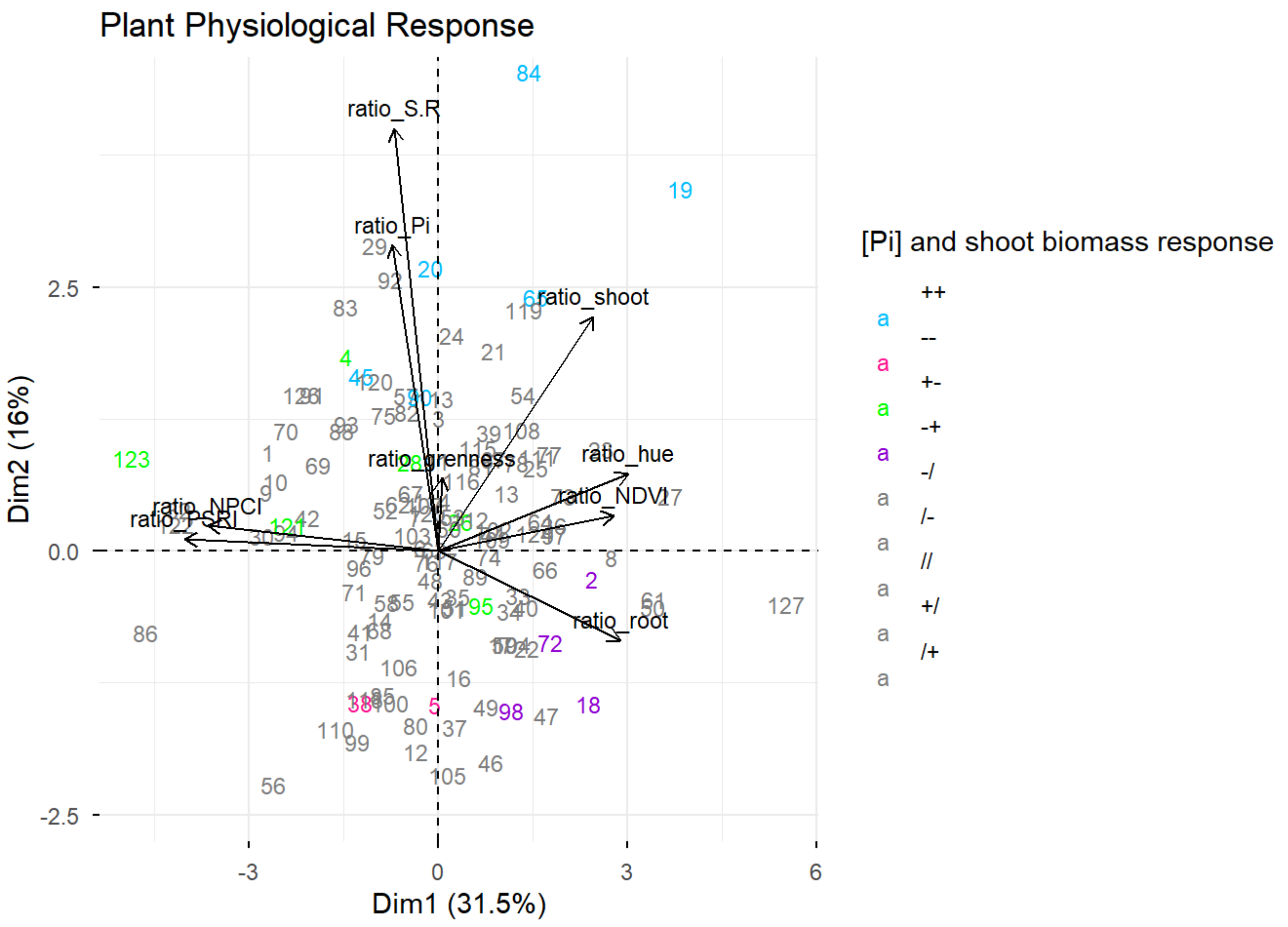

```
###################################### Color by selected genotypes - Figure 4 #######
#################################

# Function to assign genotypes to specific groups
assign_group <- function(genotype) {
  if (genotype %in% c("38", "57", "80", "85", "90")) {
    return("group1")
  } else if (genotype %in% c("13", "19", "66", "59", "94", "95")) {
    return("group2")
  } else if (genotype %in% c("11", "53", "72", "103")) {
    return("group3")
  } else if (genotype %in% c("24", "34", "71", "99", "100", "106")) {
    return("group4")
  } else {
    return("default")
  }
}

# Convert genotype to character type for consistency
data_response$genotype <- as.character(data_response$genotype)

# Apply the 'assign_group' function to assign genotypes to groups
data_response <- data_response %>%
  mutate(group = sapply(genotype, assign_group))

# Define a color palette for each genotype group
genotype_colors <- c(
  "group1" = "#00BFFF",   # Group 1
  "group2" = "#00FF00",   # Group 2
  "group3" = "#FF1493",   # Group 3
  "group4" = "#9400D3",   # Group 4
  "default" = "#D3D3D3"   # Default group in light gray
)

# Convert 'group' to a factor with a specific order
data_response$group <- factor(data_response$group, levels = c("group1", "group2", "gr
oup3", "group4", "default"))

# Perform PCA again after adjusting the dataset (excluding irrelevant columns)
res.pca <- PCA(data_response[,-c(1,6,8:12,17:20)], graph = FALSE, scale = TRUE)
```

```
## Warning in PCA(data_response[, -c(1, 6, 8:12, 17:20)], graph = FALSE, scale =
## TRUE): Missing values are imputed by the mean of the variable: you should use
## the imputePCA function of the missMDA package
```

```
# Extract PCA coordinates for the individuals (genotypes)
pca_data <- as.data.frame(res.pca$ind$coord)

# Add 'group' and 'genotype' columns to the PCA data for visualization
pca_data$group <- data_response$group
pca_data$genotype <- data_response$genotype

# Estrazione delle coordinate PCA per le variabili
var_data <- as.data.frame(res.pca$var$coord)
var_names <- rownames(res.pca$var$coord)  # Nomi delle variabili

# Estendere le frecce per posizionare le etichette all'apice
var_data_extended <- var_data %>%
  mutate(Dim.1_end = Dim.1 * 6,  # Estensione del 20% (puoi regolare il fattore)
         Dim.2_end = Dim.2 * 6)

# Create the PCA biplot with color-coded genotypes
pca_biplot <- fviz_pca_biplot(res.pca,
                               axes = c(1, 2),
                               geom.ind = "point",  # Only plot points for individual
s
                               col.var = "black",  # Color for variable arrows
                               geom.var = "arrow",
                               labelsize = 3,
                               title = "Plant Physiological Response by Genotype Grou
p") +
  # Points for the "default" group in gray with black border
  geom_point(data = pca_data %>% filter(group == "default"),
             aes(x = Dim.1, y = Dim.2),
             size = 4,  # Size of points
             fill = "#D3D3D3",  # Light gray fill color
             color = "black",  # Black border
             stroke = 0.5,  # Border thickness
             shape = 21) +  # Circle with border

  # Points for genotypes that are not "default" with their respective color
  geom_point(data = pca_data %>% filter(group != "default"),
             aes(x = Dim.1, y = Dim.2, fill = group),
             size = 4,
             color = "black",  # Black border
             stroke = 0.5,  # Border thickness
             shape = 21) +  # Circle with border

  # Add variable labels at the end of the arrows
  geom_text(data = var_data_extended,
            aes(x = Dim.1_end, y = Dim.2_end, label = var_names),
            size = 2.5, color = "black", fontface = "plain") +  # Label size and colo
r

  # Define the color palette for genotype groups
  scale_fill_manual(values = genotype_colors) +  # Fill points with genotype colors

  # Apply minimal theme and custom labels
  theme_minimal() +
  labs(fill = "Genotype Group")  # Legend for genotype groups
```

```
# Display the final PCA biplot
print(pca_biplot)
```

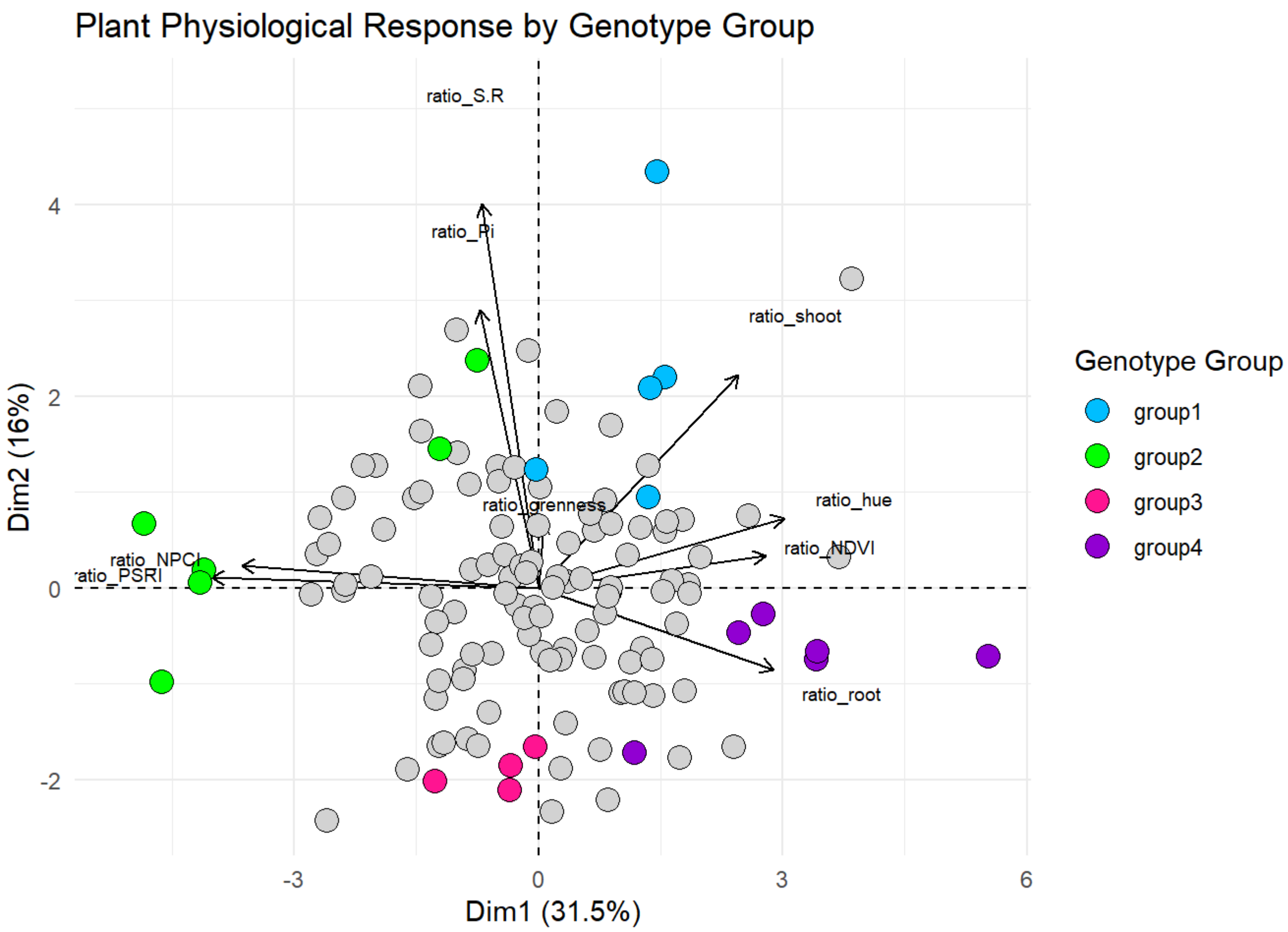

13 - FigureS3

```
library(corrplot)
```

```
## corrplot 0.95 loaded
```

```
#FigureS3A -  correlation matrix for traits in the entire dataset

#entire dataset
data_corr<- data[11:29]

#correlation matrix

cor_matrix <- cor(data_corr, use = "complete.obs")

default_colors <- colorRampPalette(c("#053061", "#2166AC", "#4393C3", "#92C5DE", "#D1
E5F0",
                                     "#FFFFFF", "#FDDBC7", "#F4A582", "#D6604D", "#B2
182B",
                                     "#67001F"))(200)

corrplot(cor_matrix, type = "upper", order = "hclust", col = default_colors,
         tl.col = "black", tl.srt = 45, diag = FALSE)
```

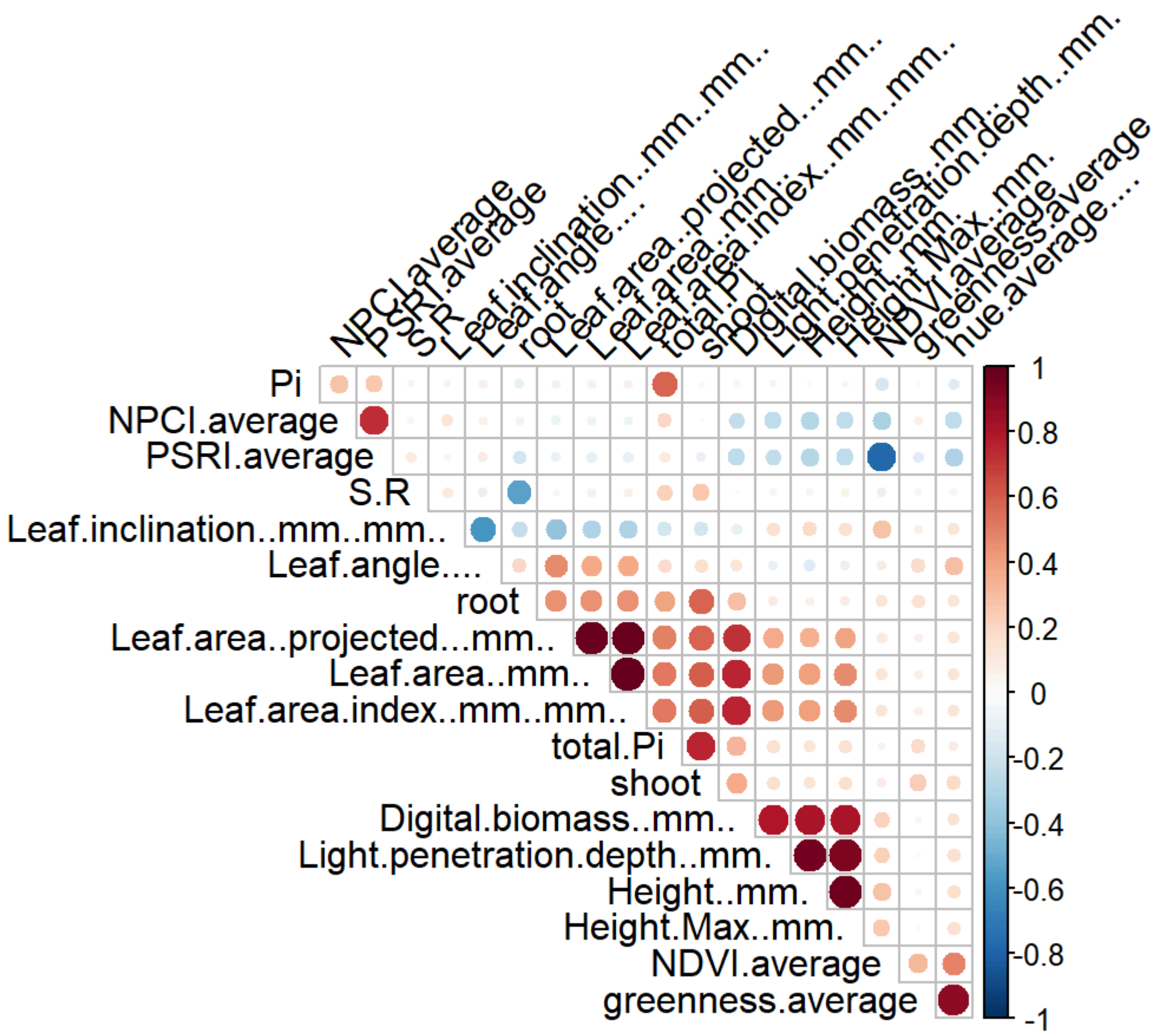


```
#FigureS3B- correlation matrix for traits in treated plants
data_T<- data %>%
  filter(treatment=="T")

data_T<- data_T[11:29]

#correlation matrix

cor_matrix <- cor(data_T, use = "complete.obs")

default_colors <- colorRampPalette(c("#053061", "#2166AC", "#4393C3", "#92C5DE", "#D1
E5F0",
                                     "#FFFFFF", "#FDDBC7", "#F4A582", "#D6604D", "#B2
182B",
                                     "#67001F"))(200)

corrplot(cor_matrix, type = "upper", order = "hclust", col = default_colors,
         tl.col = "black", tl.srt = 45, diag = FALSE)
```

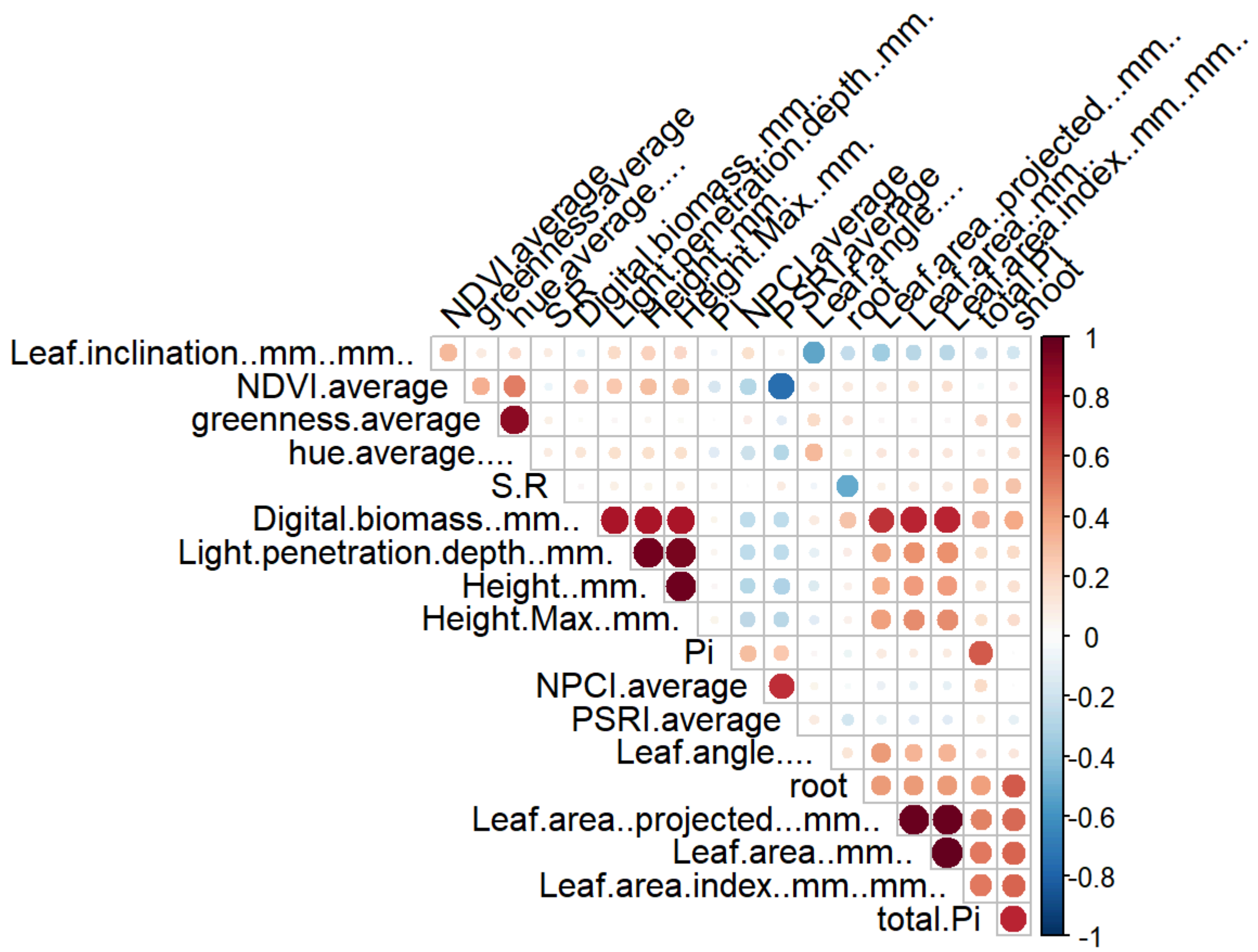


```
#FigureS3B - correlation matrix for traits in control plants
data_K<- data %>%
  filter(treatment=="K")

data_K<- data_K[11:29]

#entire dataset
data_corr<- data[11:29]

#correlation matrix

cor_matrix <- cor(data_K, use = "complete.obs")

default_colors <- colorRampPalette(c("#053061", "#2166AC", "#4393C3", "#92C5DE", "#D1
E5F0",
                                     "#FFFFFF", "#FDDBC7", "#F4A582", "#D6604D", "#B2
182B",
                                     "#67001F"))(200)

corrplot(cor_matrix, type = "lower", order = "hclust", col = default_colors,
         tl.col = "black", tl.srt = 45, diag = FALSE)
```

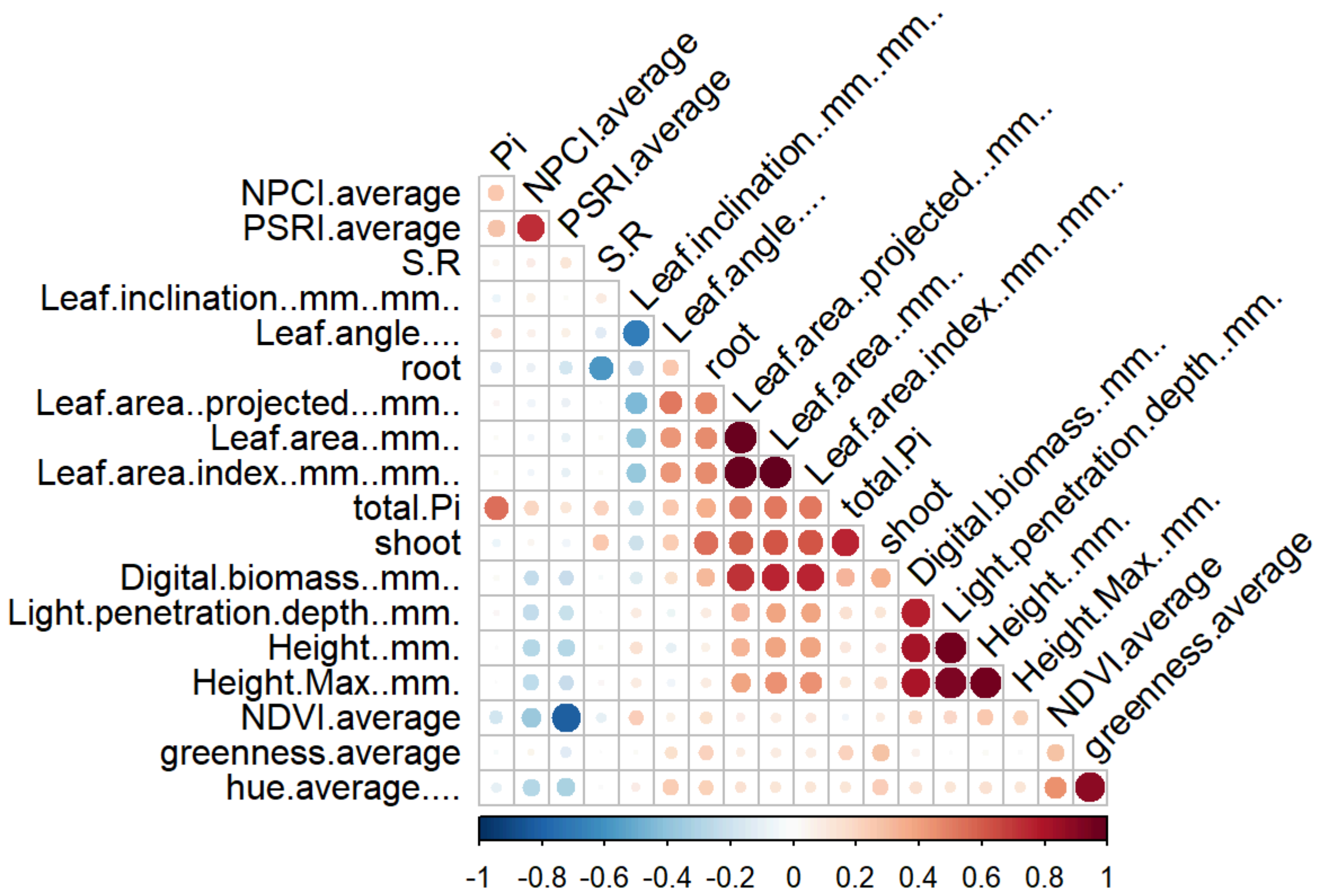

14 - Figure S5A

```
###install the following packages and/or load them

library(ggExtra)
library(tidyverse)
library(multcomp)
library(ggpubr)
library(rstatix)
library(patchwork)
library(dplyr)

###your path
#use Supplementary_table_9.csv
data_metabolites<- read.csv("C:/Users/Alber/Desktop/DOTTORATO/LATTUGA/LANDLAB/METABOL
ITES/metaboliti_landlab/metabolites.csv",
                 header=T,sep = ";", na.string=c("na", "NA", "null"))


data_metabolites$position <- as.factor(data_metabolites$position)
data_metabolites$genotype <- as.factor(data_metabolites$genotype)
data_metabolites$treatment <- as.factor(data_metabolites$treatment)
data_metabolites$variety <- as.factor(data_metabolites$variety)
data_metabolites$S.R <- as.numeric(data_metabolites$S.R)

#Figure S5 A

library(corrplot)

mydata_metabolites <- data_metabolites[, c(6:length(data_metabolites))]

mydata_metabolites <- na.omit(mydata_metabolites)

cor_matrix <- cor(mydata_metabolites, use = "complete.obs")

default_colors <- colorRampPalette(c("#053061", "#2166AC", "#4393C3", "#92C5DE", "#D1
E5F0",
                                     "#FFFFFF", "#FDDBC7", "#F4A582", "#D6604D", "#B2
182B",
                                     "#67001F"))(200)

corrplot(cor_matrix, type = "upper", order = "hclust", col = default_colors,
         tl.col = "black", tl.srt = 45, diag = FALSE)
```

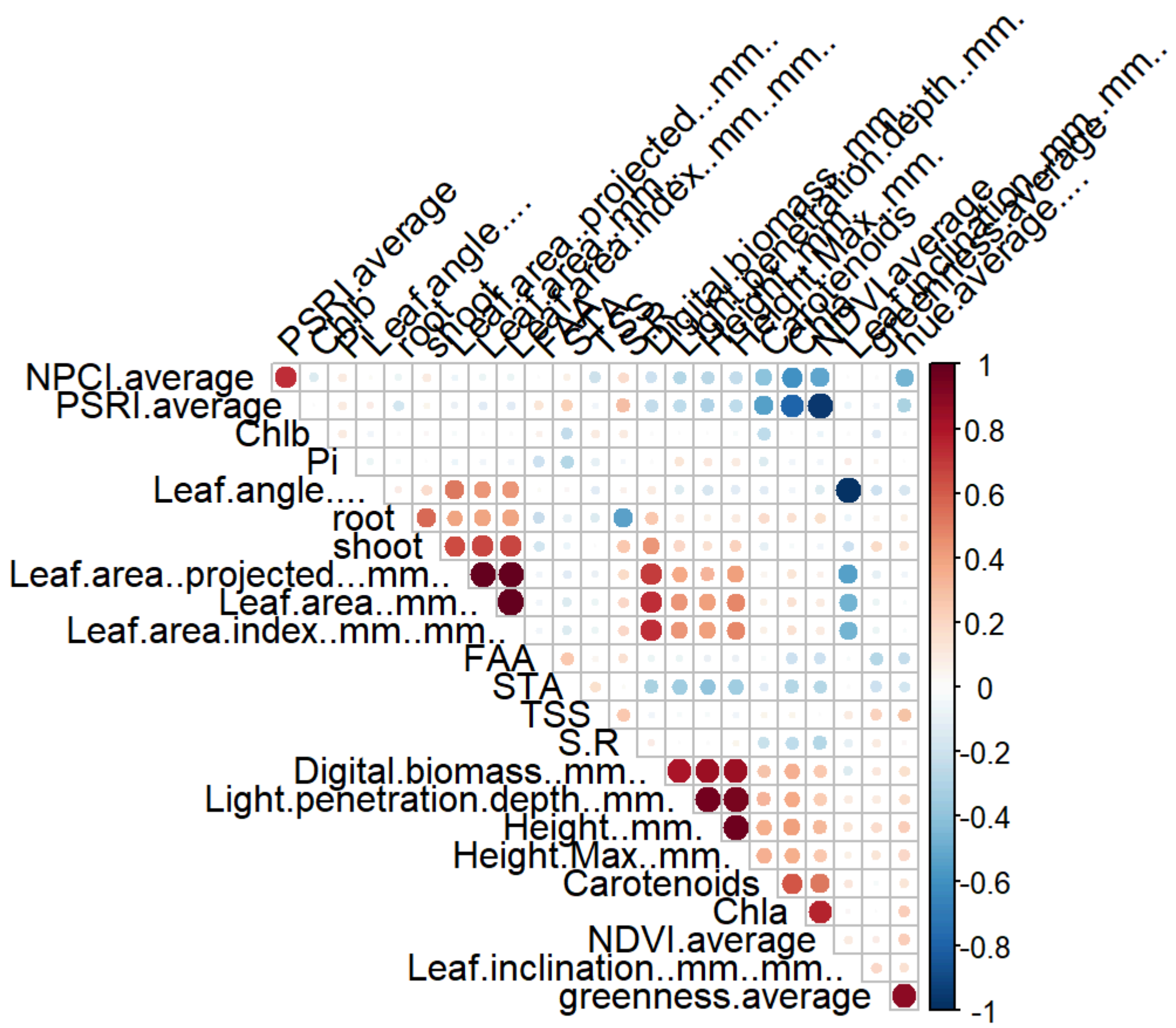

15 - Figure S5B

15.1 - Stat for metabolites

```
#1. Tables with general statistics

data_stats_medians  <- data_metabolites %>%
  group_by(genotype, treatment) %>%
 summarise(medianChla = median(Chla),
medianChlb = median(Chlb),
 medianFAA = median(FAA),
 medianTSS = median(TSS),
medianSTA = median(STA),
          medianPi = median(Pi),
           medianS.R = median(S.R),
           medianshoot = median(shoot),
           medianroot = median(root),
           medianDigital.biomass..mm.. = median(Digital.biomass..mm..),
           medianHeight..mm. = median(Height..mm.),
           medianhue.average.... = median(hue.average....),
           medianLeaf.angle.... = median(Leaf.angle....),
           medianLeaf.area..mm.. = median(Leaf.area..mm..),
           medianLeaf.inclination..mm..mm.. = median(Leaf.inclination..mm..mm..),
           medianLeaf.area..projected...mm.. = median(Leaf.area..projected...mm..),
           medianNDVI.average = median(NDVI.average),
           medianNPCI.average = median(NPCI.average),
           medianPSRI.average = median(PSRI.average),
           medianGrenness = median(greenness.average))
```

```
## `summarise()` has grouped output by 'genotype'. You can override using the
## `.groups` argument.
```

```
#2. pivoted table
data_stats_medians %>%
  pivot_wider(
    names_from = treatment,
    values_from = c(medianChla, medianChlb, medianFAA, medianTSS, medianSTA, medianP
i, medianS.R,medianshoot,medianroot, medianDigital.biomass..mm.., medianHeight..mm.,m
edianhue.average.... , medianLeaf.angle...., medianLeaf.area..mm.., medianLeaf.inclin
ation..mm..mm.., medianLeaf.area..projected...mm.., medianNDVI.average, medianNPCI.av
erage,  medianPSRI.average,medianGrenness)
  ) -> data_stat_pivot

#3. table with ratios
data_stat_pivot %>%
  group_by(genotype) %>%
      summarise(ratio_Chla =medianChla_T/medianChla_K,
                ratio_Chlb =medianChlb_T/medianChlb_K,
                ratio_FAA = medianFAA_T/medianFAA_K,
                ratio_TSS = medianTSS_T/medianTSS_K,
                ratio_STA =medianSTA_T/medianSTA_K,
                ratio_Pi = medianPi_T/medianPi_K,
                ratio_S.R = medianS.R_T/medianS.R_K,
                ratio_shoot = medianshoot_T/medianshoot_K,
                ratio_root = medianroot_T/medianroot_K,
                ratio_hue = medianhue.average...._T/medianhue.average...._K,
                ratio_NDVI = medianNDVI.average_T/medianNDVI.average_K,
                ratio_NPCI = medianNPCI.average_T/medianNPCI.average_K,
                ratio_PSRI = medianPSRI.average_T/medianPSRI.average_K,
                ratio_grenness = medianGrenness_T/medianGrenness_K) -> data_ratio_met
abolites
```

```
#Figure S5B

### Libraries
library(devtools)
library(ggbiplot)
library(FactoMineR)
library(psych)
library(factoextra)
library(dplyr)
library(ggplot2)

### Prepare the Data with Categorical Variables

# Add categorical variables based on the ratio thresholds
data_ratio_metabolites <- data_ratio_metabolites %>%
  mutate(interval_Pi = case_when(ratio_Pi < 0.80 ~ "negative",
                                 ratio_Pi > 1.20 ~ "positive",
                                 ratio_Pi >= 0.80 & ratio_Pi <= 1.20 ~ "non respondin
g"),
         interval_shoot = case_when(ratio_shoot < 0.80 ~ "negative",
                                    ratio_shoot > 1.20 ~ "positive",
                                    ratio_shoot >= 0.80 & ratio_shoot <= 1.20 ~ "non
responding"))

# Create a new column for combined response types
data_response_metabolites <- data_ratio_metabolites %>%
  mutate(interval_response = case_when(
    interval_Pi == "positive" & interval_shoot == "positive" ~ "++",
    interval_Pi == "positive" & interval_shoot == "negative" ~ "+-",
    interval_Pi == "negative" & interval_shoot == "negative" ~ "--",
    interval_Pi == "negative" & interval_shoot == "positive" ~ "-+",
    interval_Pi == "positive" & interval_shoot == "non responding" ~ "+/",
    interval_Pi == "negative" & interval_shoot == "non responding" ~ "-/",
    interval_Pi == "non responding" & interval_shoot == "non responding" ~ "//",
    interval_Pi == "non responding" & interval_shoot == "negative" ~ "/-",
    interval_Pi == "non responding" & interval_shoot == "positive" ~ "/+"
  ))


# Run PCA on the filtered dataset (excluding categorical response columns)
res.pca <- PCA(data_response_metabolites[, -c(1, 16, 17, 18)], graph = FALSE, scale =
TRUE)
```

```
## Warning in PCA(data_response_metabolites[, -c(1, 16, 17, 18)], graph = FALSE, :
## Missing values are imputed by the mean of the variable: you should use the
## imputePCA function of the missMDA package
```

```
# Convert the interval_response to a factor
data_response_metabolites$interval_response <- as.factor(data_response_metabolites$in
terval_response)

# Define the order for interval_response and assign colors
data_response_metabolites$interval_response <- factor(data_response_metabolites$inter
val_response, levels = c("++", "--", "+-", "-+", "-/", "/-", "//", "+/", "/+"))

response_colors <- c("++" = "#00BFFF", "--" = "#FF1493", "+-" = "#00FF00", "-+" = "#9
400D3",
                     "-/" = "#808080", "/-" = "#808080", "//" = "#808080", "+/" = "#8
08080", "/+" = "#808080")

# Create PCA biplot for plant physiological responses
pca_biplot <- fviz_pca_biplot(res.pca,
                               axes = c(1, 2),
                               geom = "text",  # Labels only (no points)
                               col.ind = data_response_metabolites$interval_response,
                               palette = response_colors,
                               legend.title = "[Pi] and shoot biomass response",
                               col.var = "black",
                               labelsize = 4,
                               title = "Plant Physiological Response"
)

# Display PCA biplot for plant responses
pca_biplot
```

```
## Ignoring unknown labels:
## • fill : "[Pi] and shoot biomass response"
## • linetype : "[Pi] and shoot biomass response"
## • shape : "[Pi] and shoot biomass response"
```

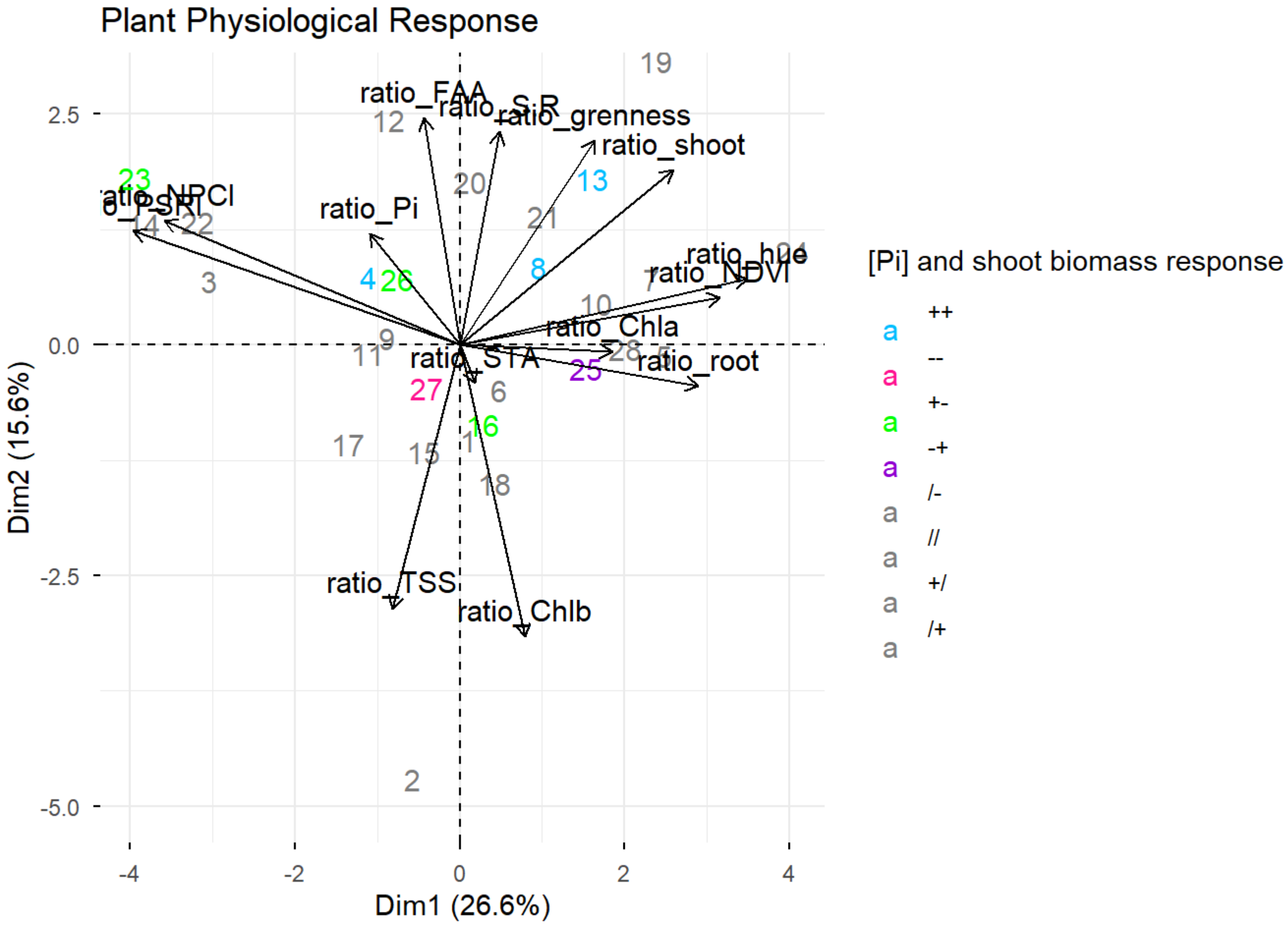
Plant Physiological Response
Dim1 (26.6%)
Dim2 (15.6%)
ratio_FAA
ratio_S.R
ratio_grenness
ratio_shoot
ratio_NPCI
ratio_PSRI
ratio_Pi
ratio_hue
ratio_NDVI
ratio_Chla
ratio_STA
ratio_root
ratio_TSS
ratio_Chlb
[Pi] and shoot biomass response
++
--
+-
-+
/-
//
+/
/+

```
### Genotype Group Assignment

# Function to assign genotype groups
assign_group <- function(genotype) {
  if (genotype %in% c("38", "57", "80", "85", "90")) {
    return("group1")
  } else if (genotype %in% c("11", "53", "66", "72", "103")) {
    return("group2")
  } else if (genotype %in% c("13", "19", "59", "94", "95")) {
    return("group3")
  } else if (genotype %in% c("24", "34", "71", "99", "100", "106")) {
    return("group4")
  } else {
    return("default")
  }
}

# Apply the group assignment function to the dataset
data_response_metabolites$group <- sapply(data_response_metabolites$genotype, assign_
group)

# Color palette for genotypes
genotype_colors <- c(
  "group1" = "#00BFFF",   # group 1
  "group2" = "#00FF00",   # group 2
  "group3" = "#FF1493",   # group 3
  "group4" = "#9400D3",   # group 4
  "default" = "#D3D3D3"   # default gray
)

# Convert 'group' to factor
data_response_metabolites$group <- factor(data_response_metabolites$group, levels = c
("group1", "group2", "group3", "group4", "default"))

# Perform PCA again, now with selected columns
res.pca <- PCA(data_response_metabolites[, -c(1, 16:19)], graph = FALSE, scale = TRU
E)
```

```
## Warning in PCA(data_response_metabolites[, -c(1, 16:19)], graph = FALSE, :
## Missing values are imputed by the mean of the variable: you should use the
## imputePCA function of the missMDA package
```

```
# Extract PCA coordinates for individuals and genotypes
pca_data <- as.data.frame(res.pca$ind$coord)
pca_data$group <- data_response_metabolites$group
pca_data$genotype <- data_response_metabolites$genotype

# Extract PCA coordinates for variables
var_data <- as.data.frame(res.pca$var$coord)
var_names <- rownames(res.pca$var$coord)

# Extend arrows for variable labels
var_data_extended <- var_data %>%
  mutate(Dim.1_end = Dim.1 * 5,  # Extend the arrow by a factor of 5
         Dim.2_end = Dim.2 * 5)

# Create PCA biplot with genotype grouping
pca_biplot <- fviz_pca_biplot(res.pca,
                               axes = c(1, 2),
                               geom.ind = "point",  # Show points for individuals
                               col.var = "black",  # Color of arrows for variables
                               geom.var = "arrow",
                               labelsize = 3,
                               title = "Plant Physiological Response by Genotype Grou
p"
) +
  # Add default group points (grey)
  geom_point(data = pca_data %>% filter(group == "default"),
             aes(x = Dim.1, y = Dim.2),
             size = 4,
             fill = "#D3D3D3",
             color = "black",
             stroke = 0.5,
             shape = 21) +

  # Add colored points for non-default genotypes
  geom_point(data = pca_data %>% filter(group != "default"),
             aes(x = Dim.1, y = Dim.2, fill = group),
             size = 4,
             color = "black",
             stroke = 0.5,
             shape = 21) +

  # Add variable labels
  geom_text(data = var_data_extended,
            aes(x = Dim.1_end, y = Dim.2_end, label = var_names),
            size = 2.5, color = "black", fontface = "plain") +

  # Apply genotype colors
  scale_fill_manual(values = genotype_colors) +

  # Apply theme and titles
  theme_minimal() +
  labs(fill = "Genotype Group")

# Display the final PCA biplot
print(pca_biplot)
```

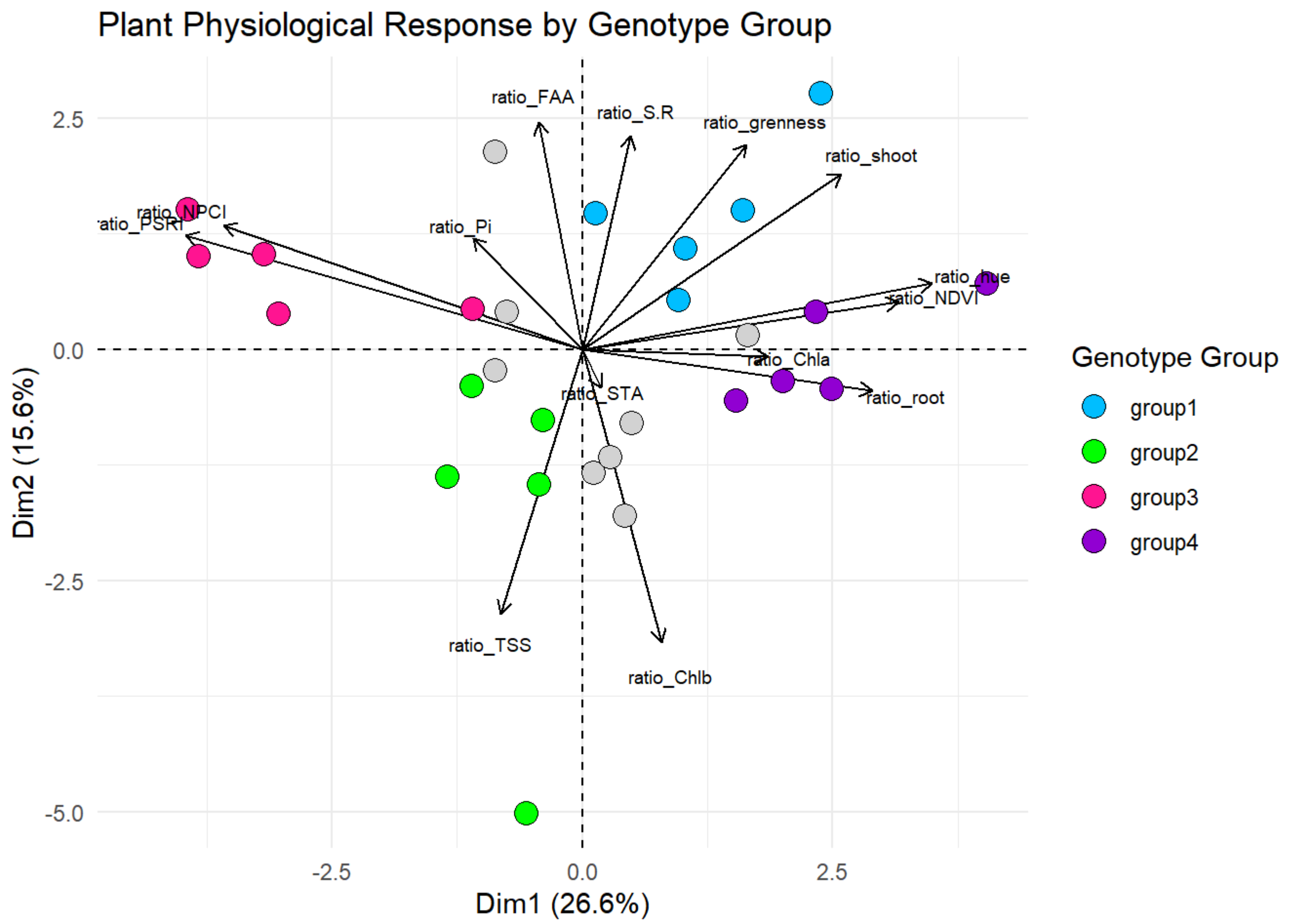

16 - 16S analysis

16.1 - import, filter, merge sequences and assign taxonomy

```
# Dada2 Pipeline for 16S Data Processing Loading Required Libraries
library(dada2)  # DADA2 package for amplicon sequencing analysis

## Defining File Paths Set the path to the directory containing the raw
## sequence files
path <- "C:your path"

# List all files in the directory
list.files(path)

## Extracting Sample Names Extract the forward and reverse fastq filenames for
## paired-end sequences
fnFs <- sort(list.files(path, pattern = "_1.fastq.gz", full.names = TRUE))
fnRs <- sort(list.files(path, pattern = "_2.fastq.gz", full.names = TRUE))

# Extract sample names, assuming filenames have format: SAMPLENAME_XXX.fastq
sample.names <- sapply(strsplit(basename(fnFs), "_"), `[`, 1)

## Inspecting Quality Profiles Visualize the quality profile of forward and
## reverse reads
plotQualityProfile(fnFs[1:103]) -> pq_for
plotQualityProfile(fnRs[1:103]) -> pq_rev

## Filtering Sequences Place filtered files in the 'filtered/' subdirectory
filtFs <- file.path(path, "filtered", paste0(sample.names, "_F_filt.fastq.gz"))
filtRs <- file.path(path, "filtered", paste0(sample.names, "_R_filt.fastq.gz"))
names(filtFs) <- sample.names
names(filtRs) <- sample.names

# Apply filtering steps including trimming, removing phiX, and multi-threading
# if possible
out <- filterAndTrim(fnFs, filtFs, fnRs, filtRs, maxN = 0, maxEE = c(2, 2), truncQ =
2,
    rm.phix = TRUE, compress = TRUE, multithread = FALSE)  # Set multithread=FALSE fo
r Windows
head(out)

## Learning the Error Rates Learn the error rates for forward and reverse
## sequences
errF <- learnErrors(filtFs, multithread = TRUE)
errR <- learnErrors(filtRs, multithread = TRUE)

# Plot error rates to assess quality
plotErrors(errF, nominalQ = TRUE)

## Sample Inference Infer sequence variants for forward and reverse reads
dadaFs <- dada(filtFs, err = errF, multithread = TRUE)
dadaRs <- dada(filtRs, err = errR, multithread = TRUE)

# Inspect the first inferred sequence variant
dadaFs[[1]]

## Merging Forward and Reverse Reads Merge forward and reverse reads to form
## the final sequences
mergers <- mergePairs(dadaFs, filtFs, dadaRs, filtRs, verbose = TRUE)
```

```
# Inspect the merged data frame for the first sample
head(mergers[[1]])

## Constructing the Sequence Table Create a sequence table by combining merged
## reads
seqtab <- makeSequenceTable(mergers)
dim(seqtab)

# Inspect the distribution of sequence lengths
table(nchar(getSequences(seqtab)))

## Removing Chimeras Remove chimeric sequences using the consensus method
seqtab.nochim <- removeBimeraDenovo(seqtab, method = "consensus", multithread = TRUE,
    verbose = TRUE)
dim(seqtab.nochim)

# Calculate the proportion of non-chimeric sequences
sum(seqtab.nochim)/sum(seqtab)

# Save the sequence table without chimeras
write.csv(t(seqtab.nochim), "16S_table-FINAL.csv")

## Tracking Reads Function to get the number of unique sequences
getN <- function(x) sum(getUniques(x))

# Track reads at each step of the pipeline
track <- cbind(out, sapply(dadaFs, getN), sapply(dadaRs, getN), sapply(mergers, get
N),
    rowSums(seqtab.nochim))
# If processing a single sample, remove the sapply calls: e.g. replace
# sapply(dadaFs, getN) with getN(dadaFs)
colnames(track) <- c("input", "filtered", "denoisedF", "denoisedR", "merged", "nonchi
m")
rownames(track) <- sample.names
head(track)

## Assigning Taxonomy Set the working directory for the 16S data
setwd("C:/your_path/")
getwd()

# Load reference database for taxonomy assignment (Silva or others)
ref_database <- "C:/your_path/silva_nr_v138_train_set.fa.gz"

# Assign taxonomy to sequences using the reference database
taxa <- assignTaxonomy(seqtab.nochim, ref_database, multithread = TRUE)

# Remove sequence row names for display
taxa.print <- taxa
rownames(taxa.print) <- NULL
write.csv(taxa, "taxonomy.csv")
```

16.2 - Phyloseq object creation and contamination removal

```
#2- Construct Phyloseq Objects

# Load necessary libraries
library("phyloseq")
```

```
##
## Caricamento pacchetto: 'phyloseq'
```

```
## Il seguente oggetto è mascherato da 'package:psych':
##
##     distance
```

```
library("ggplot2")      # For graphics
library("readxl")       # For importing data from Excel
library("dplyr")        # For filtering and reformatting data frames
library("tibble")
library(devtools)
library(ranacapa)
library("tidyverse")
library("vegan")
```

```
## Caricamento del pacchetto richiesto: permute
```

```
##
## Caricamento pacchetto: 'permute'
```

```
## Il seguente oggetto è mascherato da 'package:devtools':
##
##     check
```

```
##
## Caricamento pacchetto: 'vegan'
```

```
## Il seguente oggetto è mascherato da 'package:psych':
##
##     pca
```

```
## Il seguente oggetto è mascherato da 'package:phytools':
##
##     scores
```

```
# Upload data tables

## ASV Table
asv16S <- read.table(file="C:/Users/Alber/Desktop/DOTTORATO/LATTUGA/LANDLAB/DNA/16S-I
TS/16S/Phyloseq object/16S_table-FINAL.txt", sep="\t", dec = ".", header=TRUE) #repla
ce with your path

## Taxonomy Table
tax16S <- read.table(file="/Users/Alber/Desktop/DOTTORATO/LATTUGA/LANDLAB/DNA/16S-IT
S/16S/Phyloseq object/taxonomy.txt", sep="\t", dec = ".", header=TRUE)#replace with y
our path

## Experimental Design
design <- read.table(file="/Users/Alber/Desktop/DOTTORATO/LATTUGA/LANDLAB/DNA/16S-IT
S/16S/Phyloseq object/design.txt", sep="\t", dec = ",", header=TRUE)#replace with you
r path

# Format data for Phyloseq
asv16S <- asv16S %>%
    tibble::column_to_rownames("OTU.ID")

tax16S <- tax16S %>%
    tibble::column_to_rownames("OTU.ID")

design <- design %>%
    tibble::column_to_rownames("SAMPLE.ID")

# Clean the Taxonomy Table
library(stringr)
tax16S[tax16S == ""] <- NA
tax16S[tax16S == " "] <- NA

# Convert to matrices
OTU <- as.matrix(asv16S, taxa_are_rows = TRUE)
TAXA <- as.matrix(tax16S)

# Create the Phyloseq Object
ps16S_0 <- phyloseq(otu_table(OTU, taxa_are_rows = TRUE), tax_table(TAXA), sample_dat
a(design))

#3- Contamination Removal

# Calculate the number of taxa before filtering
num_taxa_before <- ntaxa(ps16S_0)

# Filter ASV based on taxonomy
ps16S_1.0 <- subset_taxa(ps16S_0, !is.na(Phylum) & !Phylum %in% c("Unassigned"))
ps16S_1a <- subset_taxa(ps16S_1.0, !Order %in% c("Chloroplast"))
ps16S_1b <- subset_taxa(ps16S_1a, !Family %in% c("Mitochondria"))
ps16S_1c <- subset_taxa(ps16S_1b, !Kingdom %in% c("Eukaryota"))
ps16S_1d <- subset_taxa(ps16S_1c, !Kingdom %in% c("Archaea"))

# Rename ASVs
dna.16S <- taxa_names(ps16S_1d)
names(dna.16S) <- taxa_names(ps16S_1d)
```

```
ps16S_2 <- merge_phyloseq(ps16S_1d, dna.16S)
taxa_names(ps16S_2) <- paste0("ASV", seq(ntaxa(ps16S_2)))

# Filter Samples (remove those with less than 100 reads)
ps16S_3 <- prune_samples(sample_sums(ps16S_2) >= 100, ps16S_2)  # From 424 to 417 sam
ples
ps16S_4 <- filter_taxa(ps16S_3, function(x) sum(x) > 0, TRUE)

# Filter ASVs (keep ASVs with greater than 0.1% relative abundance per sample)
ps16S_5 <- transform_sample_counts(ps16S_4, function(x) ifelse((x / sum(x)) >= 0.000
1, x, 0))
ps16S_6 <- filter_taxa(ps16S_5, function(x) sum(x) > 0, TRUE)  # From 5954 to 3345 AS
Vs

# Save cleaned Phyloseq object
saveRDS(ps16S_6, "ps16S_clean.rds")

#4- Sequencing Depth and Reads Lost at Each Step

# Upload cleaned dataset
d16S <- readRDS("ps16S_clean.rds")

# Assess sequencing depth
library(data.table)
```

```
##
## Caricamento pacchetto: 'data.table'
```

```
## I seguenti oggetti sono mascherati da 'package:reshape2':
##
##     dcast, melt
```

```
## I seguenti oggetti sono mascherati da 'package:lubridate':
##
##     hour, isoweek, mday, minute, month, quarter, second, wday, week,
##     yday, year
```

```
## I seguenti oggetti sono mascherati da 'package:dplyr':
##
##     between, first, last
```

```
## Il seguente oggetto è mascherato da 'package:purrr':
##
##     transpose
```

```
sdt16S <- data.table(as(sample_data(d16S), "data.frame"),
                     TotalReads = sample_sums(d16S), keep.rownames = TRUE)
```

16.3 - Rarefaction

```
## Warning in vegan::rarefy(x[i, , drop = FALSE], n, se = se): most observed count
## data have counts 1, but smallest count is 2
## Warning in vegan::rarefy(x[i, , drop = FALSE], n, se = se): most observed count
## data have counts 1, but smallest count is 2
## Warning in vegan::rarefy(x[i, , drop = FALSE], n, se = se): most observed count
## data have counts 1, but smallest count is 2
## Warning in vegan::rarefy(x[i, , drop = FALSE], n, se = se): most observed count
## data have counts 1, but smallest count is 2
```

```
## Warning in vegan::rarefy(x[i, , drop = FALSE], n, se = se): most observed count
## data have counts 1, but smallest count is 3
```

```
## Warning in vegan::rarefy(x[i, , drop = FALSE], n, se = se): most observed count
## data have counts 1, but smallest count is 2
```

```
## Warning in vegan::rarefy(x[i, , drop = FALSE], n, se = se): most observed count
## data have counts 1, but smallest count is 3
```

```
## Warning in vegan::rarefy(x[i, , drop = FALSE], n, se = se): most observed count
## data have counts 1, but smallest count is 4
```

```
## Warning in vegan::rarefy(x[i, , drop = FALSE], n, se = se): most observed count
## data have counts 1, but smallest count is 2
## Warning in vegan::rarefy(x[i, , drop = FALSE], n, se = se): most observed count
## data have counts 1, but smallest count is 2
```

```
## Warning in vegan::rarefy(x[i, , drop = FALSE], n, se = se): most observed count
## data have counts 1, but smallest count is 3
## Warning in vegan::rarefy(x[i, , drop = FALSE], n, se = se): most observed count
## data have counts 1, but smallest count is 3
```

```
## Warning in vegan::rarefy(x[i, , drop = FALSE], n, se = se): most observed count
## data have counts 1, but smallest count is 2
## Warning in vegan::rarefy(x[i, , drop = FALSE], n, se = se): most observed count
## data have counts 1, but smallest count is 2
## Warning in vegan::rarefy(x[i, , drop = FALSE], n, se = se): most observed count
## data have counts 1, but smallest count is 2
```

```
## Warning in vegan::rarefy(x[i, , drop = FALSE], n, se = se): most observed count
## data have counts 1, but smallest count is 3
```

```
## Warning in vegan::rarefy(x[i, , drop = FALSE], n, se = se): most observed count
## data have counts 1, but smallest count is 5
```

```
## Warning in vegan::rarefy(x[i, , drop = FALSE], n, se = se): most observed count
## data have counts 1, but smallest count is 2
```

```
## Warning in vegan::rarefy(x[i, , drop = FALSE], n, se = se): most observed count
## data have counts 1, but smallest count is 5
```

```
## Warning in vegan::rarefy(x[i, , drop = FALSE], n, se = se): most observed count
## data have counts 1, but smallest count is 4
```

```
## Warning in vegan::rarefy(x[i, , drop = FALSE], n, se = se): most observed count
## data have counts 1, but smallest count is 2
```

```
## Warning in vegan::rarefy(x[i, , drop = FALSE], n, se = se): most observed count
## data have counts 1, but smallest count is 3
```

```
## Warning in vegan::rarefy(x[i, , drop = FALSE], n, se = se): most observed count
## data have counts 1, but smallest count is 2
```

```
## Warning in vegan::rarefy(x[i, , drop = FALSE], n, se = se): most observed count
## data have counts 1, but smallest count is 3
```

```
## Warning in vegan::rarefy(x[i, , drop = FALSE], n, se = se): most observed count
## data have counts 1, but smallest count is 2
## Warning in vegan::rarefy(x[i, , drop = FALSE], n, se = se): most observed count
## data have counts 1, but smallest count is 2
```

```
## Warning in vegan::rarefy(x[i, , drop = FALSE], n, se = se): most observed count
## data have counts 1, but smallest count is 3
```

```
## Warning in vegan::rarefy(x[i, , drop = FALSE], n, se = se): most observed count
## data have counts 1, but smallest count is 2
## Warning in vegan::rarefy(x[i, , drop = FALSE], n, se = se): most observed count
## data have counts 1, but smallest count is 2
```

```
## Warning in vegan::rarefy(x[i, , drop = FALSE], n, se = se): most observed count
## data have counts 1, but smallest count is 4
```

```
## Warning in vegan::rarefy(x[i, , drop = FALSE], n, se = se): most observed count
## data have counts 1, but smallest count is 2
```

```
## Warning in vegan::rarefy(x[i, , drop = FALSE], n, se = se): most observed count
## data have counts 1, but smallest count is 3
```

```
## Warning in vegan::rarefy(x[i, , drop = FALSE], n, se = se): most observed count
## data have counts 1, but smallest count is 2
```

```
## Warning in vegan::rarefy(x[i, , drop = FALSE], n, se = se): most observed count
## data have counts 1, but smallest count is 3
## Warning in vegan::rarefy(x[i, , drop = FALSE], n, se = se): most observed count
## data have counts 1, but smallest count is 3
## Warning in vegan::rarefy(x[i, , drop = FALSE], n, se = se): most observed count
## data have counts 1, but smallest count is 3
## Warning in vegan::rarefy(x[i, , drop = FALSE], n, se = se): most observed count
## data have counts 1, but smallest count is 3
```

```
## Warning in vegan::rarefy(x[i, , drop = FALSE], n, se = se): most observed count
## data have counts 1, but smallest count is 2
## Warning in vegan::rarefy(x[i, , drop = FALSE], n, se = se): most observed count
## data have counts 1, but smallest count is 2
```

```
## Warning in vegan::rarefy(x[i, , drop = FALSE], n, se = se): most observed count
## data have counts 1, but smallest count is 3
```

```
## Warning in vegan::rarefy(x[i, , drop = FALSE], n, se = se): most observed count
## data have counts 1, but smallest count is 2
```

```
## Warning in vegan::rarefy(x[i, , drop = FALSE], n, se = se): most observed count
## data have counts 1, but smallest count is 5
```

```
## Warning in vegan::rarefy(x[i, , drop = FALSE], n, se = se): most observed count
## data have counts 1, but smallest count is 3
```

```
## Warning in vegan::rarefy(x[i, , drop = FALSE], n, se = se): most observed count
## data have counts 1, but smallest count is 4
```

```
## Warning in vegan::rarefy(x[i, , drop = FALSE], n, se = se): most observed count
## data have counts 1, but smallest count is 3
```

```
## Warning in vegan::rarefy(x[i, , drop = FALSE], n, se = se): most observed count
## data have counts 1, but smallest count is 2
```

```
## Warning in vegan::rarefy(x[i, , drop = FALSE], n, se = se): most observed count
## data have counts 1, but smallest count is 4
```

```
## Warning in vegan::rarefy(x[i, , drop = FALSE], n, se = se): most observed count
## data have counts 1, but smallest count is 3
```

```
## Warning in vegan::rarefy(x[i, , drop = FALSE], n, se = se): most observed count
## data have counts 1, but smallest count is 2
```

```
## Warning in vegan::rarefy(x[i, , drop = FALSE], n, se = se): most observed count
## data have counts 1, but smallest count is 4
```

```
## Warning in vegan::rarefy(x[i, , drop = FALSE], n, se = se): most observed count
## data have counts 1, but smallest count is 2
## Warning in vegan::rarefy(x[i, , drop = FALSE], n, se = se): most observed count
## data have counts 1, but smallest count is 2
```

```
## Warning: `aes_string()` was deprecated in ggplot2 3.0.0.
## ℹ Please use tidy evaluation idioms with `aes()`.
## ℹ See also `vignette("ggplot2-in-packages")` for more information.
## ℹ The deprecated feature was likely used in the ranacapa package.
##   Please report the issue at <https://github.com/gauravsk/ranacapa/issues>.
## This warning is displayed once every 8 hours.
## Call `lifecycle::last_lifecycle_warnings()` to see where this warning was
## generated.
```

16.4 - Figure S7C - Root-associated bacterial phylum composition

```
# Required Libraries
library(ggplot2)
library(vegan)
library(dplyr)
library(scales)
```

```
##
## Caricamento pacchetto: 'scales'
```

```
## I seguenti oggetti sono mascherati da 'package:psych':
##
##     alpha, rescale
```

```
## Il seguente oggetto è mascherato da 'package:phytools':
##
##     rescale
```

```
## Il seguente oggetto è mascherato da 'package:purrr':
##
##     discard
```

```
## Il seguente oggetto è mascherato da 'package:readr':
##
##     col_factor
```

```
library(grid)
library(reshape2)
library(phyloseq)

# Phylum-Level Agglomeration and Visualization

# Aggregate data by Phylum, calculate relative abundance, and filter low-abundance ta
xa
erie_phylum <- r16S %>%
  tax_glom(taxrank = "Phylum") %>%                     # Agglomerate data at Phylum l
evel
  transform_sample_counts(function(x) {x / sum(x)}) %>% # Transform to relative abund
ance
  psmelt() %>%                                         # Melt data to long format
  filter(Abundance > 0.001) %>%                         # Filter out taxa with low ab
undance (< 0.1%)
  arrange(Phylum)                                      # Sort data by Phylum

# Ensure 'Sample' and 'Tissue' columns are factors for grouping and visualization
erie_phylum$Sample <- as.factor(erie_phylum$Sample)
erie_phylum$tissue <- as.factor(erie_phylum$tissue)

# Set custom colors for Phyla visualization
phylum_colors <- c(
  "#CBD588", "#5F7FC7", "orange", "#DA5724", "#508578", "#CD9BCD",
  "#AD6F3B", "#673770", "#D14285", "#652926", "#C84248",
  "#8569D5", "#5E738F", "#8A7C64", "#7CFC00", "#599861",
  "#FF0000", "#00FF00", "#0000FF", "#FFFF00", "#00FFFF", "#FFC0CB",
  "#008000", "#000080", "#808000", "#D1A33D", "#008080", "#FFD700",
  "#800080", "#FF00FF", "#ADFF2F", "#32CD32", "#00FF7F", "#C0C0C0",
  "#228B22", "#20B2AA", "#40E0D0", "#00BFFF", "#1E90FF", "#4169E1",
  "#0000CD", "#8A2BE2", "#6A5ACD"
)

# Add a dummy variable to group tissues
erie_phylum <- erie_phylum %>%
  mutate(tissue_group = ifelse(tissue == "root", "root", "other"))  # Group 'root' ti
ssue separately

# Plot the relative abundance of Phyla by Sample and Tissue
ggplot(erie_phylum, aes(x = Sample, y = Abundance, fill = Phylum)) +
  facet_wrap(~ tissue_group, scales = 'free_x', nrow = 2) +  # Facet by tissue group,
adjust scales
  geom_bar(stat = "identity") +  # Create bar chart
  scale_fill_manual(values = phylum_colors) +  # Apply custom color palette
  theme(
    axis.title.x = element_text(),
    axis.title.y = element_text(size = 11, face = "bold"),
    plot.title = element_text(size = 12, face = "bold"),
    axis.text.x = element_text(angle = 45, hjust = 1, size = 7),
    strip.background = element_blank(),  # Remove background from facet strips
    strip.placement = "outside",  # Place facet labels outside the plot area
    panel.spacing = unit(0, "lines"),  # Remove spacing between panels
    legend.position = "bottom",  # Position legend at the bottom
    legend.justification = "center",  # Center the legend
```

```
    legend.box.spacing = unit(0.5, "lines"),  # Adjust spacing around legend
    legend.title = element_text(size = 8),  # Reduce size of legend title
    legend.text = element_text(size = 7)  # Reduce size of legend text
  ) +
  guides(fill = guide_legend(reverse = FALSE, keywidth = 0.5, keyheight = 0.5, ncol =
7)) +  # Adjust legend keys and orientation
  ylab("Relative Abundance (Phyla > 0.001%)") +  # Label y-axis
  ggtitle("Landlab 16S Root-Associated Bacterial Community")  # Plot title
```

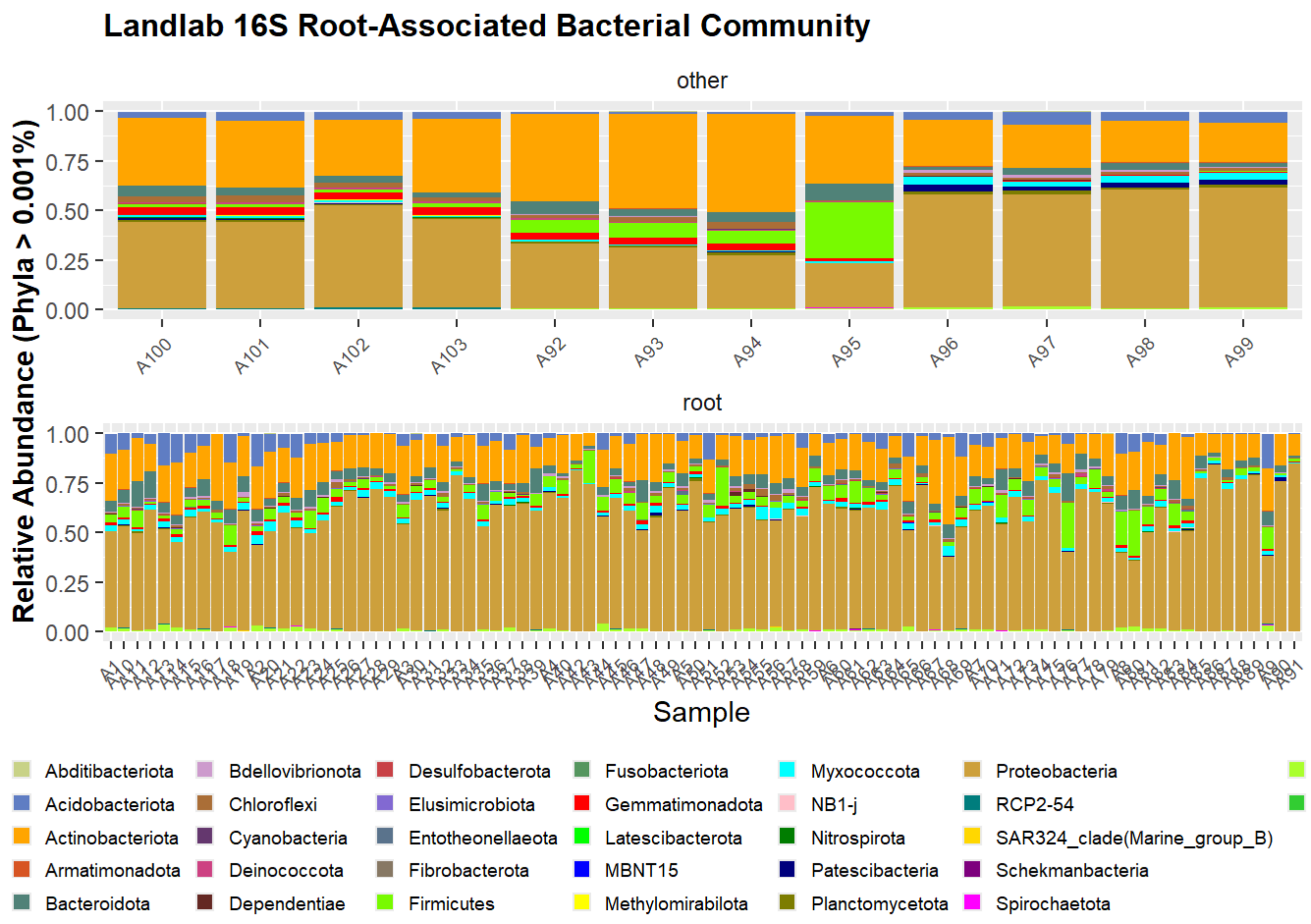

```
#####################Supplementary table 18

# Calculate Relative Abundance for Germination Soil

# Aggregate data by Phylum and calculate relative abundance for germination soil
erie_phylum <- r16S %>%
  tax_glom(taxrank = "Phylum") %>%                     # Agglomerate at Phylum level
  transform_sample_counts(function(x) {x / sum(x) * 100}) %>% # Calculate relative ab
undance in percentage
  psmelt() %>%                                         # Convert to long format
  filter(tissue == "root") %>%              # Change with the other tissues (inoculu
m, root, growth soil)
  arrange(Phylum)                                      # Sort by Phylum

# Identify the top Phyla by mean relative abundance
top_phyla <- erie_phylum %>%
  group_by(Phylum) %>%
  summarize(mean_abundance = round(mean(Abundance), 1)) %>%  # Calculate mean and rou
nd to 1 decimal place
  arrange(desc(mean_abundance))  # Sort by descending mean abundance

top_phyla
```

**Phylum**<br><chr>	**mean_abundance**<br><dbl>
Proteobacteria	60.0
Actinobacteriota	18.4
Firmicutes	5.4
Bacteroidota	5.1
Acidobacteriota	4.2
Myxococcota	2.4
Verrucomicrobiota	1.0
Chloroflexi	0.8
Gemmatimonadota	0.7
Bdellovibrionota	0.6

1-10 of 37 rows Previous **1** 2 3 4 Next

16.5 - alpha diversity

```
# Load the required package for alpha-diversity estimation
library(picante)
```

```
## Caricamento del pacchetto richiesto: nlme
```

```
##
## Caricamento pacchetto: 'nlme'
```

```
## Il seguente oggetto è mascherato da 'package:dplyr':
##
##     collapse
```

```
library(tidyverse)
library(ggpubr)
library(rstatix)
library(tidyverse)
library(ggthemes)
library(multcompView)
library(agricolae)
```

```
##
## Caricamento pacchetto: 'agricolae'
```

```
## Il seguente oggetto è mascherato da 'package:factoextra':
##
##     hcut
```

```
## Il seguente oggetto è mascherato da 'package:ape':
##
##     consensus
```

```
library(dplyr)



# Estimate alpha-diversity measures: Observed, InvSimpson, and Shannon
table_r16S <- estimate_richness(r16S, split = TRUE, measures=c("Observed", "InvSimpso
n","Shannon"))

# Bind the design data with the alpha-diversity measures
sdr16S <- sample_data(r16S)
datar16S <- cbind(sample_data(sdr16S), table_r16S)

#tranform to factor
datar16S$genotype <- as.factor(datar16S$genotype)
datar16S$tissue<-as.factor(datar16S$tissue)
datar16S$Treatment<-as.factor(datar16S$Treatment)
datar16S$variety<-as.factor(datar16S$variety)
datar16S$genetic.distance<- as.factor(datar16S$genetic.distance)
datar16S$interval_response<- as.factor(datar16S$interval_response)
datar16S$Pi.response<- as.factor(datar16S$Pi.response)
datar16S$Shoot.response<- as.factor(datar16S$Shoot.response)
datar16S$phenotypic_group<- as.factor(datar16S$phenotypic_group)
```

16.6 - Figure S7A-B (upper part)

```
# Define a color palette for compartments
compartment_palette <- c("#a78b4d", "#cfb26e", "#e1cba8", "#cc742c")

#1. Perform Dunn's test for Observed diversity between tissues, with FDR correction
res_obs <- datar16S %>%
  dunn_test(Observed ~ tissue, p.adjust.method = "fdr")

# Add statistical test results to plot
stat.test <- res_obs %>% add_xy_position(x = "tissue")

# Create a boxplot for Observed ASV richness with jittered points
plot_16S_Observed_b <- ggplot(datar16S, aes(tissue, Observed)) +
  geom_boxplot(aes(fill = tissue), show.legend = TRUE, outlier.shape = NA) +  # Hide
outliers
  geom_jitter(size = 3) +  # Add jittered points for better visibility
  labs(x = "Tissue Type", y = "Observed ASV Richness") +  # Label axes
  theme_bw() +  # Use a clean theme
  theme(panel.grid.major = element_blank(), panel.grid.minor = element_blank()) +  #
Remove grid lines
  stat_pvalue_manual(stat.test, hide.ns = TRUE) +  # Display p-values, hide non-signi
ficant results
  scale_fill_manual(values = compartment_palette)  # Apply custom color palette

# Display the plot
plot_16S_Observed_b
```

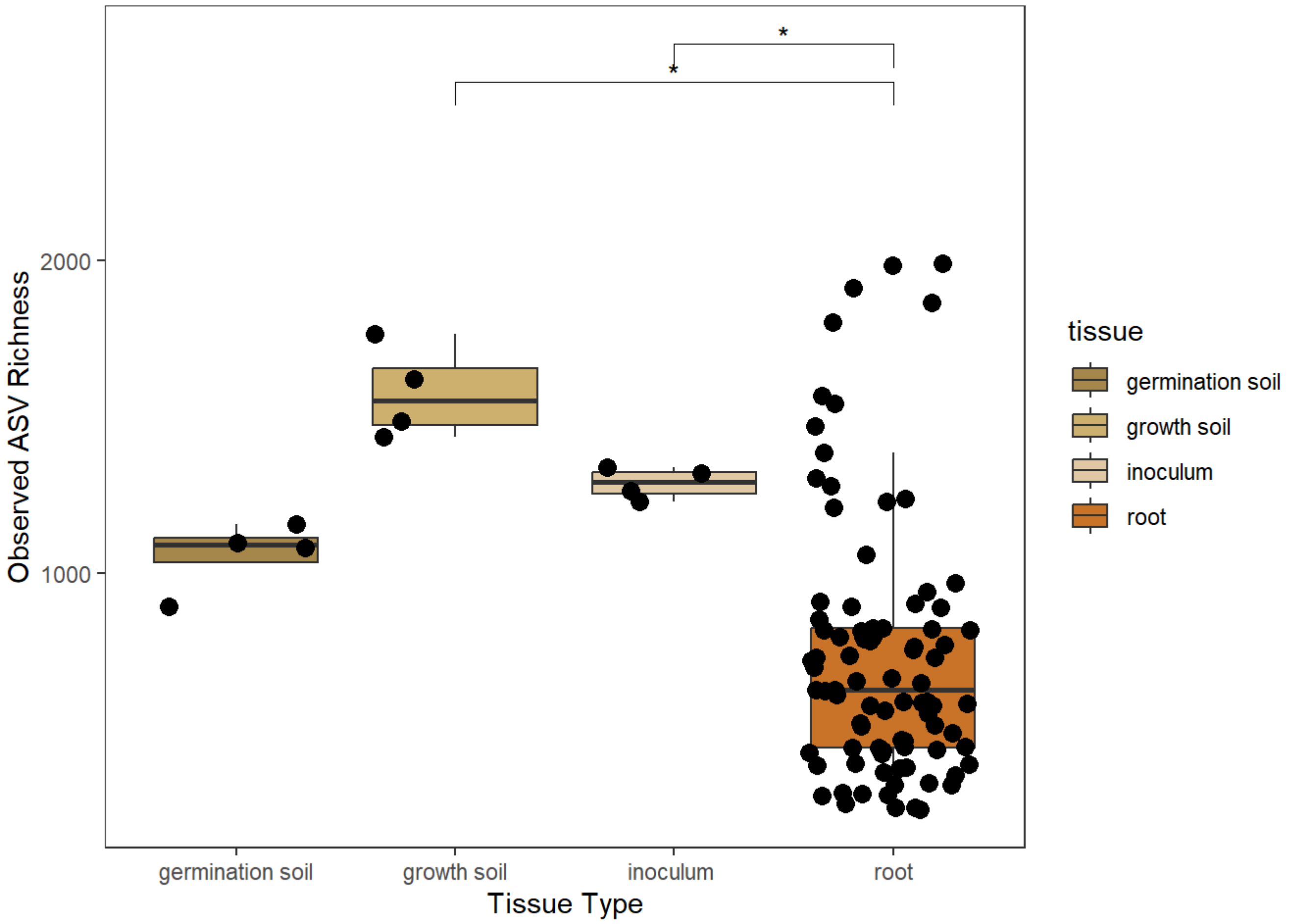

```
#2. Perform Dunn's test for Shannon diversity between tissues, with FDR correction
res_shan <- datar16S %>%
  dunn_test(Shannon ~ tissue, p.adjust.method = "fdr")

# Add statistical test results to plot
stat.test <- res_shan %>% add_xy_position(x = "tissue")

# Create a boxplot for Shannon ASV richness with jittered points
plot_16S_Shannon_b <- ggplot(datar16S, aes(tissue, Shannon)) +
  geom_boxplot(aes(fill = tissue), show.legend = TRUE, outlier.shape = NA) +  # Hide
outliers
  geom_jitter(size = 3) +  # Add jittered points
  labs(x = "Tissue Type", y = "Shannon ASV Richness") +  # Label axes
  theme_bw() +  # Use a clean theme
  theme(panel.grid.major = element_blank(), panel.grid.minor = element_blank()) +  #
Remove grid lines
  stat_pvalue_manual(stat.test, hide.ns = TRUE) +  # Display p-values
  scale_fill_manual(values = compartment_palette)  # Apply custom color palette

# Display the plot
plot_16S_Shannon_b
```

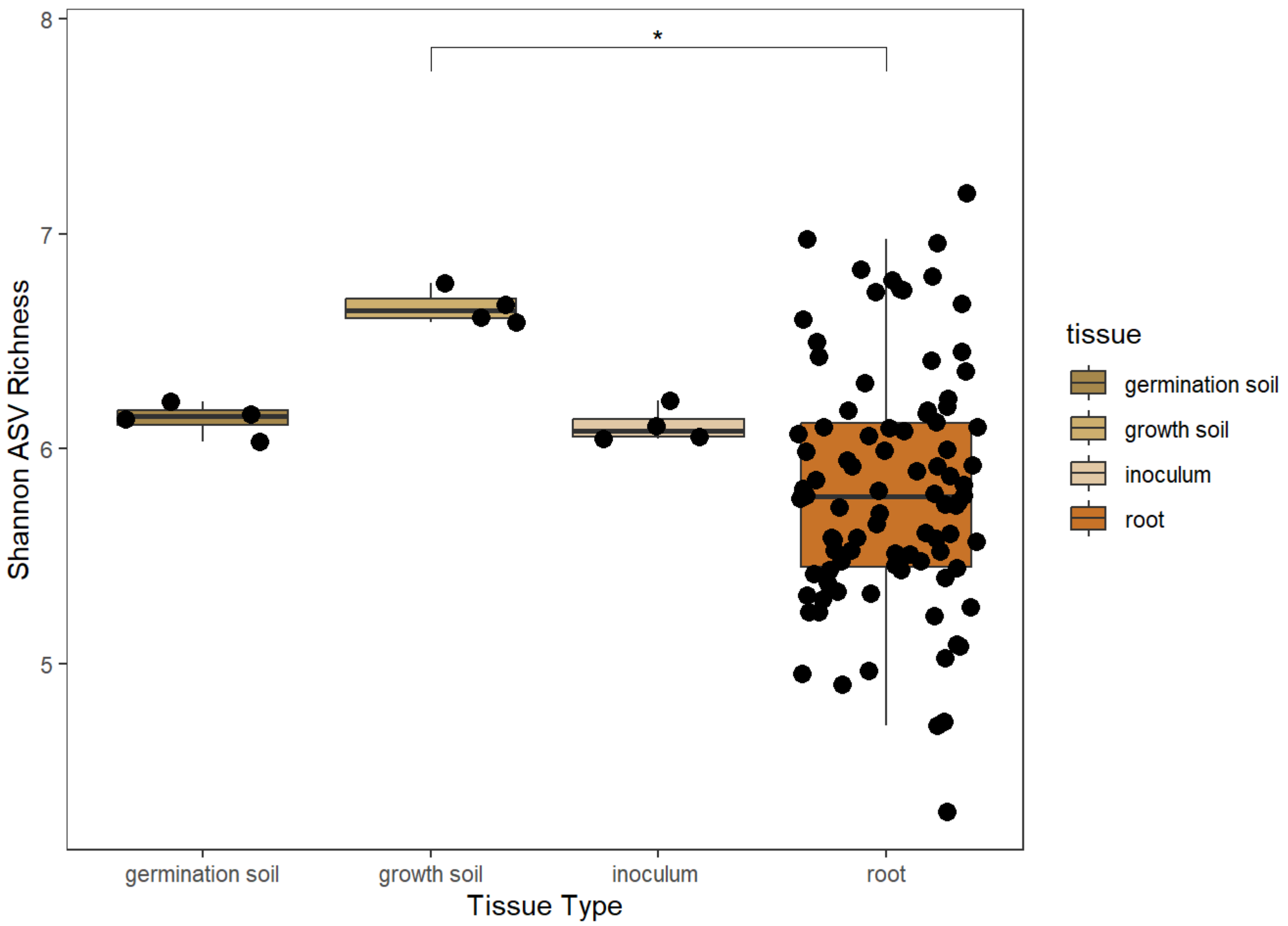

16.7 - Figure S7A-B (lower part)

```
# Subset the dataset for the root tissue and specific treatments (T and CTRL)
datar16S_root <- datar16S %>%
  filter(tissue == "root") %>%
  filter(Treatment == "T" | Treatment == "CTRL") %>%
  droplevels()

# Define a color palette for treatments
paletteT <- c("gray90", "#cc742c")

# Convert relevant variables to factors for appropriate statistical analysis
datar16S_root$genotype <- as.factor(datar16S_root$genotype)
datar16S_root$tissue <- as.factor(datar16S_root$tissue)
datar16S_root$Treatment <- as.factor(datar16S_root$Treatment)
datar16S_root$interval_response <- as.factor(datar16S_root$interval_response)
datar16S_root$variety <- as.factor(datar16S_root$variety)
datar16S_root$genetic.distance <- as.factor(datar16S_root$genetic.distance)
datar16S_root$phenotypic_group <- as.factor(datar16S_root$phenotypic_group)

# Perform Dunn's test for observed ASV richness between treatments with FDR correctio
n
res_obs <- datar16S_root %>%
  dunn_test(Observed ~ Treatment, p.adjust.method = "fdr")

# Add statistical test results to plot
stat.test_obs <- res_obs %>% add_xy_position(x = "Treatment")

# Create a boxplot for observed ASV richness with jittered points
plot_16S_Observed_b <- ggplot(datar16S_root, aes(Treatment, Observed)) +
  geom_boxplot(aes(fill = Treatment), show.legend = TRUE, outlier.shape = NA) +  # Hi
de outliers
  geom_jitter(size = 3) +  # Add jittered points for better visibility
  labs(x = "Treatment", y = "Observed ASV Richness") +  # Label axes
  theme_bw() +  # Use a clean theme
  theme(panel.grid.major = element_blank(), panel.grid.minor = element_blank()) +  #
Remove grid lines
  stat_pvalue_manual(stat.test_obs, hide.ns = TRUE) +  # Display p-values, hide non-s
ignificant results
  scale_fill_manual(values = paletteT)  # Apply custom color palette

# Display the plot for observed ASV richness
plot_16S_Observed_b
```

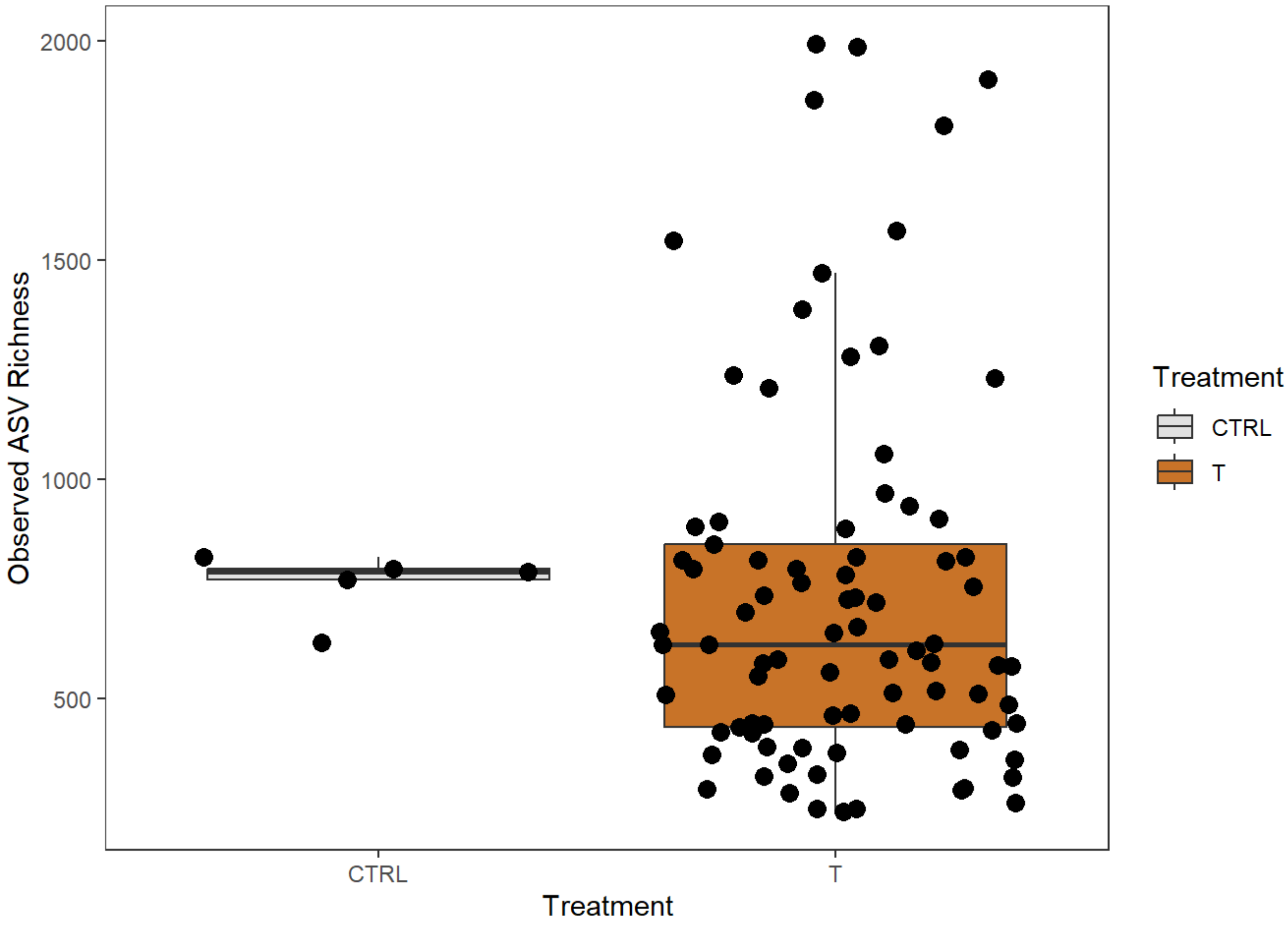


```
# Perform Dunn's test for Shannon diversity between treatments with FDR correction
res_shan <- datar16S_root %>%
  dunn_test(Shannon ~ Treatment, p.adjust.method = "fdr")

# Add statistical test results to plot
stat.test_shan <- res_shan %>% add_xy_position(x = "Treatment")

# Create a boxplot for Shannon ASV richness with jittered points
plot_16S_Shannon_b <- ggplot(datar16S_root, aes(Treatment, Shannon)) +
  geom_boxplot(aes(fill = Treatment), show.legend = TRUE, outlier.shape = NA) +  # Hi
de outliers
  geom_jitter(size = 3) +  # Add jittered points
  labs(x = "Treatment", y = "Shannon ASV Richness") +  # Label axes
  theme_bw() +  # Use a clean theme
  theme(panel.grid.major = element_blank(), panel.grid.minor = element_blank()) +  #
Remove grid lines
  stat_pvalue_manual(stat.test_shan, hide.ns = TRUE) +  # Display p-values
  scale_fill_manual(values = paletteT)  # Apply custom color palette

# Display the plot for Shannon ASV richness
plot_16S_Shannon_b
```

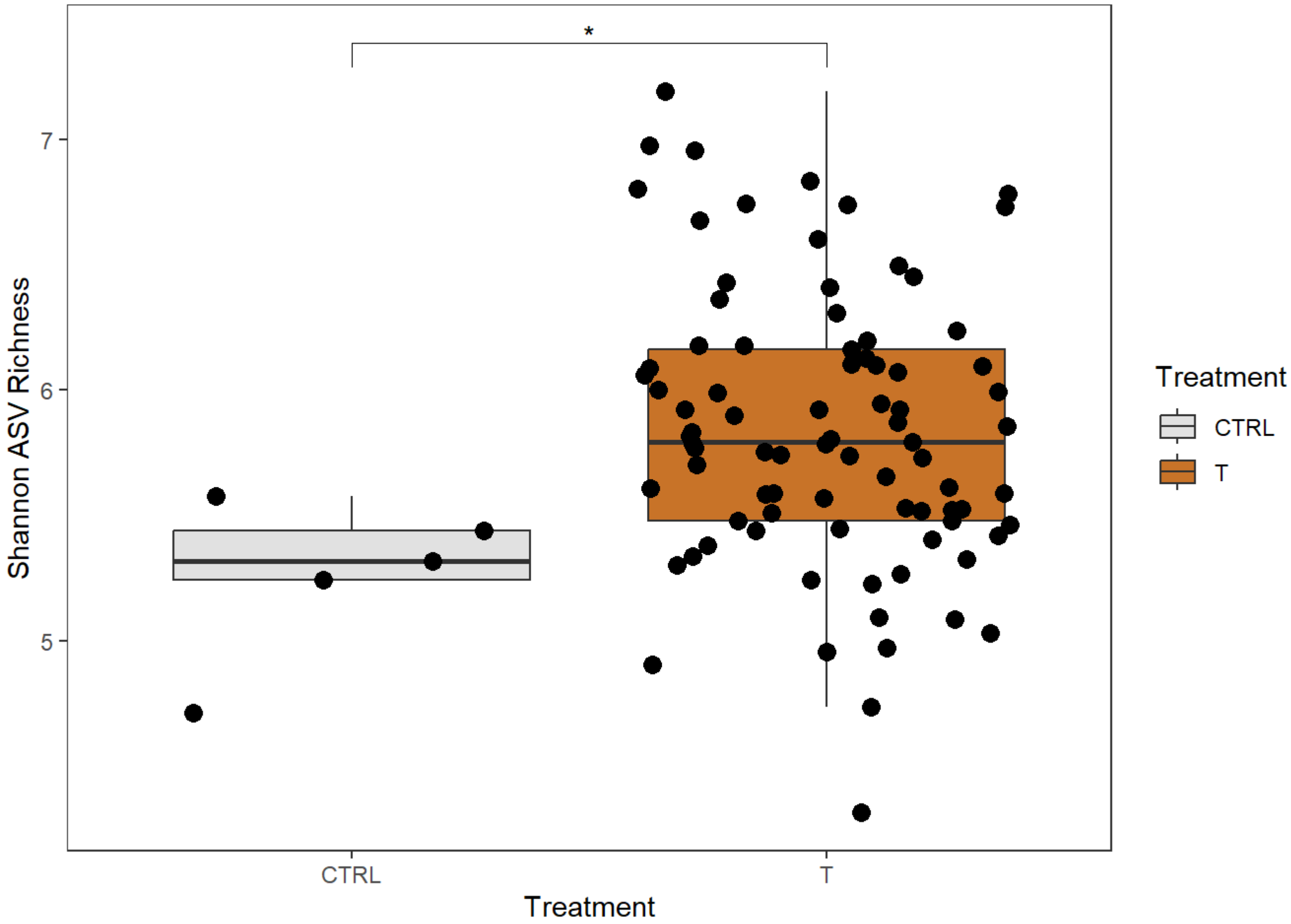

16.8 - Figure S8-B - alpha diversity across phenotypic groups

```
# Filter the dataset for the 'T' treatment and specific phenotypic groups (1-4)
datar16S_root_pheno <- datar16S_root %>%
  filter(Treatment == "T") %>%
  filter(phenotypic_group %in% c("1", "2", "3", "4")) %>%
  droplevels()

#color palette for phenotypic group
genotype_colors <- c(
  "1" = "#00BFFF",   # gruppo 1
  "2" = "#00FF00",   # gruppo 2
  "3" = "#FF1493",   # gruppo 3
  "4" = "#9400D3"    # gruppo 4
)

# 1. Shannon alpha diversity

# Perform Dunn's test for Shannon diversity across phenotypic groups, with FDR correc
tion
res_obs <- dunn_test(Shannon ~ phenotypic_group, data = datar16S_root_pheno, p.adjus
t.method = "fdr")
stat.test <- res_obs %>% add_xy_position(x = "phenotypic_group")

# Convert 'phenotypic_group' to a factor
datar16S_root_pheno$phenotypic_group <- as.factor(datar16S_root_pheno$phenotypic_grou
p)

# Create a plot to visualize Shannon diversity across phenotypic groups
plot_interval_response_shannon <- ggplot(datar16S_root_pheno, aes(x = phenotypic_grou
p, y = Shannon)) +
  geom_jitter(aes(fill = phenotypic_group),
              width = 0.2,        # Slight jitter along the x-axis
              height = 0,         # No jitter along the y-axis
              size = 7,           # Point size
              shape = 21) +       # Point type (filled)
  stat_summary(fun = median,
               geom = "crossbar",
               width = 0.7,       # Crossbar width
               size = 0.6,        # Crossbar thickness
               color = "black") +  # Crossbar color
  labs(x = "Phenotypic Response", y = "Shannon Index") +  # Axis labels
  scale_fill_manual(values = genotype_colors) +  # Custom color palette
  stat_pvalue_manual(stat.test, hide.ns = TRUE) +  # Display p-values, hide non-signi
ficant results
  theme_bw() +  # Clean theme
  theme(panel.grid.major = element_blank(),
        panel.grid.minor = element_blank(),
        legend.title = element_blank(),  # Remove legend title
        axis.title.x = element_text(hjust = 0.5),  # Center the x-axis title
        axis.text.x = element_text(hjust = 0.5))   # Center the x-axis text

# Display the Shannon diversity plot
plot_interval_response_shannon
```

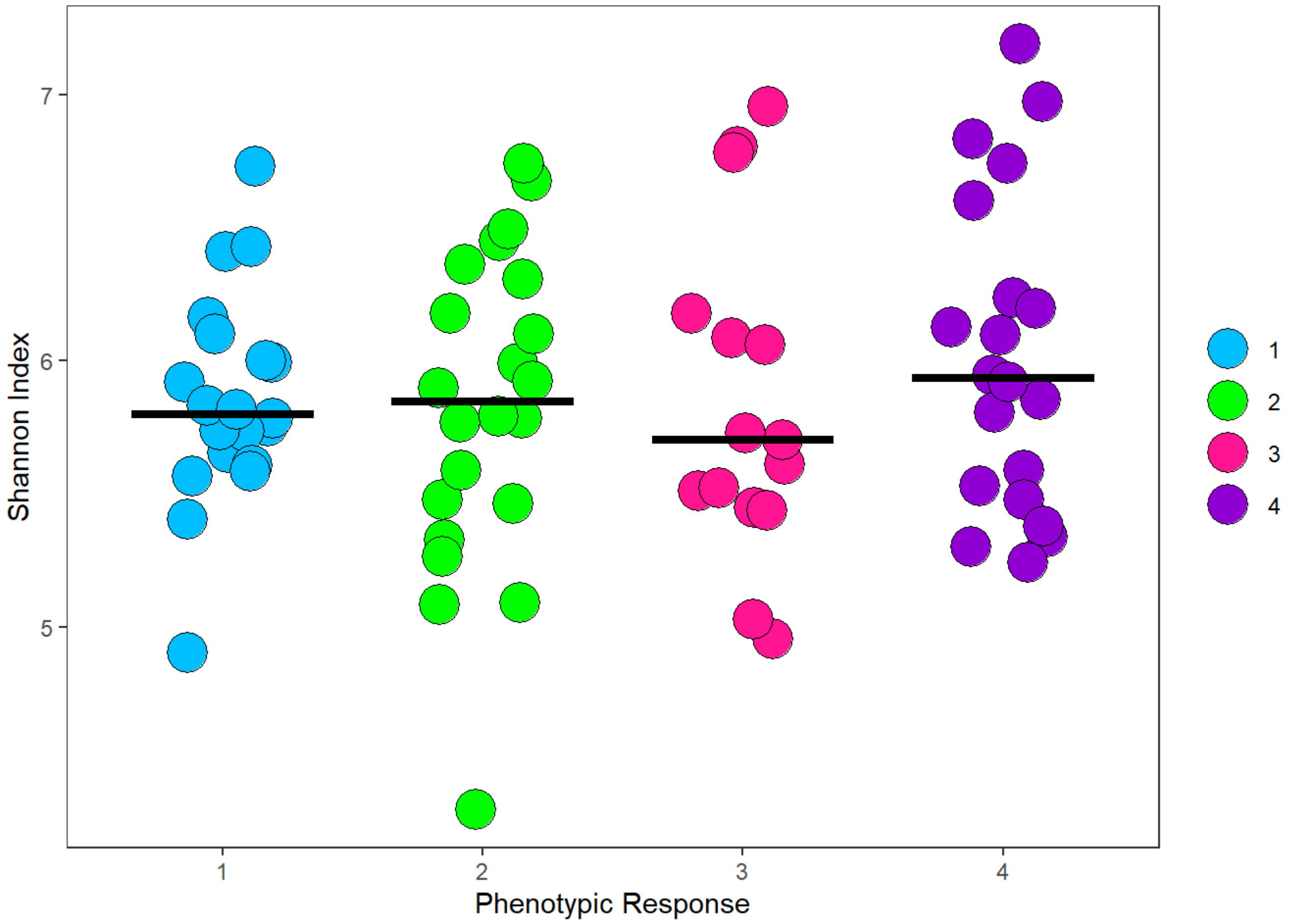

7
6
5
Shannon Index
1
2
3
4
Phenotypic Response
1
2
3
4

```
# 2. Observed alpha diversity

# Repeat for Observed ASV richness
datar16S_root_pheno <- datar16S_root %>%
  filter(Treatment == "T") %>%
  filter(phenotypic_group %in% c("1", "2", "3", "4")) %>%
  droplevels()

# Perform Dunn's test for Observed ASV richness across phenotypic groups, with FDR co
rrection
res_obs <- dunn_test(Observed ~ phenotypic_group, data = datar16S_root_pheno, p.adjus
t.method = "fdr")
stat.test <- res_obs %>% add_xy_position(x = "phenotypic_group")

# Convert 'phenotypic_group' to a factor
datar16S_root_pheno$phenotypic_group <- as.factor(datar16S_root_pheno$phenotypic_grou
p)

# Create a plot to visualize Observed ASV richness across phenotypic groups
plot_interval_response_observed <- ggplot(datar16S_root_pheno, aes(x = phenotypic_gro
up, y = Observed)) +
  geom_jitter(aes(fill = phenotypic_group),
              width = 0.2,        # Slight jitter along the x-axis
              height = 0,         # No jitter along the y-axis
              size = 7,           # Point size
              shape = 21) +       # Point type (filled)
  stat_summary(fun = median,
               geom = "crossbar",
               width = 0.7,       # Crossbar width
               size = 0.6,        # Crossbar thickness
               color = "black") +  # Crossbar color
  labs(x = "Phenotypic Response", y = "Observed Index") +  # Axis labels
  scale_fill_manual(values = genotype_colors) +  # Custom color palette
  stat_pvalue_manual(stat.test, hide.ns = TRUE) +  # Display p-values, hide non-signi
ficant results
  theme_bw() +  # Clean theme
  theme(panel.grid.major = element_blank(),
        panel.grid.minor = element_blank(),
        legend.title = element_blank(),  # Remove legend title
        axis.title.x = element_text(hjust = 0.5),  # Center the x-axis title
        axis.text.x = element_text(hjust = 0.5))   # Center the x-axis text

# Display the Observed ASV richness plot
plot_interval_response_observed
```

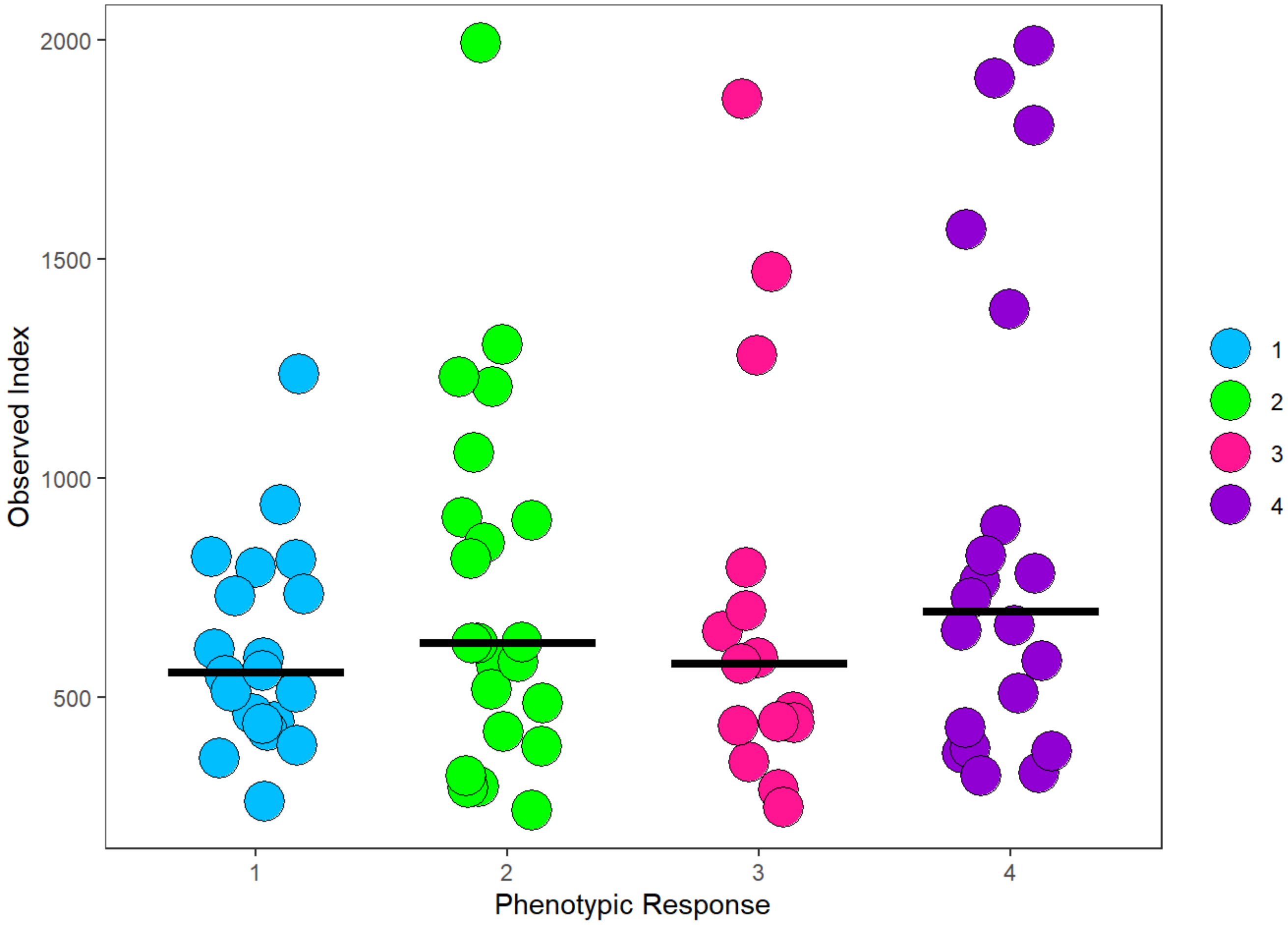

16.9 - Figure 5B, Figure S10A - alpha diversity between Leaf Pi and shoot biomass response groups

```
#1. Shannon Index

# Filter data for treatment 'T' and selected interval responses
datar16S_root2 <- datar16S_root %>%
  filter(Treatment == "T") %>%
  filter(interval_response %in% c("--", "-+", "+-", "++")) %>%
  droplevels()

# Statistical analysis: Dunn's test for Shannon Index across interval responses
res_obs <- dunn_test(Shannon ~ interval_response, data = datar16S_root2, p.adjust.met
hod = "fdr")
stat.test <- res_obs %>% add_xy_position(x = "interval_response")

# Convert 'interval_response' and 'phenotypic_group' to factors
datar16S_root2$interval_response <- as.factor(datar16S_root2$interval_response)
datar16S_root2$phenotypic_group <- as.factor(datar16S_root2$phenotypic_group)

# Plot Shannon Index across interval responses
plot_interval_response_shannon <- ggplot(datar16S_root2, aes(x = interval_response, y
= Shannon)) +
  geom_jitter(aes(fill = phenotypic_group),
              width = 0.2,        # Slight jitter along the x-axis
              height = 0,         # No jitter along the y-axis
              size = 7,           # Point size
              shape = 21) +       # Filled circle shape
  stat_summary(fun = median,
               geom = "crossbar",
               width = 0.7,       # Crossbar width
               size = 0.6,        # Crossbar thickness
               color = "black") +  # Crossbar color
  labs(x = "Interval Response", y = "Shannon Index") +  # Axis labels
  scale_fill_manual(values = genotype_colors) +  # Custom color palette for phenotypi
c groups
  stat_pvalue_manual(stat.test, hide.ns = TRUE) +  # Add p-values, hide non-significa
nt results
  theme_bw() +  # Clean theme
  theme(panel.grid.major = element_blank(),
        panel.grid.minor = element_blank(),
        legend.title = element_blank(),  # Remove legend title
        axis.title.x = element_text(hjust = 0.5),  # Center x-axis title
        axis.text.x = element_text(hjust = 0.5))   # Center x-axis text

# Display Shannon Index plot
plot_interval_response_shannon
```

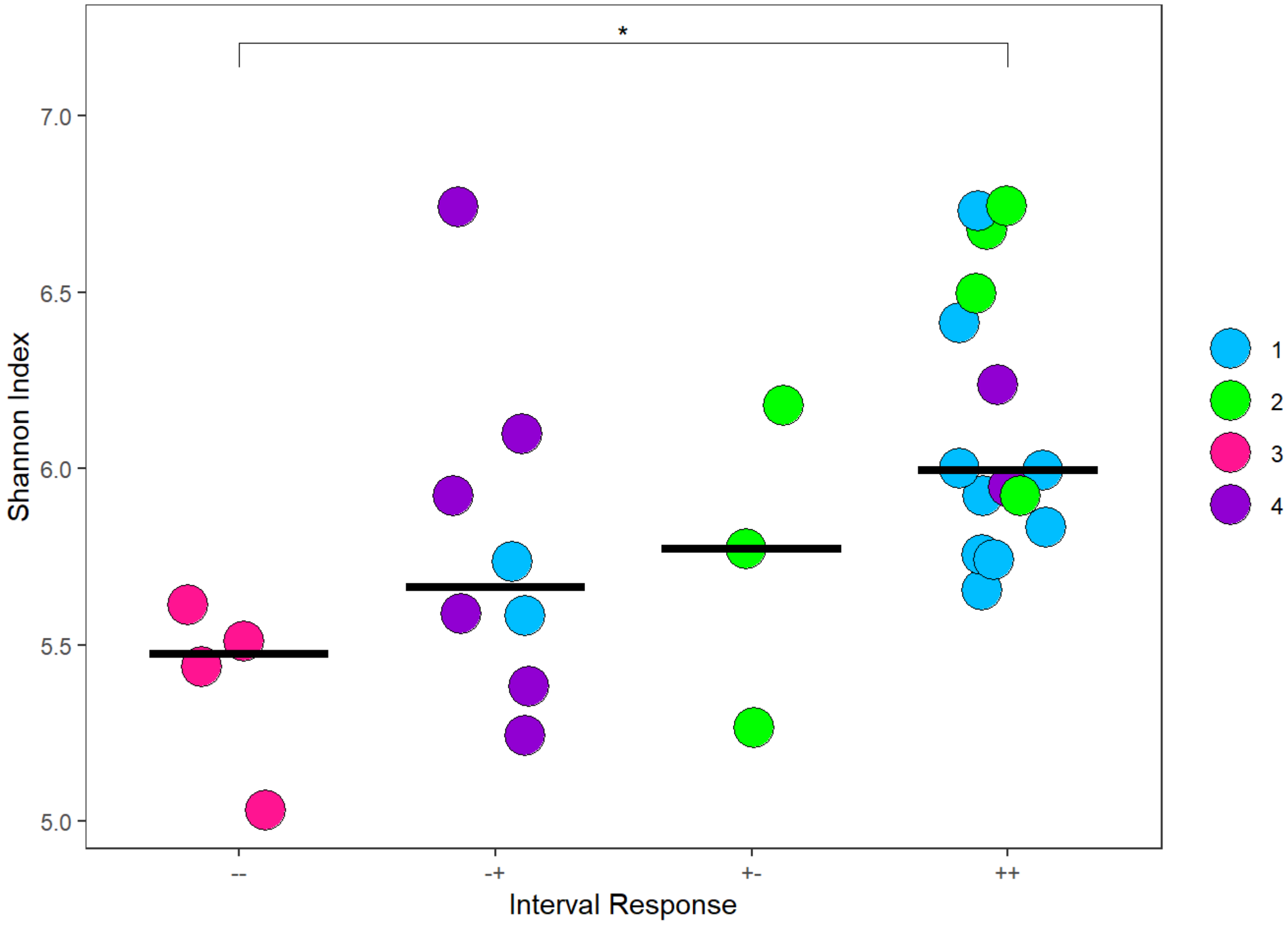
*
7.0
6.5
6.0
5.5
5.0
Shannon Index
--
-+
+-
++
Interval Response
1
2
3
4

```
#2. Observed Index

# Statistical analysis: Dunn's test for Observed ASV richness across interval respons
es
res_obs <- dunn_test(Observed ~ interval_response, data = datar16S_root2, p.adjust.me
thod = "fdr")
stat.test <- res_obs %>% add_xy_position(x = "interval_response")

# Convert 'interval_response' and 'phenotypic_group' to factors
datar16S_root2$interval_response <- as.factor(datar16S_root2$interval_response)
datar16S_root2$phenotypic_group <- as.factor(datar16S_root2$phenotypic_group)

# Plot Observed ASV richness across interval responses
plot_interval_response_observed <- ggplot(datar16S_root2, aes(x = interval_response,
y = Observed)) +
  geom_jitter(aes(fill = phenotypic_group),
              width = 0.2,        # Slight jitter along the x-axis
              height = 0,         # No jitter along the y-axis
              size = 5,           # Point size
              shape = 21) +       # Filled circle shape
  stat_summary(fun = median,
               geom = "crossbar",
               width = 0.6,       # Crossbar width
               size = 0.6,        # Crossbar thickness
               color = "black") +  # Crossbar color
  labs(x = "Interval Response", y = "Observed Index") +  # Axis labels
  scale_fill_manual(values = genotype_colors) +  # Custom color palette for phenotypi
c groups
  stat_pvalue_manual(stat.test, hide.ns = TRUE) +  # Add p-values, hide non-significa
nt results
  theme_bw() +  # Clean theme
  theme(panel.grid.major = element_blank(),
        panel.grid.minor = element_blank(),
        legend.title = element_blank(),  # Remove legend title
        axis.title.x = element_text(hjust = 0.5),  # Center x-axis title
        axis.text.x = element_text(hjust = 0.5))   # Center x-axis text

# Display Observed ASV richness plot
plot_interval_response_observed
```

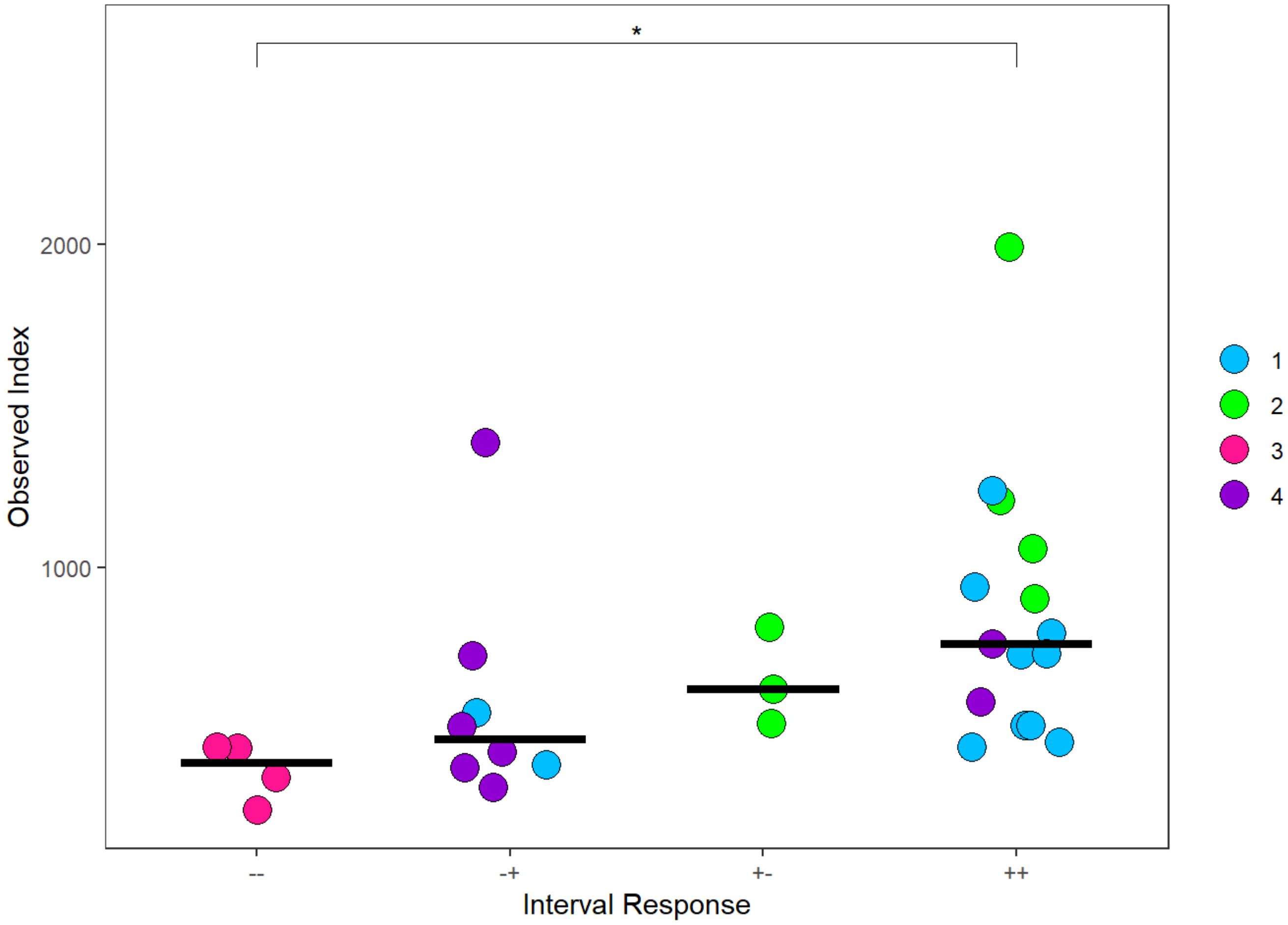

16.10 - Figure 5E, Figure S10B - alpha diversity between Leaf Pi response groups

```
#Figure 5C
#1. Shannon Index - Pi.response

# Filter data for treatment "T" and selected Pi.response values
datar16S_root3 <- datar16S_root %>%
  filter(Treatment == "T") %>%
  filter(Pi.response %in% c("-", "+")) %>%
  droplevels()

# Perform Dunn's test for Shannon diversity index by Pi.response
res_shan <- dunn_test(Shannon ~ Pi.response, data = datar16S_root3, p.adjust.method =
"fdr")
stat.test <- res_shan %>% add_xy_position(x = "Pi.response")

# Plot Shannon Index across Pi.response groups
plot_16S_Shannon_b <- ggplot(datar16S_root3, aes(Pi.response, Shannon)) +
  geom_boxplot(aes(fill = Pi.response), show.legend = TRUE, outlier.shape = NA) +
  geom_jitter(size = 3) +  # Add jittered points for better visualization
  labs(x = "Response Group", y = "Shannon ASV Richness") +  # Axis labels
  theme_bw() +  # Clean theme
  theme(panel.grid.major = element_blank(), panel.grid.minor = element_blank()) +  #
Remove grid lines
  stat_pvalue_manual(stat.test, hide.ns = TRUE) +  # Add statistical p-values, hiding
non-significant results
  scale_fill_manual(values = c("#ece7f2", "#2b8cbe"))  # Custom color palette

# Display Shannon Index plot
plot_16S_Shannon_b
```

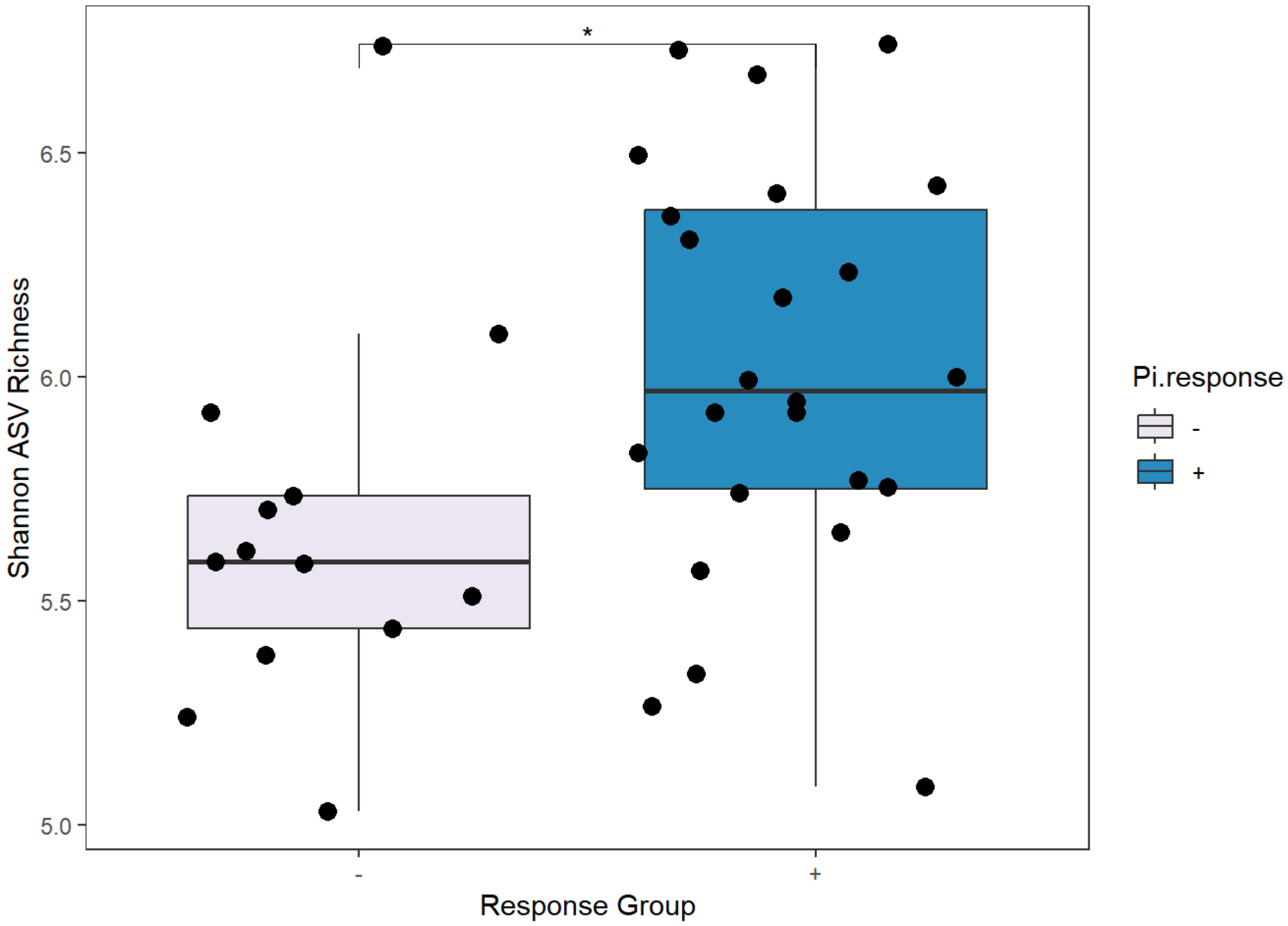

```
#Figure S10B
#2. Observed Index - Pi.response

# Transform Pi.response to a factor
datar16S_root3$Pi.response <- as.factor(datar16S_root3$Pi.response)

# Perform Dunn's test for Observed ASV richness by Pi.response
res_obs <- dunn_test(Observed ~ Pi.response, data = datar16S_root3, p.adjust.method =
"fdr")
stat.test <- res_obs %>% add_xy_position(x = "Pi.response")

# Plot Observed ASV richness across Pi.response groups
plot_16S_Observed_b <- ggplot(datar16S_root3, aes(Pi.response, Observed)) +
  geom_boxplot(aes(fill = Pi.response), show.legend = TRUE, outlier.shape = NA) +
  geom_jitter(size = 3) +  # Add jittered points for better visualization
  labs(x = "Response Group", y = "Observed ASV Richness") +  # Axis labels
  theme_bw() +  # Clean theme
  theme(panel.grid.major = element_blank(), panel.grid.minor = element_blank()) +  #
Remove grid lines
  stat_pvalue_manual(stat.test, hide.ns = TRUE) +  # Add statistical p-values, hiding
non-significant results
  scale_fill_manual(values = c("#ece7f2", "#2b8cbe"))  # Custom color palette

# Display Observed ASV richness plot
plot_16S_Observed_b
```

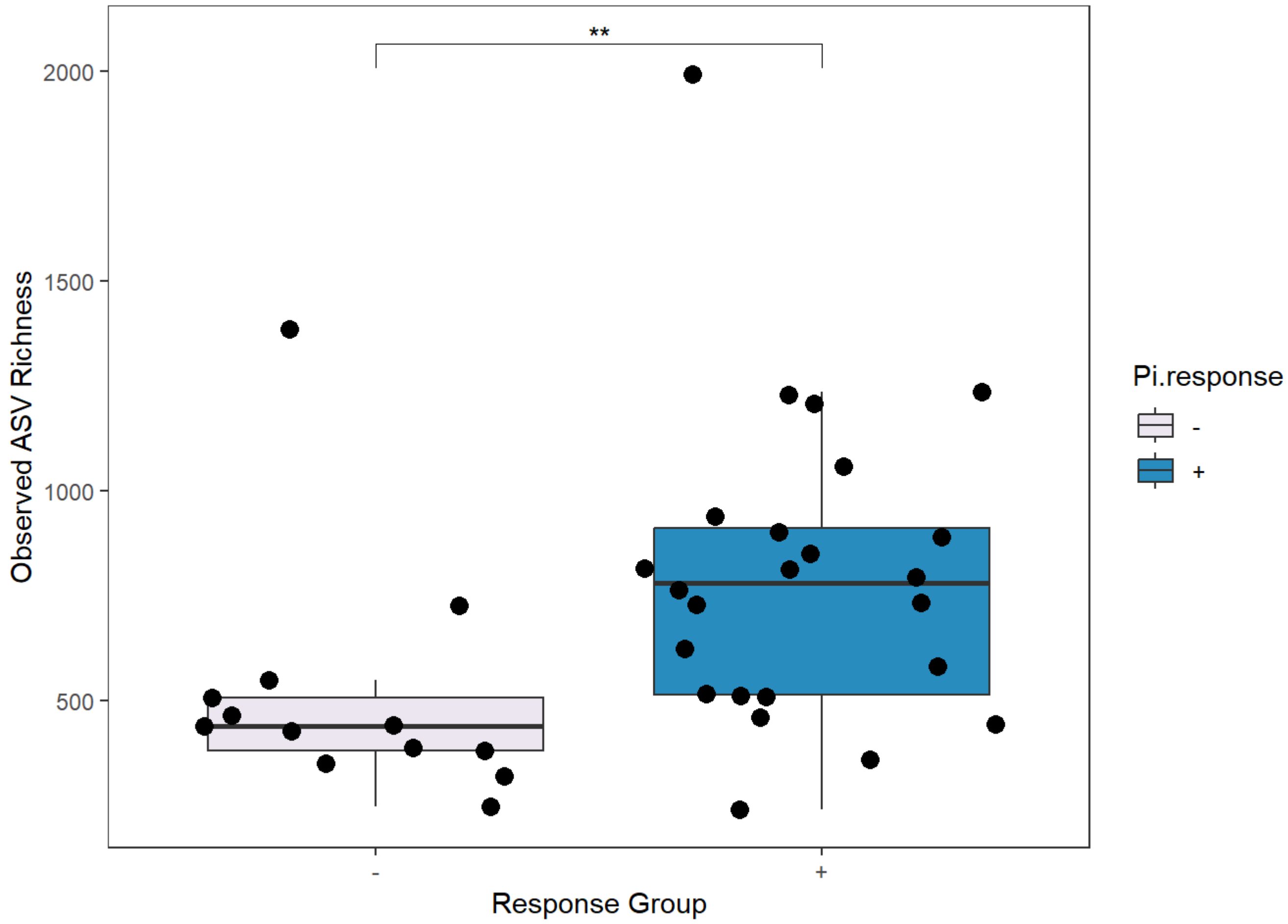

16.11 - Figure S10C - alpha diversity between Shoot response groups

```
library(ggplot2)
library(dplyr)

#1. Shannon Index - Shoot.response

# Filter data for treatment "T" and selected Shoot.response values
datar16S_root4 <- datar16S_root %>%
  filter(Treatment == "T") %>%
  filter(Shoot.response %in% c("-", "+")) %>%
  droplevels()

# Perform Dunn's test for Shannon ASV richness by Shoot.response
res_shan <- dunn_test(Shannon ~ Shoot.response, data = datar16S_root4, p.adjust.metho
d = "fdr")
stat.test <- res_shan %>% add_xy_position(x = "Shoot.response")

# Plot Shannon Index across Shoot.response groups
plot_16S_Shannon_b <- ggplot(datar16S_root4, aes(Shoot.response, Shannon)) +
  geom_boxplot(aes(fill = Shoot.response), show.legend = TRUE, outlier.shape = NA) +
  geom_jitter(size = 3) +  # Add jittered points for better visualization
  labs(x = "Response Group", y = "Shannon ASV Richness") +  # Axis labels
  theme_bw() +  # Clean theme
  theme(panel.grid.major = element_blank(), panel.grid.minor = element_blank()) +  #
Remove grid lines
  stat_pvalue_manual(stat.test, hide.ns = TRUE) +  # Add statistical p-values, hiding
non-significant results
  scale_fill_manual(values = c("#e5f5f9", "#2ca25f"))  # Custom color palette

# Display Shannon Index plot
plot_16S_Shannon_b
```

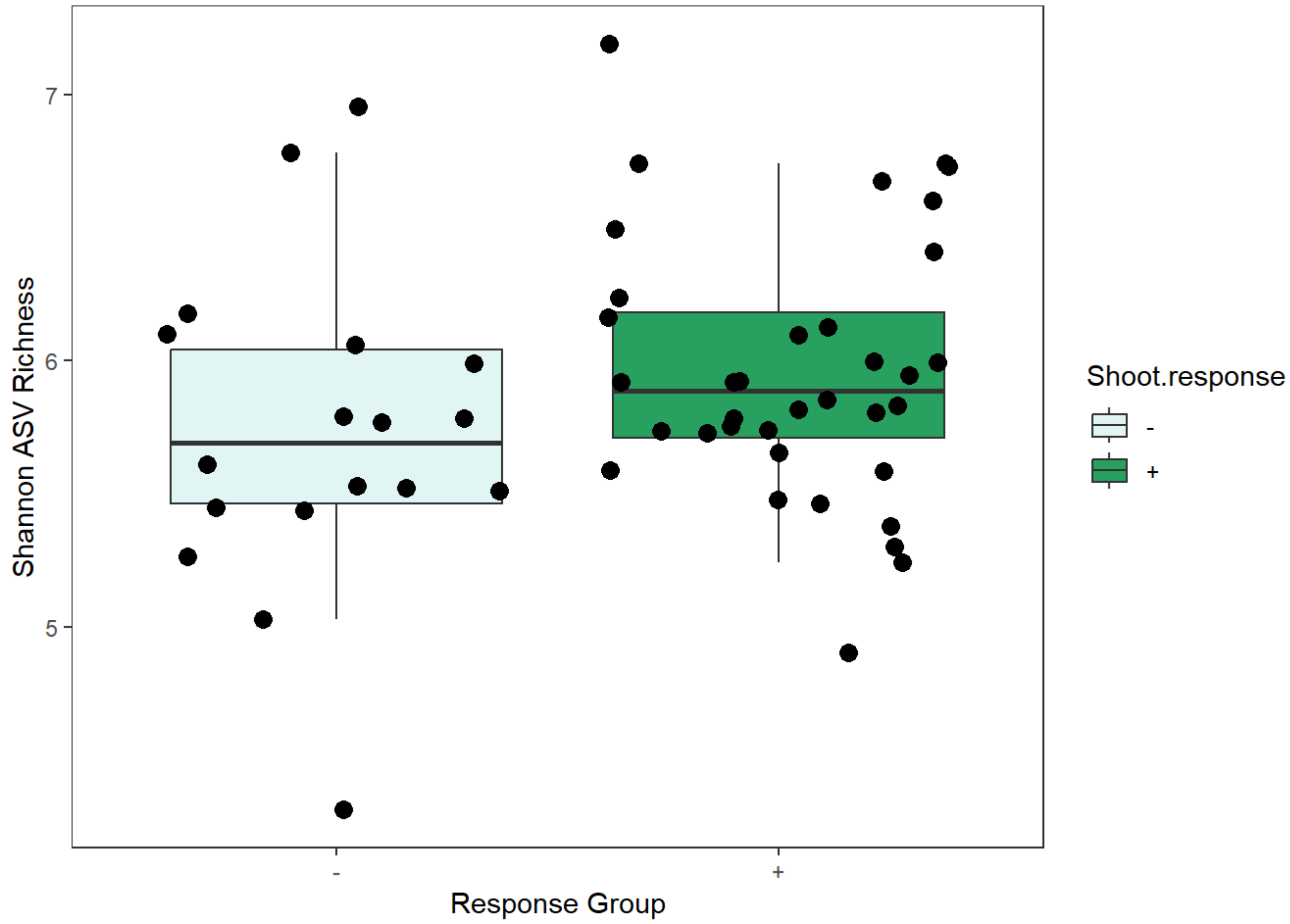


```
#2.Observed Index - Shoot.response

# Perform Dunn's test for Observed ASV richness by Shoot.response
res_obs <- dunn_test(Observed ~ Shoot.response, data = datar16S_root4, p.adjust.metho
d = "fdr")
stat.test <- res_obs %>% add_xy_position(x = "Shoot.response")

# Plot Observed ASV richness across Shoot.response groups
plot_16S_Observed_b <- ggplot(datar16S_root4, aes(Shoot.response, Observed)) +
  geom_boxplot(aes(fill = Shoot.response), show.legend = TRUE, outlier.shape = NA) +
  geom_jitter(size = 3) +  # Add jittered points for better visualization
  labs(x = "Response Group", y = "Observed ASV Richness") +  # Axis labels
  theme_bw() +  # Clean theme
  theme(panel.grid.major = element_blank(), panel.grid.minor = element_blank()) +  #
Remove grid lines
  stat_pvalue_manual(stat.test, hide.ns = TRUE) +  # Add statistical p-values, hiding
non-significant results
  scale_fill_manual(values = c("#e5f5f9", "#2ca25f"))  # Custom color palette

# Display Observed ASV richness plot
plot_16S_Observed_b
```

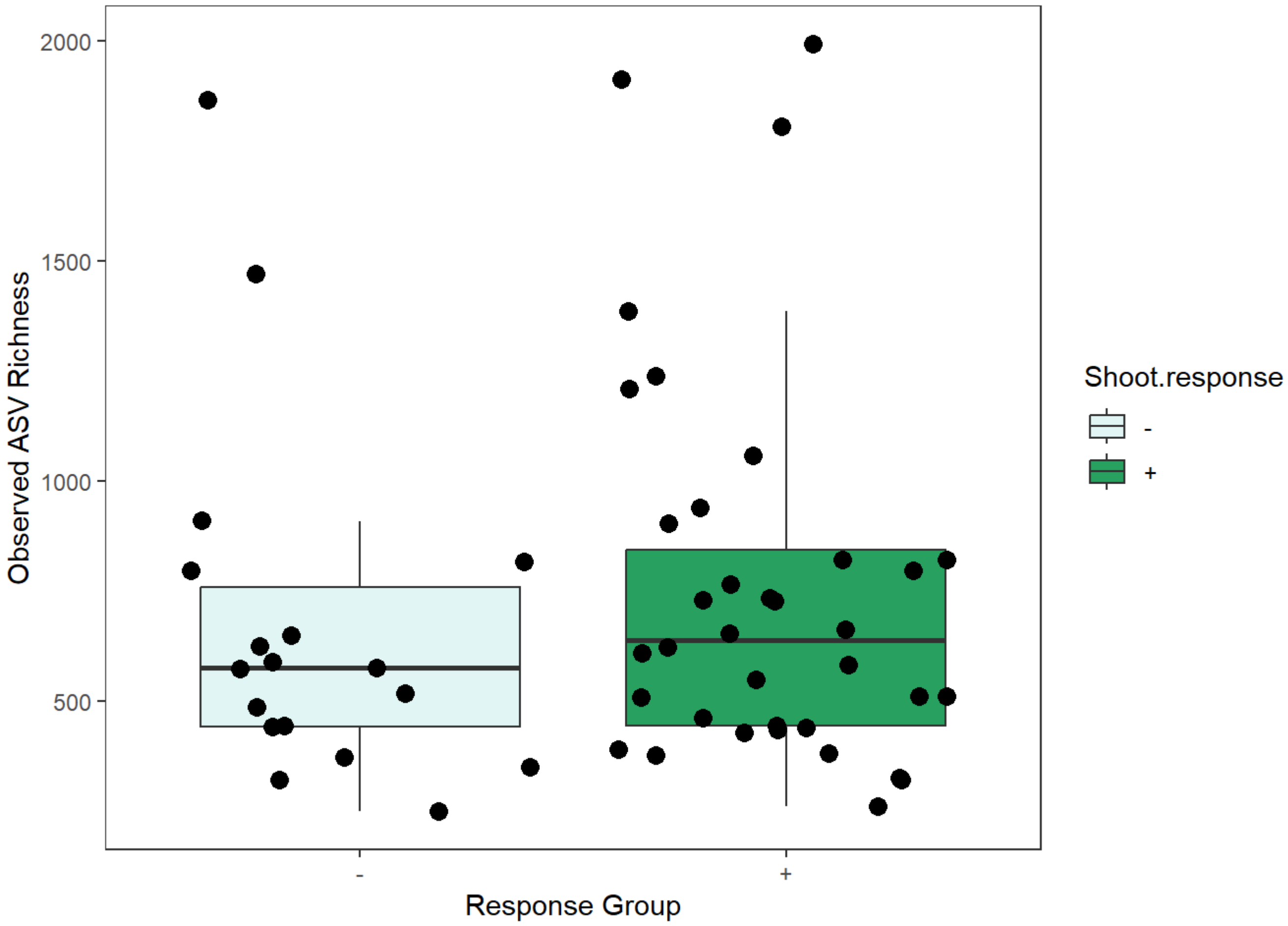

16.12 - Figure 5F - Figure S10D - alpha diversity between Root biomass response groups

```
############# Data Filtering and Preparation

# Filter data for Treatment "T" and exclude genotypes 71 and 495
datar16S_root_pheno <- datar16S_root %>%
  filter(Treatment == "T") %>%
  filter(genotype != "71" & genotype != "495") %>%
  droplevels()

# Define interval_ratioroot based on ratioroot values
datar16S_root_pheno$interval_ratioroot <- as.factor(
  ifelse(datar16S_root_pheno$ratioroot < 0.80, "-",
         ifelse(datar16S_root_pheno$ratioroot > 1.20, "+", "/"))
)

# Check if the factor levels are correct
levels(datar16S_root_pheno$interval_ratioroot)
```

```
## [1] "-" "/" "+"
```

```
############# Select Rows for Specific Root Response Groups

# Select rows with interval_ratioroot in ("-", "+")
datar16S_root_response <- datar16S_root_pheno %>%
  filter(interval_ratioroot %in% c("-", "+")) %>%
  droplevels()

############# 1. Shannon Index - Dunn's Test

# Perform Dunn's test for Shannon ASV richness across interval_ratioroot groups
res_obs_shannon <- dunn_test(Shannon ~ interval_ratioroot,
                             data = datar16S_root_response,
                             p.adjust.method = "fdr")

# Extract statistical results for annotation
stat.test_shannon <- res_obs_shannon %>% add_xy_position(x = "interval_ratioroot")

# Create plot for Shannon ASV richness
plot_16S_Shannon_b <- ggplot(datar16S_root_response, aes(interval_ratioroot, Shanno
n)) +
  geom_boxplot(aes(fill = interval_ratioroot),
               show.legend = TRUE,
               outlier.shape = NA) +  # Hide outliers
  geom_jitter(size = 3) +  # Add jittered points for better visualization
  labs(x = "Root Response Group", y = "Shannon ASV Richness") +  # Axis labels
  theme_bw() +  # Clean theme
  theme(panel.grid.major = element_blank(),
        panel.grid.minor = element_blank()) +  # Remove grid lines
  stat_pvalue_manual(stat.test_shannon, hide.ns = TRUE) +  # Add p-values
  scale_fill_manual(values = c("-" = "#f5ebe0",
                               "+" = "#8b5e34"))  # Custom color palette

# Display the Shannon plot
plot_16S_Shannon_b
```

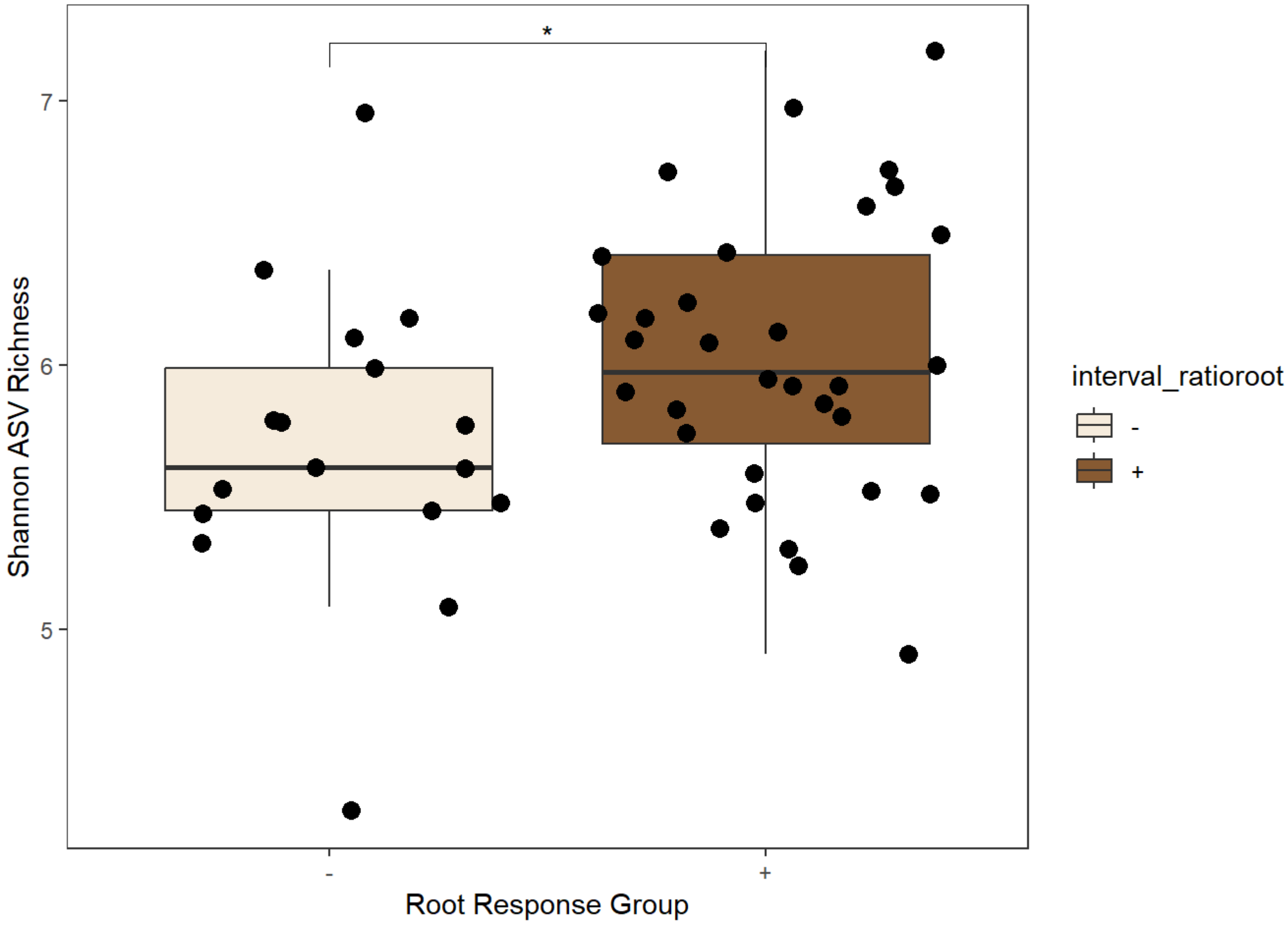


```
############# 2. Observed Index - Dunn's Test

# Perform Dunn's test for Observed ASV richness across interval_ratioroot groups
res_obs_observed <- dunn_test(Observed ~ interval_ratioroot,
                              data = datar16S_root_response,
                              p.adjust.method = "fdr")

# Extract statistical results for annotation
stat.test_observed <- res_obs_observed %>% add_xy_position(x = "interval_ratioroot")

# Create plot for Observed ASV richness
plot_16S_Observed_b <- ggplot(datar16S_root_response, aes(interval_ratioroot, Observe
d)) +
  geom_boxplot(aes(fill = interval_ratioroot),
               show.legend = TRUE,
               outlier.shape = NA) +  # Hide outliers
  geom_jitter(size = 3) +  # Add jittered points for better visualization
  labs(x = "Root Response Group", y = "Observed ASV Richness") +  # Axis labels
  theme_bw() +  # Clean theme
  theme(panel.grid.major = element_blank(),
        panel.grid.minor = element_blank()) +  # Remove grid lines
  stat_pvalue_manual(stat.test_observed, hide.ns = TRUE) +  # Add p-values
  scale_fill_manual(values = c("-" = "#f5ebe0",
                               "+" = "#8b5e34"))  # Custom color palette

# Display the Observed plot
plot_16S_Observed_b
```

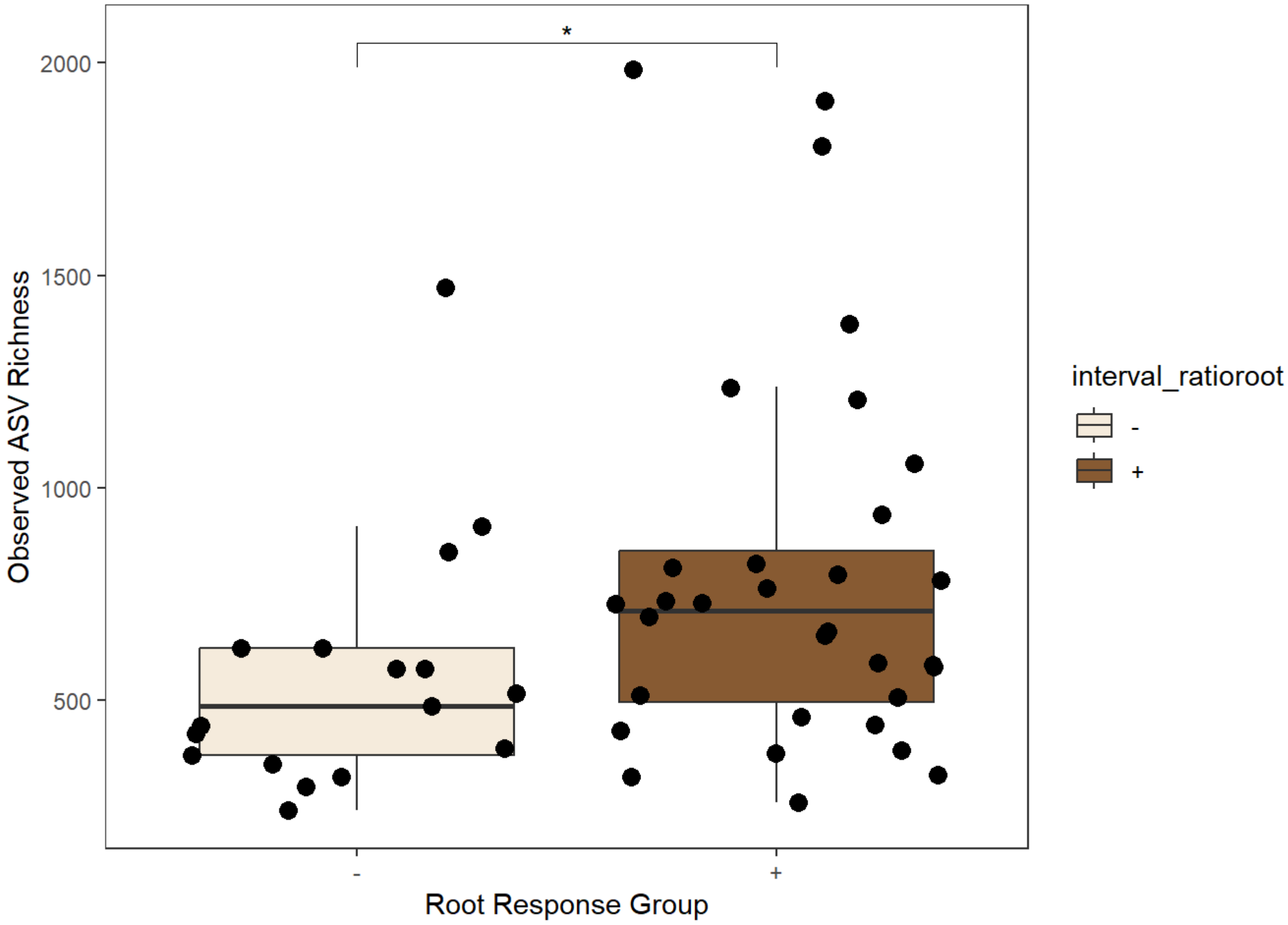

16.13 - Figure S10E - alpha diversity between Shoot biomass/root biomass response groups

```
# Data Transformation: Ensure the variables are numeric
datar16S_root_pheno <- datar16S_root %>%
  filter(Treatment == "T") %>%
  filter(genotype != "71" & genotype != "495") %>%
  droplevels()

# Transforming variables into numeric
datar16S_root_pheno$ratioS.R <- as.numeric(datar16S_root_pheno$ratioS.R)

# Create interval for 'ratioS.R'
datar16S_root_pheno$interval_ratioS.R <- as.factor(ifelse(datar16S_root_pheno$ratioS.
R < 0.80, "-",
                                                                 ifelse(datar16S_root_pheno
$ratioS.R > 1.20, "+", "/")))

# Select rows where 'interval_ratioS.R' is in ("-", "+")
datar16S_S.R_response <- datar16S_root_pheno %>%
  filter(interval_ratioS.R %in% c("-", "+"))

############# 1. Shannon Index - Dunn's Test

# Dunn's test for Shannon ASV richness
res_obs_shannon <- dunn_test(Shannon ~ interval_ratioS.R,
                             data = datar16S_S.R_response,
                             p.adjust.method = "fdr")

# Extract statistical results for annotation
stat.test_shannon <- res_obs_shannon %>% add_xy_position(x = "interval_ratioS.R")

# Create Shannon ASV richness plot
plot_16S_Shannon_b <- ggplot(datar16S_S.R_response, aes(interval_ratioS.R, Shannon))
+
  geom_boxplot(aes(fill = interval_ratioS.R),
               show.legend = TRUE,
               outlier.shape = NA) +  # Hide outliers
  geom_jitter(size = 3) +  # Add jittered points
  labs(x = "Root Response Group", y = "Shannon ASV Richness") +  # Axis labels
  theme_bw() +  # Clean theme
  theme(panel.grid.major = element_blank(),
        panel.grid.minor = element_blank()) +  # Remove grid lines
  stat_pvalue_manual(stat.test_shannon, hide.ns = TRUE) +  # Add p-values
  scale_fill_manual(values = c("-" = "#e6ece5",
                               "+" = "#556b2f",
                               "/" = "#9caf91"))  # Custom color palette

# Display the Shannon plot
plot_16S_Shannon_b
```

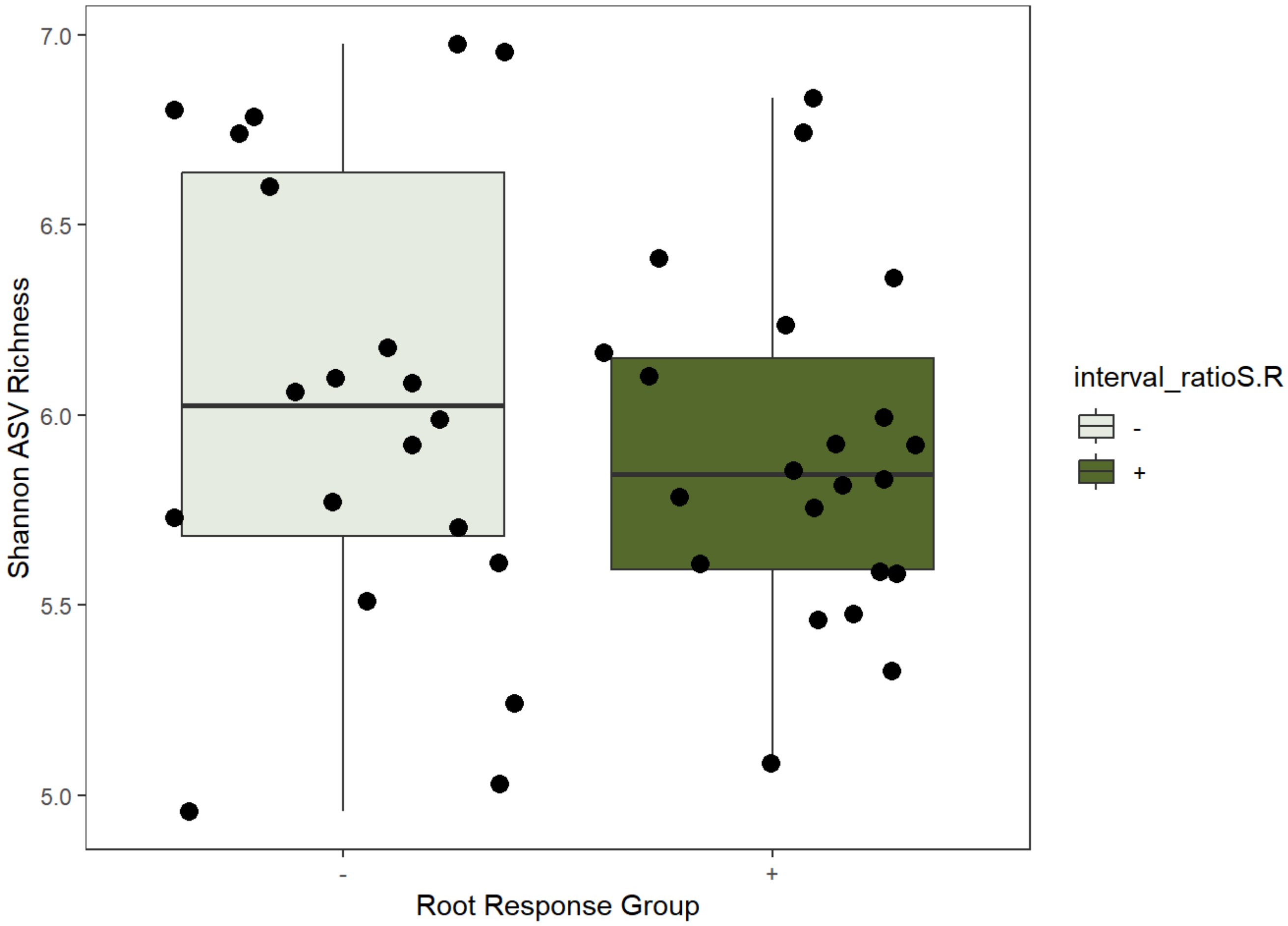


```
############# 2. Observed Index - Dunn's Test

# Dunn's test for Observed ASV richness
res_obs_observed <- dunn_test(Observed ~ interval_ratioS.R,
                              data = datar16S_S.R_response,
                              p.adjust.method = "fdr")

# Extract statistical results for annotation
stat.test_observed <- res_obs_observed %>% add_xy_position(x = "interval_ratioS.R")

# Create Observed ASV richness plot
plot_16S_Observed_b <- ggplot(datar16S_S.R_response, aes(interval_ratioS.R, Observe
d)) +
  geom_boxplot(aes(fill = interval_ratioS.R),
               show.legend = TRUE,
               outlier.shape = NA) +  # Hide outliers
  geom_jitter(size = 3) +  # Add jittered points
  labs(x = "Root Response Group", y = "Observed ASV Richness") +  # Axis labels
  theme_bw() +  # Clean theme
  theme(panel.grid.major = element_blank(),
        panel.grid.minor = element_blank()) +  # Remove grid lines
  stat_pvalue_manual(stat.test_observed, hide.ns = TRUE) +  # Add p-values
  scale_fill_manual(values = c("-" = "#e6ece5",
                               "+" = "#556b2f",
                               "/" = "#9caf91"))  # Custom color palette

# Display the Observed plot
plot_16S_Observed_b
```

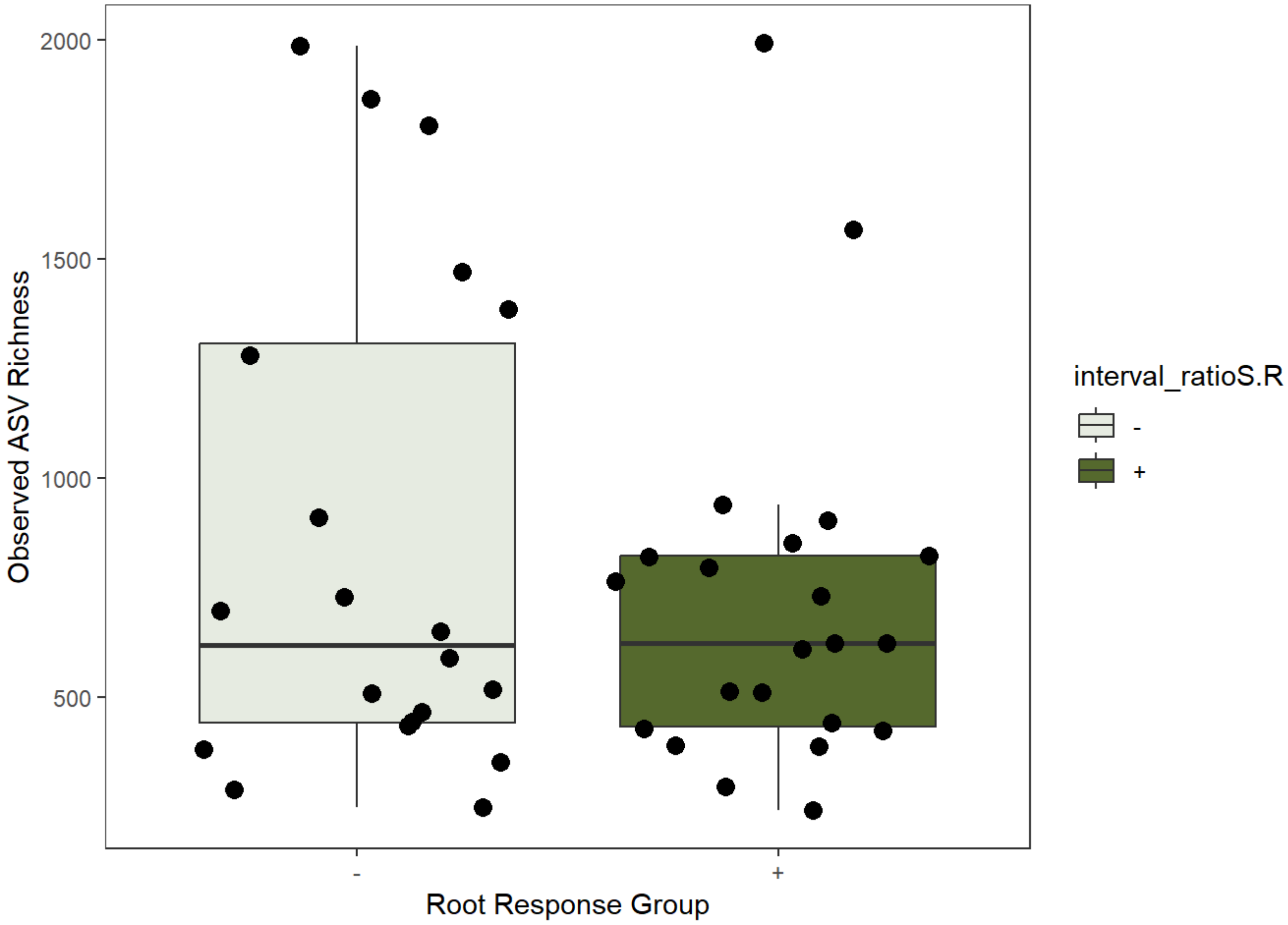

16.14 - Beta diversity across interval response groups

```
r16S_root1= subset_samples(r16S, phenotypic_group == "1"|phenotypic_group == "2"|phen
otypic_group == "3"|phenotypic_group == "4")
r16S_root1 <- subset_taxa(r16S_root1, !taxa_sums(r16S_root1) == 0)

sample_data(r16S_root1)$phenotypic_group<-as.factor(sample_data(r16S_root1)$phenotypi
c_group)

bray.16S <- phyloseq::distance(r16S_root1, method = "bray")
mdat.16Sb <- as(sample_data(r16S_root1), "data.frame")

adonis.16Sbray <- adonis2(bray.16S ~ phenotypic_group,
                                data = mdat.16Sb, perm = 20000)

print(adonis.16Sbray)
```

```
## Permutation test for adonis under reduced model
## Permutation: free
## Number of permutations: 20000
##
## adonis2(formula = bray.16S ~ phenotypic_group, data = mdat.16Sb, permutations = 20
000)
##          Df SumOfSqs      R2      F Pr(>F)
## Model     3   1.0778 0.03981 1.0088 0.3902
## Residual 73  25.9984 0.96019
## Total    76  27.0762 1.00000
```

16.15 - Beta diversity across interval response groups

```
r16S_root2= subset_samples(r16S, interval_response == "--"|interval_response == "++")
r16S_root2 <- subset_taxa(r16S_root2, !taxa_sums(r16S_root2) == 0)

sample_data(r16S_root2)$interval_response<-as.factor(sample_data(r16S_root2)$interval
_response)

bray.16S <- phyloseq::distance(r16S_root2, method = "bray")
mdat.16Sb <- as(sample_data(r16S_root2), "data.frame")

adonis.16Sbray <- adonis2(bray.16S ~ interval_response,
                                data = mdat.16Sb, perm = 20000)
print(adonis.16Sbray)
```

```
## Permutation test for adonis under reduced model
## Permutation: free
## Number of permutations: 20000
##
## adonis2(formula = bray.16S ~ interval_response, data = mdat.16Sb, permutations = 2
0000)
##          Df SumOfSqs      R2      F  Pr(>F)
## Model     1   0.4343 0.06905 1.2608 0.05485 .
## Residual 17   5.8555 0.93095
## Total    18   6.2898 1.00000
## ---
## Signif. codes:  0 '***' 0.001 '**' 0.01 '*' 0.05 '.' 0.1 ' ' 1
```

16.16 - Beta diversity across Pi response groups

```
# 1. ANOVA

# Subset the samples based on the 'Pi.response' variable being either "-" or "+"
r16S_root3 <- subset_samples(r16S, Pi.response == "-" | Pi.response == "+")

# Remove taxa with zero abundance
r16S_root3 <- subset_taxa(r16S_root3, !taxa_sums(r16S_root3) == 0)

# Convert 'Pi.response' and 'variety' to factors
sample_data(r16S_root3)$Pi.response <- as.factor(sample_data(r16S_root3)$Pi.response)
sample_data(r16S_root3)$variety <- as.factor(sample_data(r16S_root3)$variety)

# Calculate Bray-Curtis dissimilarity
bray.16S <- phyloseq::distance(r16S_root3, method = "bray")

# Extract metadata for use in the ANOVA test
mdat.16Sb <- as(sample_data(r16S_root3), "data.frame")

# Perform the PERMANOVA (adonis2) test using Bray-Curtis dissimilarity
adonis.16Sbray <- adonis2(bray.16S ~ Pi.response,
                          data = mdat.16Sb, perm = 20000)

# Print the results of the PERMANOVA test
print(adonis.16Sbray)
```

```
## Permutation test for adonis under reduced model
## Permutation: free
## Number of permutations: 20000
##
## adonis2(formula = bray.16S ~ Pi.response, data = mdat.16Sb, permutations = 20000)
##          Df SumOfSqs      R2    F Pr(>F)
## Model     1   0.4396 0.03422 1.24 0.0546 .
## Residual 35  12.4093 0.96578
## Total    36  12.8489 1.00000
## ---
## Signif. codes:  0 '***' 0.001 '**' 0.01 '*' 0.05 '.' 0.1 ' ' 1
```

```
# 2. Plot

# Load the ggrepel package for improved labeling in the plot
library(ggrepel)

# Perform Principal Coordinate Analysis (PCoA) based on Bray-Curtis dissimilarity
bray.pcoa.16S_s <- ordinate(r16S_root3, "PCoA", "bray")

# Define a custom color palette for the 'Pi.response' groups
palette_custom <- c("-" = "#ece7f2", "+" = "#2b8cbe")

# Create the PCoA plot with customized aesthetics
i.bray.pcoa.16S <- plot_ordination(r16S_root3,
                                   bray.pcoa.16S_s,
                                   type = "samples",
                                   color = "Pi.response") +
  stat_ellipse(aes(group = Pi.response), color = "black") +  # Add ellipses for each
group
  geom_point(size = 5, shape = 19, stroke = 0.5, aes(color = Pi.response), fill = NA,
color = "black") +  # Points with black border
  geom_point(size = 4.5, aes(color = Pi.response)) +  # Slightly smaller colored poin
ts
  ggtitle("16S rRNA - Bray-Curtis PCoA") +  # Add a title to the plot
  scale_color_manual(values = palette_custom) +  # Apply custom color palette
  theme_bw() +  # Use a black-and-white theme
  theme(panel.grid.major = element_blank(),  # Remove major grid lines
        panel.grid.minor = element_blank(),  # Remove minor grid lines
        panel.background = element_blank())  # Remove background color

# Display the final PCoA plot
i.bray.pcoa.16S
```

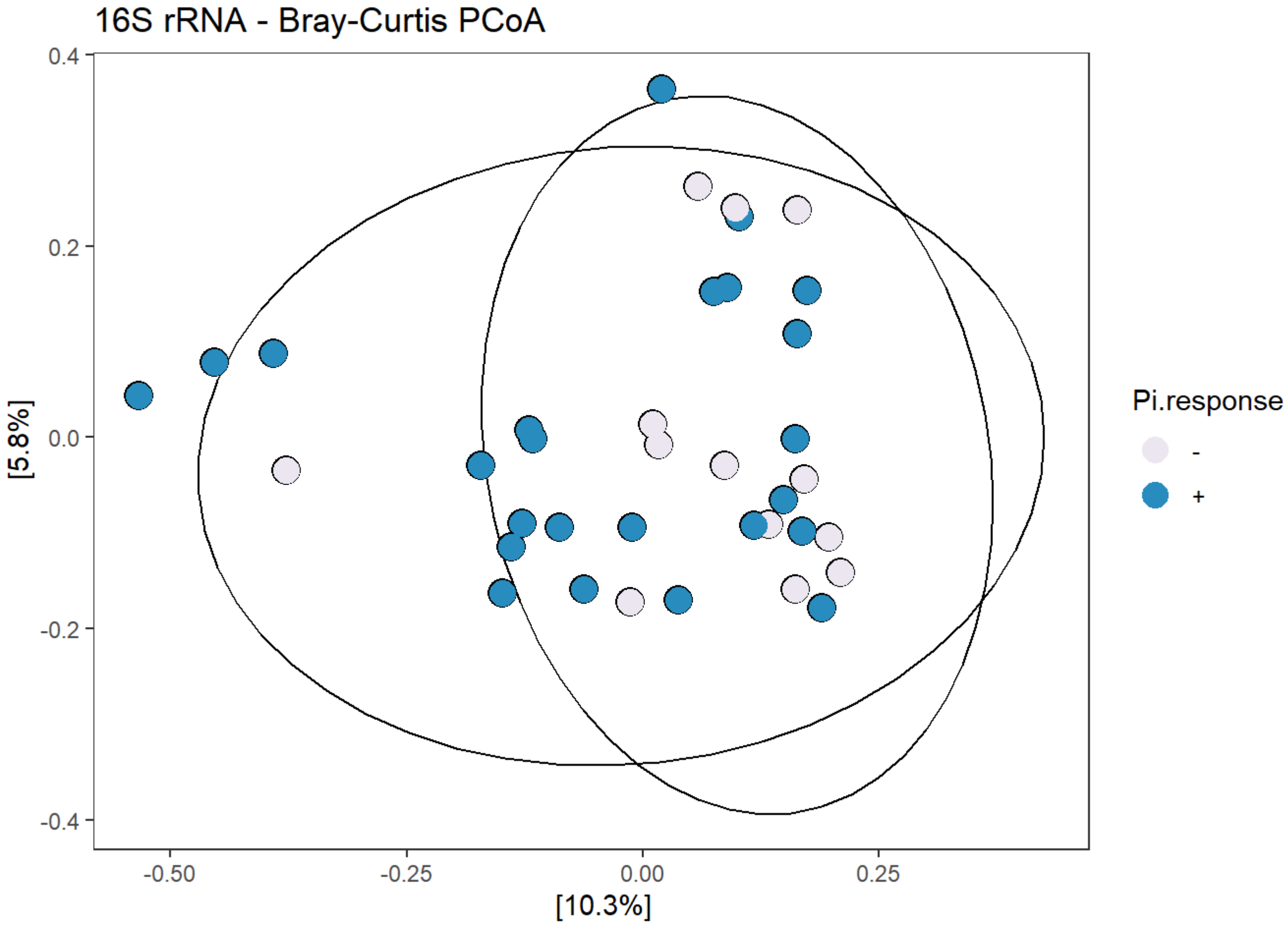

16.17 - Beta diversity across Shoot response groups

```
# 1. PERMANOVA

# Subset the samples based on the 'Shoot.response' variable being either "-" or "+"
r16S_root4 <- subset_samples(r16S, Shoot.response == "-" | Shoot.response == "+")

# Convert 'Shoot.response' to a factor
sample_data(r16S_root4)$Shoot.response <- as.factor(sample_data(r16S_root4)$Shoot.res
ponse)

# Calculate Bray-Curtis dissimilarity
bray.16S <- phyloseq::distance(r16S_root4, method = "bray")

# Extract metadata for use in the PERMANOVA test
mdat.16Sb <- as(sample_data(r16S_root4), "data.frame")

# Check the levels of 'Shoot.response'
levels(sample_data(r16S_root4)$Shoot.response)
```

```
## [1] "-" "+"
```

```
# Perform the PERMANOVA (adonis2) test using Bray-Curtis dissimilarity
adonis.16Sbray <- adonis2(bray.16S ~ Shoot.response,
                          data = mdat.16Sb, perm = 10000)

# Print the results of the PERMANOVA test
print(adonis.16Sbray)
```

```
## Permutation test for adonis under reduced model
## Permutation: free
## Number of permutations: 10000
##
## adonis2(formula = bray.16S ~ Shoot.response, data = mdat.16Sb, permutations = 1000
0)
##          Df SumOfSqs      R2      F Pr(>F)
## Model     1   0.3318 0.01751 0.9269 0.6355
## Residual 52  18.6137 0.98249
## Total    53  18.9454 1.00000
```

```
# 2. Plot

# Load the ggrepel package for improved labeling in the plot
library(ggrepel)

# Perform Principal Coordinate Analysis (PCoA) based on Bray-Curtis dissimilarity
bray.pcoa.16S_s <- ordinate(r16S_root4, "PCoA", "bray")

# Define a custom color palette for the 'Shoot.response' groups
palette_custom <- c("-" = "#e5f5f9", "+" = "#2ca25f")

# Create the PCoA plot with customized aesthetics
i.bray.pcoa.16S <- plot_ordination(r16S_root4,
                                   bray.pcoa.16S_s,
                                   type = "samples",
                                   color = "Shoot.response") +
  stat_ellipse(aes(group = Shoot.response), color = "black") +  # Add ellipses for ea
ch group
  geom_point(size = 5, shape = 19, stroke = 0.5, aes(color = Shoot.response), fill =
NA, color = "black") +  # Points with black border
  geom_point(size = 4.5, aes(color = Shoot.response)) +  # Slightly smaller colored p
oints
  ggtitle("16S rRNA - Bray-Curtis PCoA") +  # Add a title to the plot
  scale_color_manual(values = palette_custom) +  # Apply custom color palette
  theme_bw() +  # Use a black-and-white theme
  theme(panel.grid.major = element_blank(),  # Remove major grid lines
        panel.grid.minor = element_blank(),  # Remove minor grid lines
        panel.background = element_blank())  # Remove background color

# Display the final PCoA plot
i.bray.pcoa.16S
```

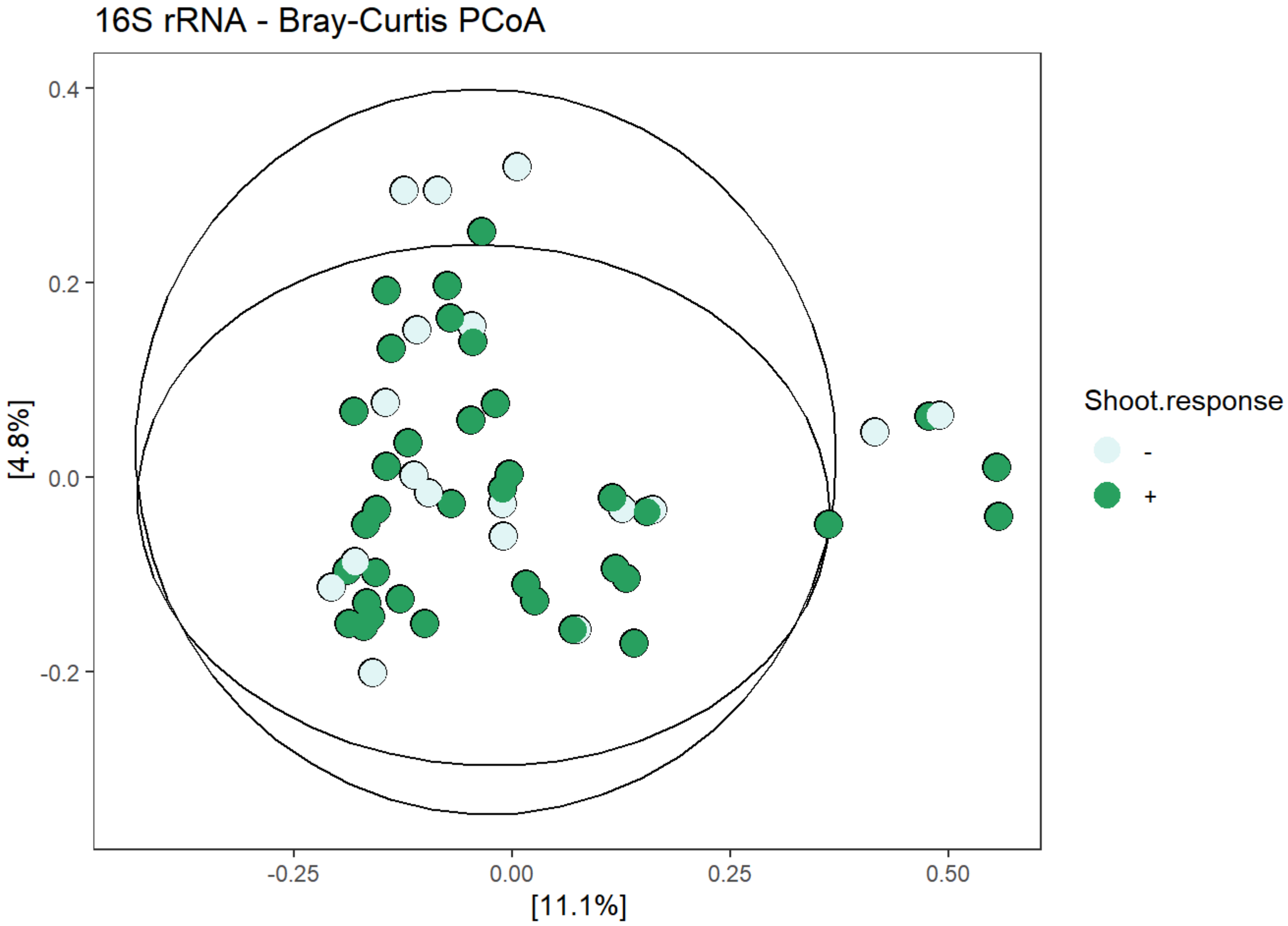

16.18 - Beta diversity across root response groups

```
# 1. PERMANOVA

r16S_ratioPi <- subset_samples(r16S, tissue %in% c("root") & !genotype %in% c("71",
"495"))
r16S_ratioPi <- subset_taxa(r16S_ratioPi, !taxa_sums(r16S_ratioPi) == 0)

# Add interval for root
sample_data(r16S_ratioPi)$interval_ratioroot <- as.factor(ifelse(sample_data(r16S_rat
ioPi)$ratioroot < 0.80, "-",
                                                                   ifelse(sample_data(r16S_ra
tioPi)$ratioroot > 1.20, "+", "/")))

r16S_root5 <- subset_samples(r16S_ratioPi, interval_ratioroot == "-" | interval_ratio
root == "+")

# Calculate Bray-Curtis dissimilarity
bray.16S <- phyloseq::distance(r16S_root5, method = "bray")
mdat.16Sb <- as(sample_data(r16S_root5), "data.frame")

mdat.16Sb$ratioroot <- as.numeric(mdat.16Sb$ratioroot)

# Perform PERMANOVA (adonis2) test
adonis.16Sbray <- adonis2(bray.16S ~ ratioroot,
                          data = mdat.16Sb, perm = 999)

# Print PERMANOVA results
print(adonis.16Sbray)
```

```
## Permutation test for adonis under reduced model
## Permutation: free
## Number of permutations: 999
##
## adonis2(formula = bray.16S ~ ratioroot, data = mdat.16Sb, permutations = 999)
##          Df SumOfSqs      R2      F Pr(>F)
## Model     1   0.5254 0.03045 1.4762  0.012 *
## Residual 47  16.7292 0.96955
## Total    48  17.2546 1.00000
## ---
## Signif. codes:  0 '***' 0.001 '**' 0.01 '*' 0.05 '.' 0.1 ' ' 1
```

```
# 2. Plot

library(ggrepel)

# Perform Principal Coordinate Analysis (PCoA) using Bray-Curtis dissimilarity
bray.pcoa.16S_s <- ordinate(r16S_root5, "PCoA", "bray")

# Define a custom color palette for the 'interval_ratioroot' groups
palette_custom <- c("-" = "#f5ebe0", "+" = "#8b5e34")

# Create the PCoA plot with customized aesthetics
i.bray.pcoa.16S <- plot_ordination(r16S_root5,
                                   bray.pcoa.16S_s,
                                   type = "samples",
                                   color = "interval_ratioroot") +
  stat_ellipse(aes(group = interval_ratioroot), color = "black") +  # Add ellipses fo
r each group
  geom_point(size = 5, shape = 19, stroke = 0.5, aes(color = interval_ratioroot), fil
l = NA, color = "black") +  # Points with black border
  geom_point(size = 4.5, aes(color = interval_ratioroot)) +  # Slightly smaller color
ed points
  ggtitle("16S rRNA - Bray-Curtis PCoA") +  # Add a title to the plot
  scale_color_manual(values = palette_custom) +  # Apply custom color palette
  theme_bw() +  # Use a black-and-white theme
  theme(panel.grid.major = element_blank(),  # Remove major grid lines
        panel.grid.minor = element_blank(),  # Remove minor grid lines
        panel.background = element_blank())  # Remove background color

# Display the final PCoA plot
i.bray.pcoa.16S
```

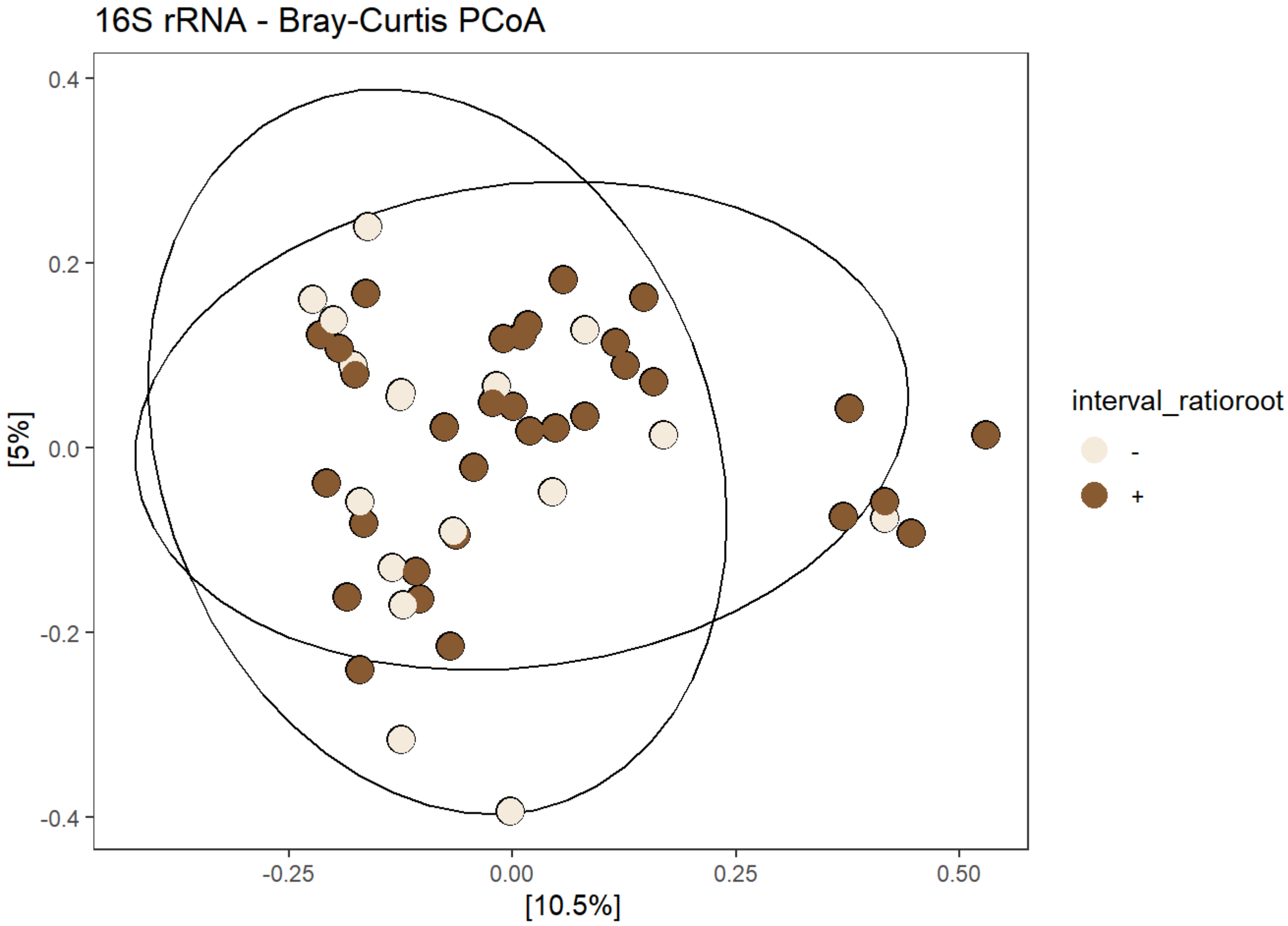

16.19 - Beta diversity across Shoot/root response groups

```
#1. PERMANOVA

## Subsetting samples to include only 'root' tissue and exclude specific genotypes (7
1 and 495)
r16S_ratioPi <- subset_samples(r16S, tissue %in% c("root") & !genotype %in% c("71",
"495"))

# Removing taxa with zero sums (i.e., taxa not present in any samples)
r16S_ratioPi <- subset_taxa(r16S_ratioPi, !taxa_sums(r16S_ratioPi) == 0)

# Creating an interval variable based on the ratioS.R value
# Assigning "-" if ratioS.R < 0.80, "+" if ratioS.R > 1.20, and "/" for values in bet
ween
sample_data(r16S_ratioPi)$interval_S.R_ratio <- as.factor(ifelse(sample_data(r16S_rat
ioPi)$ratioS.R < 0.80, "-",
                                                                  ifelse(sample_data(r16S_ra
tioPi)$ratioS.R > 1.20, "+", "/")))

# Subsetting the samples where the interval classification is either "-" or "+"
r16S_root6 <- subset_samples(r16S_ratioPi, interval_S.R_ratio == "-" | interval_S.R_r
atio == "+")

# Calculating the Bray-Curtis dissimilarity matrix for the subsetted samples
bray.16S <- phyloseq::distance(r16S_root6, method = "bray")

# Converting sample data to a data frame and ensuring ratioS.R is numeric for the ana
lysis
mdat.16Sb <- as(sample_data(r16S_root6), "data.frame")
mdat.16Sb$ratioS.R <- as.numeric(mdat.16Sb$ratioS.R)

# Running the PERMANOVA test (adonis2) with Bray-Curtis dissimilarity as the dependen
t variable and ratioS.R as the independent variable
# Using 999 permutations to test for significance
adonis.16Sbray <- adonis2(bray.16S ~ ratioS.R,
                          data = mdat.16Sb, perm = 999)

# Printing the results of the PERMANOVA test
print(adonis.16Sbray)
```

```
## Permutation test for adonis under reduced model
## Permutation: free
## Number of permutations: 999
##
## adonis2(formula = bray.16S ~ ratioS.R, data = mdat.16Sb, permutations = 999)
##          Df SumOfSqs      R2      F Pr(>F)
## Model     1   0.4439 0.03067 1.2655  0.072 .
## Residual 40  14.0311 0.96933
## Total    41  14.4750 1.00000
## ---
## Signif. codes:  0 '***' 0.001 '**' 0.01 '*' 0.05 '.' 0.1 ' ' 1
```

```
#2. PLOT

# Loading the 'ggrepel' package for improved plotting with text labels (if needed)
library(ggrepel)

# Performing Principal Coordinate Analysis (PCoA) based on Bray-Curtis dissimilarity
bray.pcoa.16S_s <- ordinate(r16S_root6, "PCoA", "bray")

# Defining a custom color palette for the three levels of 'interval_S.R_ratio'
# "-" will be represented by a light green color, "+" by a dark olive green
palette_custom <- c("-" = "#e6ece5", "+" = "#556b2f")

# Creating the PCoA plot with customized aesthetics
i.bray.pcoa.16S <- plot_ordination(r16S_root6,
                                   bray.pcoa.16S_s,
                                   type = "samples",  # Indicating that we're plottin
g sample data
                                   color = "interval_S.R_ratio") +  # Coloring the po
ints by the 'interval_S.R_ratio' factor
  stat_ellipse(aes(group = interval_S.R_ratio), color = "black") +  # Adding black el
lipses to indicate groupings
  geom_point(size = 5, shape = 19, stroke = 0.5, aes(color = interval_S.R_ratio), fil
l = NA, color = "black") +  # Adding large points with a black border
  geom_point(size = 4.5, aes(color = interval_S.R_ratio)) +  # Adding smaller colored
points for each sample
  ggtitle("16S rRNA - Bray-Curtis PCoA") +  # Adding a title to the plot
  scale_color_manual(values = palette_custom) +  # Applying the custom color palette
to the plot
  theme_bw() +  # Using a black-and-white theme for the plot
  theme(panel.grid.major = element_blank(),  # Removing major grid lines for a cleane
r look
        panel.grid.minor = element_blank(),  # Removing minor grid lines
        panel.background = element_blank())  # Removing the background color

# Displaying the final PCoA plot
i.bray.pcoa.16S
```

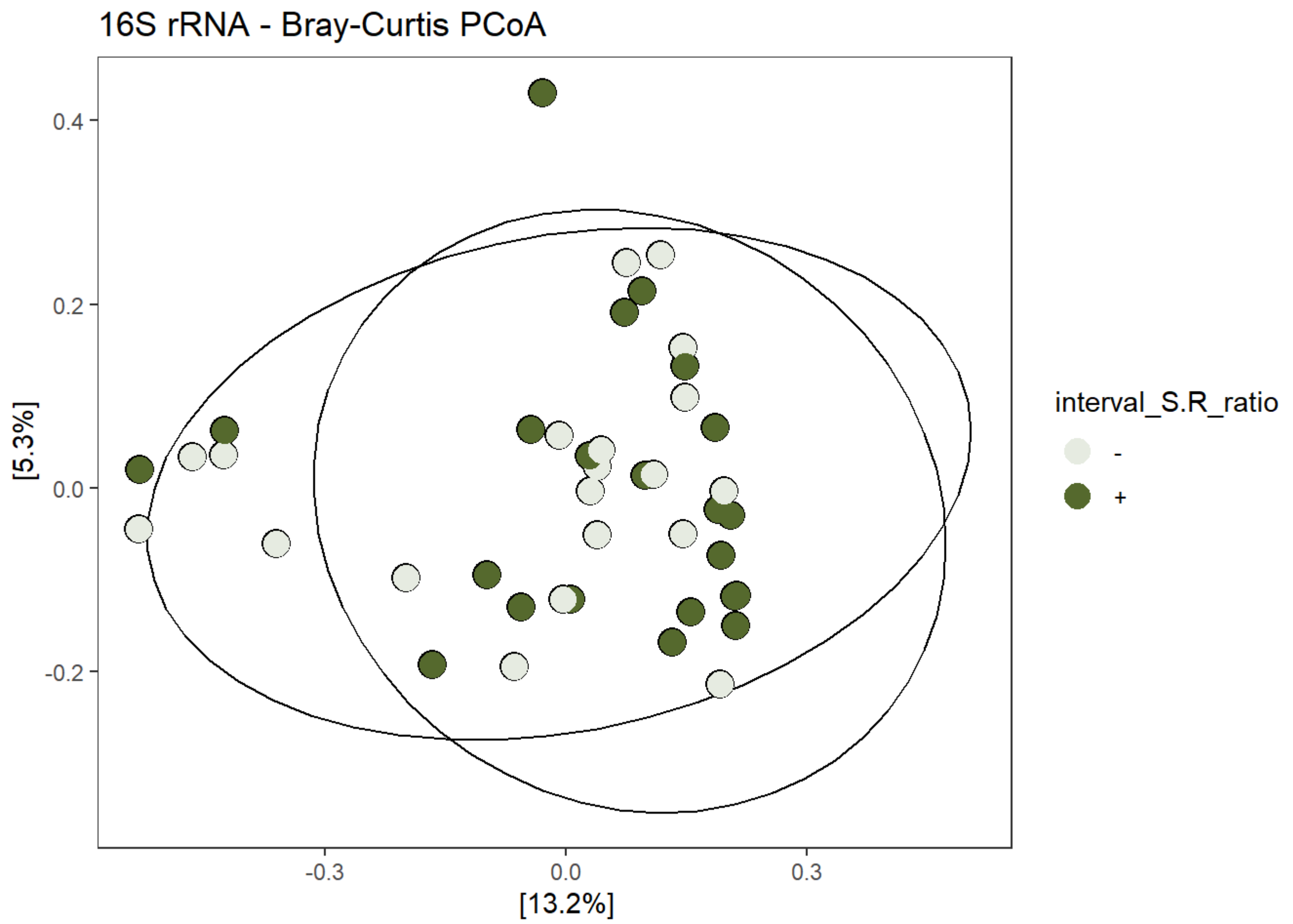

16.20 - Log2fold change between phosphate groups

```
# Load necessary libraries
library(DESeq2)
```

```
## Caricamento del pacchetto richiesto: S4Vectors
```

```
## Caricamento del pacchetto richiesto: stats4
```

```
## Caricamento del pacchetto richiesto: BiocGenerics
```

```
## Caricamento del pacchetto richiesto: generics
```

```
##
## Caricamento pacchetto: 'generics'
```

```
## Il seguente oggetto è mascherato da 'package:lubridate':
##
##     as.difftime
```

```
## Il seguente oggetto è mascherato da 'package:dplyr':
##
##     explain
```

```
## I seguenti oggetti sono mascherati da 'package:base':
##
##     as.difftime, as.factor, as.ordered, intersect, is.element, setdiff,
##     setequal, union
```

```
##
## Caricamento pacchetto: 'BiocGenerics'
```

```
## Il seguente oggetto è mascherato da 'package:dplyr':
##
##     combine
```

```
## I seguenti oggetti sono mascherati da 'package:stats':
##
##     IQR, mad, sd, var, xtabs
```

```
## I seguenti oggetti sono mascherati da 'package:base':
##
##     anyDuplicated, aperm, append, as.data.frame, basename, cbind,
##     colnames, dirname, do.call, duplicated, eval, evalq, Filter, Find,
##     get, grep, grepl, is.unsorted, lapply, Map, mapply, match, mget,
##     order, paste, pmax, pmax.int, pmin, pmin.int, Position, rank,
##     rbind, Reduce, rownames, sapply, saveRDS, table, tapply, unique,
##     unsplit, which.max, which.min
```

```
##
## Caricamento pacchetto: 'S4Vectors'
```

```
## I seguenti oggetti sono mascherati da 'package:data.table':
##
##     first, second
```

```
## I seguenti oggetti sono mascherati da 'package:lubridate':
##
##     second, second<-
```

```
## I seguenti oggetti sono mascherati da 'package:dplyr':
##
##     first, rename
```

```
## Il seguente oggetto è mascherato da 'package:tidyr':
##
##     expand
```

```
## Il seguente oggetto è mascherato da 'package:utils':
##
##     findMatches
```

```
## I seguenti oggetti sono mascherati da 'package:base':
##
##     expand.grid, I, unname
```

```
## Caricamento del pacchetto richiesto: IRanges
```

```
##
## Caricamento pacchetto: 'IRanges'
```

```
## Il seguente oggetto è mascherato da 'package:nlme':
##
##     collapse
```

```
## Il seguente oggetto è mascherato da 'package:data.table':
##
##     shift
```

```
## Il seguente oggetto è mascherato da 'package:phyloseq':
##
##     distance
```

```
## I seguenti oggetti sono mascherati da 'package:psych':
##
##     distance, reflect
```

```
## Il seguente oggetto è mascherato da 'package:ggbiplot':
##
##     reflect
```

```
## Il seguente oggetto è mascherato da 'package:rstatix':
##
##     desc
```

```
## Il seguente oggetto è mascherato da 'package:lubridate':
##
##     %within%
```

```
## I seguenti oggetti sono mascherati da 'package:dplyr':
##
##     collapse, desc, slice
```

```
## Il seguente oggetto è mascherato da 'package:purrr':
##
##     reduce
```

```
## Il seguente oggetto è mascherato da 'package:grDevices':
##
##     windows
```

```
## Caricamento del pacchetto richiesto: GenomicRanges
```

```
## Caricamento del pacchetto richiesto: GenomeInfoDb
```

```
## Caricamento del pacchetto richiesto: SummarizedExperiment
```

```
## Caricamento del pacchetto richiesto: MatrixGenerics
```

```
## Caricamento del pacchetto richiesto: matrixStats
```

```
##
## Caricamento pacchetto: 'matrixStats'
```

```
## Il seguente oggetto è mascherato da 'package:dplyr':
##
##     count
```

```
##
## Caricamento pacchetto: 'MatrixGenerics'
```

```
## I seguenti oggetti sono mascherati da 'package:matrixStats':
##
##     colAlls, colAnyNAs, colAnys, colAvgsPerRowSet, colCollapse,
##     colCounts, colCummaxs, colCummins, colCumprods, colCumsums,
##     colDiffs, colIQRDiffs, colIQRs, colLogSumExps, colMadDiffs,
##     colMads, colMaxs, colMeans2, colMedians, colMins, colOrderStats,
##     colProds, colQuantiles, colRanges, colRanks, colSdDiffs, colSds,
##     colSums2, colTabulates, colVarDiffs, colVars, colWeightedMads,
##     colWeightedMeans, colWeightedMedians, colWeightedSds,
##     colWeightedVars, rowAlls, rowAnyNAs, rowAnys, rowAvgsPerColSet,
##     rowCollapse, rowCounts, rowCummaxs, rowCummins, rowCumprods,
##     rowCumsums, rowDiffs, rowIQRDiffs, rowIQRs, rowLogSumExps,
##     rowMadDiffs, rowMads, rowMaxs, rowMeans2, rowMedians, rowMins,
##     rowOrderStats, rowProds, rowQuantiles, rowRanges, rowRanks,
##     rowSdDiffs, rowSds, rowSums2, rowTabulates, rowVarDiffs, rowVars,
##     rowWeightedMads, rowWeightedMeans, rowWeightedMedians,
##     rowWeightedSds, rowWeightedVars
```

```
## Caricamento del pacchetto richiesto: Biobase
```

```
## Welcome to Bioconductor
##
##     Vignettes contain introductory material; view with
##     'browseVignettes()'. To cite Bioconductor, see
##     'citation("Biobase")', and for packages 'citation("pkgname")'.
```

```
##
## Caricamento pacchetto: 'Biobase'
```

```
## Il seguente oggetto è mascherato da 'package:MatrixGenerics':
##
##     rowMedians
```

```
## I seguenti oggetti sono mascherati da 'package:matrixStats':
##
##     anyMissing, rowMedians
```

```
## Il seguente oggetto è mascherato da 'package:phyloseq':
##
##     sampleNames
```

```
library(ggplot2)
library(tidyverse)

########################################### Phosphate Response Analysis

# Subset the dataset for phosphate treatment "T" and exclude empty or "/" responses f
or Pi.response
phosphate_data <- subset_samples(r16S, Treatment == "T")
phosphate_data <- subset_samples(phosphate_data, Pi.response != "/" & Pi.response !=
"")

# Remove taxa with zero sums
phosphate_data <- subset_taxa(phosphate_data, !taxa_sums(phosphate_data) == 0)

# Create a binary response variable based on Pi.response
sample_data(phosphate_data)$Pi.response_binary <-
  ifelse(sample_data(phosphate_data)$Pi.response %in% c("+"), "high", "low")

# Convert the binary response variable into a factor
sample_data(phosphate_data)$Pi.response_binary <- as.factor(sample_data(phosphate_dat
a)$Pi.response_binary)

# Check the data structure
levels(sample_data(phosphate_data)$Pi.response_binary)
```

```
## [1] "high" "low"
```

```
# Differential abundance analysis using DESeq2
diagdds_phosphate <- phyloseq_to_deseq2(phosphate_data, ~ Pi.response_binary)
```

```
## converting counts to integer mode
```

```
# Define the geometric mean function to estimate size factors
gm_mean <- function(x, na.rm = TRUE) {
  exp(sum(log(x[x > 0]), na.rm = na.rm) / length(x))
}

# Calculate geometric means and estimate size factors
geoMeans <- apply(counts(diagdds_phosphate), 1, gm_mean)
diagdds_phosphate <- estimateSizeFactors(diagdds_phosphate, geoMeans = geoMeans)

# Perform DESeq2 analysis with local fit
diagdds_phosphate <- DESeq(diagdds_phosphate, fitType = "local")
```

```
## using pre-existing size factors
```

```
## estimating dispersions
```

```
## gene-wise dispersion estimates
```

```
## mean-dispersion relationship
```

```
## final dispersion estimates
```

```
## fitting model and testing
```

```
## -- replacing outliers and refitting for 12546 genes
## -- DESeq argument 'minReplicatesForReplace' = 7
## -- original counts are preserved in counts(dds)
```

```
## estimating dispersions
```

```
## fitting model and testing
```

```
# Extract results for high vs low phosphate response
res_phosphate <- results(diagdds_phosphate, cooksCutoff = FALSE, contrast = c("Pi.res
ponse_binary", "high", "low"))

# Order results by adjusted p-value
res_phosphate <- res_phosphate[order(res_phosphate$padj, na.last = NA), ]

# Display the top results
head(res_phosphate)
```

```
## log2 fold change (MLE): Pi.response_binary high vs low
## Wald test p-value: Pi.response_binary high vs low
## DataFrame with 6 rows and 6 columns
##         baseMean log2FoldChange     lfcSE      stat      pvalue        padj
##        <numeric>      <numeric> <numeric> <numeric>   <numeric>   <numeric>
## ASV240   27.3366        24.3816   1.96743  12.39257 2.86692e-35 4.14270e-32
## ASV318   24.2357        24.2158   2.25863  10.72148 8.07026e-27 5.83076e-24
## ASV292   34.4075        24.6558   2.33481  10.56006 4.56383e-26 2.19824e-23
## ASV392   17.3552        23.7536   2.46434   9.63893 5.47538e-22 1.97798e-19
## ASV225   34.4243        24.6944   2.58544   9.55132 1.28056e-21 3.08401e-19
## ASV438   19.2256        23.8961   2.50070   9.55575 1.22687e-21 3.08401e-19
```

```
# Set significance threshold (alpha = 0.05) for results
alpha <- 0.05
sigtab_phosphate <- res_phosphate[which(res_phosphate$padj < alpha), ]

# Combine the results with taxonomic information
sigtab_phosphate <- cbind(as(sigtab_phosphate, "data.frame"), as(tax_table(phosphate_
data)[rownames(sigtab_phosphate), ], "matrix"))

# Display dimensions and top results of the significant taxa
dimnames(sigtab_phosphate)
```

```
## [[1]]
##  [1] "ASV240" "ASV318" "ASV292" "ASV392" "ASV225" "ASV438" "ASV887" "ASV294"
##  [9] "ASV298" "ASV201" "ASV950" "ASV727" "ASV491" "ASV680" "ASV132" "ASV215"
## [17] "ASV274" "ASV95"  "ASV451" "ASV133" "ASV186" "ASV711"
##
## [[2]]
##  [1] "baseMean"       "log2FoldChange" "lfcSE"          "stat"
##  [5] "pvalue"         "padj"           "Kingdom"        "Phylum"
##  [9] "Class"          "Order"          "Family"         "Genus"
```

```
head(sigtab_phosphate)
```

	baseM... <dbl>	log2FoldChange <dbl>	lfcSE <dbl>	stat <dbl>	pvalue <dbl>	padj <dbl>	Kingdor <chr>
ASV240	27.33658	24.38157	1.967434	12.392574	2.866919e-35	4.142699e-32	Bacteria
ASV318	24.23573	24.21581	2.258626	10.721479	8.070263e-27	5.830765e-24	Bacteria
ASV292	34.40748	24.65575	2.334812	10.560057	4.563827e-26	2.198243e-23	Bacteria
ASV392	17.35516	23.75358	2.464337	9.638934	5.475383e-22	1.977982e-19	Bacteria
ASV225	34.42432	24.69437	2.585441	9.551319	1.280559e-21	3.084014e-19	Bacteria
ASV438	19.22562	23.89609	2.500702	9.555754	1.226875e-21	3.084014e-19	Bacteria

6 rows | 1-8 of 13 columns

```
dim(sigtab_phosphate)
```

```
## [1] 22 12
```

```
###################################################### Root Biomass Analysis

# Define the subset of samples for root tissue, excluding genotypes 71 and 495
root_biomass_data <- subset_samples(r16S, tissue %in% c("root") & !genotype %in% c("7
1", "495"))
root_biomass_data <- subset_taxa(root_biomass_data, !taxa_sums(root_biomass_data) ==
0)

# Add interval categorization for root biomass (ratioroot < 0.80 as "-", ratioroot >
1.20 as "+", else "/")
sample_data(root_biomass_data)$interval_ratioroot <- as.factor(
  ifelse(sample_data(root_biomass_data)$ratioroot < 0.80, "-",
         ifelse(sample_data(root_biomass_data)$ratioroot > 1.20, "+", "/"))
)

# Create a subset with only "+" and "-" intervals for root biomass
root_biomass_fold <- subset_samples(root_biomass_data, interval_ratioroot != "/" & in
terval_ratioroot != "")
root_biomass_fold <- subset_taxa(root_biomass_fold, taxa_sums(root_biomass_fold) > 0)
# Remove taxa with zero sum

# Rename the interval categorization to binary ("high" for "+" and "low" for "-")
sample_data(root_biomass_fold)$interval_ratioroot_binary <-
  ifelse(sample_data(root_biomass_fold)$interval_ratioroot %in% c("+"), "high", "lo
w")

sample_data(root_biomass_fold)$interval_ratioroot_binary <- as.factor(sample_data(roo
t_biomass_fold)$interval_ratioroot_binary)

# Check the levels of the new binary factor for root biomass intervals
levels(sample_data(root_biomass_fold)$interval_ratioroot_binary)
```

```
## [1] "high" "low"
```

```
# Perform DESeq2 analysis to compare root biomass groups
diagdds_root_biomass <- phyloseq_to_deseq2(root_biomass_fold, ~ interval_ratioroot_bi
nary)
```

```
## converting counts to integer mode
```

```
# Function to calculate geometric mean for size factor estimation
gm_mean <- function(x, na.rm = TRUE) {
  exp(sum(log(x[x > 0]), na.rm = na.rm) / length(x))
}

# Estimate geometric means across samples for size factor normalization
geoMeans <- apply(counts(diagdds_root_biomass), 1, gm_mean)
diagdds_root_biomass <- estimateSizeFactors(diagdds_root_biomass, geoMeans = geoMean
s)

# Perform differential expression analysis using DESeq2
diagdds_root_biomass <- DESeq(diagdds_root_biomass, fitType = "local")
```

```
## using pre-existing size factors
```

```
## estimating dispersions
```

```
## gene-wise dispersion estimates
```

```
## mean-dispersion relationship
```

```
## final dispersion estimates
```

```
## fitting model and testing
```

```
## -- replacing outliers and refitting for 16357 genes
## -- DESeq argument 'minReplicatesForReplace' = 7
## -- original counts are preserved in counts(dds)
```

```
## estimating dispersions
```

```
## fitting model and testing
```

```
# Alternatively, retrieve the results comparing "high" and "low" root biomass
res_root_biomass <- results(diagdds_root_biomass, cooksCutoff = FALSE, contrast = c
("interval_ratioroot_binary", "high", "low"))

# Display the top results for high vs. low root biomass
head(res_root_biomass)  # WaldPvalue for the comparison between high and low root bio
mass
```

```
## log2 fold change (MLE): interval_ratioroot_binary high vs low
## Wald test p-value: interval_ratioroot_binary high vs low
## DataFrame with 6 rows and 6 columns
##       baseMean log2FoldChange     lfcSE      stat    pvalue      padj
##      <numeric>      <numeric> <numeric> <numeric> <numeric> <numeric>
## ASV1  1260.728      0.1016542  0.421084  0.241411 0.8092366  0.929135
## ASV2   743.614      2.4042523  1.244075  1.932562 0.0532902  0.929135
## ASV3   339.422      0.0541030  0.393492  0.137495 0.8906398  0.941761
## ASV4   300.453     -0.4747774  0.833063 -0.569918 0.5687334  0.929135
## ASV5   194.718     -0.2858142  0.569407 -0.501951 0.6157023  0.929135
## ASV6   289.419     -0.0796962  0.659157 -0.120906 0.9037653  0.943576
```

```
# Set alpha threshold for significance (adjusted p-value < 0.05)
alpha <- 0.05
sigtab_root_biomass <- res_root_biomass[which(res_root_biomass$padj < alpha), ]  # Su
bset results for significant taxa
sigtab_root_biomass <- cbind(as(sigtab_root_biomass, "data.frame"), as(tax_table(root
_biomass_fold)[rownames(sigtab_root_biomass), ], "matrix"))

# Check the dimensions and head of the significant results
dimnames(sigtab_root_biomass)
```

```
## [[1]]
##  [1] "ASV156"   "ASV166"   "ASV292"   "ASV318"   "ASV521"   "ASV534"
##  [7] "ASV1037"  "ASV1803"  "ASV2775"  "ASV2862"  "ASV5282"  "ASV5475"
## [13] "ASV5541"  "ASV5608"  "ASV6004"  "ASV6126"  "ASV6558"  "ASV7716"
## [19] "ASV10389" "ASV10471" "ASV14131" "ASV29354"
##
## [[2]]
##  [1] "baseMean"       "log2FoldChange" "lfcSE"          "stat"
##  [5] "pvalue"         "padj"           "Kingdom"        "Phylum"
##  [9] "Class"          "Order"          "Family"         "Genus"
```

```
head(sigtab_root_biomass)
```

	baseM... <dbl>	log2FoldChange <dbl>	lfcSE <dbl>	stat <dbl>	pvalue <dbl>	padj <dbl>	Kingdom <chr>
ASV156	12.49802	23.538930	3.071665	7.663247	1.812905e-14	1.584842e-11	Bacteria
ASV166	22.33884	24.309322	3.043248	7.987952	1.371987e-15	1.499239e-12	Bacteria
ASV292	37.94345	24.948487	2.533765	9.846408	7.103769e-23	1.552529e-19	Bacteria
ASV318	21.69279	9.358961	2.071703	4.517521	6.256788e-06	1.243110e-03	Bacteria
ASV521	17.13204	23.765088	2.410655	9.858354	6.307517e-23	1.552529e-19	Bacteria
ASV534	13.44384	21.316529	2.583675	8.250468	1.577748e-16	2.298779e-13	Bacteria

6 rows | 1-8 of 13 columns

```
# Display the dimension of the significant results table
dim(sigtab_root_biomass)
```

```
## [1] 22 12
```

16.21 - Figure 6A

```
#####################################log2FoldChange

# Load necessary libraries
library(ggplot2)
library(tidyverse)

# Set the default theme for plots
theme_set(theme_bw())

# Custom color palette for the Order variable
scale_fill_discrete <- function(palname = "Set1", ...) {
    scale_fill_brewer(palette = palname, ...)
}

# Phylum-level analysis for log2FoldChange
x <- tapply(sigtab_phosphate$log2FoldChange, sigtab_phosphate$Phylum, function(x) max
(x))
x <- sort(x, decreasing = TRUE)
sigtab_phosphate$Phylum <- factor(as.character(sigtab_phosphate$Phylum), levels = nam
es(x))

# Order-level analysis for log2FoldChange
x <- tapply(sigtab_phosphate$log2FoldChange, sigtab_phosphate$Order, function(x) max
(x))
x <- sort(x, decreasing = TRUE)
sigtab_phosphate$Order <- factor(as.character(sigtab_phosphate$Order), levels = names
(x))

# Define custom colors for the Order variable
order_colors <- c(
  "Flavobacteriales" = "#CBD588",
  "Burkholderiales" = "#5F7FC7",
  "Propionibacteriales" = "#DA5724",
  "Haliangiales" = "#508578",
  "Pedosphaerales" = "#B8860B",
  "Xanthomonadales" = "#AD6F3B",
  "Acidobacteriales" = "#673770",
  "Micromonosporales" = "#20B2AA",
  "Bryobacterales" = "orange",
  "Catenulisporales" = "#C84248",
  "Micropepsales" = "#8569D5",
  "Streptomycetales" = "#32CD32",
  "Chloroflexales" = "#D1A33D",
  "KF-JG30-C25" = "#8B4513",
  "Subgroup_2" = "yellow",
  "Caulobacterales" = "#FF6347",
  "Enterobacterales" = "#4682B4",
  "Paenibacillales" = "#5E738F",
  "Acetobacterales" = "#8A2BE2",
  "Gaiellales" = "#4682B4",
  "Chloroflexales" = "#A52A2A",
  "Solirubrobacterales" = "#DC143C",
  "Solibacterales" = "#FF8C00",
  "Xanthomonadales" = "#800080",
  "Corynebacteriales" = "#00FA9A",
```

```
  "Elsterales" = "#FF1493",
  "Frankiales" = "black",
  "Burkholderiales" = "#D2691E",
  "Thermoactinomycetales" = "#2E8B57",
  "Micrococcales" = "#CD9BCD",
  "Staphylococcales" = "#FF4500",
  "Rhizobiales" = "#D14285",
  "Sphingobacteriales" = "#B22222",
  "Rhodospirillales" = "#FF69B4",
  "Myxococcales" = "#32CD32"
)

# Set the overall plot theme
theme_set(theme_bw() +
          theme(panel.grid.major = element_blank(),
                panel.grid.minor = element_blank(),
                panel.border = element_rect(color = "black", fill = NA),
                axis.title.x = element_text(size = 12),
                axis.title.y = element_text(size = 12),
                axis.text.x = element_text(size = 7),
                axis.text.y = element_text(size = 7),
                legend.title = element_text(size = 10),
                legend.text = element_text(size = 8)))

# Ensure the ASV column is available for the plot
sigtab_phosphate$ASV <- rownames(sigtab_phosphate)

# Reorder data based on log2FoldChange (ascending order)
sigtab_phosphate <- sigtab_phosphate[order(sigtab_phosphate$log2FoldChange, decreasin
g = FALSE), ]
sigtab_phosphate$ASV <- factor(sigtab_phosphate$ASV, levels = sigtab_phosphate$ASV)

# Assign colors based on the Order variable
sigtab_phosphate$Order <- as.factor(sigtab_phosphate$Order)  # Convert Order to facto
r if necessary
sigtab_phosphate$ColorOrder <- ifelse(sigtab_phosphate$Order %in% names(order_color
s),
                            order_colors[sigtab_phosphate$Order], "#D3D3D3")  # Defau
lt gray for missing colors

# Categorize baseMean into intervals for better visualization
sigtab_phosphate$baseMeanCategory <- cut(sigtab_phosphate$baseMean,
                               breaks = c(0, 1, 10, 20, 30, 40, Inf),  # Custom inter
vals for baseMean
                               labels = c("0-1", "1-10", "10-20", "20-30", "30-40",
"40+"),
                               include.lowest = TRUE)

# Create the plot
gg <- ggplot(sigtab_phosphate, aes(x = log2FoldChange, y = ASV, fill = Order, size =
baseMeanCategory)) +
  geom_point(shape = 21, color = "black", stroke = 0.5) +  # Point with black borders
  geom_vline(xintercept = 0, linetype = "dashed", color = "black") +  # Vertical line
at 0
  scale_size_manual(values = c(2, 4, 6, 8, 10)) +  # Define point size based on baseM
eanCategory
```

```
  scale_fill_manual(values = order_colors) +  # Custom color palette for the Order va
riable
  # Add labels for Class, positioned based on log2FoldChange value
  geom_text(aes(label = Class),
            hjust = ifelse(sigtab_phosphate$log2FoldChange > 10, 1.3, -0.3),  # Left
or right alignment
            size = 3, color = "black") +  # Label size and color
  labs(x = "log2 Fold Change", y = "Taxa", size = "Base Mean", fill = "Order") +
  theme(axis.text.x = element_text(angle = 90, vjust = 0.5, hjust = 1, size = 7),
        axis.text.y = element_text(color = "black"),  # Axis labels color
        axis.title.x = element_text(color = "black"),
        axis.title.y = element_text(color = "black"))

# Display the plot
gg
```

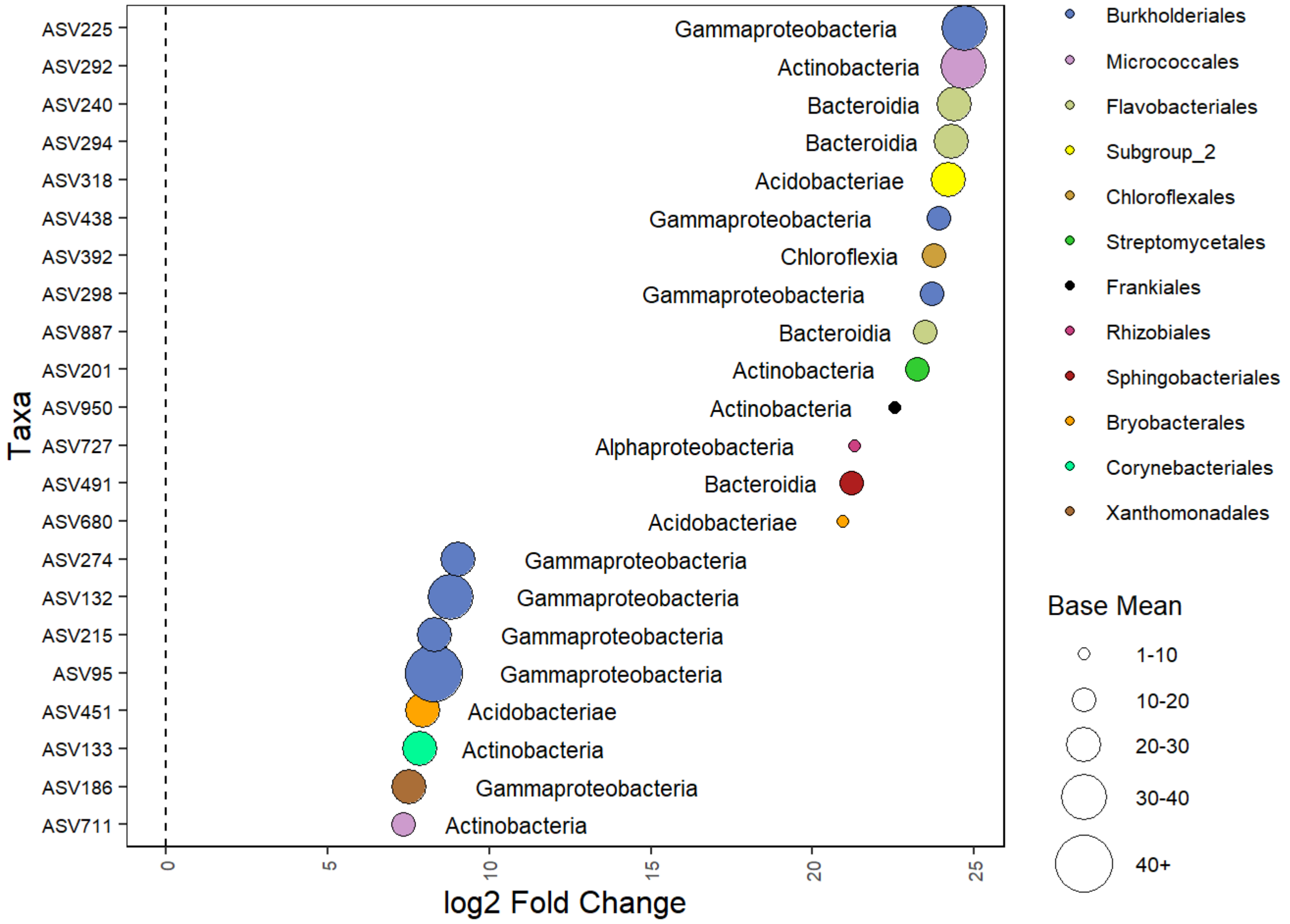

```
######################################## Broad sense Heritability

#1- ASV that are differentially expressed in Pi-/+ conditions

asv_vector = c("ASV225","ASV292","ASV240","ASV294","ASV318","ASV438","ASV392","ASV29
8","ASV887","ASV201","ASV950","ASV727","ASV491","ASV680","ASV274","ASV132","ASV21
5","ASV95","ASV451","ASV133","ASV186","ASV711")

#2- Create a file with relative abundances of these ASV in all samples

otu_mat <- otu_table(r16S)
otu_rel_abund <- apply(otu_mat, 2, function(x) x / sum(x))
otu_selected <- otu_rel_abund[rownames(otu_rel_abund) %in% asv_vector, ]
df_rel_abund <- as.data.frame(t(otu_selected))
metadata <- as.data.frame(sample_data(r16S))
df_rel_abund$genotype <- metadata$genotype
df_rel_abund <- df_rel_abund[, c("genotype", setdiff(names(df_rel_abund), "genotyp
e"))]


write.table(df_rel_abund, "heritability_ASV_extended.txt", sep = "\t", row.names = FA
LSE, quote = FALSE)


#3- lmer method

library(lme4)
```

```
## Caricamento del pacchetto richiesto: Matrix
```

```
##
## Caricamento pacchetto: 'Matrix'
```

```
## Il seguente oggetto è mascherato da 'package:S4Vectors':
##
##     expand
```

```
## I seguenti oggetti sono mascherati da 'package:tidyr':
##
##     expand, pack, unpack
```

```
##
## Caricamento pacchetto: 'lme4'
```

```
## Il seguente oggetto è mascherato da 'package:generics':
##
##     refit
```

```
## Il seguente oggetto è mascherato da 'package:nlme':
##
##     lmList
```

```
#4 - Load your data

df <- read.table('/Users/Alber/Desktop/DOTTORATO/LATTUGA/LANDLAB/DNA/16S-ITS/16S/heri
tability_ASV_extended.txt', #substitute with your path
                 header = TRUE, sep = "\t", stringsAsFactors = FALSE, na.strings = "N
A")

#5 - define which are the traits in your table

traits <- colnames(df)[2:ncol(df)]

#6 - Calculate lmer Heritability

H2_stdN <- data.frame()

for(trait in traits){
  # formula
  f <- as.formula(paste0(trait, ' ~ (1|genotype) + (1|block)'))
  if (!"block" %in% colnames(df)) {
    f <- as.formula(paste0(trait, ' ~ (1|genotype)'))
  }
  fit <- lmer(f, data=df, REML=TRUE)
  v <- as.data.frame(VarCorr(fit))
  Vg <- v$vcov[v$grp == "genotype"]
  Ve <- v$vcov[v$grp == "Residual"]

  # Calcola il numero medio di repliche per genotipo (per questo trait)
  trait_data <- df[, c("genotype", trait)]
  trait_data <- trait_data[!is.na(trait_data[[trait]]), ]
  rep_per_genotype <- table(trait_data$genotype)
  mean_replicates <- mean(rep_per_genotype)

  H2 <- round(Vg / (Vg + Ve / mean_replicates), 6)
  H2_stdN <- rbind(H2_stdN, data.frame("ASV"=trait, "H2_stdN_19"=H2))
}
```

```
## boundary (singular) fit: see help('isSingular')
```

```
## boundary (singular) fit: see help('isSingular')
## boundary (singular) fit: see help('isSingular')
## boundary (singular) fit: see help('isSingular')
## boundary (singular) fit: see help('isSingular')
## boundary (singular) fit: see help('isSingular')
## boundary (singular) fit: see help('isSingular')
## boundary (singular) fit: see help('isSingular')
```

```
#7 - Visualize result
print(H2_stdN)
```

```
##        ASV H2_stdN_19
## 1    ASV95   0.000000
## 2   ASV132   0.071823
## 3   ASV133   0.439234
## 4   ASV186   0.549030
## 5   ASV201   0.312847
## 6   ASV215   0.000000
## 7   ASV225   0.366628
## 8   ASV240   0.000000
## 9   ASV274   0.357978
## 10  ASV292   0.464628
## 11  ASV294   0.201201
## 12  ASV298   0.094564
## 13  ASV318   0.103598
## 14  ASV392   0.689119
## 15  ASV438   0.091682
## 16  ASV451   0.538743
## 17  ASV491   0.000000
## 18  ASV680   0.000000
## 19  ASV711   0.000000
## 20  ASV727   0.000000
## 21  ASV887   0.000000
## 22  ASV950   0.456125
```

```
############Heatmap

library(ggplot2)
library(reshape2)
library(dplyr)

#1- Create a vector with the Broad sense heritability values

H2_stdN <- data.frame(
  ASV = c("ASV95", "ASV132", "ASV133", "ASV186", "ASV201", "ASV215", "ASV225", "ASV24
0",
          "ASV274", "ASV292", "ASV294", "ASV298", "ASV318", "ASV392", "ASV438", "ASV4
51",
          "ASV491", "ASV680", "ASV711", "ASV727", "ASV887", "ASV950"),
  H2_stdN = c(0.000000, 0.071823, 0.439234, 0.549030, 0.312847, 0.000000, 0.366628,
0.000000,
              0.357978, 0.464628, 0.201201, 0.094564, 0.103598, 0.689119, 0.091682,
0.538743,
              0.000000, 0.000000, 0.000000, 0.000000, 0.000000, 0.456125),
  Relative_abundance = rep("R.A%", 22)
)


#2- Order the data frame
heritability_ordered <- H2_stdN %>%
  arrange(H2_stdN)

heritability_ordered$ASV <- factor(heritability_ordered$ASV, levels = heritability_or
dered$ASV)

#3- Create the Heatmap

H2 <- ggplot(heritability_ordered, aes(x = Relative_abundance, y = ASV, fill = H2_std
N)) +
  geom_tile(color = "white") +
  scale_fill_gradient(low = "white", high = "purple", na.value = "grey") +
  theme_minimal() +
  theme(axis.text.x = element_text(angle = 0, hjust = 1),
        axis.text.y = element_text(size = 10)) +
  labs(title = "Broad Sense Heritability", x = "Group", y = "ASV", fill = "Heritabili
ty")

H2
```

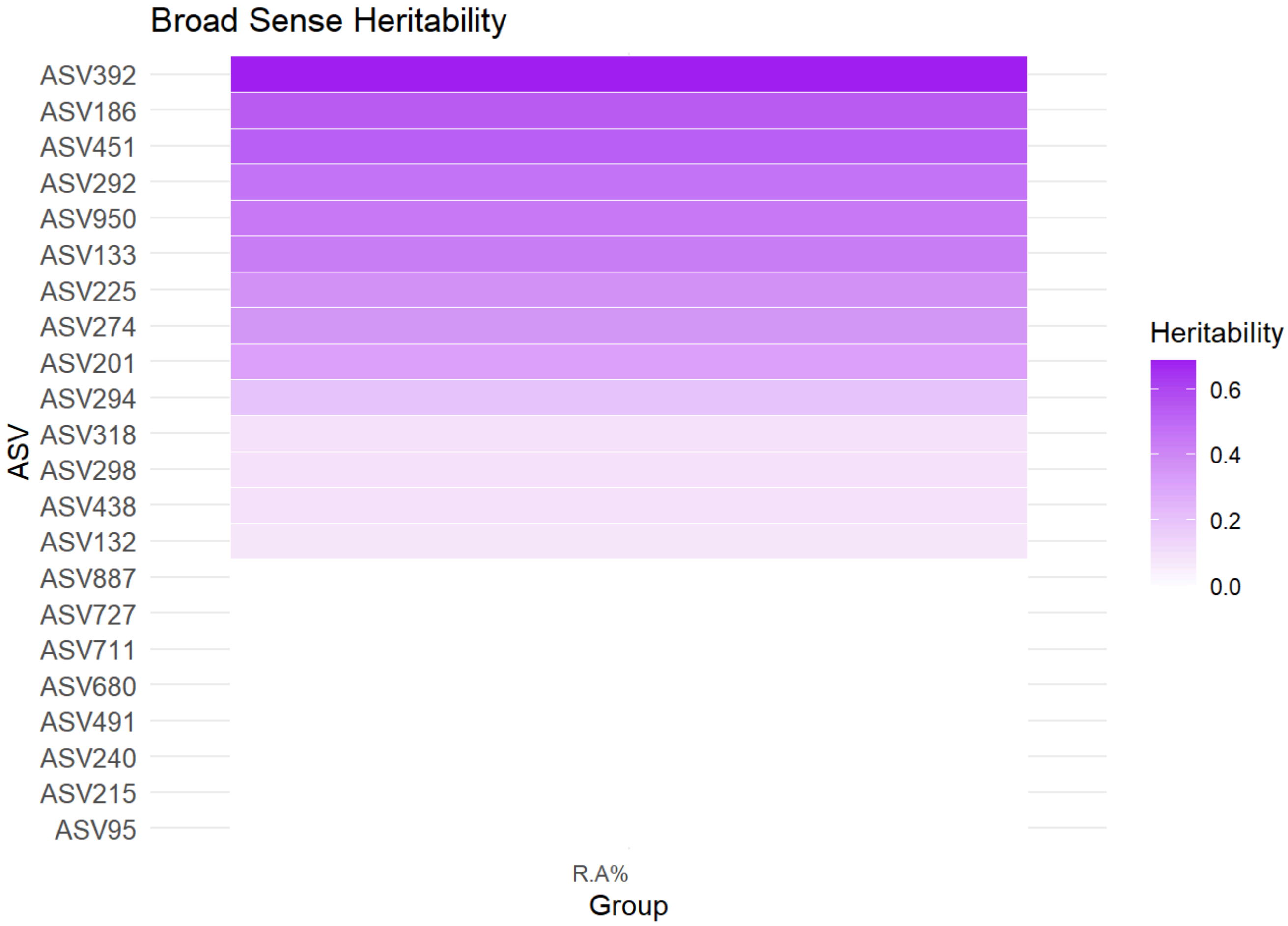

16.22 - Figure 6B and Figure S12A

```
######################################log2FoldChange

# Load necessary libraries
library(ggplot2)
library(tidyverse)

# Set the default theme for plots
theme_set(theme_bw())

# Custom color palette for the Order variable
scale_fill_discrete <- function(palname = "Set1", ...) {
    scale_fill_brewer(palette = palname, ...)
}

# Phylum-level analysis for log2FoldChange
x <- tapply(sigtab_root_biomass$log2FoldChange, sigtab_root_biomass$Phylum, function
(x) max(x))
x <- sort(x, decreasing = TRUE)
sigtab_root_biomass$Phylum <- factor(as.character(sigtab_root_biomass$Phylum), levels
= names(x))

# Order-level analysis for log2FoldChange
x <- tapply(sigtab_root_biomass$log2FoldChange, sigtab_root_biomass$Order, function
(x) max(x))
x <- sort(x, decreasing = TRUE)
sigtab_root_biomass$Order <- factor(as.character(sigtab_root_biomass$Order), levels =
names(x))

# Define custom colors for the Order variable
order_colors <- c(
  "Flavobacteriales" = "#CBD588",
  "Burkholderiales" = "#5F7FC7",
  "Propionibacteriales" = "#DA5724",
  "Haliangiales" = "#508578",
  "Pedosphaerales" = "#B8860B",
  "Xanthomonadales" = "#AD6F3B",
  "Acidobacteriales" = "#673770",
  "Micromonosporales" = "#20B2AA",
  "Bryobacterales" = "orange",
  "Catenulisporales" = "#C84248",
  "Micropepsales" = "#8569D5",
  "Streptomycetales" = "#32CD32",
  "Chloroflexales" = "#D1A33D",
  "KF-JG30-C25" = "#8B4513",
  "Subgroup_2" = "yellow",
  "Caulobacterales" = "#FF6347",
  "Enterobacterales" = "#4682B4",
  "Paenibacillales" = "#5E738F",
  "Acetobacterales" = "#8A2BE2",
  "Gaiellales" = "#4682B4",
  "Chloroflexales" = "#A52A2A",
  "Solirubrobacterales" = "#DC143C",
  "Solibacterales" = "#FF8C00",
  "Xanthomonadales" = "#800080",
  "Corynebacteriales" = "#00FA9A",
```

```
  "Elsterales" = "#FF1493",
  "Frankiales" = "black",
  "Burkholderiales" = "#D2691E",
  "Thermoactinomycetales" = "#2E8B57",
  "Micrococcales" = "#CD9BCD",
  "Staphylococcales" = "#FF4500",
  "Rhizobiales" = "#D14285",
  "Sphingobacteriales" = "#B22222",
  "Rhodospirillales" = "#FF69B4",
  "Myxococcales" = "#32CD32"
)

# Set the overall plot theme
theme_set(theme_bw() +
          theme(panel.grid.major = element_blank(),
                panel.grid.minor = element_blank(),
                panel.border = element_rect(color = "black", fill = NA),
                axis.title.x = element_text(size = 12),
                axis.title.y = element_text(size = 12),
                axis.text.x = element_text(size = 7),
                axis.text.y = element_text(size = 7),
                legend.title = element_text(size = 10),
                legend.text = element_text(size = 8)))

# Ensure the ASV column is available for the plot
sigtab_root_biomass$ASV <- rownames(sigtab_root_biomass)

# Reorder data based on log2FoldChange (ascending order)
sigtab_root_biomass <- sigtab_root_biomass[order(sigtab_root_biomass$log2FoldChange,
decreasing = FALSE), ]
sigtab_root_biomass$ASV <- factor(sigtab_root_biomass$ASV, levels = sigtab_root_bioma
ss$ASV)

# Assign colors based on the Order variable
sigtab_root_biomass$Order <- as.factor(sigtab_root_biomass$Order)  # Convert Order to
factor if necessary
sigtab_root_biomass$ColorOrder <- ifelse(sigtab_root_biomass$Order %in% names(order_c
olors),
                            order_colors[sigtab_root_biomass$Order], "#D3D3D3")  # De
fault gray for missing colors

# Categorize baseMean into intervals for better visualization
sigtab_root_biomass$baseMeanCategory <- cut(sigtab_root_biomass$baseMean,
                               breaks = c(0, 1, 10, 20, 30, 40, Inf),  # Custom inter
vals for baseMean
                               labels = c("0-1", "1-10", "10-20", "20-30", "30-40",
"40+"),
                               include.lowest = TRUE)

# Create the plot
gg <- ggplot(sigtab_root_biomass, aes(x = log2FoldChange, y = ASV, fill = Order, size
= baseMeanCategory)) +
  geom_point(shape = 21, color = "black", stroke = 0.5) +  # Point with black borders
  geom_vline(xintercept = 0, linetype = "dashed", color = "black") +  # Vertical line
at 0
  scale_size_manual(values = c(2, 4, 6, 8, 10)) +  # Define point size based on baseM
```

```
eanCategory
  scale_fill_manual(values = order_colors) +  # Custom color palette for the Order va
riable
  # Add labels for Class, positioned based on log2FoldChange value
  geom_text(aes(label = Class),
            hjust = ifelse(sigtab_root_biomass$log2FoldChange > 10, 1.3, -0.3),  # Le
ft or right alignment
            size = 3, color = "black") +  # Label size and color
  labs(x = "log2 Fold Change", y = "Taxa", size = "Base Mean", fill = "Order") +
  theme(axis.text.x = element_text(angle = 90, vjust = 0.5, hjust = 1, size = 7),
        axis.text.y = element_text(color = "black"),  # Axis labels color
        axis.title.x = element_text(color = "black"),
        axis.title.y = element_text(color = "black"))

# Display the plot
gg
```

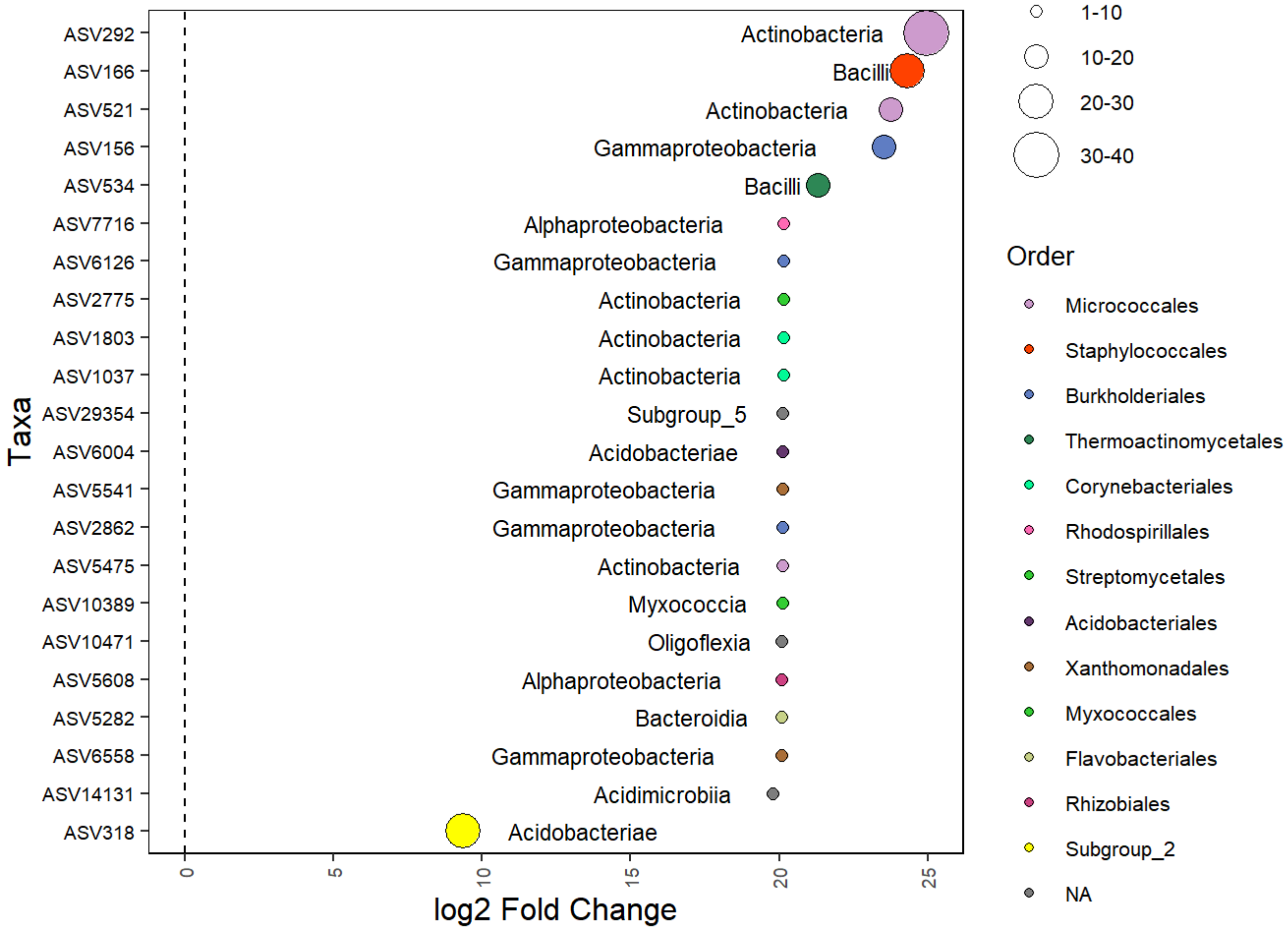

```
#####################################Broad sense Heritability

#1- ASV that are differentially expressed in Pi-/+ conditions
asv_vector <- c(
  "ASV156", "ASV166", "ASV292", "ASV318", "ASV521", "ASV534", "ASV1037", "ASV1803",
  "ASV2775", "ASV2862", "ASV5282", "ASV5475", "ASV5541", "ASV5608", "ASV6004",
  "ASV6126", "ASV6558", "ASV7716", "ASV10389", "ASV10471", "ASV14131", "ASV29354"
)

#2- Create a file with relative abundances of these ASV in all samples

otu_mat <- otu_table(r16S)
otu_rel_abund <- apply(otu_mat, 2, function(x) x / sum(x))
otu_selected <- otu_rel_abund[rownames(otu_rel_abund) %in% asv_vector, ]
df_rel_abund <- as.data.frame(t(otu_selected))
metadata <- as.data.frame(sample_data(r16S))
df_rel_abund$genotype <- metadata$genotype
df_rel_abund <- df_rel_abund[, c("genotype", setdiff(names(df_rel_abund), "genotyp
e"))]


write.table(df_rel_abund, "heritability_ASV_extended_root.txt", sep = "\t", row.names
= FALSE, quote = FALSE)

#3- Imer method
library(lme4)

#4 - Load your data

df <- read.table('/Users/Alber/Desktop/DOTTORATO/LATTUGA/LANDLAB/DNA/16S-ITS/16S/heri
tability_ASV_extended_root.txt',#substitute with your path
                 header = TRUE, sep = "\t", stringsAsFactors = FALSE, na.strings = "N
A")

#5 - define the traits in your table

traits <- colnames(df)[2:ncol(df)]

#6 - Calculate Imer Heritability

H2_stdN <- data.frame()

for(trait in traits){
  # formula
  f <- as.formula(paste0(trait, ' ~ (1|genotype) + (1|block)'))
  if (!"block" %in% colnames(df)) {
    f <- as.formula(paste0(trait, ' ~ (1|genotype)'))
  }
  fit <- lmer(f, data=df, REML=TRUE)
  v <- as.data.frame(VarCorr(fit))
  Vg <- v$vcov[v$grp == "genotype"]
  Ve <- v$vcov[v$grp == "Residual"]

  # Calcola il numero medio di repliche per genotipo (per questo trait)
  trait_data <- df[, c("genotype", trait)]
```

```
  trait_data <- trait_data[!is.na(trait_data[[trait]]), ]
  rep_per_genotype <- table(trait_data$genotype)
  mean_replicates <- mean(rep_per_genotype)

  H2 <- round(Vg / (Vg + Ve / mean_replicates), 6)
  H2_stdN <- rbind(H2_stdN, data.frame("ASV"=trait, "H2_stdN_19"=H2))
}
```

```
## boundary (singular) fit: see help('isSingular')
## boundary (singular) fit: see help('isSingular')
## boundary (singular) fit: see help('isSingular')
## boundary (singular) fit: see help('isSingular')
## boundary (singular) fit: see help('isSingular')
## boundary (singular) fit: see help('isSingular')
## boundary (singular) fit: see help('isSingular')
## boundary (singular) fit: see help('isSingular')
## boundary (singular) fit: see help('isSingular')
## boundary (singular) fit: see help('isSingular')
## boundary (singular) fit: see help('isSingular')
## boundary (singular) fit: see help('isSingular')
## boundary (singular) fit: see help('isSingular')
## boundary (singular) fit: see help('isSingular')
```

```
#7 - Visualize result
print(H2_stdN)
```

```
##         ASV H2_stdN_19
## 1    ASV156   0.000000
## 2    ASV166   0.628785
## 3    ASV292   0.464628
## 4    ASV318   0.103598
## 5    ASV521   0.335263
## 6    ASV534   0.202231
## 7   ASV1037   0.000000
## 8   ASV1803   0.000000
## 9   ASV2775   0.618801
## 10  ASV2862   0.000000
## 11  ASV5282   0.000000
## 12  ASV5475   0.000000
## 13  ASV5541   0.000000
## 14  ASV5608   0.000000
## 15  ASV6004   0.000000
## 16  ASV6126   0.000000
## 17  ASV6558   0.787111
## 18  ASV7716   0.000000
## 19 ASV10389   0.193520
## 20 ASV10471   0.000000
## 21 ASV14131   0.000000
## 22 ASV29354   0.000000
```

```
################ Heatmap

library(ggplot2)
library(reshape2)
library(dplyr)

#1- Create a vector with the Broad sense Heritability values
H2_stdN <- data.frame(
  ASV = c("ASV156", "ASV166", "ASV292", "ASV318", "ASV521", "ASV534", "ASV1037", "ASV
1803",
          "ASV2775", "ASV2862", "ASV5282", "ASV5475", "ASV5541", "ASV5608", "ASV600
4", "ASV6126",
          "ASV6558", "ASV7716", "ASV10389", "ASV10471", "ASV14131", "ASV29354"),
  H2_stdN = c(0.000000, 0.628785, 0.464628, 0.103598, 0.335263, 0.202231, 0.000000,
0.000000,
              0.618801, 0.000000, 0.000000, 0.000000, 0.000000, 0.000000, 0.000000,
0.000000,
              0.787111, 0.000000, 0.193520, 0.000000, 0.000000, 0.000000),
  Relative_abundance = rep("R.A%", 22)
)


#2- Order the dataframe
heritability_ordered <- H2_stdN %>%
  arrange(H2_stdN)

heritability_ordered$ASV <- factor(heritability_ordered$ASV, levels = heritability_or
dered$ASV)

#3- Heatmap
H2 <- ggplot(heritability_ordered, aes(x = Relative_abundance, y = ASV, fill = H2_std
N)) +
  geom_tile(color = "white") +
  scale_fill_gradient(low = "white", high = "purple", na.value = "grey") +
  theme_minimal() +
  theme(axis.text.x = element_text(angle = 0, hjust = 1),
        axis.text.y = element_text(size = 10)) +
  labs(title = "Broad Sense Heritability", x = "Group", y = "ASV", fill = "Heritabili
ty")

H2
```

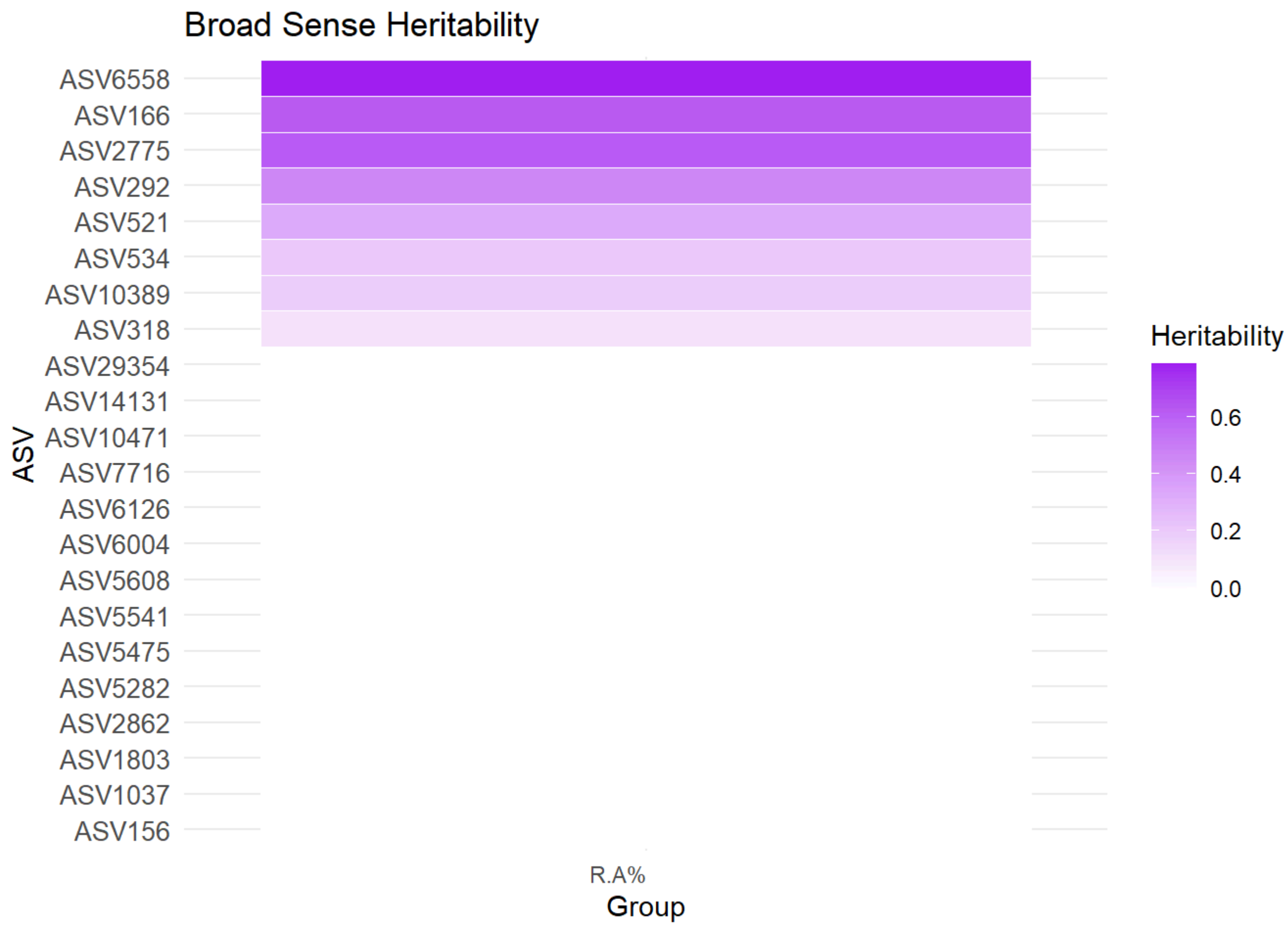

17 - ITS analysis

17.1 - import, filter, merge sequences and assign taxonomy

```
## 1 - Load Required Libraries
library(dada2)  # For sequence processing and analysis
packageVersion("dada2")  # Check installed version of dada2 package

library(ShortRead)  # For working with FASTQ files
packageVersion("ShortRead")  # Check installed version of ShortRead package

library(Biostrings)  # For biological string manipulation
packageVersion("Biostrings")  # Check installed version of Biostrings package

library(phyloseq)  # For microbiome analysis and visualization

## 2 - Set File Path for Input Data
path <- "C:/your_path/Raw.data/"  # Define the path to the directory containing the r
aw FASTQ files.
list.files(path)  # List all files in the specified directory

## 3 - Import Forward and Reverse Reads Import reads without primers or
## adapters.
fnFs <- sort(list.files(path, pattern = "_1.fastq.gz", full.names = TRUE))  # Forward
read files

## 4 - Primer Sequences Primer sequences for forward and reverse reads (for
## reference).
FWD <- "GCATCGATGAAGAACGCAGC"  # CHANGE to your forward primer sequence
REV <- "TCCTCCGCTTATTGATATGC"  # CHANGE to your reverse primer sequence

## 5 - Remove Sequences with Ambiguous Bases Filter out sequences with
## ambiguous bases (Ns).
fnFs.filtN <- file.path(path, "filtN", basename(fnFs))  # Create output directory for
N-filtered files
filterAndTrim(fnFs, fnFs.filtN, maxN = 0, multithread = TRUE)  # Remove sequences wit
h Ns (ambiguous bases)

## 6 - String Manipulation to Match Forward and Reverse Files Ensure that
## forward and reverse FASTQ files are correctly paired.
path.filt <- file.path(path, "filtN")
cutFs <- sort(list.files(path.filt, pattern = "_1.fastq.gz", full.names = TRUE))  # F
iltered forward reads

# Extract sample names from file names.
get.sample.name <- function(fname) strsplit(basename(fname), "_")[[1]][1]
sample.names <- unname(sapply(cutFs, get.sample.name))  # Extract sample names from f
orward read filenames
head(sample.names)

## 7 - Inspect Quality Profile Plot quality profile for the first few forward
## and reverse reads.
pFs <- plotQualityProfile(cutFs[1:103])  # Quality profile for the first 103 forward
reads

## 8 - Trimming Reads Trim reads to remove low-quality bases and other unwanted
## regions.
filtFs <- file.path(path.filt, "filtered", basename(cutFs))  # Filtered forward read
output path
```

```
out <- filterAndTrim(cutFs, filtFs, maxN = 0, maxEE = c(2), truncQ = 2, minLen = 50,
    rm.phix = TRUE, compress = TRUE, multithread = FALSE)  # Apply filters to trim re
ads
head(out)

## 9 - Inspect the Error Rate Learn the error rates for forward and reverse
## reads.
errF <- learnErrors(filtFs, multithread = TRUE)  # Error rates for forward reads
plotErrors(errF, nominalQ = TRUE)  # Plot error rates for forward reads

## 10 - Sample Inference Infer the sample sequences (denoising process).
dadaFs <- dada(filtFs, err = errF, multithread = TRUE)  # Denoise forward reads

## 11 - Construct Sequence Table Create a sequence table from the denoised
## reads.
seqtab <- makeSequenceTable(dadaFs)  # Create sequence table from forward reads
dim(seqtab)  # Display dimensions of the sequence table

## 12 - Remove Chimeras Remove chimeric sequences from the sequence table.
seqtab.nochim <- removeBimeraDenovo(seqtab, method = "consensus", multithread = TRUE,
    verbose = TRUE)  # Remove chimeras using the consensus method
table(nchar(getSequences(seqtab.nochim)))  # Check the lengths of the sequences after
chimera removal
write.csv(t(seqtab.nochim), "16S_table-FINAL.csv")  # Write the final sequence table
to CSV

## 13 - Read Track Track the progress of each step for each sample.
getN <- function(x) sum(getUniques(x))  # Function to get the number of unique sequen
ces
track <- cbind(out, sapply(dadaFs, getN), rowSums(seqtab.nochim))  # Combine informat
ion on input, filtered, denoised, and non-chimeric sequences
colnames(track) <- c("input", "filtered", "denoisedF", "nonchim")  # Set column names
for the track table
rownames(track) <- sample.names  # Set row names as sample names
head(track)  # Display the first few rows of the track table
write.csv(track, "track_table_ITS.csv")  # Write the track table to CSV

## 14 - Assign Taxonomy Assign taxonomy to the sequences using a reference
## database.
getwd()  # Get the current working directory
unite.ref <- "C:/your_path/sh_general_release_dynamic_25.07.2023.fasta"  # Path to th
e UNITE reference database
taxa <- assignTaxonomy(seqtab.nochim, unite.ref, multithread = TRUE, tryRC = TRUE)  #
Assign taxonomy using the UNITE reference database
taxa.print <- taxa  # Create a copy of the taxonomy assignment for display
rownames(taxa.print) <- NULL  # Remove row names for display purposes
head(taxa.print)  # Display the first few rows of the taxonomy assignment
write.csv(taxa, "taxonomy.csv")  # Write the taxonomy assignment to CSV
```

17.2 - Phyloseq object creation and contamination removal

```
# Load necessary packages
library("phyloseq")
library("ggplot2")      # for data visualization
library("readxl")       # for importing data from Excel files
library("dplyr")        # for data manipulation and formatting
library("tibble")       # for handling tibbles
library(devtools)
library(ranacapa)
library("tidyverse")
library(rstatix)
library(ggpubr)

# Upload data tables

## ASV Table
asv16S <- read.table(file="C:/Users/Alber/Desktop/DOTTORATO/LATTUGA/LANDLAB/DNA/16S-I
TS/ITS/Phyloseq Object/16S_table-FINAL.txt", sep="\t", dec = ".", header=TRUE) #repla
ce with your path
## Taxonomy Table
tax16S <- read.table(file="C:/Users/Alber/Desktop/DOTTORATO/LATTUGA/LANDLAB/DNA/16S-I
TS/ITS/Phyloseq Object/taxonomy.txt", sep="\t", dec = ".", header=TRUE)#replace with
your path
## Design Table
design <- read.table(file="C:/Users/Alber/Desktop/DOTTORATO/LATTUGA/LANDLAB/DNA/16S-I
TS/ITS/Phyloseq Object/design.txt", sep="\t", dec = ",", header=TRUE)#replace with yo
ur path

# Prepare data for creating the Phyloseq object
asv16S <- asv16S %>%
    tibble::column_to_rownames("OTU.ID")
colnames(asv16S) <- sub("_1.fastq.gz", "", colnames(asv16S))
tax16S <- tax16S %>%
    tibble::column_to_rownames("OTU.ID")

# Clean and reformat the taxonomy table
library(stringr)
# tax16S[c("Kingdom","Phylum", "Class", "Order", "Family", "Genus", "Species")] <- st
r_split_fixed(tax16S$Taxon, ';', 7)
tax16S[tax16S == ""] <- NA
tax16S[tax16S == " "] <- NA
design <- design %>%
    tibble::column_to_rownames("SAMPLE.ID")

otu_mat <- as.matrix(asv16S)
tax_mat <- as.matrix(tax16S)

# Create the Phyloseq object
OTU = otu_table(otu_mat, taxa_are_rows = TRUE)
TAX = tax_table(tax_mat)
samples = sample_data(design)
ps16S_0 <- phyloseq(OTU, TAX, samples)

# Remove contaminants from the data

ps16S_1a <- subset_taxa(ps16S_0, !is.na(Phylum) & !Phylum %in% c("Unassigned"))
```

```
ps16S_1b <- subset_taxa(ps16S_1a, !Order %in% c("o__Chloroplast"))
ps16S_1c <- subset_taxa(ps16S_1b, !Family %in% c("o__Chloroplast"))
ps16S_1d <- subset_taxa(ps16S_1c, !Order %in% c("o__Mitochondria"))
ps16S_1e <- subset_taxa(ps16S_1d, !Family %in% c("f__Mitochondria"))
ps16S_1f <- subset_taxa(ps16S_1e, !Genus %in% c("g__Mitochondria"))
ps16S_1h <- subset_taxa(ps16S_1f, !Kingdom %in% c("d__Eukaryota"))
ps16S_1i <- subset_taxa(ps16S_1h, !Kingdom %in% c("d__Archaea"))

# Rename ASVs
dna.16S <- taxa_names(ps16S_1i)
names(dna.16S) <- taxa_names(ps16S_1i)
ps16S_2 <- merge_phyloseq(ps16S_1i, dna.16S)
taxa_names(ps16S_2) <- paste0("ASV", seq(ntaxa(ps16S_2)))

# Subset samples (remove samples with fewer than 100 counts)
ps16S_3 <- prune_samples(sample_sums(ps16S_2) >= 100, ps16S_2)
ps16S_4 <- filter_taxa(ps16S_3, function(x) sum(x) > 0, TRUE)

# Filter ASVs (retain only ASVs with >0.01% relative abundance per sample)
ps16S_5 <- transform_sample_counts(ps16S_4, function(x) ifelse((x/sum(x)) >= 0.0001 , 
x, 0))
ps16S_6 <- filter_taxa(ps16S_5, function(x) sum(x) > 0, TRUE)

# Save the cleaned Phyloseq object
saveRDS(ps16S_6, "ps16S_clean_ITS.rds")  # 12656 taxa and 417 samples

# Upload the cleaned dataset
library(phyloseq); packageVersion("phyloseq")
```

```
## [1] '1.52.0'
```

```
dITS <- readRDS("ps16S_clean_ITS.rds")
```

17.3 - Figure S6C- Rarefaction per compartment

```
library(vegan)
# Rarefy growth soil samples
dITS_filtered_growth_soil <- subset_samples(dITS, tissue %in% c("growth soil"))

# Get the minimum sequencing depth
depth_growth_soil = min(colSums(otu_table(dITS_filtered_growth_soil)))

# Rarefy the growth soil data
rITS_growth_soil <- rarefy_even_depth(dITS_filtered_growth_soil, sample.size = 42893, 
rngseed = 900)
```

```
## `set.seed(900)` was used to initialize repeatable random subsampling.
```

```
## Please record this for your records so others can reproduce.
```

```
## Try `set.seed(900); .Random.seed` for the full vector
```

```
## ...
```

```
## 4207OTUs were removed because they are no longer
## present in any sample after random subsampling
```

```
## ...
```

```
# Filter out taxa with zero counts
rITS_growth_soil_filtered <- subset_taxa(rITS_growth_soil, !taxa_sums(rITS_growth_soi
l) == 0)

# Rarefy germination soil samples
dITS_filtered_germination_soil <- subset_samples(dITS, tissue %in% c("germination soi
l"))

# Get the minimum sequencing depth
depth_germination_soil = min(colSums(otu_table(dITS_filtered_germination_soil)))

# Rarefy the germination soil data
rITS_germination_soil <- rarefy_even_depth(dITS_filtered_germination_soil, sample.siz
e = 29357, rngseed = 900)
```

```
## `set.seed(900)` was used to initialize repeatable random subsampling.
```

```
## Please record this for your records so others can reproduce.
```

```
## Try `set.seed(900); .Random.seed` for the full vector
```

```
## ...
```

```
## 5543OTUs were removed because they are no longer
## present in any sample after random subsampling
```

```
## ...
```

```
# Filter out taxa with zero counts
rITS_germination_soil_filtered <- subset_taxa(rITS_germination_soil, !taxa_sums(rITS_
germination_soil) == 0)

# Rarefy inoculum samples
dITS_filtered_inoculum <- subset_samples(dITS, tissue %in% c("inoculum "))

# Get the minimum sequencing depth
depth_inoculum = min(colSums(otu_table(dITS_filtered_inoculum)))

# Rarefy the inoculum data
rITS_inoculum <- rarefy_even_depth(dITS_filtered_inoculum, sample.size = 70255, rngse
ed = 900)
```

```
## `set.seed(900)` was used to initialize repeatable random subsampling.
```

```
## Please record this for your records so others can reproduce.
```

```
## Try `set.seed(900); .Random.seed` for the full vector
```

```
## ...
```

```
## 5677OTUs were removed because they are no longer
## present in any sample after random subsampling
```

```
## ...
```

```
# Filter out taxa with zero counts
rITS_inoculum_filtered <- subset_taxa(rITS_inoculum, !taxa_sums(rITS_inoculum) == 0)

# Rarefy root samples
dITS_filtered_roots <- subset_samples(dITS, tissue %in% c("root"))

# Get the minimum sequencing depth
depth_roots = min(colSums(otu_table(dITS_filtered_roots)))

# Rarefy the root data
rITS_roots <- rarefy_even_depth(dITS_filtered_roots, sample.size = 1000, rngseed = 90
0)
```

```
## `set.seed(900)` was used to initialize repeatable random subsampling.
```

```
## Please record this for your records so others can reproduce.
```

```
## Try `set.seed(900); .Random.seed` for the full vector
```

```
## ...
```

```
## 36 samples removedbecause they contained fewer reads than `sample.size`.
```

```
## Up to first five removed samples are:
```

```
## A1A17A19A20A31
```

```
## ...
```

```
## 3581OTUs were removed because they are no longer
## present in any sample after random subsampling
```

```
## ...
```

```
# Filter out taxa with zero counts
rITS_roots_filtered <- subset_taxa(rITS_roots, !taxa_sums(rITS_roots) == 0)


# Merge the filtered Phyloseq objects into one
merged_phylo <- merge_phyloseq(
  rITS_growth_soil_filtered,
  rITS_germination_soil_filtered,
  rITS_inoculum_filtered,
  rITS_roots_filtered
)

#Figure S6C
rcurveITS <- ggrare(merged_phylo,color = "tissue",
                    step = 999,
                    se = FALSE)  + labs (title = "ITS rRNA gene")
```

```
## rarefying sample A100
## rarefying sample A101
## rarefying sample A102
## rarefying sample A103
## rarefying sample A96
## rarefying sample A97
## rarefying sample A98
## rarefying sample A99
## rarefying sample A92
```

```
## Warning in vegan::rarefy(x[i, , drop = FALSE], n, se = se): most observed count
## data have counts 1, but smallest count is 3
```

```
## rarefying sample A93
```

```
## Warning in vegan::rarefy(x[i, , drop = FALSE], n, se = se): most observed count
## data have counts 1, but smallest count is 4
```

```
## rarefying sample A94
```

```
## Warning in vegan::rarefy(x[i, , drop = FALSE], n, se = se): most observed count
## data have counts 1, but smallest count is 5
```

```
## rarefying sample A95
```

```
## Warning in vegan::rarefy(x[i, , drop = FALSE], n, se = se): most observed count
## data have counts 1, but smallest count is 4
```

```
## rarefying sample A10
## rarefying sample A11
## rarefying sample A12
## rarefying sample A13
## rarefying sample A14
## rarefying sample A15
## rarefying sample A16
## rarefying sample A18
## rarefying sample A2
## rarefying sample A21
## rarefying sample A22
## rarefying sample A23
## rarefying sample A24
## rarefying sample A25
## rarefying sample A26
## rarefying sample A27
## rarefying sample A28
## rarefying sample A29
## rarefying sample A3
## rarefying sample A30
## rarefying sample A33
## rarefying sample A36
## rarefying sample A37
## rarefying sample A38
## rarefying sample A39
## rarefying sample A4
## rarefying sample A40
## rarefying sample A42
## rarefying sample A44
## rarefying sample A45
## rarefying sample A46
## rarefying sample A47
## rarefying sample A48
## rarefying sample A49
## rarefying sample A5
## rarefying sample A55
## rarefying sample A56
## rarefying sample A58
## rarefying sample A6
## rarefying sample A61
## rarefying sample A63
## rarefying sample A69
## rarefying sample A76
## rarefying sample A78
## rarefying sample A8
## rarefying sample A80
## rarefying sample A85
## rarefying sample A89
## rarefying sample A9
## rarefying sample A90
```

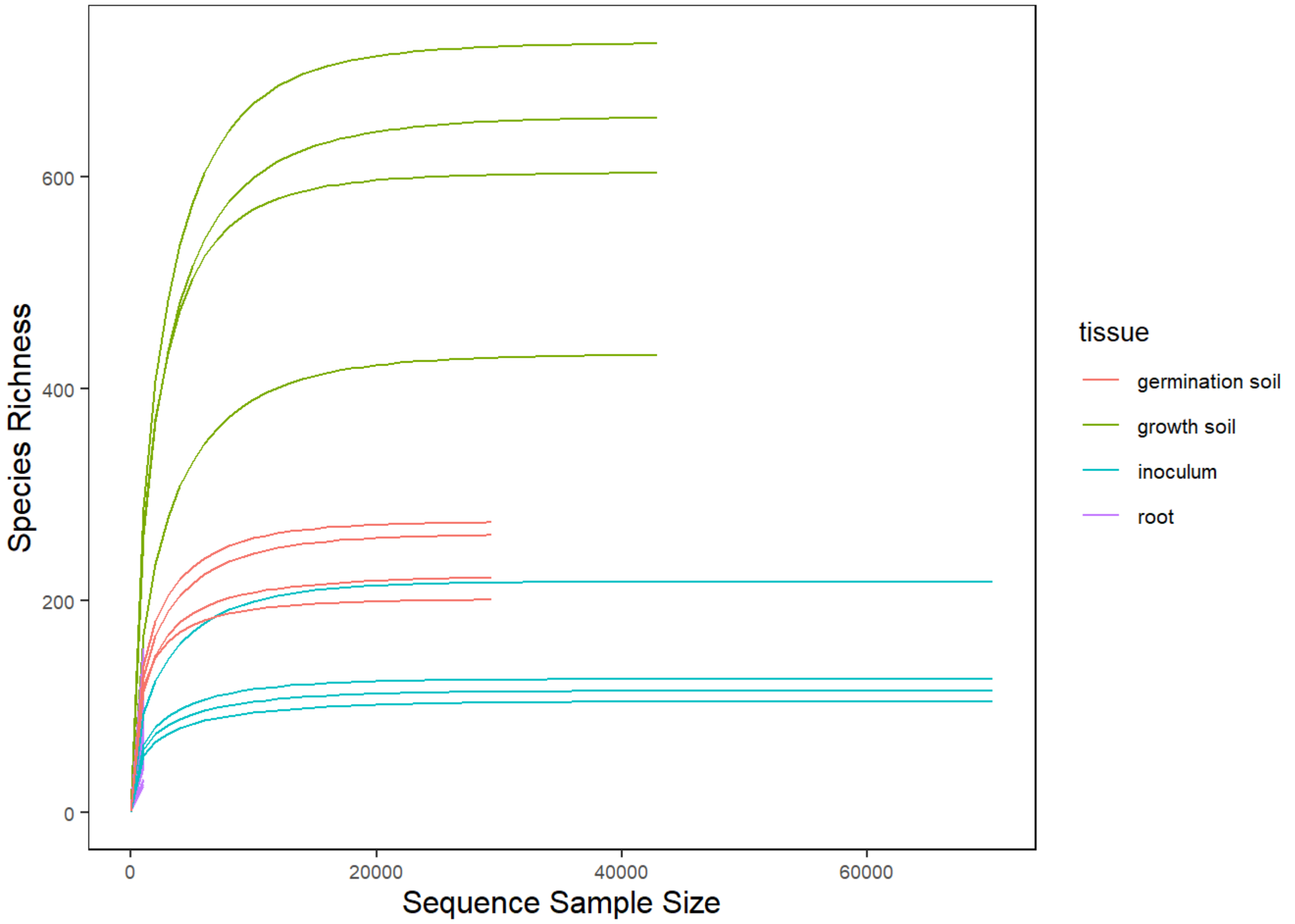


```
# Customize the rarefaction plot
rcurveITSf_rarefied <- rcurveITS +
  theme(strip.background = element_rect(color="white", fill="#585858", size=1, linety
pe="solid"),
        strip.text.x = element_text(size = 12, color = "white"),
        axis.title.x = element_text(size = 12, face = "bold"),
        axis.title.y = element_text(size = 12, face = "bold"),
        plot.title = element_text(size = 16),
        axis.text.x = element_text(face = "bold"),
        axis.text.y = element_text(face = "bold")) +
  ylab("Species Richness") +
  xlab("Sample Size") +
  ggtitle("ITS rRNA Gene") +
  facet_grid(~tissue, scales = "free")
```

```
## Warning: The `size` argument of `element_rect()` is deprecated as of ggplot2 3.4.
0.
## ℹ Please use the `linewidth` argument instead.
## This warning is displayed once every 8 hours.
## Call `lifecycle::last_lifecycle_warnings()` to see where this warning was
## generated.
```

```
rcurveITSf_rarefied
```

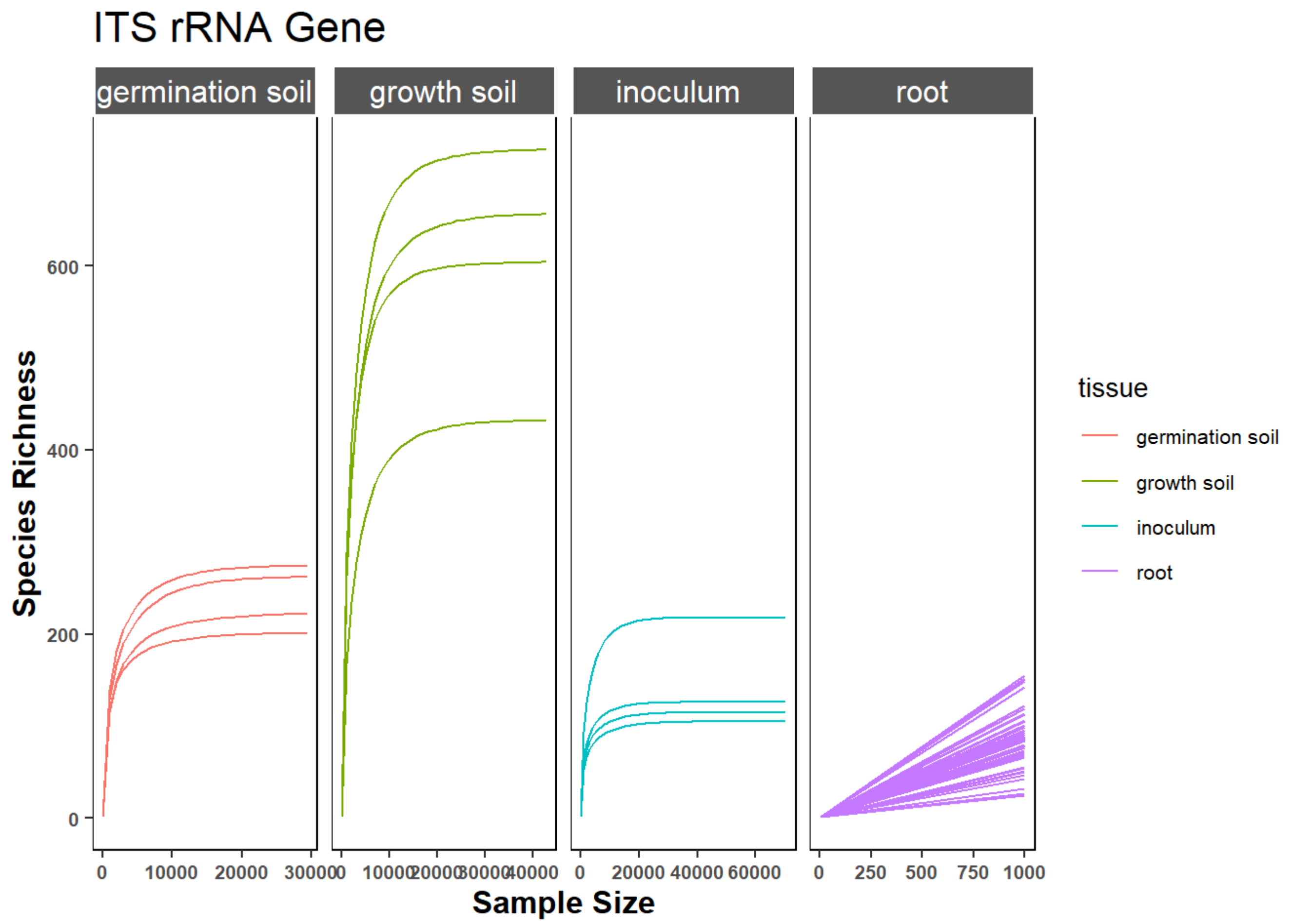


```
# Add interval classification for root tissue
sample_data(merged_phylo)$interval_ratioroot <- as.factor(
  ifelse(sample_data(merged_phylo)$ratioroot == "", NA,  # If the cell is empty, assign NA
         ifelse(sample_data(merged_phylo)$ratioroot < 0.80, "-",  # If <0.80, assign "-"
                ifelse(sample_data(merged_phylo)$ratioroot > 1.20, "+", "/")))  # If >1.20, assign "+"
)
```

17.4 - Figure S6D - Root-associated fungal phylum composition

```
#1. inoculum, germination soil and growth soil

# Subset data: Exclude 'root' tissue samples
rITS_S_I <- subset_samples(merged_phylo, tissue != "root")

# Agglomerate data at the Phylum level, transform to relative abundance, and melt to
long format
erie_Phylum <- rITS_S_I %>%
  tax_glom(taxrank = "Phylum") %>%                     # Agglomerate at the Phylum le
vel
  transform_sample_counts(function(x) { x / sum(x) }) %>% # Transform to relative abu
ndance
  psmelt() %>%                                         # Melt to long format
  # Uncomment the next line to filter out low abundance taxa
  # filter(Abundance > 0.01) %>%                        # Filter out taxa with low ab
undance
  arrange(Phylum)                                      # Sort by Phylum

# Convert relevant columns to factors for better plotting
erie_Phylum$Sample <- as.factor(erie_Phylum$Sample)
erie_Phylum$tissue <- as.factor(erie_Phylum$tissue)

# Define custom colors for Phyla
phylum_colors <- c("p__Aphelidiomycota" = "#FFC0CB",
                   "p__Ascomycota" = "#5F7FC7",
                   "p__Basidiobolomycota" = "orange",
                   "p__Basidiomycota" = "#DA5724",
                   "p__Chytridiomycota" = "#CD9BCD",
                   "p__Fungi_phy_Incertae_sedis" = "#AD6F3B",
                   "p__Glomeromycota" = "#673770",
                   "p__Mortierellomycota" = "#C84248",
                   "p__Mucoromycota" = "#8569D5",
                   "p__Olpidiomycota" = "#CBD588",
                   "p__Rozellomycota" = "#D1A33D",
                   "p__Calcarisporillomycota" = "#32CD32")

# Create the bar plot
ggplot(erie_Phylum, aes(x = Sample, y = Abundance, fill = Phylum)) +
  facet_grid(~ tissue, scales = 'free') +                    # Separate plots by tiss
ue type
  geom_bar(stat = "identity") +                               # Bar plot
  scale_fill_manual(values = phylum_colors) +                 # Apply custom colors
  theme(
    axis.title.x = element_text(),                            # Customize X-axis titl
e
    axis.title.y = element_text(size = 11, face = "bold"),    # Y-axis title
    plot.title = element_text(size = 12, face = "bold"),      # Plot title
    axis.text.x = element_text(angle = 45, hjust = 1, size = 7)  # X-axis text (angle
d)
  ) +
  guides(fill = guide_legend(reverse = FALSE, keywidth = 1, keyheight = 1)) +  # Cust
omize legend
  ylab("Relative Abundance (Phyla > 0.01%) \n") +            # Y-axis label
  ggtitle("Landlab ITS Root-Associated Bacterial Community")  # Plot title
```

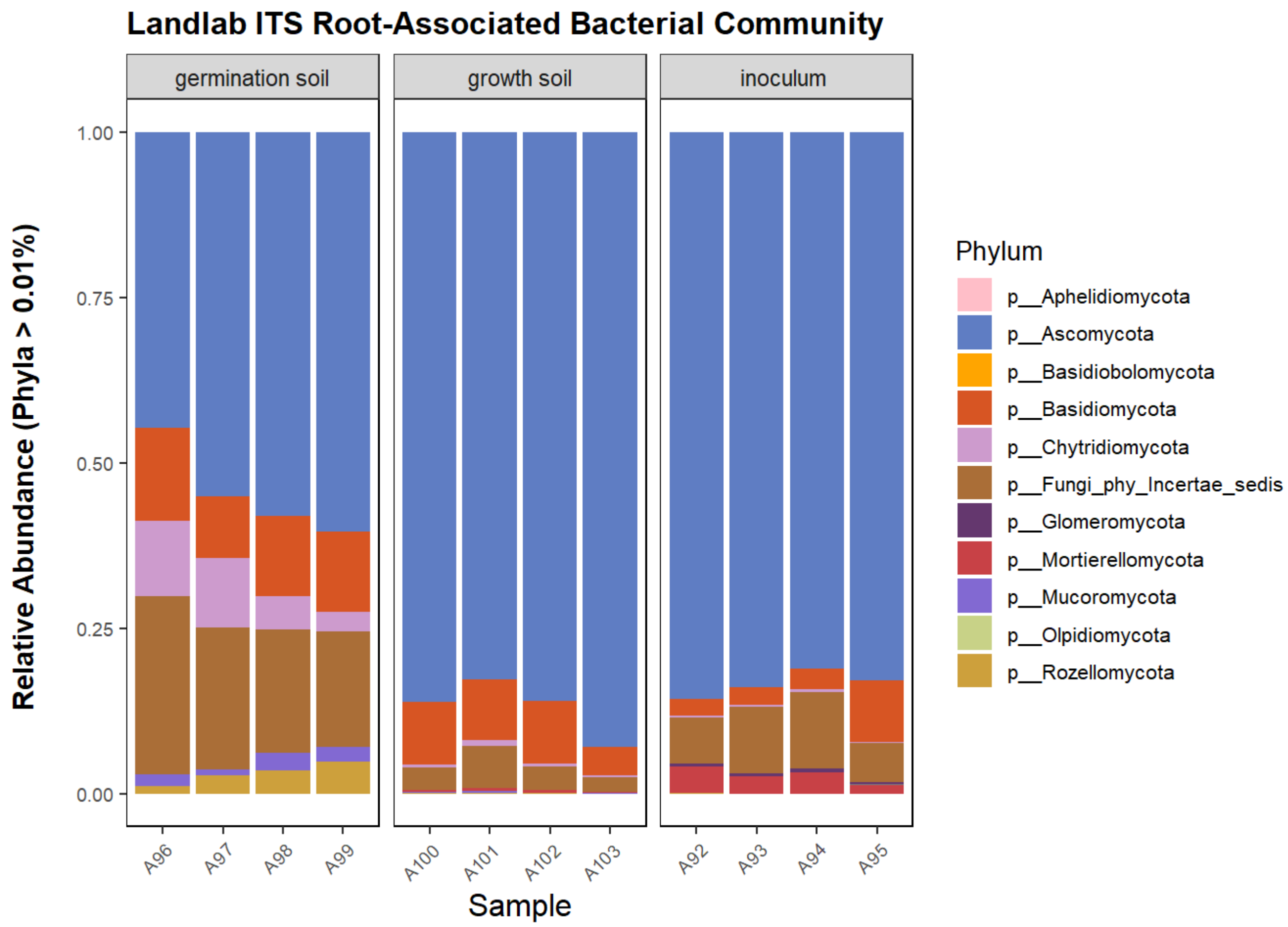
Landlab ITS Root-Associated Bacterial Community
germination soil
growth soil
inoculum
Relative Abundance (Phyla > 0.01%)
1.00
0.75
0.50
0.25
0.00
A96
A97
A98
A99
A100
A101
A102
A103
A92
A93
A94
A95
Sample
Phylum
p__Aphelidiomycota
p__Ascomycota
p__Basidiobolomycota
p__Basidiomycota
p__Chytridiomycota
p__Fungi_phy_Incertae_sedis
p__Glomeromycota
p__Mortierellomycota
p__Mucoromycota
p__Olpidiomycota
p__Rozellomycota

```
#2. Root -associated fungal community

# Subset data for 'root' tissue samples
rITS_root <- subset_samples(merged_phylo, tissue == "root")

# Agglomerate at the Phylum level, transform to relative abundance, and melt to long
format
erie_phylum <- rITS_root %>%
  tax_glom(taxrank = "Phylum") %>%                          # Agglomerate at the Phylum le
vel
  transform_sample_counts(function(x) { x / sum(x) }) %>% # Transform to relative abu
ndance
  psmelt() %>%                                              # Melt data to long format
  filter(Abundance > 0) %>%                                 # Filter out taxa with zero ab
undance
  arrange(Phylum)                                           # Sort by Phylum

# Convert columns to factors for better plotting
erie_phylum$Sample <- as.factor(erie_phylum$Sample)
erie_phylum$tissue <- as.factor(erie_phylum$tissue)
erie_phylum$Phylum <- as.factor(erie_phylum$Phylum)

# Define custom colors for different Phyla
phylum_colors <- c(
  "p__Aphelidiomycota" = "#FFC0CB",
  "p__Ascomycota" = "#5F7FC7",
  "p__Basidiobolomycota" = "orange",
  "p__Basidiomycota" = "#DA5724",
  "p__Chytridiomycota" = "#CD9BCD",
  "p__Fungi_phy_Incertae_sedis" = "#AD6F3B",
  "p__Glomeromycota" = "#673770",
  "p__Mortierellomycota" = "#C84248",
  "p__Mucoromycota" = "#8569D5",
  "p__Olpidiomycota" = "#CBD588",
  "p__Rozellomycota" = "#D1A33D",
  "p__Calcarisporillomycota" = "#32CD32"
)

# Create the bar plot
ggplot(erie_phylum, aes(x = Sample, y = Abundance, fill = Phylum)) +
  geom_bar(stat = "identity") +                               # Create bar plot
  scale_fill_manual(values = phylum_colors) +                  # Apply custom colors
  theme(
    axis.title.x = element_text(),                             # Customize X-axis title
    axis.title.y = element_text(size = 11, face = "bold"),  # Y-axis title with bold
text
    plot.title = element_text(size = 12, face = "bold"),    # Plot title with bold te
xt
    axis.text.x = element_text(angle = 45, hjust = 1, size = 7)  # Rotate X-axis text
labels
  ) +
  guides(fill = guide_legend(reverse = FALSE, keywidth = 1, keyheight = 1)) +  # Cust
omize legend
  ylab("Relative Abundance (Phyla > 0.01%) \n") +            # Y-axis label
  ggtitle("Landlab ITS Root-Associated Bacterial Community")  # Plot title
```

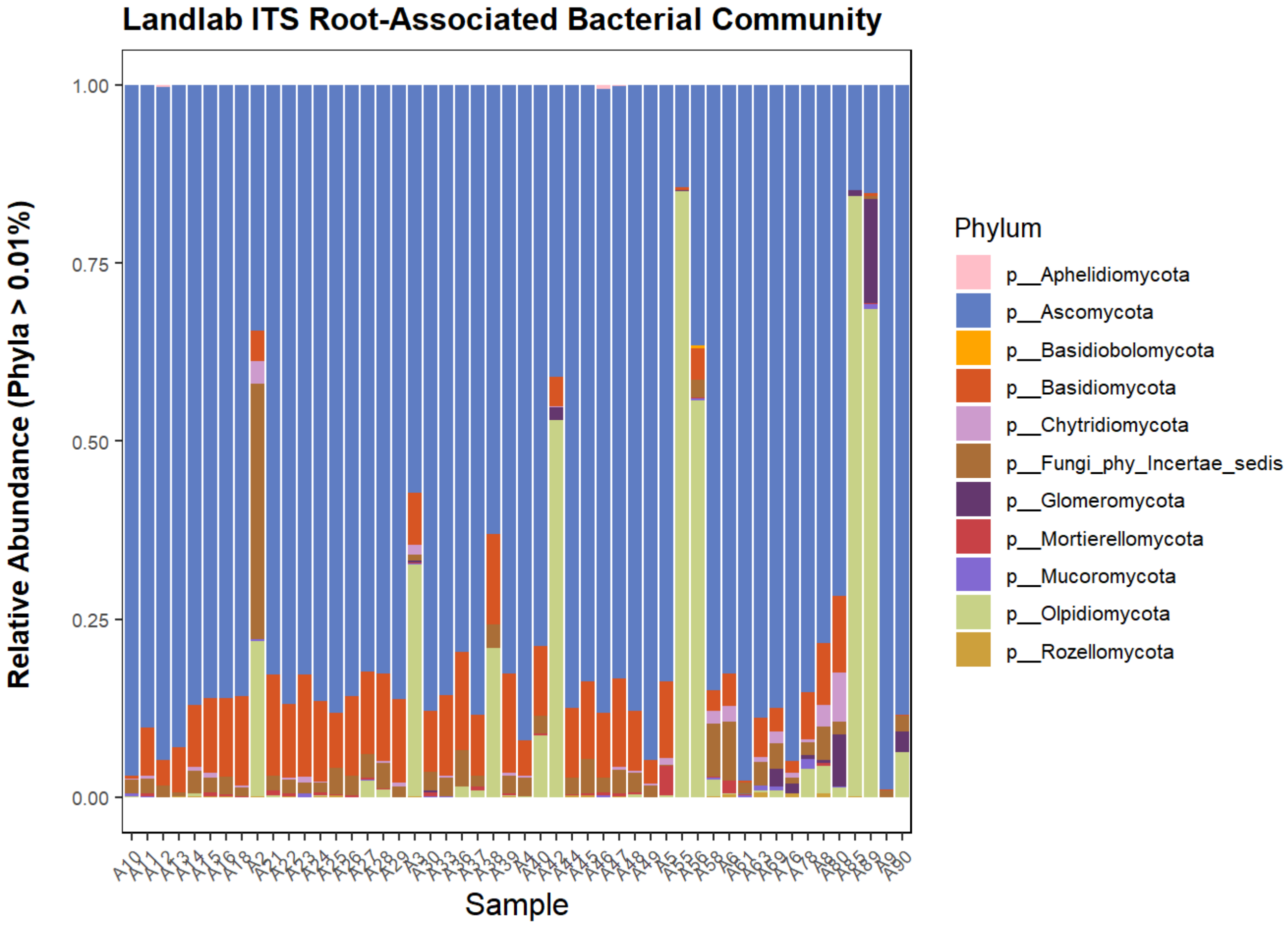


```
####################Supplementary table 18

# Calculate Relative Abundance for Germination Soil

# Aggregate data by Phylum and calculate relative abundance for germination soil
erie_phylum <- merged_phylo %>%
  tax_glom(taxrank = "Phylum") %>%                     # Agglomerate at Phylum level
  transform_sample_counts(function(x) {x / sum(x) * 100}) %>% # Calculate relative ab
undance in percentage
  psmelt() %>%                                         # Convert to long format
  filter(tissue == "germination soil") %>%              # Change with the other tissu
es (inoculum, root, growth soil)
  arrange(Phylum)                                      # Sort by Phylum



# Identify the top Phyla by mean relative abundance
top_phyla <- erie_phylum %>%
  group_by(Phylum) %>%
  summarize(mean_abundance = round(mean(Abundance), 1)) %>%  # Calculate mean and rou
nd to 1 decimal place
  arrange(desc(mean_abundance))  # Sort by descending mean abundance
top_phyla
```

Phylum<br><chr>	mean_abundance<br><dbl>
p__Ascomycota	54.6

Phylum <chr>	mean_abundance <dbl>
p__Fungi_phy_Incertae_sedis	21.1
p__Basidiomycota	11.9
p__Chytridiomycota	7.5
p__Rozellomycota	3.1
p__Mucoromycota	1.9
p__Aphelidiomycota	0.0
p__Basidiobolomycota	0.0
p__Calcarisporiellomycota	0.0
p__GS01_phy_Incertae_sedis	0.0

1-10 of 14 rows Previous **1** 2 Next

17.5 - alpha diversity

```
# Load necessary libraries
library(picante)
library(tidyverse)
library(ggpubr)
library(rstatix)
library(ggthemes)
library(multcompView)
library(agricolae)
library(dplyr)

# Table of alpha-diversity estimators (using Observed, InvSimpson, and Shannon indice
s)
table_rITS <- estimate_richness(merged_phylo, split = TRUE, measures = c("Observed",
"InvSimpson", "Shannon"))

# Bind design + table of alpha-diversity results
sdrITS <- sample_data(merged_phylo)
datarITS <- cbind(sample_data(sdrITS), table_rITS)

# Transform relevant columns to factors
datarITS$genotype <- as.factor(datarITS$genotype)
datarITS$tissue <- as.factor(datarITS$tissue)
datarITS$Treatment <- as.factor(datarITS$Treatment)
datarITS$variety <- as.factor(datarITS$variety)
datarITS$genetic.distance <- as.factor(datarITS$genetic.distance)
datarITS$interval_response <- as.factor(datarITS$interval_response)
datarITS$Pi.response <- as.factor(datarITS$Pi.response)
datarITS$Shoot.response <- as.factor(datarITS$Shoot.response)
```

17.6 - Figure S6A-B (upper part)

```
# Define a color palette for compartments
compartment_palette <- c("#a78b4d", "#cfb26e", "#e1cba8", "#cc742c")

#1. Perform Dunn's test for Observed diversity between tissues, with FDR correction
res_obs <- datarITS %>%
  dunn_test(Observed ~ tissue, p.adjust.method = "fdr")

# Add statistical test results to plot
stat.test <- res_obs %>% add_xy_position(x = "tissue")

# Create a boxplot for Observed ASV richness with jittered points
plot_ITS_Observed_b <- ggplot(datarITS, aes(tissue, Observed)) +
  geom_boxplot(aes(fill = tissue), show.legend = TRUE, outlier.shape = NA) +  # Hide
outliers
  geom_jitter(size = 3) +  # Add jittered points for better visibility
  labs(x = "Tissue Type", y = "Observed ASV Richness") +  # Label axes
  theme_bw() +  # Use a clean theme
  theme(panel.grid.major = element_blank(), panel.grid.minor = element_blank()) +  #
Remove grid lines
  stat_pvalue_manual(stat.test, hide.ns = TRUE) +  # Display p-values, hide non-signi
ficant results
  scale_fill_manual(values = compartment_palette)  # Apply custom color palette

# Display the plot
plot_ITS_Observed_b
```

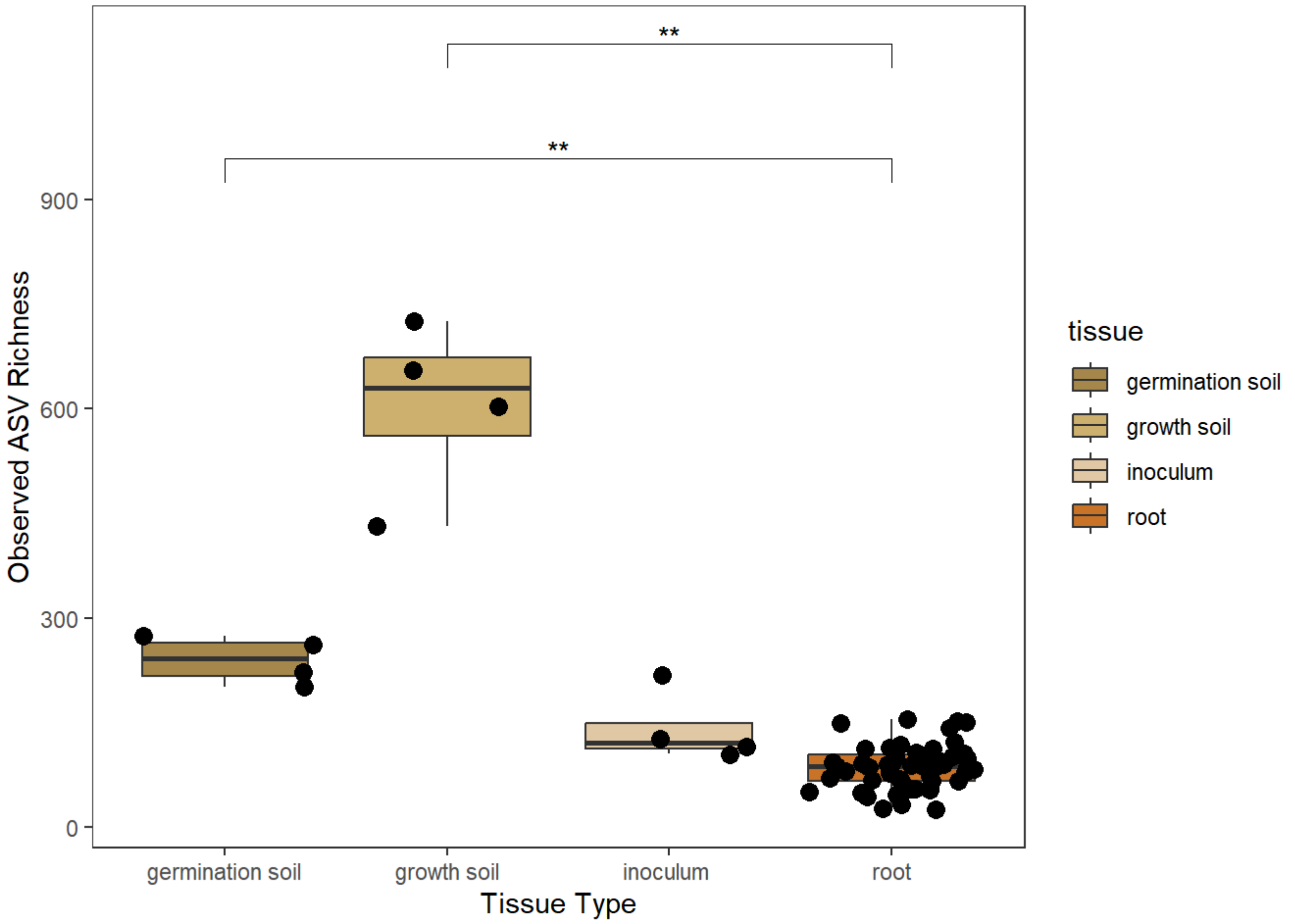

```
#2. Perform Dunn's test for Shannon diversity between tissues, with FDR correction
res_shan <- datarITS %>%
  dunn_test(Shannon ~ tissue, p.adjust.method = "fdr")

# Add statistical test results to plot
stat.test <- res_shan %>% add_xy_position(x = "tissue")

# Create a boxplot for Shannon ASV richness with jittered points
plot_ITS_Shannon_b <- ggplot(datarITS, aes(tissue, Shannon)) +
  geom_boxplot(aes(fill = tissue), show.legend = TRUE, outlier.shape = NA) +  # Hide
outliers
  geom_jitter(size = 3) +  # Add jittered points
  labs(x = "Tissue Type", y = "Shannon ASV Richness") +  # Label axes
  theme_bw() +  # Use a clean theme
  theme(panel.grid.major = element_blank(), panel.grid.minor = element_blank()) +  #
Remove grid lines
  stat_pvalue_manual(stat.test, hide.ns = TRUE) +  # Display p-values
  scale_fill_manual(values = compartment_palette)  # Apply custom color palette

# Display the plot
plot_ITS_Shannon_b
```

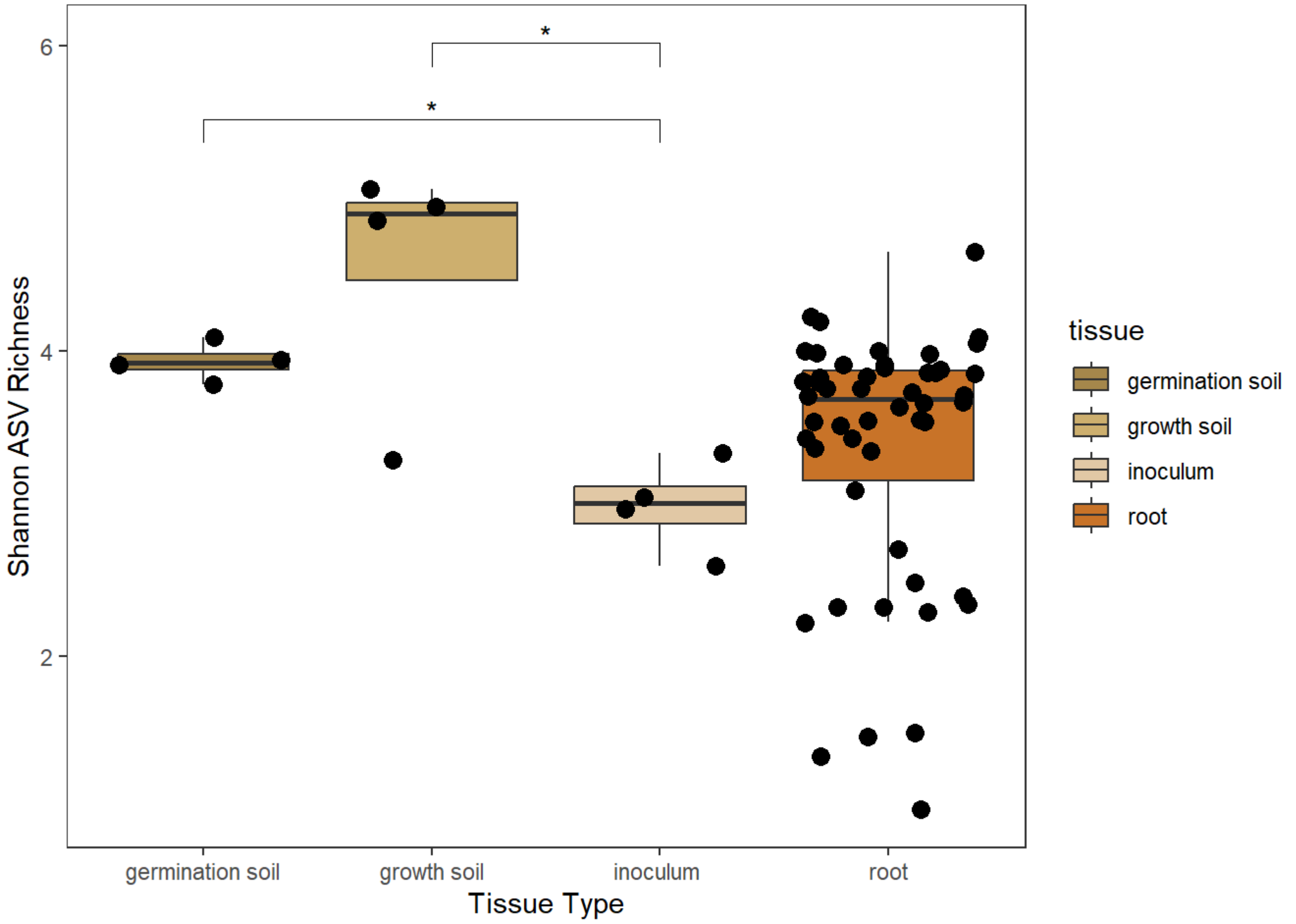

17.7 - Figure S6A-B (lower part)

```
# Subset the dataset for the root tissue and specific treatments (T and CTRL)
datarITS_root <- datarITS %>%
  filter(tissue == "root") %>%
  filter(Treatment == "T" | Treatment == "CTRL") %>%
  droplevels()

# Define a color palette for treatments
paletteT <- c("gray90", "#cc742c")

# Convert relevant variables to factors for appropriate statistical analysis
datarITS_root$genotype <- as.factor(datarITS_root$genotype)
datarITS_root$tissue <- as.factor(datarITS_root$tissue)
datarITS_root$Treatment <- as.factor(datarITS_root$Treatment)
datarITS_root$interval_response <- as.factor(datarITS_root$interval_response)
datarITS_root$variety <- as.factor(datarITS_root$variety)
datarITS_root$genetic.distance <- as.factor(datarITS_root$genetic.distance)
datarITS_root$phenotypic_group <- as.factor(datarITS_root$phenotypic_group)

# Perform Dunn's test for observed ASV richness between treatments with FDR correctio
n
res_obs <- datarITS_root %>%
  dunn_test(Observed ~ Treatment, p.adjust.method = "fdr")

# Add statistical test results to plot
stat.test_obs <- res_obs %>% add_xy_position(x = "Treatment")

# Create a boxplot for observed ASV richness with jittered points
plot_ITS_Observed_b <- ggplot(datarITS_root, aes(Treatment, Observed)) +
  geom_boxplot(aes(fill = Treatment), show.legend = TRUE, outlier.shape = NA) +  # Hi
de outliers
  geom_jitter(size = 3) +  # Add jittered points for better visibility
  labs(x = "Treatment", y = "Observed ASV Richness") +  # Label axes
  theme_bw() +  # Use a clean theme
  theme(panel.grid.major = element_blank(), panel.grid.minor = element_blank()) +  #
Remove grid lines
  stat_pvalue_manual(stat.test_obs, hide.ns = TRUE) +  # Display p-values, hide non-s
ignificant results
  scale_fill_manual(values = paletteT)  # Apply custom color palette

# Display the plot for observed ASV richness
plot_ITS_Observed_b
```

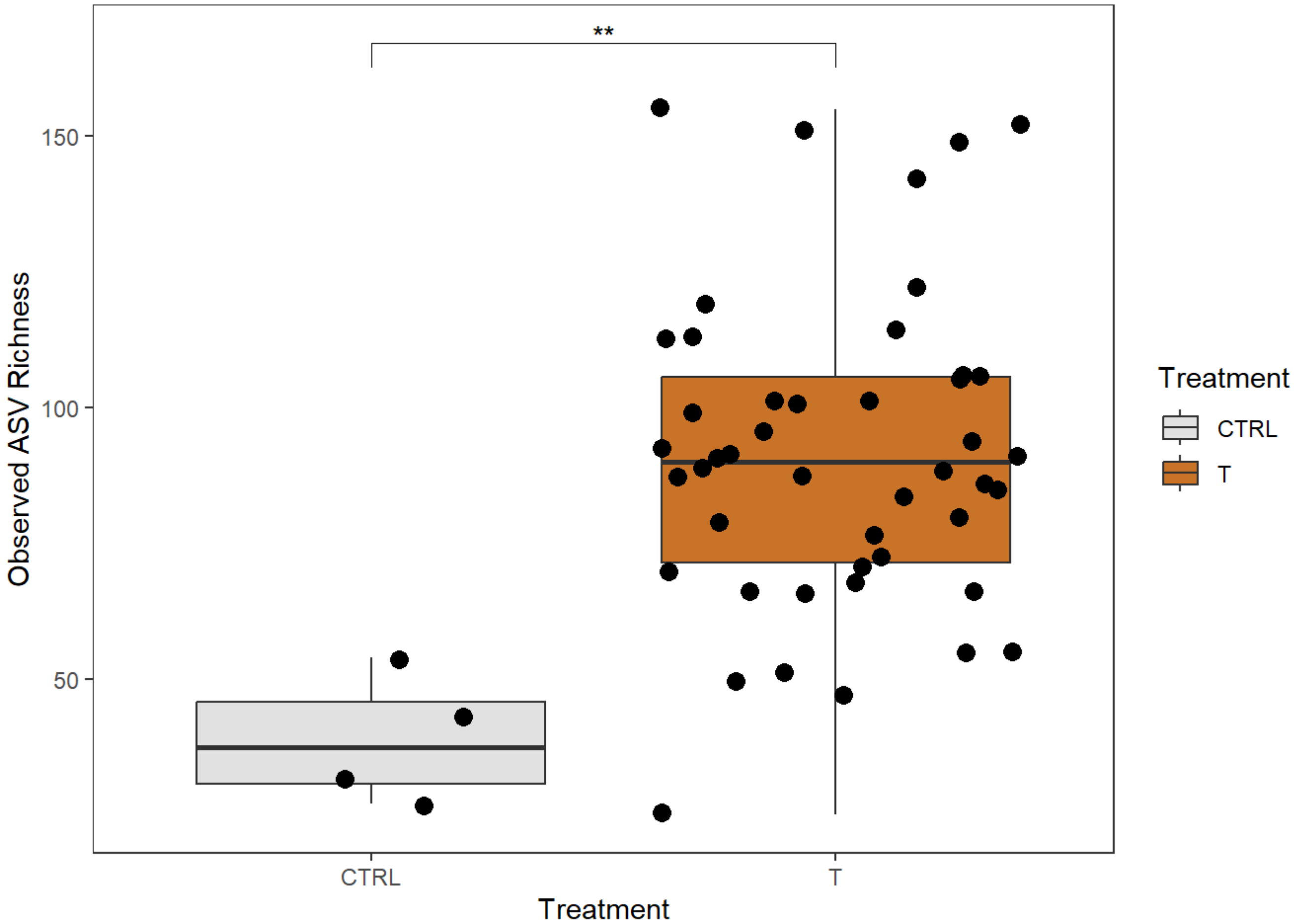


```
# Perform Dunn's test for Shannon diversity between treatments with FDR correction
res_shan <- datarITS_root %>%
  dunn_test(Shannon ~ Treatment, p.adjust.method = "fdr")

# Add statistical test results to plot
stat.test_shan <- res_shan %>% add_xy_position(x = "Treatment")

# Create a boxplot for Shannon ASV richness with jittered points
plot_ITS_Shannon_b <- ggplot(datarITS_root, aes(Treatment, Shannon)) +
  geom_boxplot(aes(fill = Treatment), show.legend = TRUE, outlier.shape = NA) +  # Hi
de outliers
  geom_jitter(size = 3) +  # Add jittered points
  labs(x = "Treatment", y = "Shannon ASV Richness") +  # Label axes
  theme_bw() +  # Use a clean theme
  theme(panel.grid.major = element_blank(), panel.grid.minor = element_blank()) +  #
Remove grid lines
  stat_pvalue_manual(stat.test_shan, hide.ns = TRUE) +  # Display p-values
  scale_fill_manual(values = paletteT)  # Apply custom color palette

# Display the plot for Shannon ASV richness
plot_ITS_Shannon_b
```

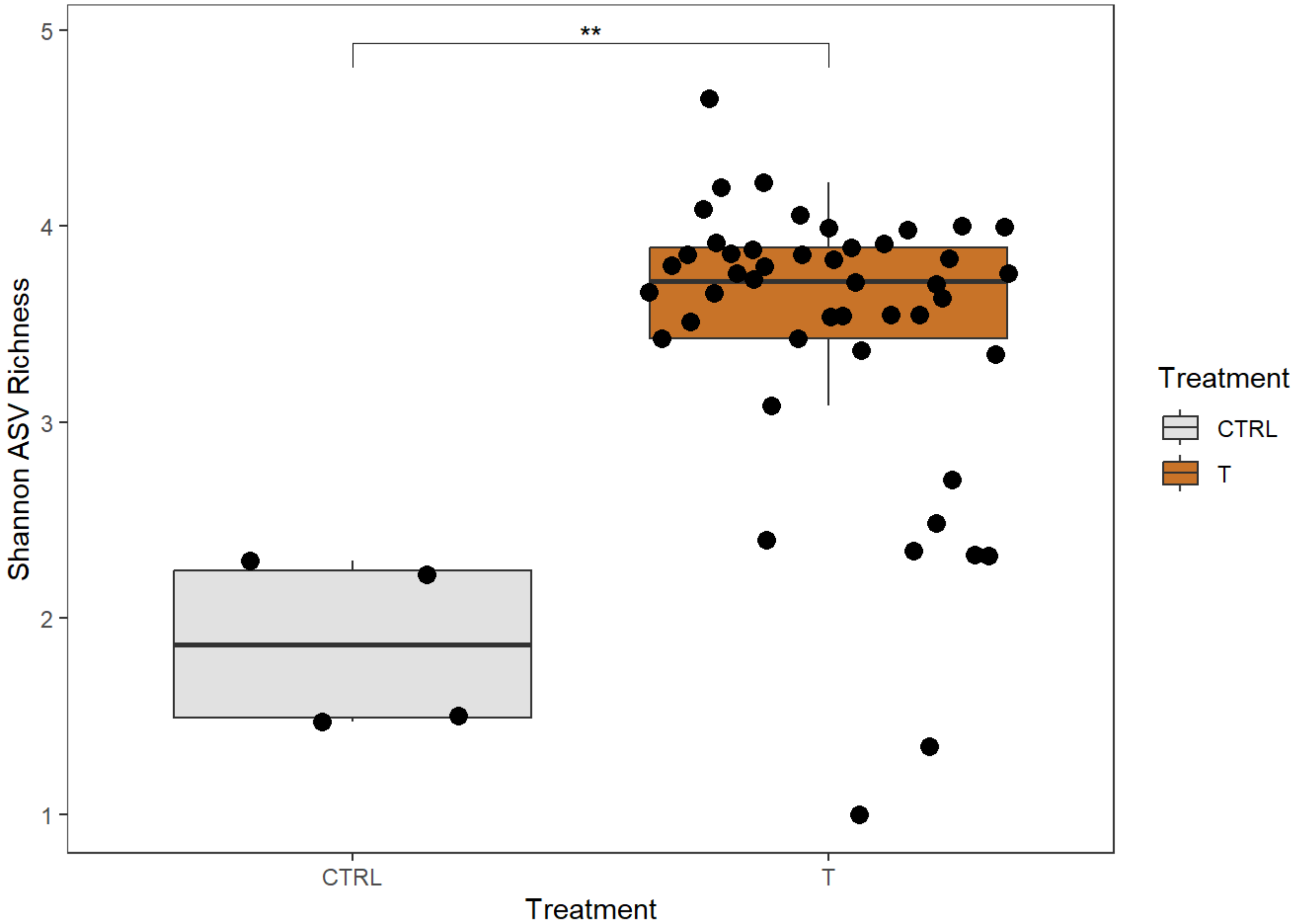

17.8 - Figure S8A - alpha diversity across phenotypic groups

```
# Filter the dataset for the 'T' treatment and specific phenotypic groups (1-4)
datarITS_root_pheno <- datarITS_root %>%
  filter(Treatment == "T") %>%
  filter(phenotypic_group %in% c("1", "2", "3", "4")) %>%
  droplevels()

#color palette for phenotypic group
genotype_colors <- c(
  "1" = "#00BFFF",   # gruppo 1
  "2" = "#00FF00",   # gruppo 2
  "3" = "#FF1493",   # gruppo 3
  "4" = "#9400D3"    # gruppo 4
)

# 1. Shannon alpha diversity

# Perform Dunn's test for Shannon diversity across phenotypic groups, with FDR correc
tion
res_obs <- dunn_test(Shannon ~ phenotypic_group, data = datarITS_root_pheno, p.adjus
t.method = "fdr")
stat.test <- res_obs %>% add_xy_position(x = "phenotypic_group")

# Convert 'phenotypic_group' to a factor
datarITS_root_pheno$phenotypic_group <- as.factor(datarITS_root_pheno$phenotypic_grou
p)

# Create a plot to visualize Shannon diversity across phenotypic groups
plot_interval_response_shannon <- ggplot(datarITS_root_pheno, aes(x = phenotypic_grou
p, y = Shannon)) +
  geom_jitter(aes(fill = phenotypic_group),
              width = 0.2,        # Slight jitter along the x-axis
              height = 0,         # No jitter along the y-axis
              size = 7,           # Point size
              shape = 21) +       # Point type (filled)
  stat_summary(fun = median,
               geom = "crossbar",
               width = 0.7,       # Crossbar width
               size = 0.6,        # Crossbar thickness
               color = "black") +  # Crossbar color
  labs(x = "Phenotypic Response", y = "Shannon Index") +  # Axis labels
  scale_fill_manual(values = genotype_colors) +  # Custom color palette
  stat_pvalue_manual(stat.test, hide.ns = TRUE) +  # Display p-values, hide non-signi
ficant results
  theme_bw() +  # Clean theme
  theme(panel.grid.major = element_blank(),
        panel.grid.minor = element_blank(),
        legend.title = element_blank(),  # Remove legend title
        axis.title.x = element_text(hjust = 0.5),  # Center the x-axis title
        axis.text.x = element_text(hjust = 0.5))   # Center the x-axis text

# Display the Shannon diversity plot
plot_interval_response_shannon
```

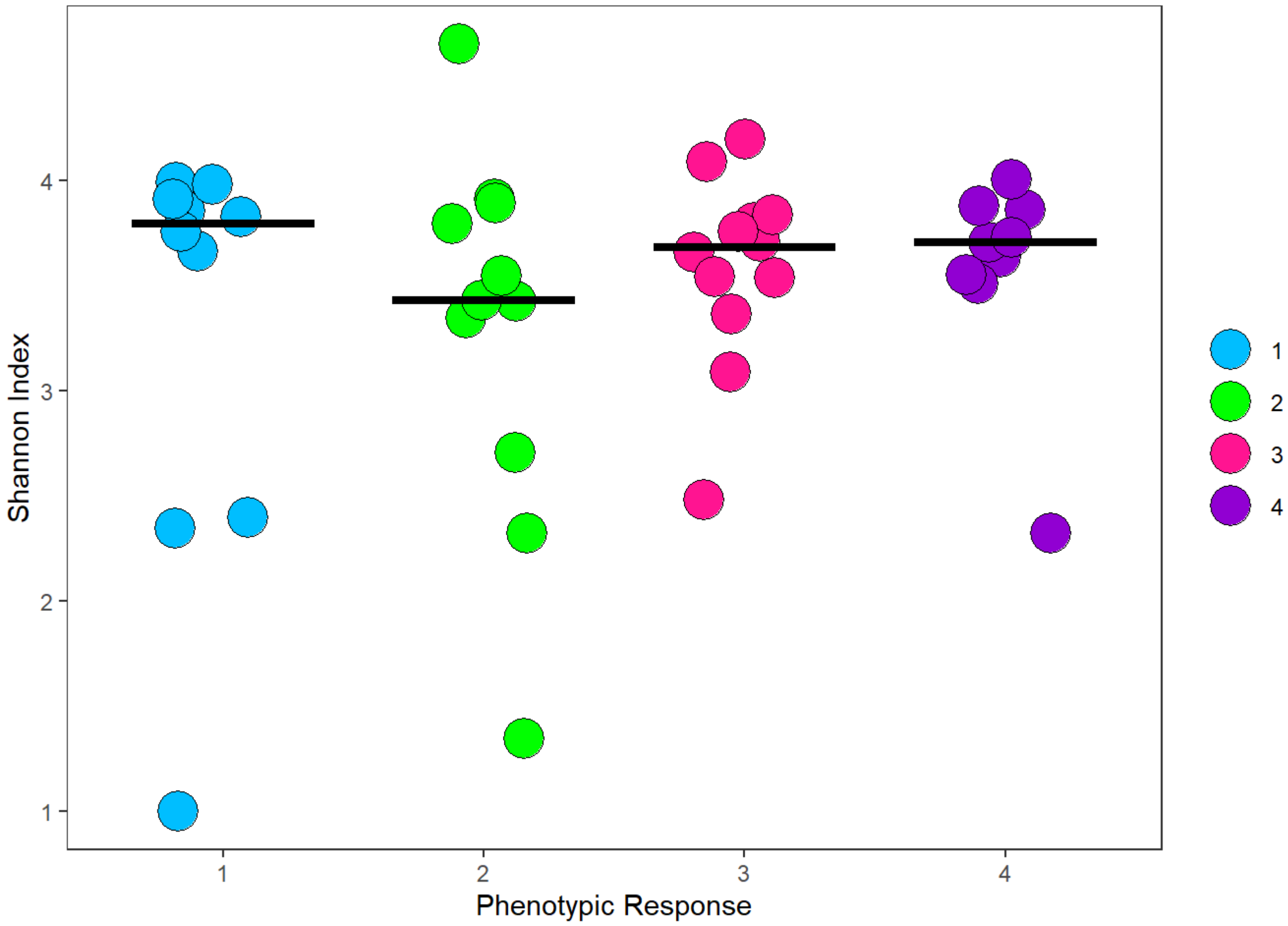
Shannon Index
4
3
2
1
1
2
3
4
Phenotypic Response
1
2
3
4

```
# 2. Observed alpha diversity

# Repeat for Observed ASV richness
datarITS_root_pheno <- datarITS_root %>%
  filter(Treatment == "T") %>%
  filter(phenotypic_group %in% c("1", "2", "3", "4")) %>%
  droplevels()

# Perform Dunn's test for Observed ASV richness across phenotypic groups, with FDR co
rrection
res_obs <- dunn_test(Observed ~ phenotypic_group, data = datarITS_root_pheno, p.adjus
t.method = "fdr")
stat.test <- res_obs %>% add_xy_position(x = "phenotypic_group")

# Convert 'phenotypic_group' to a factor
datarITS_root_pheno$phenotypic_group <- as.factor(datarITS_root_pheno$phenotypic_grou
p)

# Create a plot to visualize Observed ASV richness across phenotypic groups
plot_interval_response_observed <- ggplot(datarITS_root_pheno, aes(x = phenotypic_gro
up, y = Observed)) +
  geom_jitter(aes(fill = phenotypic_group),
              width = 0.2,        # Slight jitter along the x-axis
              height = 0,         # No jitter along the y-axis
              size = 7,           # Point size
              shape = 21) +       # Point type (filled)
  stat_summary(fun = median,
               geom = "crossbar",
               width = 0.7,       # Crossbar width
               size = 0.6,        # Crossbar thickness
               color = "black") +  # Crossbar color
  labs(x = "Phenotypic Response", y = "Observed Index") +  # Axis labels
  scale_fill_manual(values = genotype_colors) +  # Custom color palette
  stat_pvalue_manual(stat.test, hide.ns = TRUE) +  # Display p-values, hide non-signi
ficant results
  theme_bw() +  # Clean theme
  theme(panel.grid.major = element_blank(),
        panel.grid.minor = element_blank(),
        legend.title = element_blank(),  # Remove legend title
        axis.title.x = element_text(hjust = 0.5),  # Center the x-axis title
        axis.text.x = element_text(hjust = 0.5))   # Center the x-axis text

# Display the Observed ASV richness plot
plot_interval_response_observed
```

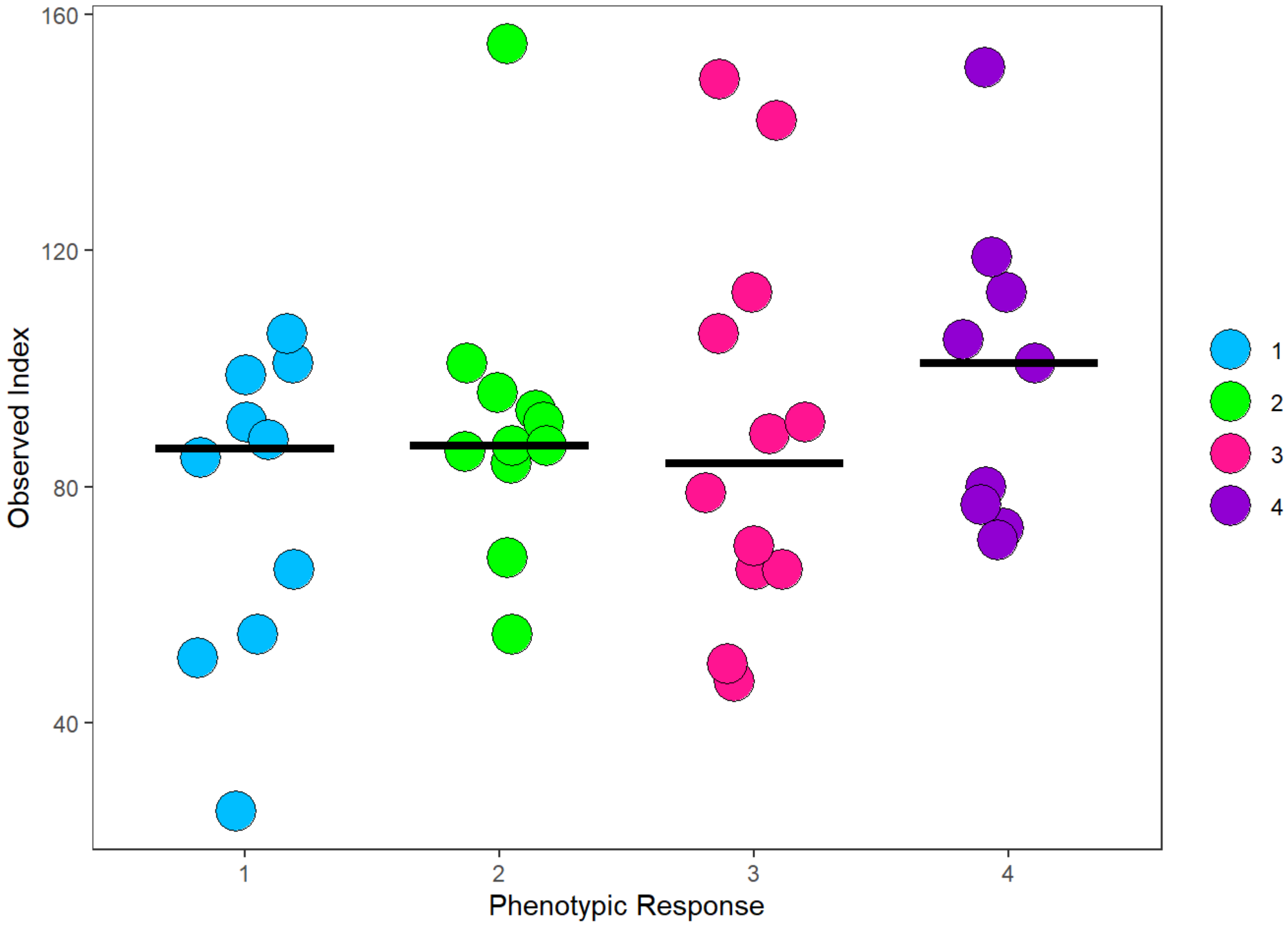

17.9 - Figure S9A-B- alpha diversity between Leaf Pi and shoot biomass response groups

```
#1. Shannon Index

# Filter data for treatment 'T' and selected interval responses
datarITS_root2 <- datarITS_root %>%
  filter(Treatment == "T") %>%
  filter(interval_response %in% c("--", "-+", "+-", "++")) %>%
  droplevels()

# Statistical analysis: Dunn's test for Shannon Index across interval responses
res_obs <- dunn_test(Shannon ~ interval_response, data = datarITS_root2, p.adjust.met
hod = "fdr")
stat.test <- res_obs %>% add_xy_position(x = "interval_response")

# Convert 'interval_response' and 'phenotypic_group' to factors
datarITS_root2$interval_response <- as.factor(datarITS_root2$interval_response)
datarITS_root2$phenotypic_group <- as.factor(datarITS_root2$phenotypic_group)

# Plot Shannon Index across interval responses
plot_interval_response_shannon <- ggplot(datarITS_root2, aes(x = interval_response, y
= Shannon)) +
  geom_jitter(aes(fill = phenotypic_group),
              width = 0.2,        # Slight jitter along the x-axis
              height = 0,         # No jitter along the y-axis
              size = 7,           # Point size
              shape = 21) +       # Filled circle shape
  stat_summary(fun = median,
               geom = "crossbar",
               width = 0.7,       # Crossbar width
               size = 0.6,        # Crossbar thickness
               color = "black") +  # Crossbar color
  labs(x = "Interval Response", y = "Shannon Index") +  # Axis labels
  scale_fill_manual(values = genotype_colors) +  # Custom color palette for phenotypi
c groups
  stat_pvalue_manual(stat.test, hide.ns = TRUE) +  # Add p-values, hide non-significa
nt results
  theme_bw() +  # Clean theme
  theme(panel.grid.major = element_blank(),
        panel.grid.minor = element_blank(),
        legend.title = element_blank(),  # Remove legend title
        axis.title.x = element_text(hjust = 0.5),  # Center x-axis title
        axis.text.x = element_text(hjust = 0.5))   # Center x-axis text

# Display Shannon Index plot
plot_interval_response_shannon
```

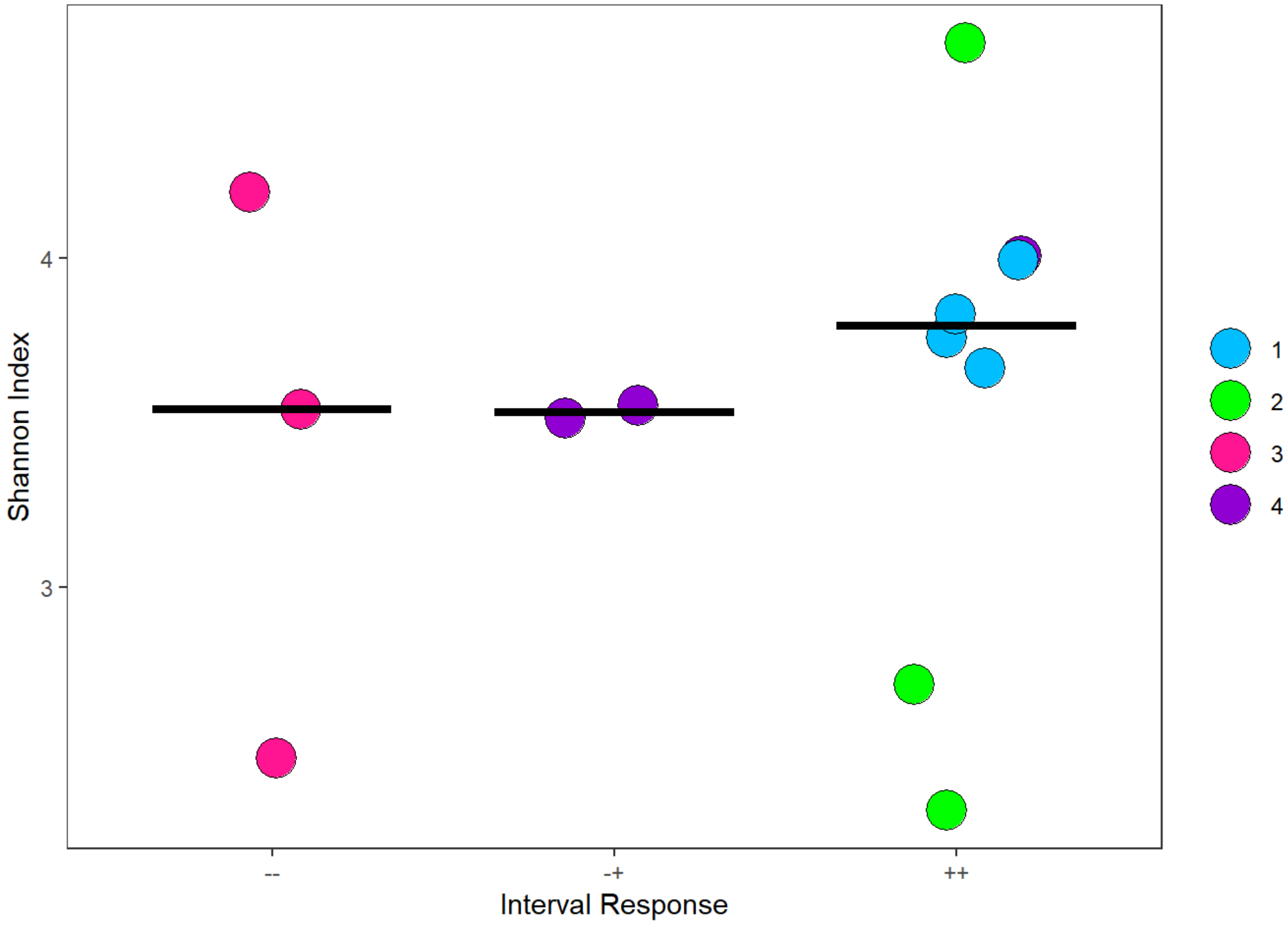
Shannon Index
4
3
--
-+
++
Interval Response
1
2
3
4

```
#2. Observed Index

# Statistical analysis: Dunn's test for Observed ASV richness across interval respons
es
res_obs <- dunn_test(Observed ~ interval_response, data = datarITS_root2, p.adjust.me
thod = "fdr")
stat.test <- res_obs %>% add_xy_position(x = "interval_response")

# Convert 'interval_response' and 'phenotypic_group' to factors
datarITS_root2$interval_response <- as.factor(datarITS_root2$interval_response)
datarITS_root2$phenotypic_group <- as.factor(datarITS_root2$phenotypic_group)

# Plot Observed ASV richness across interval responses
plot_interval_response_observed <- ggplot(datarITS_root2, aes(x = interval_response,
y = Observed)) +
  geom_jitter(aes(fill = phenotypic_group),
              width = 0.2,        # Slight jitter along the x-axis
              height = 0,         # No jitter along the y-axis
              size = 5,           # Point size
              shape = 21) +       # Filled circle shape
  stat_summary(fun = median,
               geom = "crossbar",
               width = 0.6,       # Crossbar width
               size = 0.6,        # Crossbar thickness
               color = "black") +  # Crossbar color
  labs(x = "Interval Response", y = "Observed Index") +  # Axis labels
  scale_fill_manual(values = genotype_colors) +  # Custom color palette for phenotypi
c groups
  stat_pvalue_manual(stat.test, hide.ns = TRUE) +  # Add p-values, hide non-significa
nt results
  theme_bw() +  # Clean theme
  theme(panel.grid.major = element_blank(),
        panel.grid.minor = element_blank(),
        legend.title = element_blank(),  # Remove legend title
        axis.title.x = element_text(hjust = 0.5),  # Center x-axis title
        axis.text.x = element_text(hjust = 0.5))   # Center x-axis text

# Display Observed ASV richness plot
plot_interval_response_observed
```

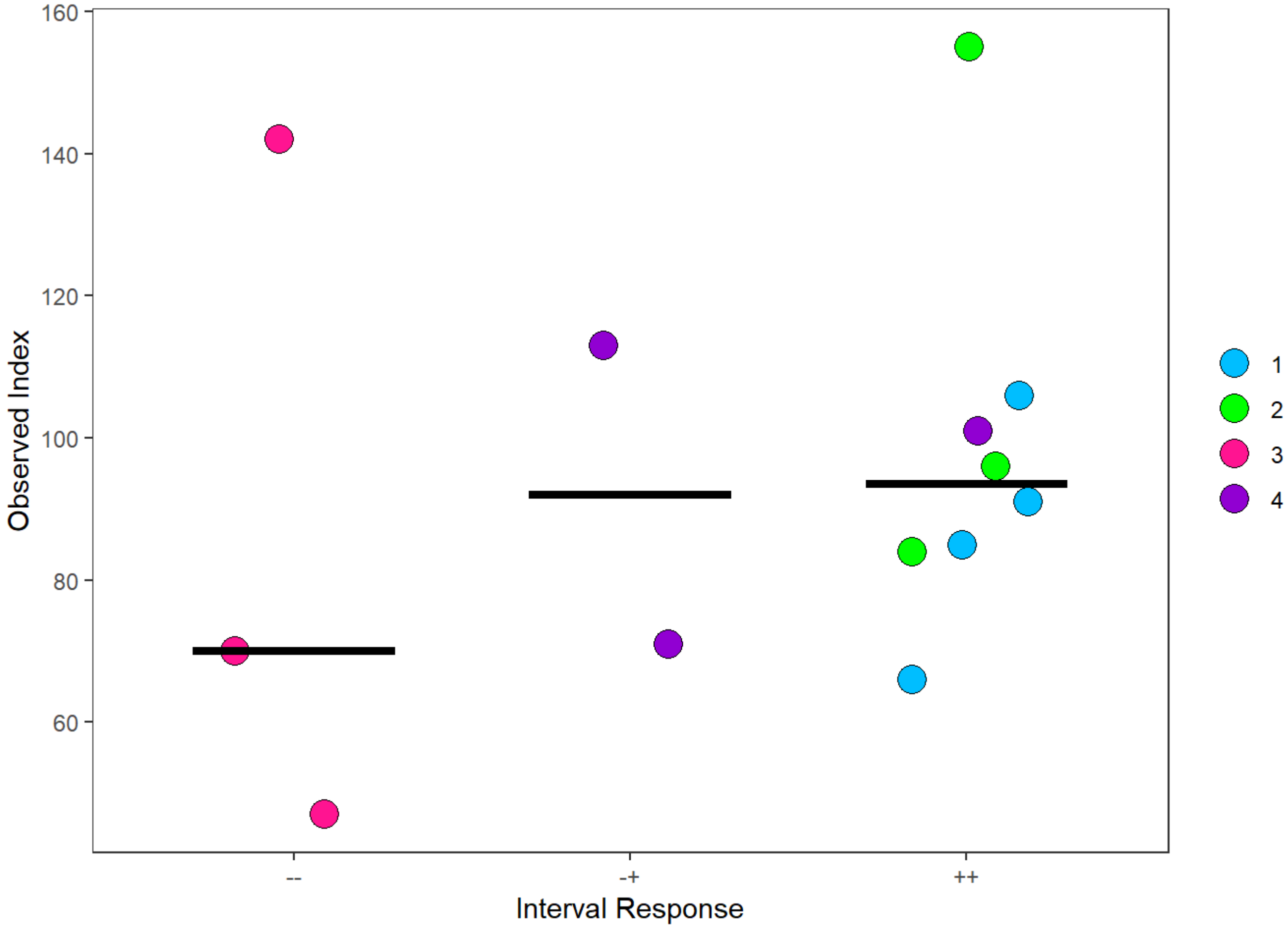

17.10 - Figure S9C - alpha diversity between Leaf Pi response groups

```
#Figure 5C
#1. Shannon Index - Pi.response

# Filter data for treatment "T" and selected Pi.response values
datarITS_root3 <- datarITS_root %>%
  filter(Treatment == "T") %>%
  filter(Pi.response %in% c("-", "+")) %>%
  droplevels()

# Perform Dunn's test for Shannon diversity index by Pi.response
res_shan <- dunn_test(Shannon ~ Pi.response, data = datarITS_root3, p.adjust.method =
"fdr")
stat.test <- res_shan %>% add_xy_position(x = "Pi.response")

# Plot Shannon Index across Pi.response groups
plot_ITS_Shannon_b <- ggplot(datarITS_root3, aes(Pi.response, Shannon)) +
  geom_boxplot(aes(fill = Pi.response), show.legend = TRUE, outlier.shape = NA) +
  geom_jitter(size = 3) +  # Add jittered points for better visualization
  labs(x = "Response Group", y = "Shannon ASV Richness") +  # Axis labels
  theme_bw() +  # Clean theme
  theme(panel.grid.major = element_blank(), panel.grid.minor = element_blank()) +  #
Remove grid lines
  stat_pvalue_manual(stat.test, hide.ns = TRUE) +  # Add statistical p-values, hiding
non-significant results
  scale_fill_manual(values = c("#ece7f2", "#2b8cbe"))  # Custom color palette

# Display Shannon Index plot
plot_ITS_Shannon_b
```

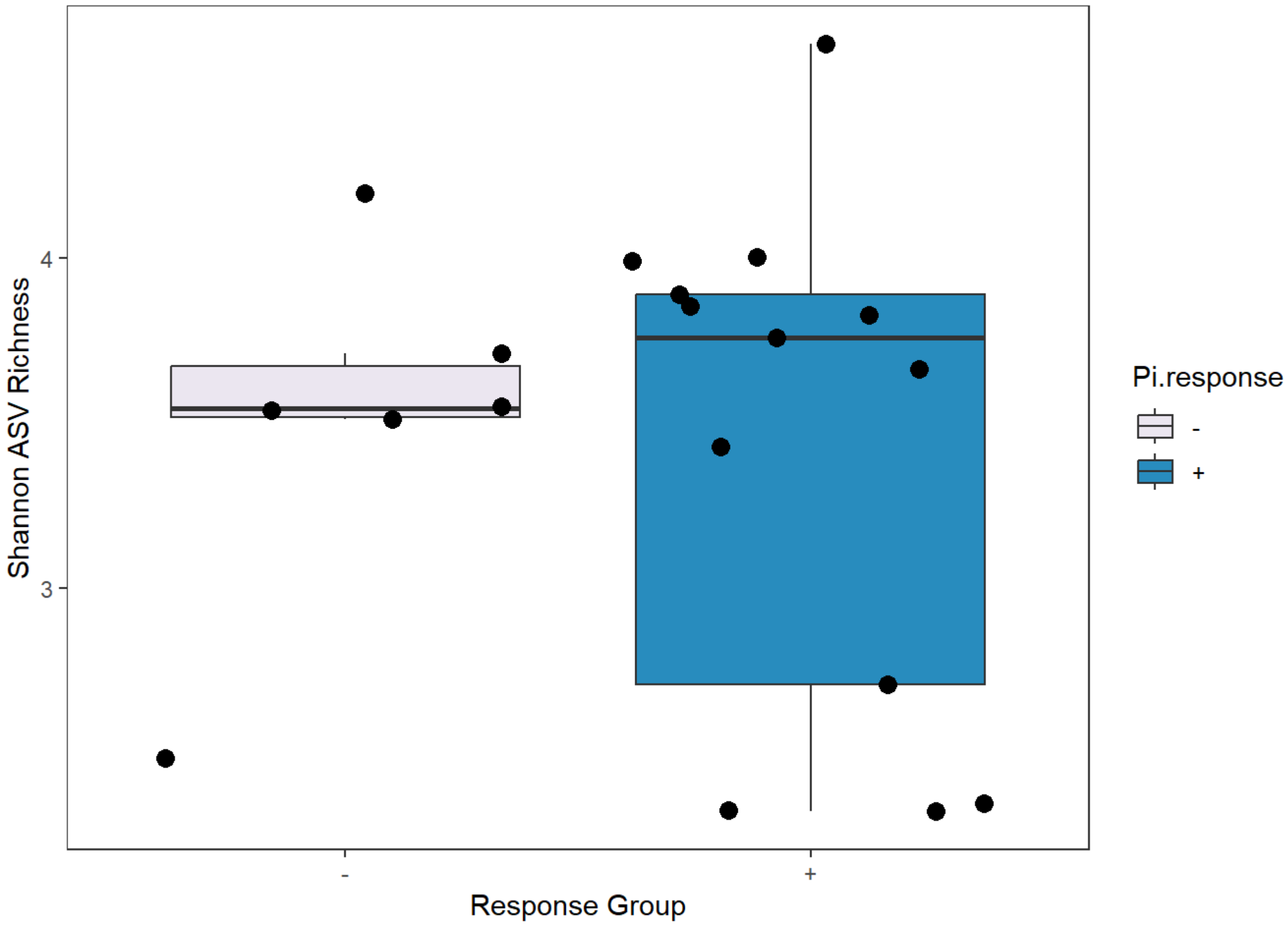

```
#Figure S10B
#2. Observed Index - Pi.response

# Transform Pi.response to a factor
datarITS_root3$Pi.response <- as.factor(datarITS_root3$Pi.response)

# Perform Dunn's test for Observed ASV richness by Pi.response
res_obs <- dunn_test(Observed ~ Pi.response, data = datarITS_root3, p.adjust.method =
"fdr")
stat.test <- res_obs %>% add_xy_position(x = "Pi.response")

# Plot Observed ASV richness across Pi.response groups
plot_ITS_Observed_b <- ggplot(datarITS_root3, aes(Pi.response, Observed)) +
  geom_boxplot(aes(fill = Pi.response), show.legend = TRUE, outlier.shape = NA) +
  geom_jitter(size = 3) +  # Add jittered points for better visualization
  labs(x = "Response Group", y = "Observed ASV Richness") +  # Axis labels
  theme_bw() +  # Clean theme
  theme(panel.grid.major = element_blank(), panel.grid.minor = element_blank()) +  #
Remove grid lines
  stat_pvalue_manual(stat.test, hide.ns = TRUE) +  # Add statistical p-values, hiding
non-significant results
  scale_fill_manual(values = c("#ece7f2", "#2b8cbe"))  # Custom color palette

# Display Observed ASV richness plot
plot_ITS_Observed_b
```

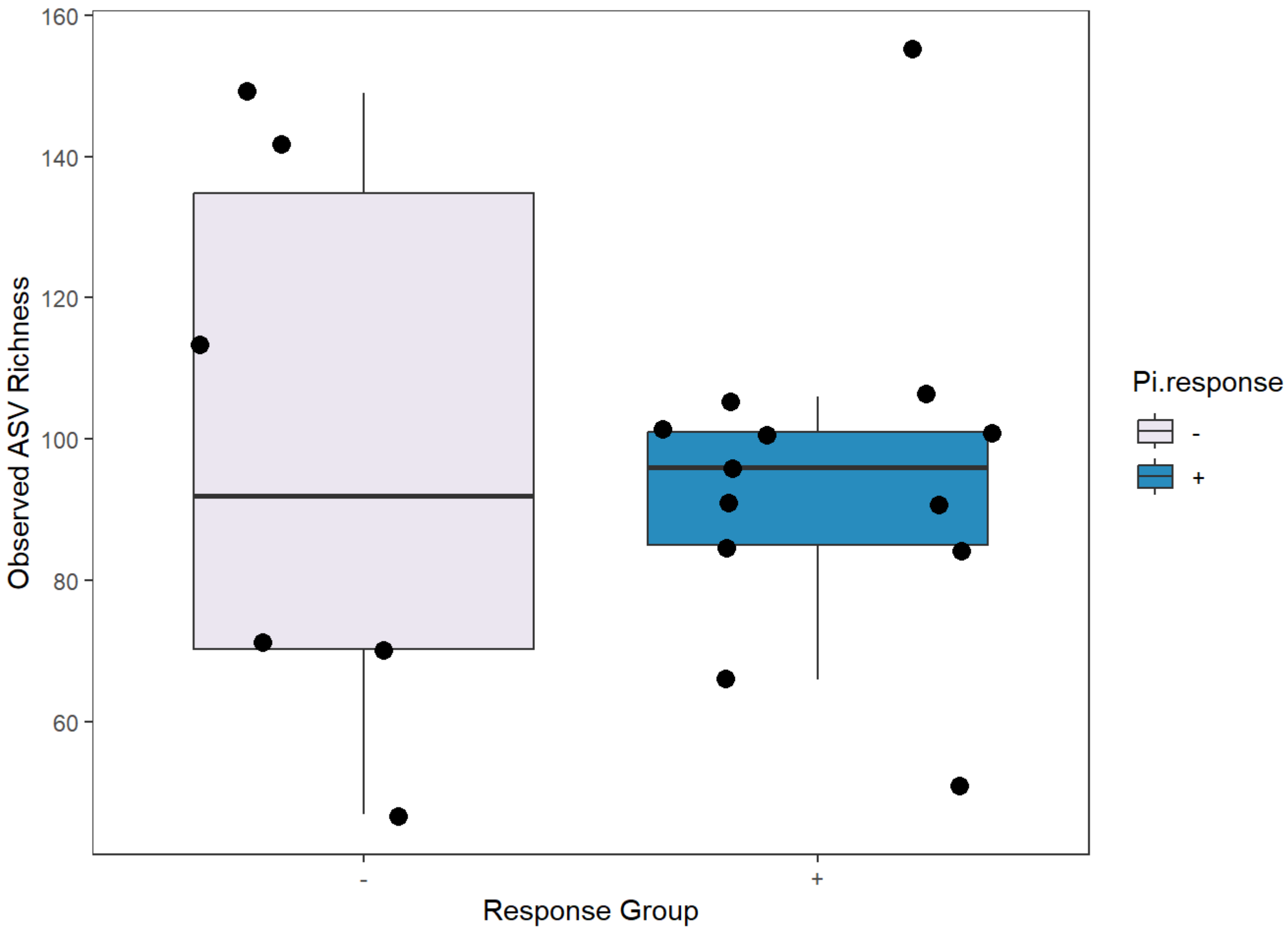

17.11 - Figure S9D - alpha diversity between Shoot response groups

```
library(ggplot2)
library(dplyr)
#1. Shannon Index - Shoot.response

# Filter data for treatment "T" and selected Shoot.response values
datarITS_root4 <- datarITS_root %>%
  filter(Treatment == "T") %>%
  filter(Shoot.response %in% c("-", "+")) %>%
  droplevels()

# Perform Dunn's test for Shannon ASV richness by Shoot.response
res_shan <- dunn_test(Shannon ~ Shoot.response, data = datarITS_root4, p.adjust.metho
d = "fdr")
stat.test <- res_shan %>% add_xy_position(x = "Shoot.response")

# Plot Shannon Index across Shoot.response groups
plot_ITS_Shannon_b <- ggplot(datarITS_root4, aes(Shoot.response, Shannon)) +
  geom_boxplot(aes(fill = Shoot.response), show.legend = TRUE, outlier.shape = NA) +
  geom_jitter(size = 3) +  # Add jittered points for better visualization
  labs(x = "Response Group", y = "Shannon ASV Richness") +  # Axis labels
  theme_bw() +  # Clean theme
  theme(panel.grid.major = element_blank(), panel.grid.minor = element_blank()) +  #
Remove grid lines
  stat_pvalue_manual(stat.test, hide.ns = TRUE) +  # Add statistical p-values, hiding
non-significant results
  scale_fill_manual(values = c("#e5f5f9", "#2ca25f"))  # Custom color palette

# Display Shannon Index plot
plot_ITS_Shannon_b
```

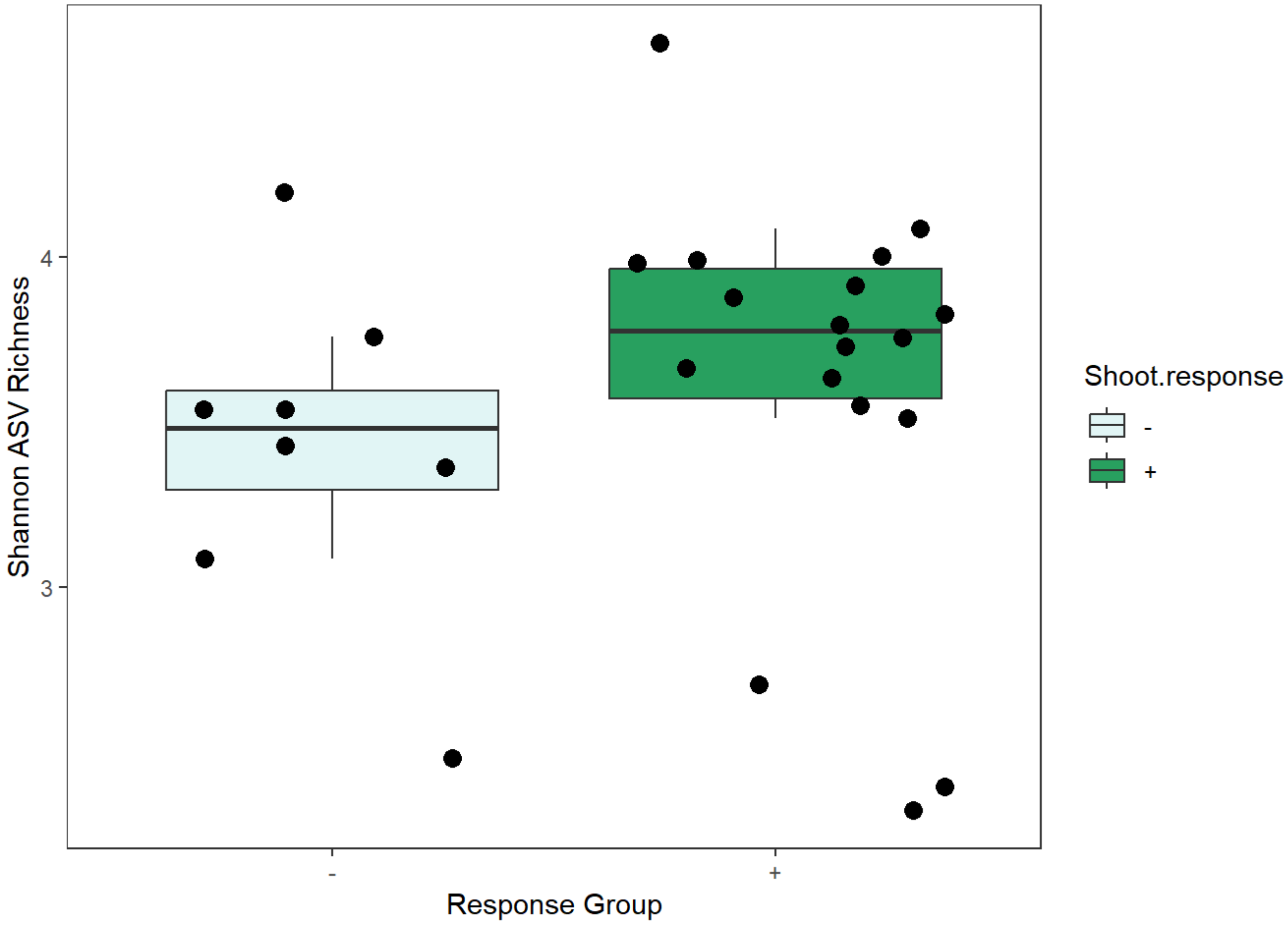


```
#2.Observed Index - Shoot.response

# Perform Dunn's test for Observed ASV richness by Shoot.response
res_obs <- dunn_test(Observed ~ Shoot.response, data = datarITS_root4, p.adjust.metho
d = "fdr")
stat.test <- res_obs %>% add_xy_position(x = "Shoot.response")

# Plot Observed ASV richness across Shoot.response groups
plot_ITS_Observed_b <- ggplot(datarITS_root4, aes(Shoot.response, Observed)) +
  geom_boxplot(aes(fill = Shoot.response), show.legend = TRUE, outlier.shape = NA) +
  geom_jitter(size = 3) +  # Add jittered points for better visualization
  labs(x = "Response Group", y = "Observed ASV Richness") +  # Axis labels
  theme_bw() +  # Clean theme
  theme(panel.grid.major = element_blank(), panel.grid.minor = element_blank()) +  #
Remove grid lines
  stat_pvalue_manual(stat.test, hide.ns = TRUE) +  # Add statistical p-values, hiding
non-significant results
  scale_fill_manual(values = c("#e5f5f9", "#2ca25f"))  # Custom color palette

# Display Observed ASV richness plot
plot_ITS_Observed_b
```

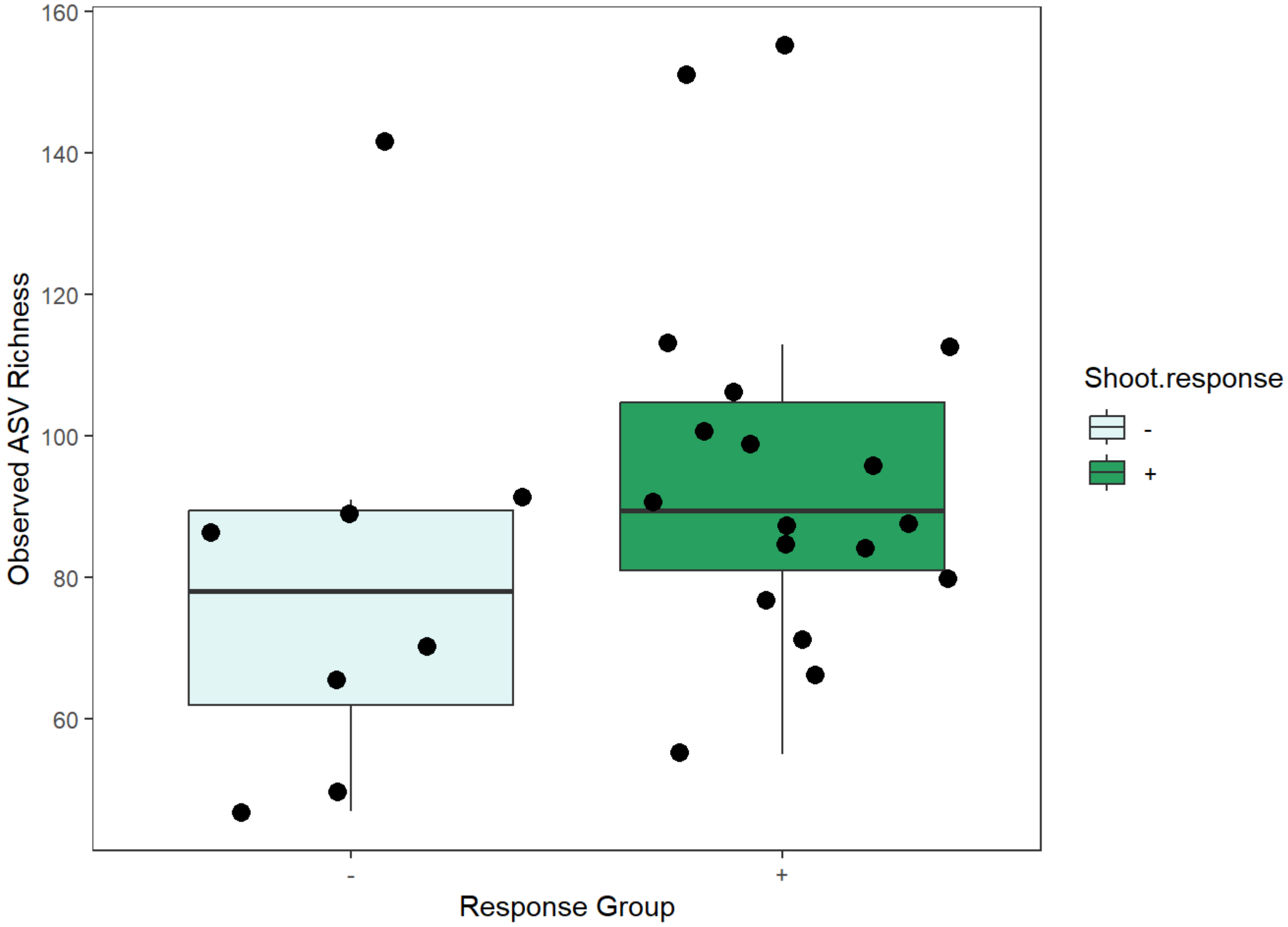

17.12 - Figure S9E - alpha diversity between Root biomass response groups

```
############# Data Filtering and Preparation

# Filter data for Treatment "T" and exclude genotypes 71 and 495
datarITS_root_pheno <- datarITS_root %>%
  filter(Treatment == "T") %>%
  filter(genotype != "71" & genotype != "495") %>%
  droplevels()

# Define interval_ratioroot based on ratioroot values
datarITS_root_pheno$interval_ratioroot <- as.factor(
  ifelse(datarITS_root_pheno$ratioroot < 0.80, "-",
         ifelse(datarITS_root_pheno$ratioroot > 1.20, "+", "/"))
)

# Check if the factor levels are correct
levels(datarITS_root_pheno$interval_ratioroot)
```

```
## [1] "-" "/" "+"
```

```
############# Select Rows for Specific Root Response Groups

# Select rows with interval_ratioroot in ("-", "+")
datarITS_root_response <- datarITS_root_pheno %>%
  filter(interval_ratioroot %in% c("-", "+")) %>%
  droplevels()

############# 1. Shannon Index - Dunn's Test

# Perform Dunn's test for Shannon ASV richness across interval_ratioroot groups
res_obs_shannon <- dunn_test(Shannon ~ interval_ratioroot,
                             data = datarITS_root_response,
                             p.adjust.method = "fdr")

# Extract statistical results for annotation
stat.test_shannon <- res_obs_shannon %>% add_xy_position(x = "interval_ratioroot")

# Create plot for Shannon ASV richness
plot_ITS_Shannon_b <- ggplot(datarITS_root_response, aes(interval_ratioroot, Shanno
n)) +
  geom_boxplot(aes(fill = interval_ratioroot),
               show.legend = TRUE,
               outlier.shape = NA) +  # Hide outliers
  geom_jitter(size = 3) +  # Add jittered points for better visualization
  labs(x = "Root Response Group", y = "Shannon ASV Richness") +  # Axis labels
  theme_bw() +  # Clean theme
  theme(panel.grid.major = element_blank(),
        panel.grid.minor = element_blank()) +  # Remove grid lines
  stat_pvalue_manual(stat.test_shannon, hide.ns = TRUE) +  # Add p-values
  scale_fill_manual(values = c("-" = "#f5ebe0",
                               "+" = "#8b5e34"))  # Custom color palette

# Display the Shannon plot
plot_ITS_Shannon_b
```

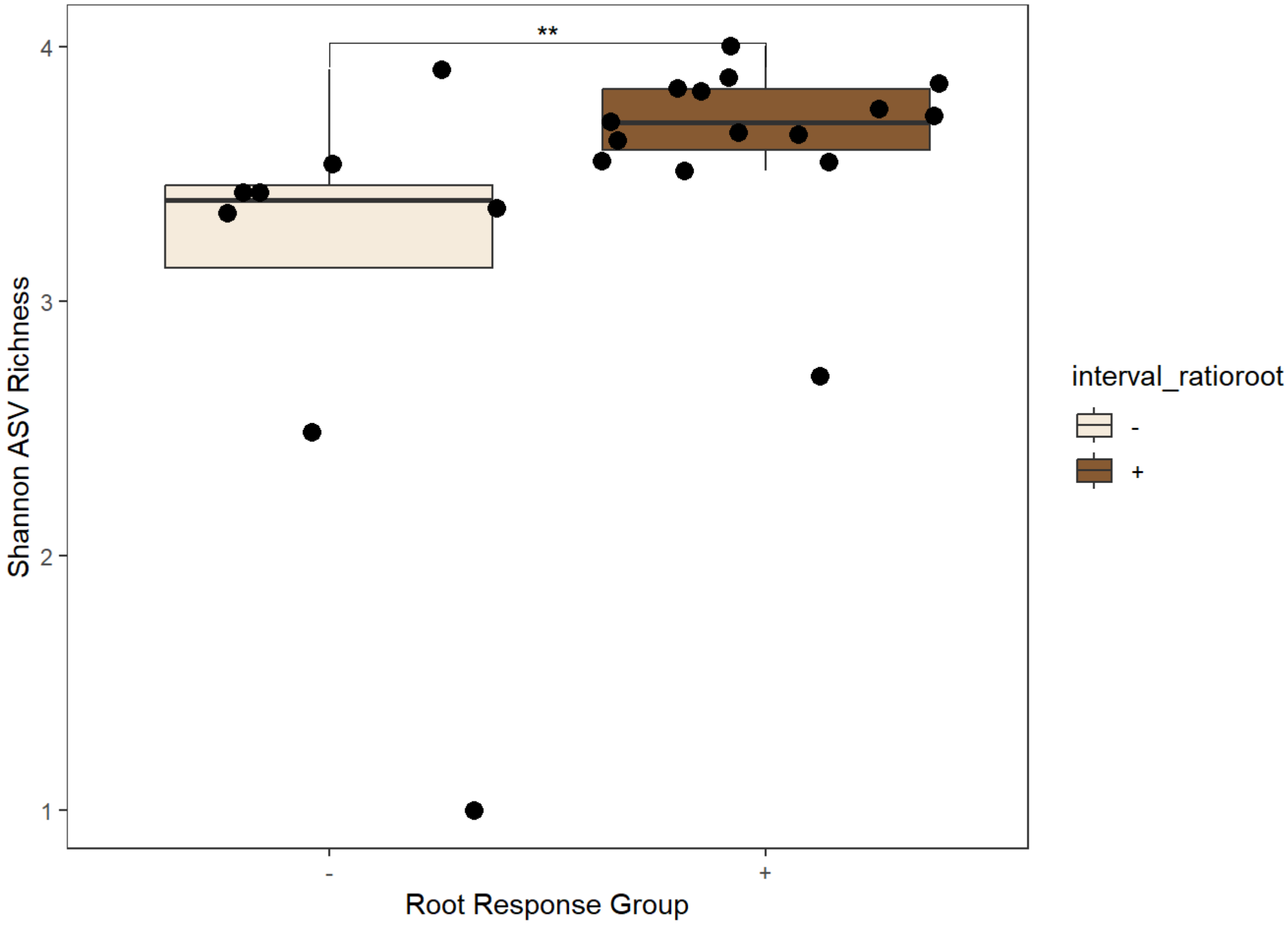


```
############# 2. Observed Index - Dunn's Test

# Perform Dunn's test for Observed ASV richness across interval_ratioroot groups
res_obs_observed <- dunn_test(Observed ~ interval_ratioroot,
                              data = datarITS_root_response,
                              p.adjust.method = "fdr")

# Extract statistical results for annotation
stat.test_observed <- res_obs_observed %>% add_xy_position(x = "interval_ratioroot")

# Create plot for Observed ASV richness
plot_ITS_Observed_b <- ggplot(datarITS_root_response, aes(interval_ratioroot, Observe
d)) +
  geom_boxplot(aes(fill = interval_ratioroot),
               show.legend = TRUE,
               outlier.shape = NA) +  # Hide outliers
  geom_jitter(size = 3) +  # Add jittered points for better visualization
  labs(x = "Root Response Group", y = "Observed ASV Richness") +  # Axis labels
  theme_bw() +  # Clean theme
  theme(panel.grid.major = element_blank(),
        panel.grid.minor = element_blank()) +  # Remove grid lines
  stat_pvalue_manual(stat.test_observed, hide.ns = TRUE) +  # Add p-values
  scale_fill_manual(values = c("-" = "#f5ebe0",
                               "+" = "#8b5e34"))  # Custom color palette

# Display the Observed plot
plot_ITS_Observed_b
```

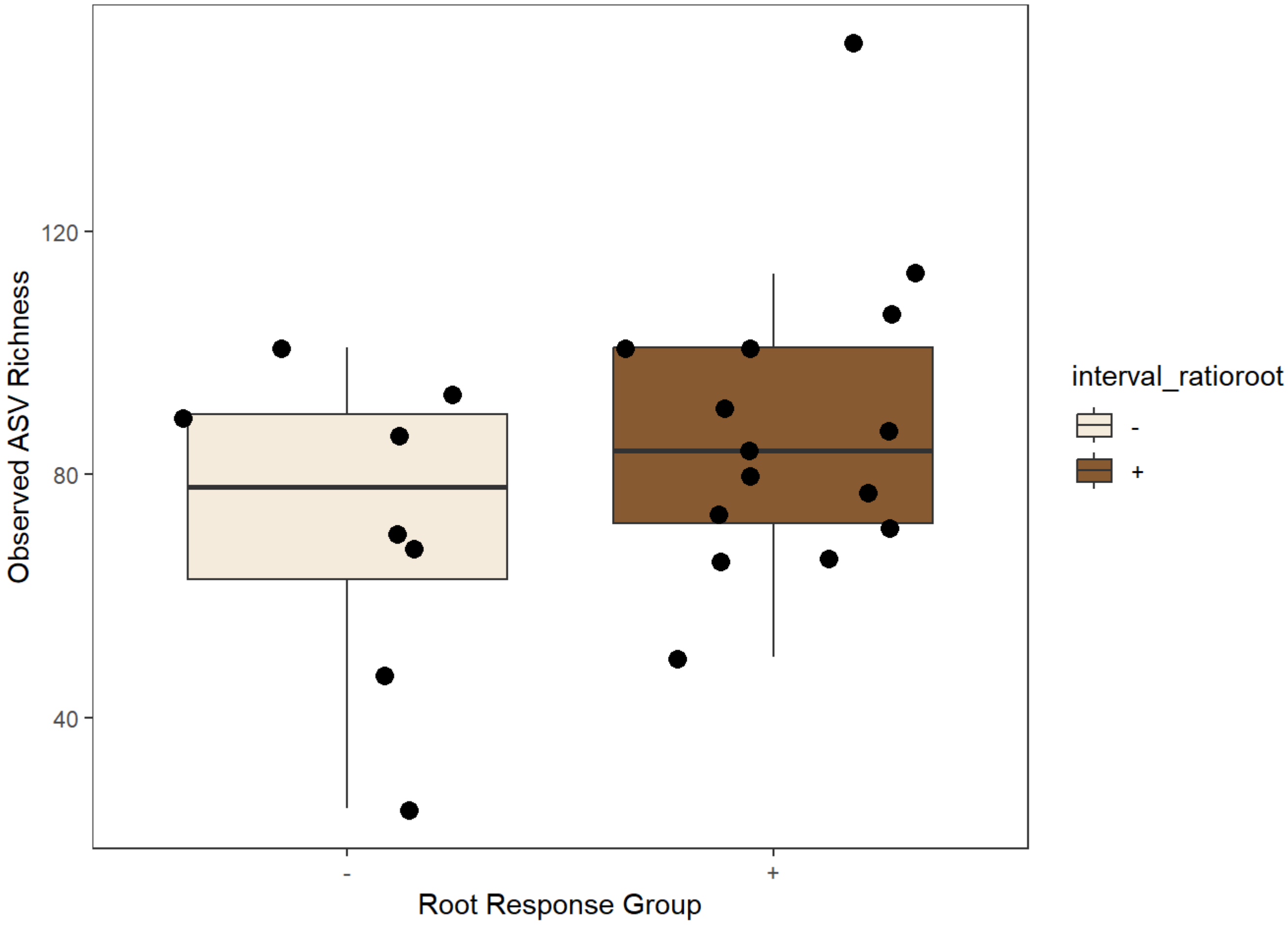

17.13 - Figure S9F - alpha diversity between Shoot biomass/root biomass response groups

```
# Data Transformation: Ensure the variables are numeric
datarITS_root_pheno <- datarITS_root %>%
  filter(Treatment == "T") %>%
  filter(genotype != "71" & genotype != "495") %>%
  droplevels()

# Transforming variables into numeric
datarITS_root_pheno$ratioS.R <- as.numeric(datarITS_root_pheno$ratioS.R)

# Create interval for 'ratioS.R'
datarITS_root_pheno$interval_ratioS.R <- as.factor(ifelse(datarITS_root_pheno$ratioS.
R < 0.80, "-",
                                                                  ifelse(datarITS_root_pheno
$ratioS.R > 1.20, "+", "/")))

# Select rows where 'interval_ratioS.R' is in ("-", "+")
datarITS_S.R_response <- datarITS_root_pheno %>%
  filter(interval_ratioS.R %in% c("-", "+"))

############# 1. Shannon Index - Dunn's Test

# Dunn's test for Shannon ASV richness
res_obs_shannon <- dunn_test(Shannon ~ interval_ratioS.R,
                             data = datarITS_S.R_response,
                             p.adjust.method = "fdr")

# Extract statistical results for annotation
stat.test_shannon <- res_obs_shannon %>% add_xy_position(x = "interval_ratioS.R")

# Create Shannon ASV richness plot
plot_ITS_Shannon_b <- ggplot(datarITS_S.R_response, aes(interval_ratioS.R, Shannon))
+
  geom_boxplot(aes(fill = interval_ratioS.R),
               show.legend = TRUE,
               outlier.shape = NA) +  # Hide outliers
  geom_jitter(size = 3) +  # Add jittered points
  labs(x = "Root Response Group", y = "Shannon ASV Richness") +  # Axis labels
  theme_bw() +  # Clean theme
  theme(panel.grid.major = element_blank(),
        panel.grid.minor = element_blank()) +  # Remove grid lines
  stat_pvalue_manual(stat.test_shannon, hide.ns = TRUE) +  # Add p-values
  scale_fill_manual(values = c("-" = "#e6ece5",
                               "+" = "#556b2f",
                               "/" = "#9caf91"))  # Custom color palette

# Display the Shannon plot
plot_ITS_Shannon_b
```

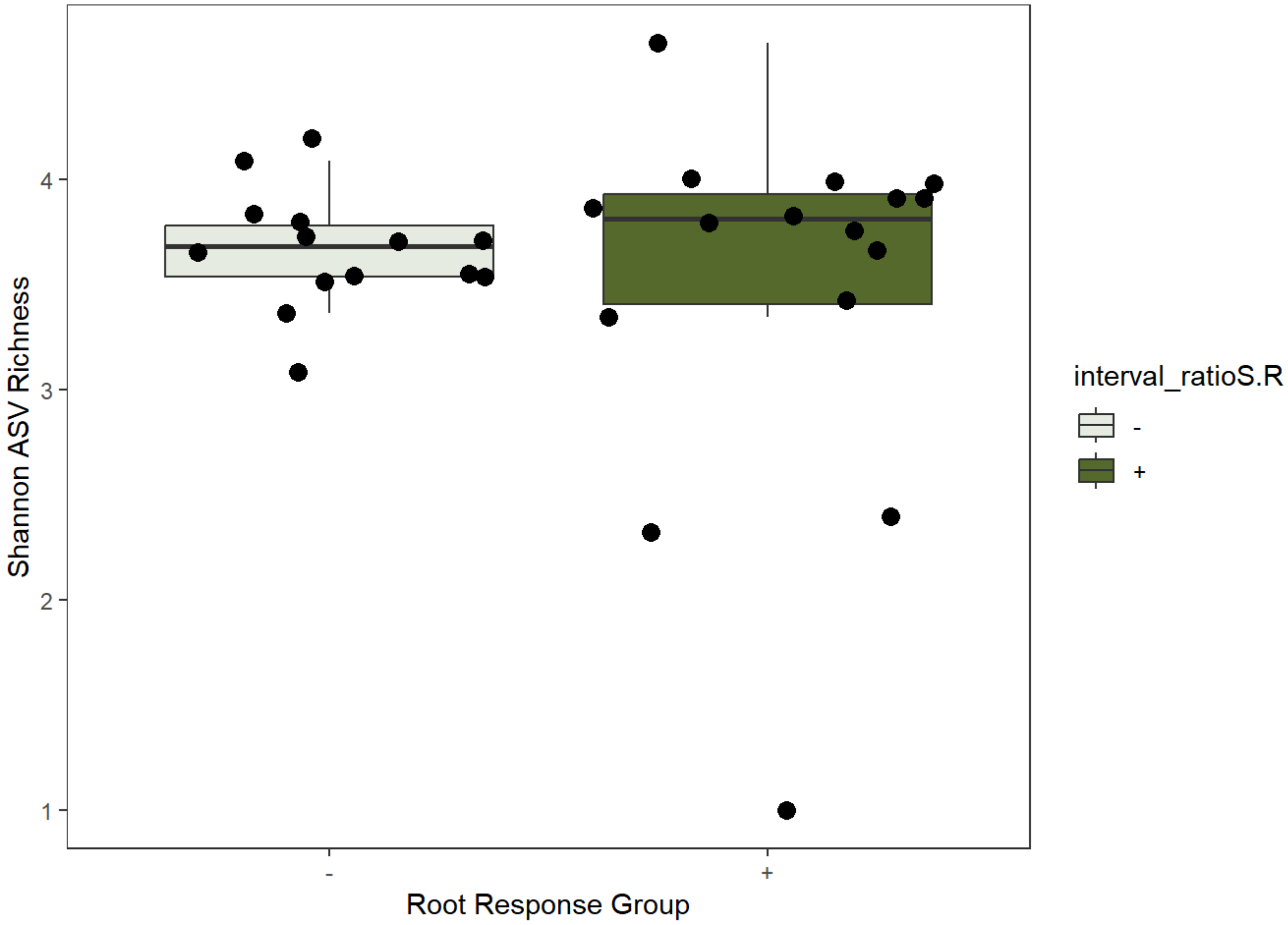


```
############# 2. Observed Index - Dunn's Test

# Dunn's test for Observed ASV richness
res_obs_observed <- dunn_test(Observed ~ interval_ratioS.R,
                              data = datarITS_S.R_response,
                              p.adjust.method = "fdr")

# Extract statistical results for annotation
stat.test_observed <- res_obs_observed %>% add_xy_position(x = "interval_ratioS.R")

# Create Observed ASV richness plot
plot_ITS_Observed_b <- ggplot(datarITS_S.R_response, aes(interval_ratioS.R, Observe
d)) +
  geom_boxplot(aes(fill = interval_ratioS.R),
               show.legend = TRUE,
               outlier.shape = NA) +  # Hide outliers
  geom_jitter(size = 3) +  # Add jittered points
  labs(x = "Root Response Group", y = "Observed ASV Richness") +  # Axis labels
  theme_bw() +  # Clean theme
  theme(panel.grid.major = element_blank(),
        panel.grid.minor = element_blank()) +  # Remove grid lines
  stat_pvalue_manual(stat.test_observed, hide.ns = TRUE) +  # Add p-values
  scale_fill_manual(values = c("-" = "#e6ece5",
                               "+" = "#556b2f",
                               "/" = "#9caf91"))  # Custom color palette

# Display the Observed plot
plot_ITS_Observed_b
```

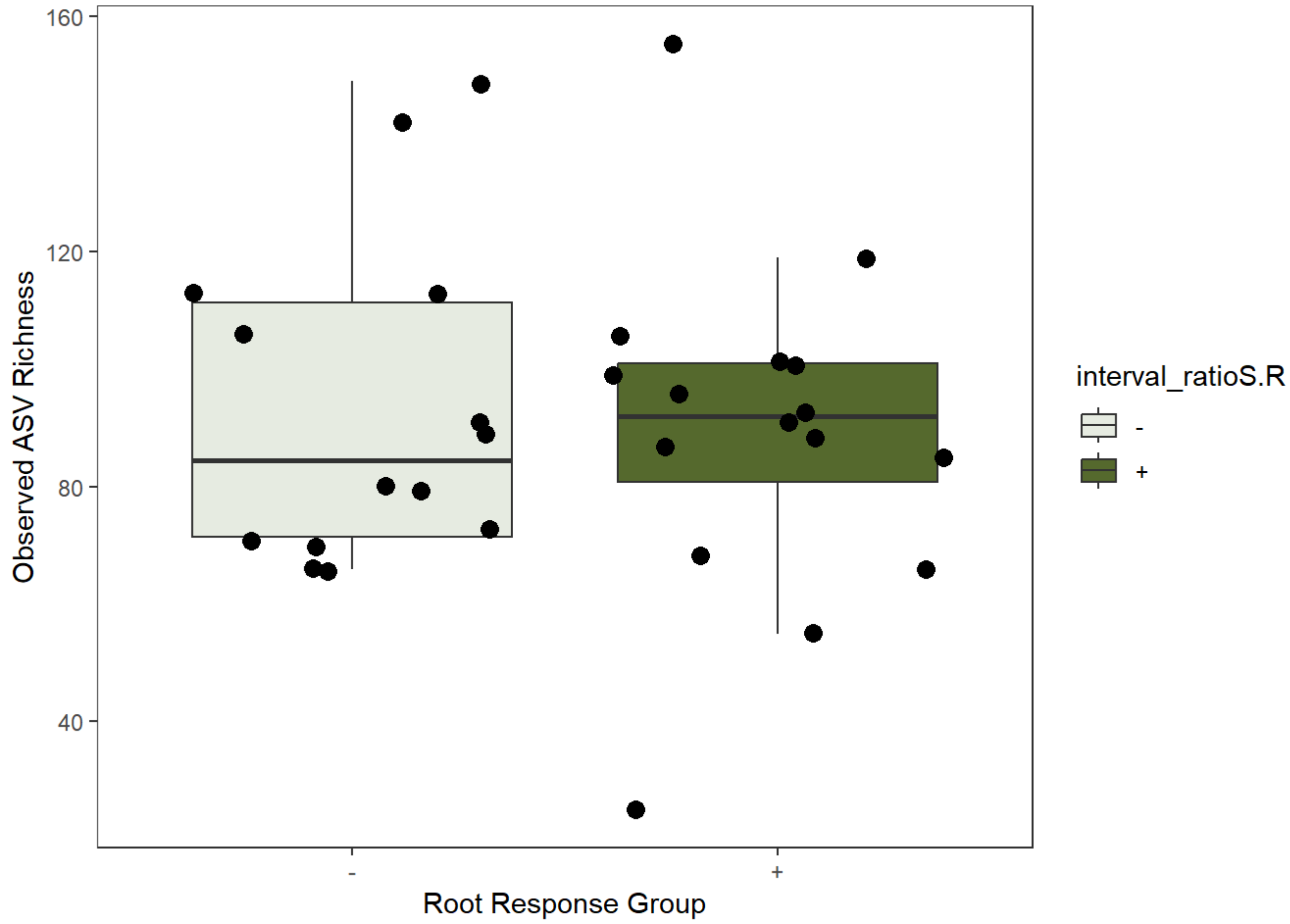

17.14 - Figure 5A - Beta diversity across interval response and phenotypic groups

```
# Subset the data to include only 'root' tissue samples
merged_phylo_root <- subset_samples(merged_phylo, tissue %in% c("root"))

# 1. PERMANOVA FOR PHENOTYPIC GROUPS AND INTERVAL RESPONSE GROUPS
# Subset samples based on specific interval_response groups
merged_phylo_root1 <- subset_samples(merged_phylo_root, interval_response == "--" | i
nterval_response == "++" | interval_response == "-+")

# Further filter to remove 'CTRL' treatment group
merged_phylo_root1 <- subset_samples(merged_phylo_root1, Treatment != "CTRL")

# Convert 'interval_response' and 'phenotypic_group' to factors
sample_data(merged_phylo_root1)$interval_response <- as.factor(sample_data(merged_phy
lo_root1)$interval_response)
sample_data(merged_phylo_root1)$phenotypic_group <- as.factor(sample_data(merged_phyl
o_root1)$phenotypic_group)

# Check levels of 'interval_response' and 'phenotypic_group'
levels(sample_data(merged_phylo_root1)$interval_response)
```

```
## [1] "--" "-+" "++"
```

```
levels(sample_data(merged_phylo_root1)$phenotypic_group)
```

```
## [1] "1" "2" "3" "4"
```

```
# Calculate Bray-Curtis dissimilarity matrix
bray.ITS <- phyloseq::distance(merged_phylo_root1, method = "bray")

# Convert sample data into a data frame for the PERMANOVA analysis
mdat.ITSb <- as(sample_data(merged_phylo_root1), "data.frame")

# Perform PERMANOVA (adonis2) with interaction between 'interval_response' and 'pheno
typic_group'
adonis2(bray.ITS ~phenotypic_group,
        data = mdat.ITSb,
        permutations = 20000)
```

	Df <dbl>	SumOfSqs <dbl>	R2 <dbl>	F <dbl>	Pr(>F) <dbl>
Model	3	1.529085	0.4168828	2.144764	0.01584921
Residual	9	2.138816	0.5831172	NA	NA
Total	12	3.667900	1.0000000	NA	NA

3 rows

```
# 2. PLOT

# Load additional libraries for plotting
library(ggrepel)
library(ggforce)

# Perform Principal Coordinate Analysis (PCoA) based on Bray-Curtis dissimilarity
bray.ITS <- ordinate(merged_phylo_root1, "PCoA", "bray")

# Define a custom color palette for the 'phenotypic_group' groups
phenotypic_group <- c(
  "1" = "#00BFFF",   # Group 1
  "2" = "#00FF00",   # Group 2
  "3" = "#FF1493",   # Group 3
  "4" = "#9400D3"    # Group 4
)

# Create the PCoA plot with customized aesthetics
i.bray.pcoa.ITS <- plot_ordination(merged_phylo_root1,
                                   bray.ITS,
                                   type = "samples",
                                   ) +
  geom_jitter(aes(color = phenotypic_group),  # Color and shape for points
              size = 10, width = 0.1, height = 0.1) +
  scale_color_manual(values = phenotypic_group) +  # Apply custom color palette
  theme_bw() +  # Use black-and-white theme
  theme(panel.grid.major = element_blank(),  # Remove major grid lines
        panel.grid.minor = element_blank()  # Remove minor grid lines
  ) +
  ggtitle("ITS rRNA - Bray-Curtis PCoA")  # Add title to the plot

# Display the final PCoA plot
i.bray.pcoa.ITS
```

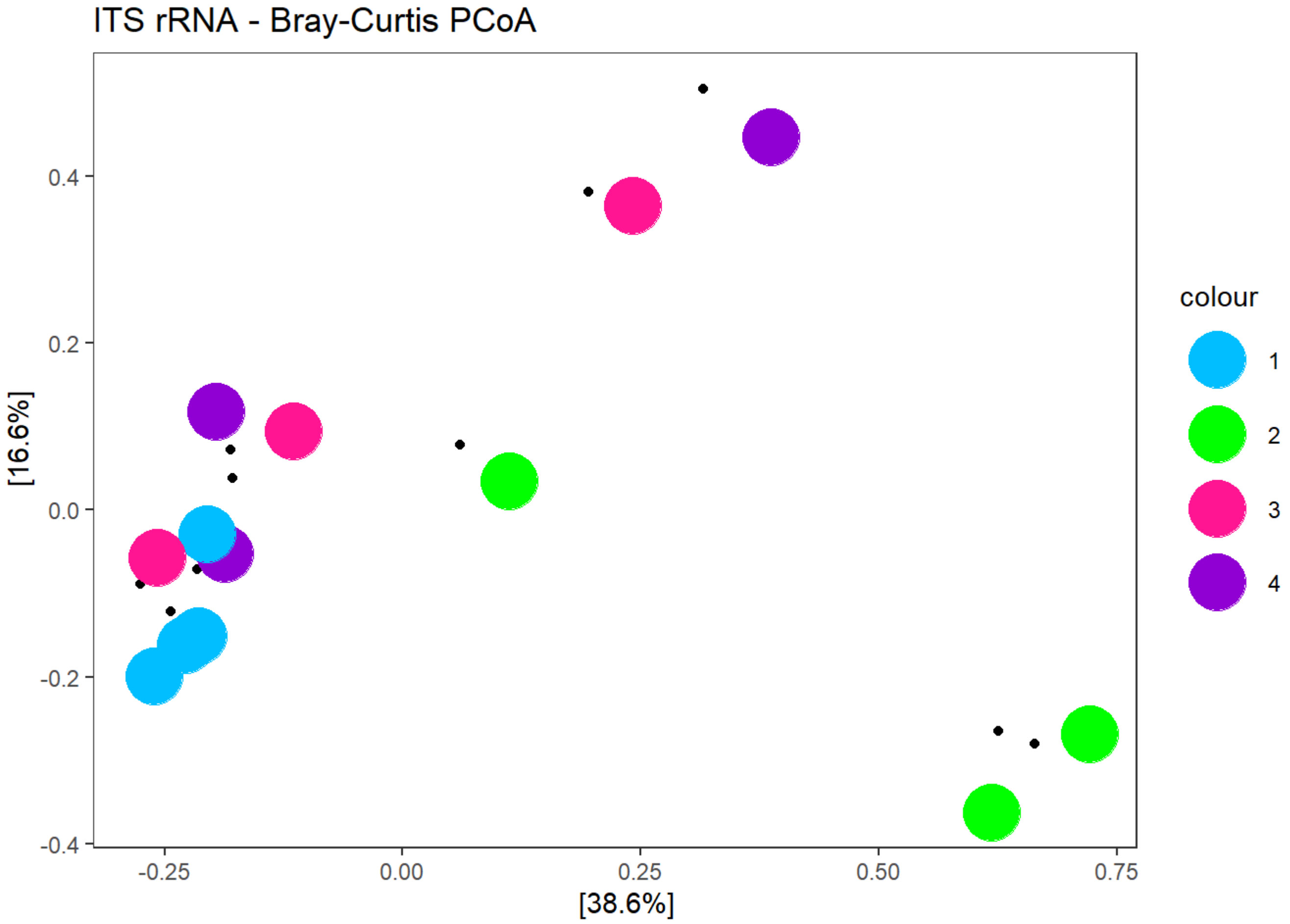

17.15 - Figure S9C - Beta diversity across Pi response groups

```
# 1. ANOVA

# Subset the samples based on the 'Pi.response' variable being either "-" or "+"
merged_phylo_root2 <- subset_samples(merged_phylo_root, Pi.response == "-" | Pi.respo
nse == "+")

# Remove taxa with zero abundance
merged_phylo_root2 <- subset_taxa(merged_phylo_root2, !taxa_sums(merged_phylo_root2)
== 0)

# Convert 'Pi.response' and 'variety' to factors
sample_data(merged_phylo_root2)$Pi.response <- as.factor(sample_data(merged_phylo_roo
t2)$Pi.response)
sample_data(merged_phylo_root2)$variety <- as.factor(sample_data(merged_phylo_root2)
$variety)

# Calculate Bray-Curtis dissimilarity
bray.ITS <- phyloseq::distance(merged_phylo_root2, method = "bray")

# Extract metadata for use in the ANOVA test
mdat.ITSb <- as(sample_data(merged_phylo_root2), "data.frame")

# Perform the PERMANOVA (adonis2) test using Bray-Curtis dissimilarity
adonis.ITSbray <- adonis2(bray.ITS ~ Pi.response,
                          data = mdat.ITSb, perm = 20000)

# Print the results of the PERMANOVA test
print(adonis.ITSbray)
```

```
## Permutation test for adonis under reduced model
## Permutation: free
## Number of permutations: 20000
##
## adonis2(formula = bray.ITS ~ Pi.response, data = mdat.ITSb, permutations = 20000)
##          Df SumOfSqs      R2      F Pr(>F)
## Model     1   0.3448 0.05857 1.0576 0.3421
## Residual 17   5.5427 0.94143
## Total    18   5.8876 1.00000
```

```
# 2. Plot

# Load the ggrepel package for improved labeling in the plot
library(ggrepel)

# Perform Principal Coordinate Analysis (PCoA) based on Bray-Curtis dissimilarity
bray.pcoa.ITS_s <- ordinate(merged_phylo_root2, "PCoA", "bray")

# Define a custom color palette for the 'Pi.response' groups
palette_custom <- c("-" = "#ece7f2", "+" = "#2b8cbe")

# Create the PCoA plot with customized aesthetics
i.bray.pcoa.ITS <- plot_ordination(merged_phylo_root2,
                                   bray.pcoa.ITS_s,
                                   type = "samples",
                                   color = "Pi.response") +
  stat_ellipse(aes(group = Pi.response), color = "black") +  # Add ellipses for each
group
  geom_point(size = 5, shape = 19, stroke = 0.5, aes(color = Pi.response), fill = NA,
color = "black") +  # Points with black border
  geom_point(size = 4.5, aes(color = Pi.response)) +  # Slightly smaller colored poin
ts
  ggtitle("ITS rRNA - Bray-Curtis PCoA") +  # Add a title to the plot
  scale_color_manual(values = palette_custom) +  # Apply custom color palette
  theme_bw() +  # Use a black-and-white theme
  theme(panel.grid.major = element_blank(),  # Remove major grid lines
        panel.grid.minor = element_blank(),  # Remove minor grid lines
        panel.background = element_blank())  # Remove background color

# Display the final PCoA plot
i.bray.pcoa.ITS
```

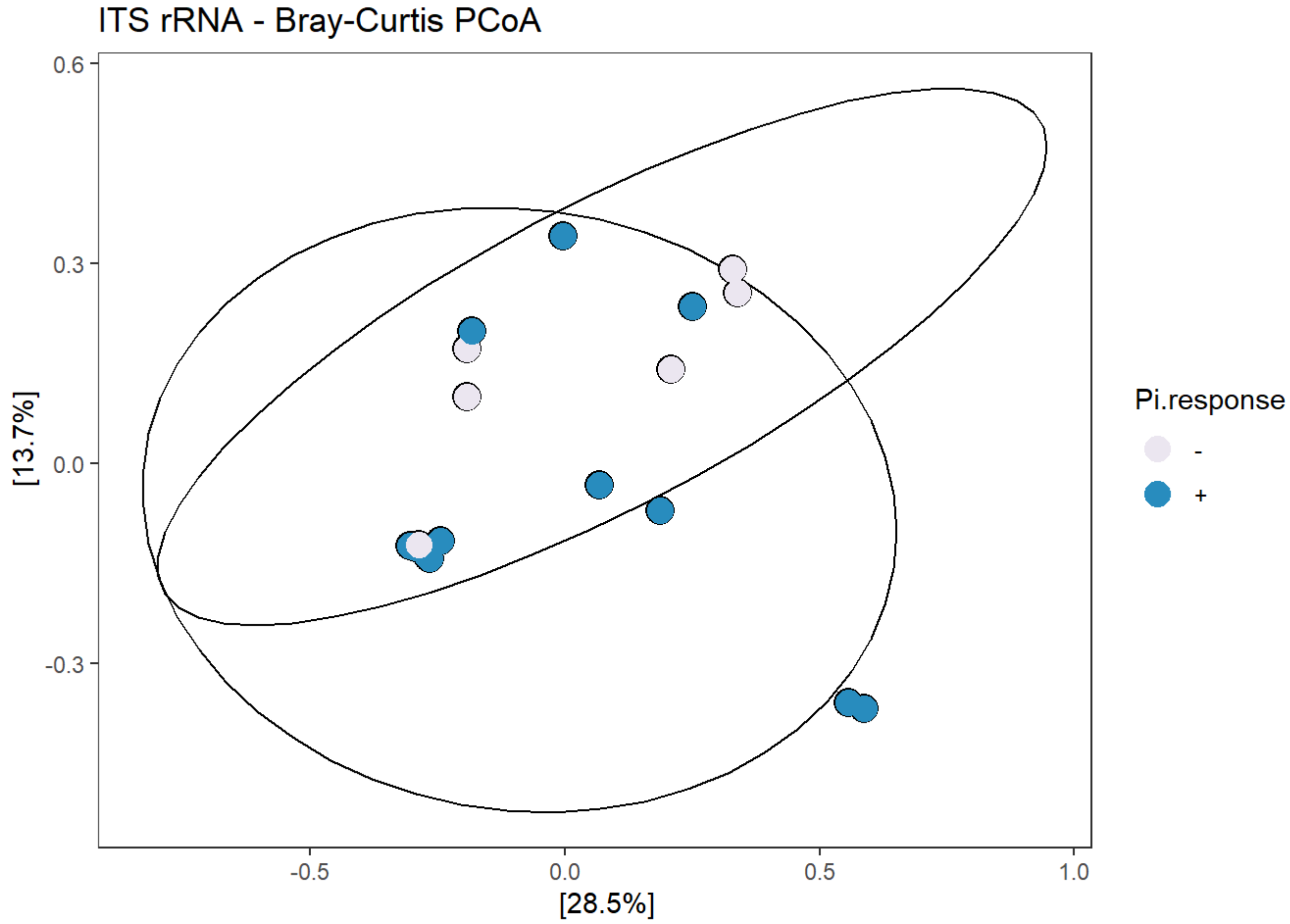

17.16 - Figure S9D - Beta diversity across Shoot response groups

```
# 1. PERMANOVA

# Subset the samples based on the 'Shoot.response' variable being either "-" or "+"
merged_phylo_root3 <- subset_samples(merged_phylo_root, Shoot.response == "-" | Shoo
t.response == "+")

# Convert 'Shoot.response' to a factor
sample_data(merged_phylo_root3)$Shoot.response <- as.factor(sample_data(merged_phylo_
root3)$Shoot.response)

# Calculate Bray-Curtis dissimilarity
bray.ITS <- phyloseq::distance(merged_phylo_root3, method = "bray")

# Extract metadata for use in the PERMANOVA test
mdat.ITSb <- as(sample_data(merged_phylo_root3), "data.frame")

# Check the levels of 'Shoot.response'
levels(sample_data(merged_phylo_root3)$Shoot.response)
```

```
## [1] "-" "+"
```

```
# Perform the PERMANOVA (adonis2) test using Bray-Curtis dissimilarity
adonis.ITSbray <- adonis2(bray.ITS ~ Shoot.response,
                          data = mdat.ITSb, perm = 10000)

# Print the results of the PERMANOVA test
print(adonis.ITSbray)
```

```
## Permutation test for adonis under reduced model
## Permutation: free
## Number of permutations: 10000
##
## adonis2(formula = bray.ITS ~ Shoot.response, data = mdat.ITSb, permutations = 1000
0)
##          Df SumOfSqs      R2      F Pr(>F)
## Model     1   0.4381 0.05077 1.2835 0.1665
## Residual 24   8.1924 0.94923
## Total    25   8.6305 1.00000
```

```
# 2. Plot

# Load the ggrepel package for improved labeling in the plot
library(ggrepel)

# Perform Principal Coordinate Analysis (PCoA) based on Bray-Curtis dissimilarity
bray.pcoa.ITS_s <- ordinate(merged_phylo_root3, "PCoA", "bray")

# Define a custom color palette for the 'Shoot.response' groups
palette_custom <- c("-" = "#e5f5f9", "+" = "#2ca25f")

# Create the PCoA plot with customized aesthetics
i.bray.pcoa.ITS <- plot_ordination(merged_phylo_root3,
                                   bray.pcoa.ITS_s,
                                   type = "samples",
                                   color = "Shoot.response") +
  stat_ellipse(aes(group = Shoot.response), color = "black") +  # Add ellipses for ea
ch group
  geom_point(size = 5, shape = 19, stroke = 0.5, aes(color = Shoot.response), fill =
NA, color = "black") +  # Points with black border
  geom_point(size = 4.5, aes(color = Shoot.response)) +  # Slightly smaller colored p
oints
  ggtitle("ITS rRNA - Bray-Curtis PCoA") +  # Add a title to the plot
  scale_color_manual(values = palette_custom) +  # Apply custom color palette
  theme_bw() +  # Use a black-and-white theme
  theme(panel.grid.major = element_blank(),  # Remove major grid lines
        panel.grid.minor = element_blank(),  # Remove minor grid lines
        panel.background = element_blank())  # Remove background color

# Display the final PCoA plot
i.bray.pcoa.ITS
```

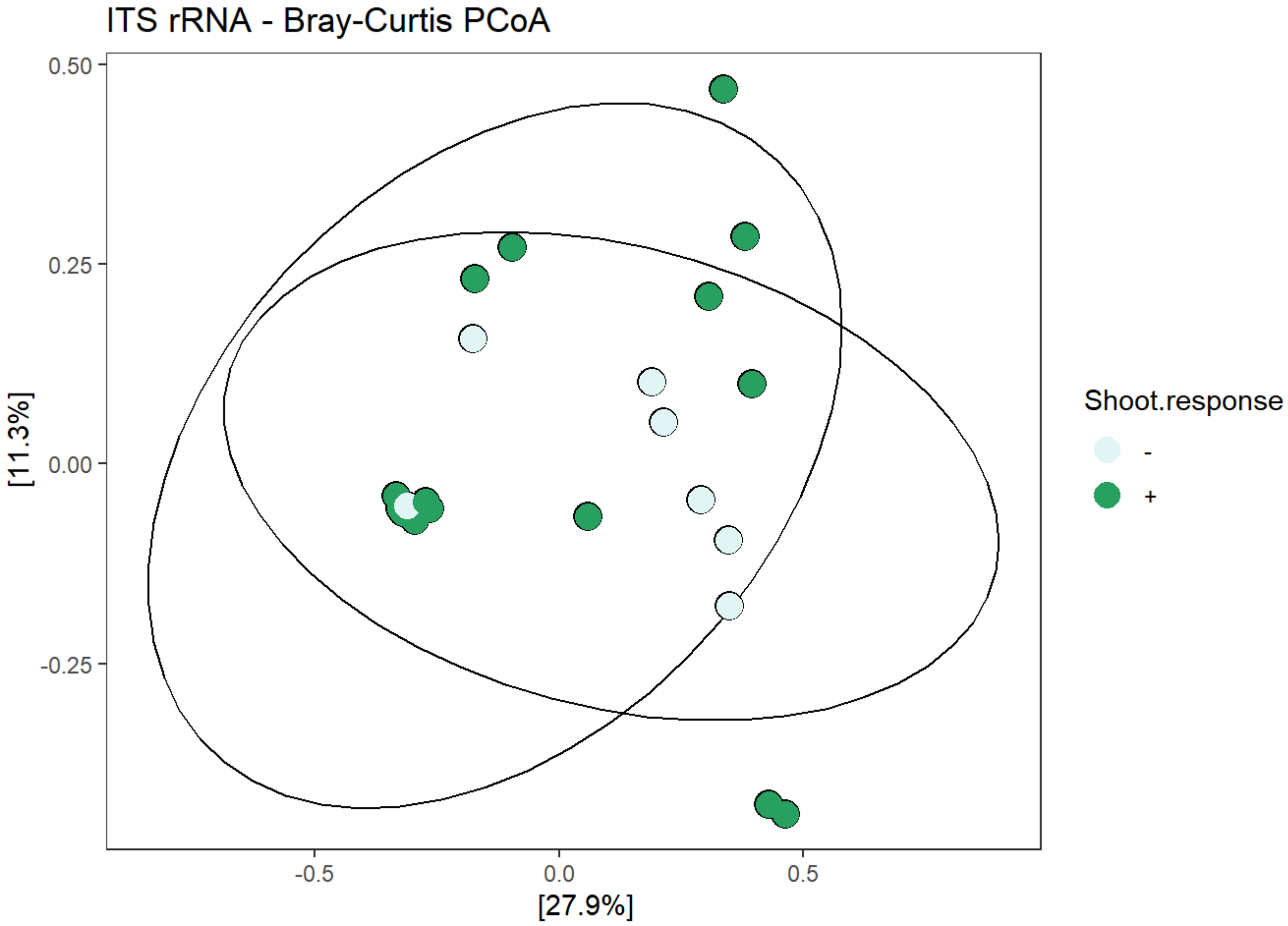

17.17 - Figure S9E - Beta diversity across root response groups

```
# 1. PERMANOVA

merged_phylo_root4 <- subset_samples(merged_phylo_root, tissue %in% c("root") & !geno
type %in% c("71", "495"))
merged_phylo_root4 <- subset_taxa(merged_phylo_root4, !taxa_sums(merged_phylo_root4)
== 0)

# Add interval for root
sample_data(merged_phylo_root4)$interval_ratioroot <- as.factor(ifelse(sample_data(me
rged_phylo_root4)$ratioroot < 0.80, "-",
                                                                  ifelse(sample_data(merged_
phylo_root4)$ratioroot > 1.20, "+", "/")))

rITS_root5 <- subset_samples(merged_phylo_root4, interval_ratioroot == "-" | interval
_ratioroot == "+")

# Calculate Bray-Curtis dissimilarity
bray.ITS <- phyloseq::distance(rITS_root5, method = "bray")
mdat.ITSb <- as(sample_data(rITS_root5), "data.frame")

mdat.ITSb$ratioroot <- as.numeric(mdat.ITSb$ratioroot)

# Perform PERMANOVA (adonis2) test
adonis.ITSbray <- adonis2(bray.ITS ~ ratioroot,
                          data = mdat.ITSb, perm = 999)

# Print PERMANOVA results
print(adonis.ITSbray)
```

```
## Permutation test for adonis under reduced model
## Permutation: free
## Number of permutations: 999
##
## adonis2(formula = bray.ITS ~ ratioroot, data = mdat.ITSb, permutations = 999)
##          Df SumOfSqs      R2      F Pr(>F)
## Model     1   0.3500 0.04722 1.0408  0.361
## Residual 21   7.0622 0.95278
## Total    22   7.4122 1.00000
```

```
# 2. Plot

library(ggrepel)

# Perform Principal Coordinate Analysis (PCoA) using Bray-Curtis dissimilarity
bray.pcoa.ITS_s <- ordinate(rITS_root5, "PCoA", "bray")

# Define a custom color palette for the 'interval_ratioroot' groups
palette_custom <- c("-" = "#f5ebe0", "+" = "#8b5e34")

# Create the PCoA plot with customized aesthetics
i.bray.pcoa.ITS <- plot_ordination(rITS_root5,
                                   bray.pcoa.ITS_s,
                                   type = "samples",
                                   color = "interval_ratioroot") +
  stat_ellipse(aes(group = interval_ratioroot), color = "black") +  # Add ellipses fo
r each group
  geom_point(size = 5, shape = 19, stroke = 0.5, aes(color = interval_ratioroot), fil
l = NA, color = "black") +  # Points with black border
  geom_point(size = 4.5, aes(color = interval_ratioroot)) +  # Slightly smaller color
ed points
  ggtitle("ITS rRNA - Bray-Curtis PCoA") +  # Add a title to the plot
  scale_color_manual(values = palette_custom) +  # Apply custom color palette
  theme_bw() +  # Use a black-and-white theme
  theme(panel.grid.major = element_blank(),  # Remove major grid lines
        panel.grid.minor = element_blank(),  # Remove minor grid lines
        panel.background = element_blank())  # Remove background color

# Display the final PCoA plot
i.bray.pcoa.ITS
```

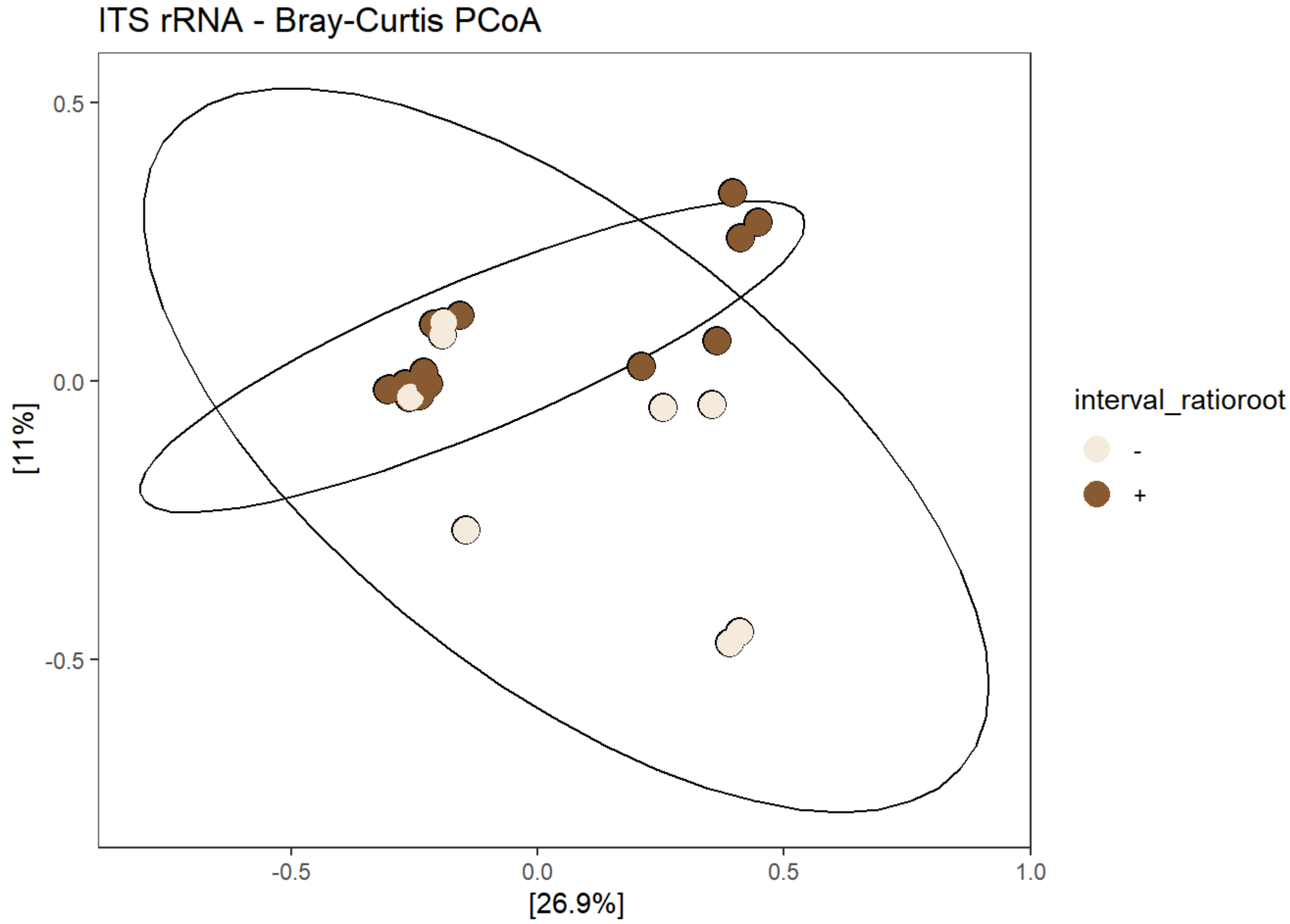

17.18 - Fugure S9F - Beta diversity across Shoot/root response groups

```
#1. PERMANOVA

## Subsetting samples to include only 'root' tissue and exclude specific genotypes (7
1 and 495)
merged_phylo_root5 <- subset_samples(merged_phylo_root, tissue %in% c("root") & !geno
type %in% c("71", "495"))

# Removing taxa with zero sums (i.e., taxa not present in any samples)
merged_phylo_root5 <- subset_taxa(merged_phylo_root5, !taxa_sums(merged_phylo_root5)
== 0)

# Creating an interval variable based on the ratioS.R value
# Assigning "-" if ratioS.R < 0.80, "+" if ratioS.R > 1.20, and "/" for values in bet
ween
sample_data(merged_phylo_root5)$interval_S.R_ratio <- as.factor(ifelse(sample_data(me
rged_phylo_root5)$ratioS.R < 0.80, "-",
                                                           ifelse(sample_data(merged_
phylo_root5)$ratioS.R > 1.20, "+", "/")))

# Subsetting the samples where the interval classification is either "-" or "+"
rITS_root6 <- subset_samples(merged_phylo_root5, interval_S.R_ratio == "-" | interval
_S.R_ratio == "+")

# Calculating the Bray-Curtis dissimilarity matrix for the subsetted samples
bray.ITS <- phyloseq::distance(rITS_root6, method = "bray")

# Converting sample data to a data frame and ensuring ratioS.R is numeric for the ana
lysis
mdat.ITSb <- as(sample_data(rITS_root6), "data.frame")
mdat.ITSb$ratioS.R <- as.numeric(mdat.ITSb$ratioS.R)

# Running the PERMANOVA test (adonis2) with Bray-Curtis dissimilarity as the dependen
t variable and ratioS.R as the independent variable
# Using 999 permutations to test for significance
adonis.ITSbray <- adonis2(bray.ITS ~ ratioS.R,
                          data = mdat.ITSb, perm = 999)

# Printing the results of the PERMANOVA test
print(adonis.ITSbray)
```

```
## Permutation test for adonis under reduced model
## Permutation: free
## Number of permutations: 999
##
## adonis2(formula = bray.ITS ~ ratioS.R, data = mdat.ITSb, permutations = 999)
##          Df SumOfSqs      R2      F Pr(>F)
## Model     1   0.2405 0.02925 0.8437   0.55
## Residual 28   7.9798 0.97075
## Total    29   8.2202 1.00000
```

```
#2. PLOT

# Loading the 'ggrepel' package for improved plotting with text labels (if needed)
library(ggrepel)

# Performing Principal Coordinate Analysis (PCoA) based on Bray-Curtis dissimilarity
bray.pcoa.ITS_s <- ordinate(rITS_root6, "PCoA", "bray")

# Defining a custom color palette for the three levels of 'interval_S.R_ratio'
# "-" will be represented by a light green color, "+" by a dark olive green
palette_custom <- c("-" = "#e6ece5", "+" = "#556b2f")

# Creating the PCoA plot with customized aesthetics
i.bray.pcoa.ITS <- plot_ordination(rITS_root6,
                                   bray.pcoa.ITS_s,
                                   type = "samples",  # Indicating that we're plottin
g sample data
                                   color = "interval_S.R_ratio") +  # Coloring the po
ints by the 'interval_S.R_ratio' factor
  stat_ellipse(aes(group = interval_S.R_ratio), color = "black") +  # Adding black el
lipses to indicate groupings
  geom_point(size = 5, shape = 19, stroke = 0.5, aes(color = interval_S.R_ratio), fil
l = NA, color = "black") +  # Adding large points with a black border
  geom_point(size = 4.5, aes(color = interval_S.R_ratio)) +  # Adding smaller colored
points for each sample
  ggtitle("ITS rRNA - Bray-Curtis PCoA") +  # Adding a title to the plot
  scale_color_manual(values = palette_custom) +  # Applying the custom color palette
to the plot
  theme_bw() +  # Using a black-and-white theme for the plot
  theme(panel.grid.major = element_blank(),  # Removing major grid lines for a cleane
r look
        panel.grid.minor = element_blank(),  # Removing minor grid lines
        panel.background = element_blank())  # Removing the background color

# Displaying the final PCoA plot
i.bray.pcoa.ITS
```

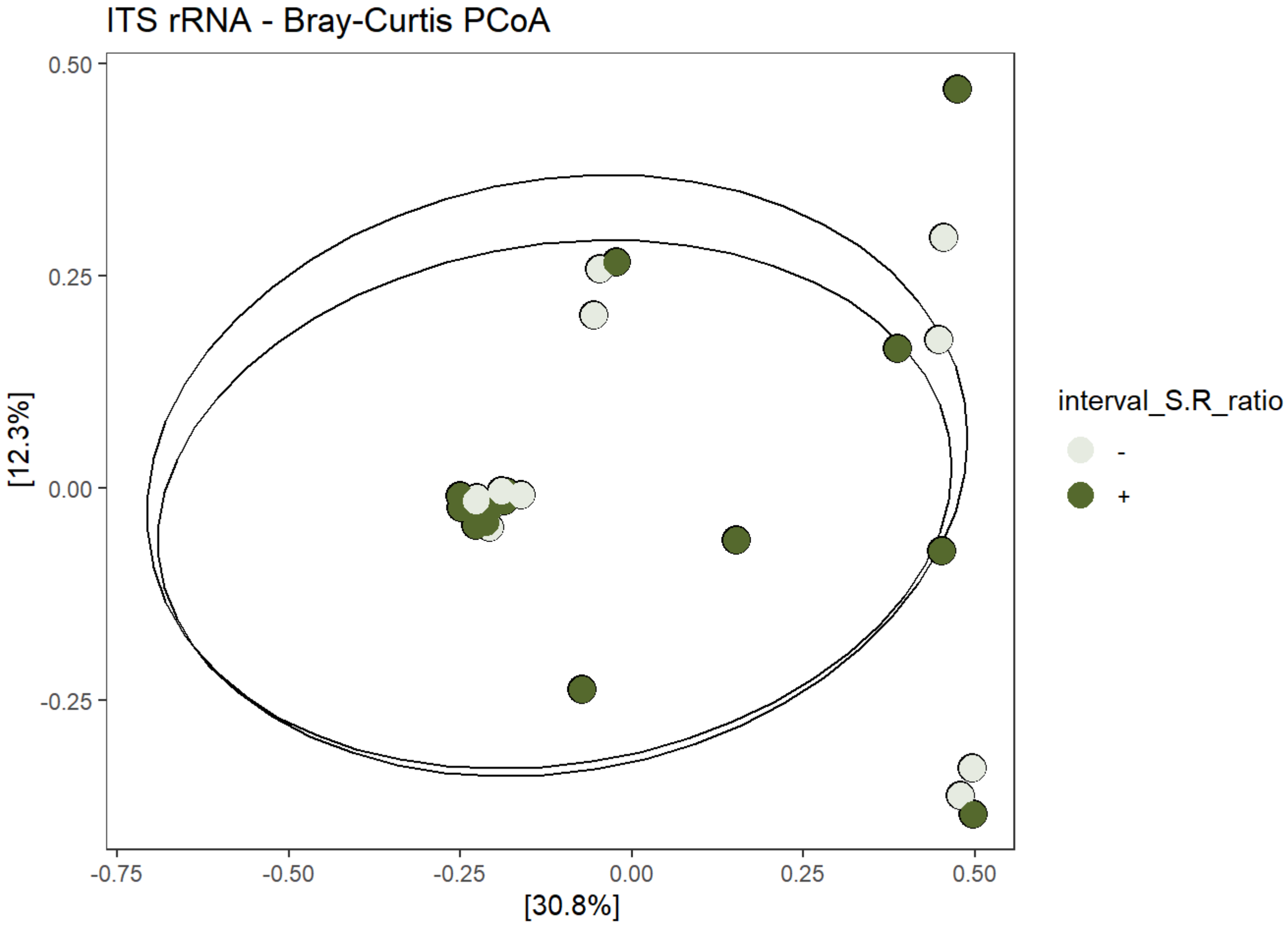

18 - Fungal and bacterial analysis

18.1 - Import fungal and bacterial phyloseq objects

```
# Load necessary libraries
library(phyloseq)
library(ggplot2)
library(readxl)
library(dplyr)
library(tibble)
library(devtools)
library(ranacapa)
library(tidyverse)
library(rstatix)
library(ggthemes)
library(multcompView)
library(agricolae)

# -----------------------------
# Fungal Data Preparation
# -----------------------------

# Read in the fungal dataset
d16S_fungus <- readRDS("ps16S_clean_ITS.rds")

# Subset for 'root' tissue type
d16S_fungus_roots <- subset_samples(d16S_fungus, tissue %in% c("root"))

# Set depth based on the smallest sample size across all samples
depth <- min(colSums(otu_table(d16S_fungus_roots)))

# Rarefy to an even depth of 200 sequences per sample
d16S_fungus_roots <- rarefy_even_depth(d16S_fungus_roots, sample.size = 1000, rngseed
= 900)
```

```
## `set.seed(900)` was used to initialize repeatable random subsampling.
```

```
## Please record this for your records so others can reproduce.
```

```
## Try `set.seed(900); .Random.seed` for the full vector
```

```
## ...
```

```
## 36 samples removedbecause they contained fewer reads than `sample.size`.
```

```
## Up to first five removed samples are:
```

```
## A1A17A19A20A31
```

```
## ...
```

```
## 3581OTUs were removed because they are no longer
## present in any sample after random subsampling
```

```
## ...
```

```
# Remove taxa that sum to zero across all samples (not detected)
d16S_fungus_roots <- subset_taxa(d16S_fungus_roots, !taxa_sums(d16S_fungus_roots) ==
0)

# Create an interval factor based on 'ratioroot'
sample_data(d16S_fungus_roots)$interval_ratioroot <- as.factor(
  ifelse(sample_data(d16S_fungus_roots)$ratioroot < 0.80, "-",
         ifelse(sample_data(d16S_fungus_roots)$ratioroot > 1.20, "+", "/"))
)

# -----------------------------
# Bacterial Data Preparation
# -----------------------------

# Read in the bacterial dataset
d16S_bacteria <- readRDS("ps16S_clean.rds")

# Subset for 'root' tissue type
d16S_bacteria_roots <- subset_samples(d16S_bacteria, tissue %in% c("root"))

# Set depth based on the smallest sample size across all samples
depth <- min(colSums(otu_table(d16S_bacteria_roots)))

# Rarefy to an even depth of 200 sequences per sample
d16S_bacteria_roots <- rarefy_even_depth(d16S_bacteria_roots, sample.size = 1000, rng
seed = 900)
```

```
## `set.seed(900)` was used to initialize repeatable random subsampling.
```

```
## Please record this for your records so others can reproduce.
```

```
## Try `set.seed(900); .Random.seed` for the full vector
```

```
## ...
```

```
## 19422OTUs were removed because they are no longer
## present in any sample after random subsampling
```

```
## ...
```

```
# Remove taxa that sum to zero across all samples (not detected)
d16S_bacteria_roots <- subset_taxa(d16S_bacteria_roots, !taxa_sums(d16S_bacteria_root
s) == 0)

# -----------------------------
# Check the prepared data
# -----------------------------

# Check the summary of the prepared fungal data
summary(d16S_fungus_roots)
```

```
##   Length    Class     Mode
##        1 phyloseq       S4
```

```
# Check the summary of the prepared bacterial data
summary(d16S_bacteria_roots)
```

```
##   Length    Class     Mode
##        1 phyloseq       S4
```

18.2 - Create the table for correlations

```
# Create a table joining fungal and bacterial relative abundances to calculate correl
ations among them

# Load necessary libraries
library(dplyr)
library(phyloseq)

# Step 1: Fungal Relative Abundance
fungi_abundance <- d16S_fungus_roots %>%
  tax_glom(taxrank = "Phylum") %>%                    # Agglomerate at Phylum level
  transform_sample_counts(function(x) {x / sum(x)}) %>% # Transform to relative abund
ance
  psmelt() %>%                                        # Melt to long format
  filter(Abundance > 0) %>%                           # Filter out rows where Abunda
nce is zero
  select(Sample, Phylum, Abundance, Pi.response, interval_ratioroot, ratioPi)

# Rinomina la colonna 'Phylum' direttamente con colnames
colnames(fungi_abundance)[which(colnames(fungi_abundance) == "Phylum")] <- "Fungi_Phy
lum"
colnames(fungi_abundance)[which(colnames(fungi_abundance) == "Abundance")] <- "Fungi_
Abundance"


# Step 2: Bacterial Relative Abundance
bacteria_abundance <- d16S_bacteria_roots %>%
  tax_glom(taxrank = "Order") %>%                    # Agglomerate at Order level
  transform_sample_counts(function(x) {x / sum(x)}) %>% # Transform to relative abund
ance
  psmelt() %>%                                        # Melt to long format
  filter(Abundance > 0) %>%                          # Filter for orders with non-ze
ro abundance
  select(Sample, Order, Abundance, Pi.response)
# Rename for clarity

# Rinomina le colonne manualmente
colnames(bacteria_abundance)[which(colnames(bacteria_abundance) == "Order")] <- "Bact
eria_Order"
colnames(bacteria_abundance)[which(colnames(bacteria_abundance) == "Abundance")] <-
"Bacteria_Abundance"

# Step 3: Fungal abundance matrix - separate by Sample and Phylum
fungi_matrix <- fungi_abundance %>%
  select(Sample, Fungi_Phylum, Fungi_Abundance) %>%
  spread(key = Fungi_Phylum, value = Fungi_Abundance, fill = 0)  # Convert to wide fo
rmat

# Step 4: Bacterial abundance matrix - separate by Sample and Order
bacteria_matrix <- bacteria_abundance %>%
  select(Sample, Bacteria_Order, Bacteria_Abundance) %>%
  spread(key = Bacteria_Order, value = Bacteria_Abundance, fill = 0)  # Convert to wi
de format

# Step 5: Combine both fungal and bacterial matrices based on the 'Sample' column
combined_matrix <- full_join(fungi_matrix, bacteria_matrix, by = "Sample")
```

```
# Step 6: Add Pi.response and other variables to the final matrix
final_matrix <- combined_matrix %>%
  left_join(fungi_abundance %>% select(Sample, Pi.response, interval_ratioroot, ratio
Pi), by = "Sample") %>%
  distinct() %>%  # Remove duplicate rows
  drop_na()  # Remove rows with missing values
```

#####18.3 - Calculate correlations

```
# Load necessary libraries
library(dplyr)
library(tidyr)
library(broom)

# Function to calculate correlation between fungal and bacterial orders
calculate_correlation <- function(data, fungi_phylum, bacteria_Order) {
    # Check if the fungi and bacteria orders exist in the data
    if (!(fungi_phylum %in% colnames(data))) {
        stop(paste("The column", fungi_phylum, "is not present in the dataset"))
    }

    if (!(bacteria_Order %in% colnames(data))) {
        stop(paste("The column", bacteria_Order, "is not present in the dataset"))
    }

    # Calculate correlation and p-value
    correlation_results <- cor.test(data[[fungi_phylum]], data[[bacteria_Order]],
        use = "complete.obs")

    # Return correlation results in a tibble
    return(tibble(fungi_phylum = fungi_phylum, bacteria_Order = bacteria_Order, corre
lation = correlation_results$estimate,
        p_value = correlation_results$p.value))
}

# Define fungal and bacterial orders for correlation
fungi_phylum <- c("p__Aphelidiomycota", "p__Ascomycota", "p__Basidiobolomycota",
    "p__Basidiomycota", "p__Calcarisporiellomycota", "p__Chytridiomycota", "p__Fungi_
phy_Incertae_sedis",
    "p__Glomeromycota", "p__Mortierellomycota", "p__Mucoromycota", "p__Olpidiomycot
a",
    "p__Rozellomycota")

# Bacterial orders for correlation
bacteria_order <- c("Burkholderiales", "Rhizobiales", "Micromonosporales", "Acholepla
smatales",
    "Streptomycetales", "Paenibacillales", "Xanthomonadales", "Flavobacteriales",
    "Bacillales", "Cellvibrionales", "Lachnospirales", "Sphingomonadales", "Bacteroid
ales",
    "Enterobacterales", "Micrococcales", "Propionibacteriales", "Caulobacterales",
    "Frankiales", "Corynebacteriales", "Acidobacteriales", "Pseudomonadales", "Subgro
up_2",
    "Staphylococcales", "Micropepsales", "Gaiellales", "Acetobacterales", "Solibacter
ales",
    "Pedosphaerales", "Polyangiales", "Cytophagales", "Oscillospirales", "Peptostrept
ococcales-Tissierellales",
    "Microtrichales", "Chloroflexales", "Lactobacillales", "Bryobacterales", "Haliang
iales",
    "Pseudonocardiales", "Solirubrobacterales", "Reyranellales", "Thermoactinomycetal
es",
    "Entotheonellales", "Thermicanales", "Steroidobacterales", "Gemmatimonadales",
    "Salinisphaerales", "Chlamydiales", "Streptosporangiales", "Babeliales", "Exiguob
acterales",
    "Bacteriovoracales", "Gammaproteobacteria_Incertae_Sedis", "Chitinophagales",
```

```
    "IMCC26256", "Tistrellales", "Rhodobacterales", "Saccharimonadales", "Sphingobact
eriales",
    "Elsterales", "WD260", "Thermoanaerobaculales", "Clostridiales", "Bdellovibrional
es",
    "Catenulisporales", "Rubrobacterales", "Brevibacillales", "Myxococcales", "Micavi
brionales",
    "Legionellales", "JG36-TzT-191", "Nannocystales", "Erysipelotrichales", "Ferrovib
rionales",
    "R7C24", "Pirellulales", "Deinococcales", "Nitrospirales", "Azospirillales",
    "Rhodospirillales", "Alicyclobacillales", "Alteromonadales", "Dongiales", "Longim
icrobiales",
    "Kineosporiales", "Fimbriimonadales", "Rhodothermales", "Vibrionales", "Chthoniob
acterales",
    "Vicinamibacterales", "PLTA13", "0319-6G20", "Spirochaetales", "Coriobacteriale
s",
    "S085", "CCD24", "Armatimonadales", "Rickettsiales", "Thermomicrobiales", "Phycis
phaerales",
    "NRL2", "Parvibaculales", "Holosporales", "Limnochordales", "Symbiobacteriales",
    "SAR202_clade", "SJA-15", "Candidatus_Kaiserbacteria", "Elev-16S-1166", "Pasteure
llales",
    "Clostridia_or", "Tepidisphaerales", "Fibrobacterales", "Actinomycetales", "Chris
tensenellales",
    "Clostridia_UCG-014", "Subgroup_7", "Aneurinibacillales", "Kapabacteriales",
    "Oligoflexales", "Nitrococcales", "KF-JG30-C25", "mle1-27", "Leptospirales",
    "Paracaedibacterales", "Ktedonobacterales", "Fusobacteriales", "Bifidobacteriale
s",
    "Dehalococcoidales", "Lineage_IV", "Kryptoniales", "Ardenticatenales", "Peptococc
ales",
    "Desulfovibrionales", "Limnochordia_or", "Subgroup_17", "Balneolales", "KI89A_cla
de",
    "Glycomycetales", "Subgroup_15", "Diplorickettsiales", "Caedibacterales", "Subgro
up_12",
    "Proteinivoracales", "Oceanospirillales", "SBR1031", "Isosphaerales", "0319-7L1
4",
    "Kallotenuales", "Desulfitobacteriales", "Ga0077536", "211ds20", "Nitrosococcale
s",
    "Izemoplasmatales", "Blfdi19", "Bradymonadales", "Abditibacteriales", "Syntrophor
habdales",
    "TSBb06", "Rokubacteriales", "Acidobacteriae_or", "Verrucomicrobiales", "Gemmatal
es",
    "Subgroup_13", "Blastocatellales", "Geobacterales", "Candidatus_Doudnabacteria",
    "Aeromonadales", "Silvanigrellales", "Desulfobaccales", "Holophagales", "Veillone
llales-Selenomonadales",
    "Entomoplasmatales", "OPB41", "C0119", "MSB-4B10", "Opitutales", "Granulosicoccal
es",
    "Planctomycetales", "B108", "EPR3968-O8a-Bc78", "mle1-8", "Acidimicrobiales",
    "Thermoanaerobacterales", "MVP-88", "Actinomarinales", "Coxiellales", "GIF9",
    "PeM15", "Defluviicoccales", "Syntrophales", "MA-28-I98C", "Thermophilales",
    "Acidaminococcales", "EV818SWSAP88", "RBG-13-54-9", "AKIW659", "Pyrinomonadales",
    "Euzebyales", "Methylococcales", "Eubacteriales", "PB19", "nov-24", "Caldilineale
s")

# Create an empty list to store the results
correlation_results <- list()

# Loop through each combination of fungi and bacteria order
```

```
for (fungi_phylum_name in fungi_phylum) {
    for (bacteria_order_name in bacteria_order) {
        # Calculate correlation for each pair and store the result
        result <- calculate_correlation(final_matrix, fungi_phylum_name, bacteria_ord
er_name)
        correlation_results[[paste(fungi_phylum_name, bacteria_order_name, sep =
"_")]] <- result
    }
}

# Convert the results list into a data frame
correlation_table <- bind_rows(correlation_results)

# Adjust p-values for multiple comparisons using FDR (False Discovery Rate)
correlation_table <- correlation_table %>%
    mutate(p_value_fdr = p.adjust(p_value, method = "fdr"))

# View the table of correlation results
print(correlation_table)
```

#####18.4 Figure S12D

```
# Load necessary libraries
library(ggpubr)
packageVersion("ggpubr")  # Correct function for checking package version
```

```
## [1] '0.6.1'
```

```
library(dplyr)

# Scatter plot without outliers
ggscatter(final_matrix, x = "Burkholderiales", y = "p__Glomeromycota",
          add = "reg.line", conf.int = TRUE,
          cor.coef = TRUE, cor.method = "pearson",
          size = 5,
          xlab = "Burkholderiales", ylab = "p__Glomeromycota")
```

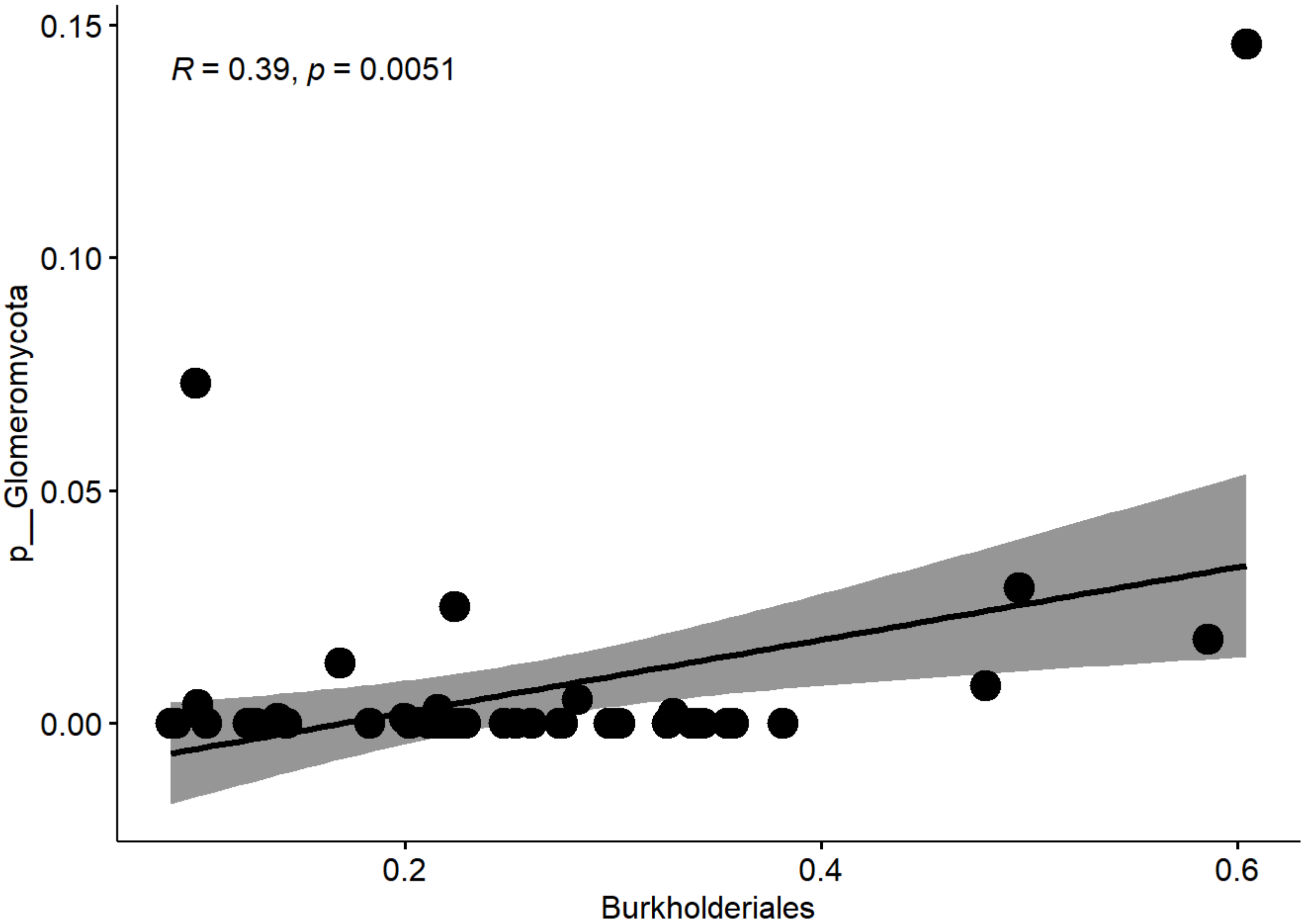

#####18.5 ASV level correlations

```
# Load necessary libraries
library(dplyr)
library(phyloseq)
library(tidyr)

# Step 1: Calculate the relative abundance for all samples
# Transform raw counts into relative abundance for each sample
d16S_bacteria_all <- d16S_bacteria_roots %>%
  transform_sample_counts(function(x) { x / sum(x) })  # Calculate relative abundance

# Step 2: Select the ASVs of interest by their name or ID
selected_ASVs <- c("ASV225", "ASV292", "ASV240","ASV294","ASV318", "ASV438", "ASV39
2", "ASV298", "ASV887", "ASV201", "ASV950", "ASV727", "ASV491", "ASV680", "ASV274",
"ASV132", "ASV215", "ASV95", "ASV451", "ASV133", "ASV186", "ASV711", "ASV166", "ASV52
1", "ASV156", "ASV534", "ASV7716", "ASV6126", "ASV2775", "ASV1803", "ASV1037", "ASV29
354", "ASV6004", "ASV5541", "ASV2862", "ASV5475", "ASV10389", "ASV10471", "ASV5608",
"ASV5282", "ASV6558", "ASV14131", "ASV318")

# Subset the data to include only the selected ASVs
d16S_bacteria_selected <- subset_taxa(d16S_bacteria_all, taxa_names(d16S_bacteria_al
l) %in% selected_ASVs)

# Step 3: Calculate the relative abundance of the selected ASVs
bacteria_abundance_selected <- d16S_bacteria_selected %>%
  psmelt() %>%  # Convert the data to long format
  filter(Abundance > 0) %>%  # Keep only non-zero abundance values
  select(Sample, OTU, Abundance, Pi.response)

# Step 4: Create a matrix with relative abundance separated by sample and OTU
bacteria_matrix_selected <- bacteria_abundance_selected %>%
  select(Sample, OTU, Abundance) %>%
  spread(key = OTU, value = Abundance, fill = 0)  # Spread the data to wide format

# Step 5: Combine the bacterial and fungal abundance matrices based on the 'Sample' c
olumn
combined_matrix_selected <- full_join(fungi_matrix, bacteria_matrix_selected, by = "S
ample")

# Step 6: Add response variables to the final matrix
final_matrix_selected <- combined_matrix_selected %>%
  left_join(fungi_abundance %>% select(Sample, Pi.response, interval_ratioroot, ratio
Pi), by = "Sample") %>%
  distinct() %>%  # Remove duplicate rows
  drop_na()  # Remove rows with missing values

# Step 7: Clean up the data types of the final matrix columns
final_matrix_selected$Sample <- as.factor(final_matrix_selected$Sample)
final_matrix_selected$Pi.response <- as.factor(final_matrix_selected$Pi.response)
final_matrix_selected$ratioPi <- as.numeric(final_matrix_selected$ratioPi)

# Step 8: Display the column names of the final matrix
colnames(final_matrix_selected)
```

```
##  [1] "Sample"                      "p__Aphelidiomycota"
##  [3] "p__Ascomycota"               "p__Basidiobolomycota"
##  [5] "p__Basidiomycota"            "p__Calcarisporiellomycota"
##  [7] "p__Chytridiomycota"          "p__Fungi_phy_Incertae_sedis"
##  [9] "p__Glomeromycota"            "p__Kickxellomycota"
## [11] "p__Mortierellomycota"        "p__Mucoromycota"
## [13] "p__Olpidiomycota"            "p__Rozellomycota"
## [15] "ASV1037"                     "ASV10471"
## [17] "ASV132"                      "ASV133"
## [19] "ASV14131"                    "ASV156"
## [21] "ASV166"                      "ASV1803"
## [23] "ASV186"                      "ASV201"
## [25] "ASV215"                      "ASV225"
## [27] "ASV240"                      "ASV274"
## [29] "ASV2775"                     "ASV2862"
## [31] "ASV292"                      "ASV294"
## [33] "ASV298"                      "ASV318"
## [35] "ASV392"                      "ASV438"
## [37] "ASV451"                      "ASV491"
## [39] "ASV521"                      "ASV5282"
## [41] "ASV534"                      "ASV5475"
## [43] "ASV5541"                     "ASV5608"
## [45] "ASV6004"                     "ASV6126"
## [47] "ASV6558"                     "ASV680"
## [49] "ASV711"                      "ASV727"
## [51] "ASV7716"                     "ASV887"
## [53] "ASV95"                       "ASV950"
## [55] "Pi.response"                 "interval_ratioroot"
## [57] "ratioPi"
```

#####18.6 Figure S12B

```
# Step 1: Convert Pi.response to a factor and filter for Pi.response == "+"
final_matrix_selected$Pi.response <- as.factor(final_matrix_selected$Pi.response)

# Filter the data for Pi.response == "+"
final_matrix_Pi_selected <- final_matrix_selected %>%
  filter(Pi.response == "+") %>%
  droplevels()  # Remove unused levels from the factor

# Step 2: Summarize the filtered data
summary(final_matrix_Pi_selected)
```

```
##      Sample  p__Aphelidiomycota  p__Ascomycota    p__Basidiobolomycota
##  A10    :1   Min.   :0.0000000   Min.   :0.3660   Min.   :0.0000000
##  A12    :1   1st Qu.:0.0000000   1st Qu.:0.8260   1st Qu.:0.0000000
##  A16    :1   Median :0.0000000   Median :0.8610   Median :0.0000000
##  A21    :1   Mean   :0.0002308   Mean   :0.8332   Mean   :0.0003077
##  A22    :1   3rd Qu.:0.0000000   3rd Qu.:0.8840   3rd Qu.:0.0000000
##  A27    :1   Max.   :0.0030000   Max.   :0.9890   Max.   :0.0040000
##  (Other):7
##  p__Basidiomycota  p__Calcarisporiellomycota p__Chytridiomycota
##  Min.   :0.00200   Min.   :0                 Min.   :0.000000
##  1st Qu.:0.04400   1st Qu.:0                 1st Qu.:0.000000
##  Median :0.08700   Median :0                 Median :0.000000
##  Mean   :0.07831   Mean   :0                 Mean   :0.004923
##  3rd Qu.:0.11600   3rd Qu.:0                 3rd Qu.:0.003000
##  Max.   :0.14200   Max.   :0                 Max.   :0.030000
##
##  p__Fungi_phy_Incertae_sedis p__Glomeromycota    p__Kickxellomycota
##  Min.   :0.00900             Min.   :0.0000000   Min.   :0
##  1st Qu.:0.01600             1st Qu.:0.0000000   1st Qu.:0
##  Median :0.02000             Median :0.0000000   Median :0
##  Mean   :0.02777             Mean   :0.0003846   Mean   :0
##  3rd Qu.:0.03300             3rd Qu.:0.0000000   3rd Qu.:0
##  Max.   :0.08300             Max.   :0.0040000   Max.   :0
##
##  p__Mortierellomycota p__Mucoromycota     p__Olpidiomycota  p__Rozellomycota
##  Min.   :0.000000     Min.   :0.0000000   Min.   :0.00000   Min.   :0.0000000
##  1st Qu.:0.000000     1st Qu.:0.0000000   1st Qu.:0.00000   1st Qu.:0.0000000
##  Median :0.002000     Median :0.0000000   Median :0.00200   Median :0.0000000
##  Mean   :0.003462     Mean   :0.0008462   Mean   :0.04969   Mean   :0.0008462
##  3rd Qu.:0.004000     3rd Qu.:0.0010000   3rd Qu.:0.01100   3rd Qu.:0.0010000
##  Max.   :0.017000     Max.   :0.0050000   Max.   :0.55700   Max.   :0.0050000
##
##     ASV1037            ASV10471             ASV132
##  Min.   :0.000e+00   Min.   :0.000e+00   Min.   :0.0000000
##  1st Qu.:0.000e+00   1st Qu.:0.000e+00   1st Qu.:0.0000000
##  Median :0.000e+00   Median :0.000e+00   Median :0.0000000
##  Mean   :7.692e-05   Mean   :7.692e-05   Mean   :0.0006154
##  3rd Qu.:0.000e+00   3rd Qu.:0.000e+00   3rd Qu.:0.0010000
##  Max.   :1.000e-03   Max.   :1.000e-03   Max.   :0.0040000
##
##      ASV133             ASV14131     ASV156      ASV166
##  Min.   :0.0000000   Min.   :0   Min.   :0   Min.   :0.0000000
##  1st Qu.:0.0000000   1st Qu.:0   1st Qu.:0   1st Qu.:0.0000000
##  Median :0.0000000   Median :0   Median :0   Median :0.0000000
##  Mean   :0.0002308   Mean   :0   Mean   :0   Mean   :0.0001538
##  3rd Qu.:0.0000000   3rd Qu.:0   3rd Qu.:0   3rd Qu.:0.0000000
##  Max.   :0.0020000   Max.   :0   Max.   :0   Max.   :0.0020000
##
##     ASV1803             ASV186              ASV201
##  Min.   :0.0000000   Min.   :0.0000000   Min.   :0.0000000
##  1st Qu.:0.0000000   1st Qu.:0.0000000   1st Qu.:0.0000000
##  Median :0.0000000   Median :0.0000000   Median :0.0000000
##  Mean   :0.0001538   Mean   :0.0001538   Mean   :0.0006154
##  3rd Qu.:0.0000000   3rd Qu.:0.0000000   3rd Qu.:0.0010000
##  Max.   :0.0010000   Max.   :0.0010000   Max.   :0.0050000
```

```
##
##      ASV215              ASV225             ASV240              ASV274
##  Min.   :0.0000000   Min.   :0.000000   Min.   :0.000e+00   Min.   :0.0000000
##  1st Qu.:0.0000000   1st Qu.:0.000000   1st Qu.:0.000e+00   1st Qu.:0.0000000
##  Median :0.0000000   Median :0.000000   Median :0.000e+00   Median :0.0000000
##  Mean   :0.0003077   Mean   :0.001538   Mean   :7.692e-05   Mean   :0.0008462
##  3rd Qu.:0.0000000   3rd Qu.:0.001000   3rd Qu.:0.000e+00   3rd Qu.:0.0000000
##  Max.   :0.0020000   Max.   :0.012000   Max.   :1.000e-03   Max.   :0.0070000
##
##     ASV2775     ASV2862              ASV292          ASV294
##  Min.   :0   Min.   :0.000e+00   Min.   :0.000   Min.   :0.0000000
##  1st Qu.:0   1st Qu.:0.000e+00   1st Qu.:0.000   1st Qu.:0.0000000
##  Median :0   Median :0.000e+00   Median :0.000   Median :0.0000000
##  Mean   :0   Mean   :7.692e-05   Mean   :0.001   Mean   :0.0007692
##  3rd Qu.:0   3rd Qu.:0.000e+00   3rd Qu.:0.002   3rd Qu.:0.0000000
##  Max.   :0   Max.   :1.000e-03   Max.   :0.007   Max.   :0.0060000
##
##      ASV298              ASV318             ASV392             ASV438
##  Min.   :0.0000000   Min.   :0.000000   Min.   :0.000000   Min.   :0.0000000
##  1st Qu.:0.0000000   1st Qu.:0.000000   1st Qu.:0.000000   1st Qu.:0.0000000
##  Median :0.0000000   Median :0.000000   Median :0.000000   Median :0.0000000
##  Mean   :0.0008462   Mean   :0.001385   Mean   :0.001231   Mean   :0.0009231
##  3rd Qu.:0.0000000   3rd Qu.:0.002000   3rd Qu.:0.000000   3rd Qu.:0.0000000
##  Max.   :0.0110000   Max.   :0.005000   Max.   :0.014000   Max.   :0.0050000
##
##      ASV451              ASV491              ASV521
##  Min.   :0.0000000   Min.   :0.0000000   Min.   :0.0000000
##  1st Qu.:0.0000000   1st Qu.:0.0000000   1st Qu.:0.0000000
##  Median :0.0000000   Median :0.0000000   Median :0.0010000
##  Mean   :0.0008462   Mean   :0.0002308   Mean   :0.0007692
##  3rd Qu.:0.0020000   3rd Qu.:0.0000000   3rd Qu.:0.0010000
##  Max.   :0.0030000   Max.   :0.0020000   Max.   :0.0020000
##
##     ASV5282              ASV534             ASV5475
##  Min.   :0.0000000   Min.   :0.000e+00   Min.   :0.0000000
##  1st Qu.:0.0000000   1st Qu.:0.000e+00   1st Qu.:0.0000000
##  Median :0.0000000   Median :0.000e+00   Median :0.0000000
##  Mean   :0.0001538   Mean   :7.692e-05   Mean   :0.0003077
##  3rd Qu.:0.0000000   3rd Qu.:0.000e+00   3rd Qu.:0.0000000
##  Max.   :0.0020000   Max.   :1.000e-03   Max.   :0.0040000
##
##     ASV5541             ASV5608              ASV6004     ASV6126
##  Min.   :0.000e+00   Min.   :0.0000000   Min.   :0   Min.   :0
##  1st Qu.:0.000e+00   1st Qu.:0.0000000   1st Qu.:0   1st Qu.:0
##  Median :0.000e+00   Median :0.0000000   Median :0   Median :0
##  Mean   :7.692e-05   Mean   :0.0001538   Mean   :0   Mean   :0
##  3rd Qu.:0.000e+00   3rd Qu.:0.0000000   3rd Qu.:0   3rd Qu.:0
##  Max.   :1.000e-03   Max.   :0.0020000   Max.   :0   Max.   :0
##
##     ASV6558              ASV680              ASV711
##  Min.   :0.000e+00   Min.   :0.0000000   Min.   :0.0000000
##  1st Qu.:0.000e+00   1st Qu.:0.0000000   1st Qu.:0.0000000
##  Median :0.000e+00   Median :0.0000000   Median :0.0000000
##  Mean   :7.692e-05   Mean   :0.0001538   Mean   :0.0006923
##  3rd Qu.:0.000e+00   3rd Qu.:0.0000000   3rd Qu.:0.0010000
##  Max.   :1.000e-03   Max.   :0.0020000   Max.   :0.0040000
```

```
##
##      ASV727              ASV7716      ASV887              ASV95
##  Min.   :0.0000000   Min.   :0   Min.   :0.0000000   Min.   :0.000000
##  1st Qu.:0.0000000   1st Qu.:0   1st Qu.:0.0000000   1st Qu.:0.000000
##  Median :0.0000000   Median :0   Median :0.0000000   Median :0.000000
##  Mean   :0.0006154   Mean   :0   Mean   :0.0006154   Mean   :0.002769
##  3rd Qu.:0.0000000   3rd Qu.:0   3rd Qu.:0.0010000   3rd Qu.:0.000000
##  Max.   :0.0040000   Max.   :0   Max.   :0.0050000   Max.   :0.030000
##
##      ASV950          Pi.response interval_ratioroot    ratioPi
##  Min.   :0.0000000   +:13        -:1                 Min.   :1.235
##  1st Qu.:0.0000000               /:7                 1st Qu.:1.314
##  Median :0.0000000               +:5                 Median :1.578
##  Mean   :0.0001538                                   Mean   :1.553
##  3rd Qu.:0.0000000                                   3rd Qu.:1.729
##  Max.   :0.0020000                                   Max.   :1.994
##
```

```
# Step 3: Check the distribution of ASV225 (or any other ASV of interest)
table(final_matrix_Pi_selected$ASV225)  # Change this to another ASV column if needed
```

```
##
##     0 0.001 0.006 0.012
##     9     2     1     1
```

```
# Step 4: Scatter plot with regression line and correlation coefficient
ggscatter(final_matrix_Pi_selected, x = "ASV225", y = "p__Glomeromycota",
          add = "reg.line", conf.int = TRUE,
          cor.coef = TRUE, cor.method = "pearson",
          xlab = "ASV225", ylab = "Abbondanza relativa di p__Glomeromycota",
          color = "Pi.response",   # Color points based on Pi.response
          palette = "jco",         # Choose a color palette
          na.rm = TRUE) +          # Remove NA values
  facet_wrap(~ Pi.response) +  # Create a separate plot for each level of Pi.response
  theme(legend.position = "none")  # Remove the legend if not necessary
```

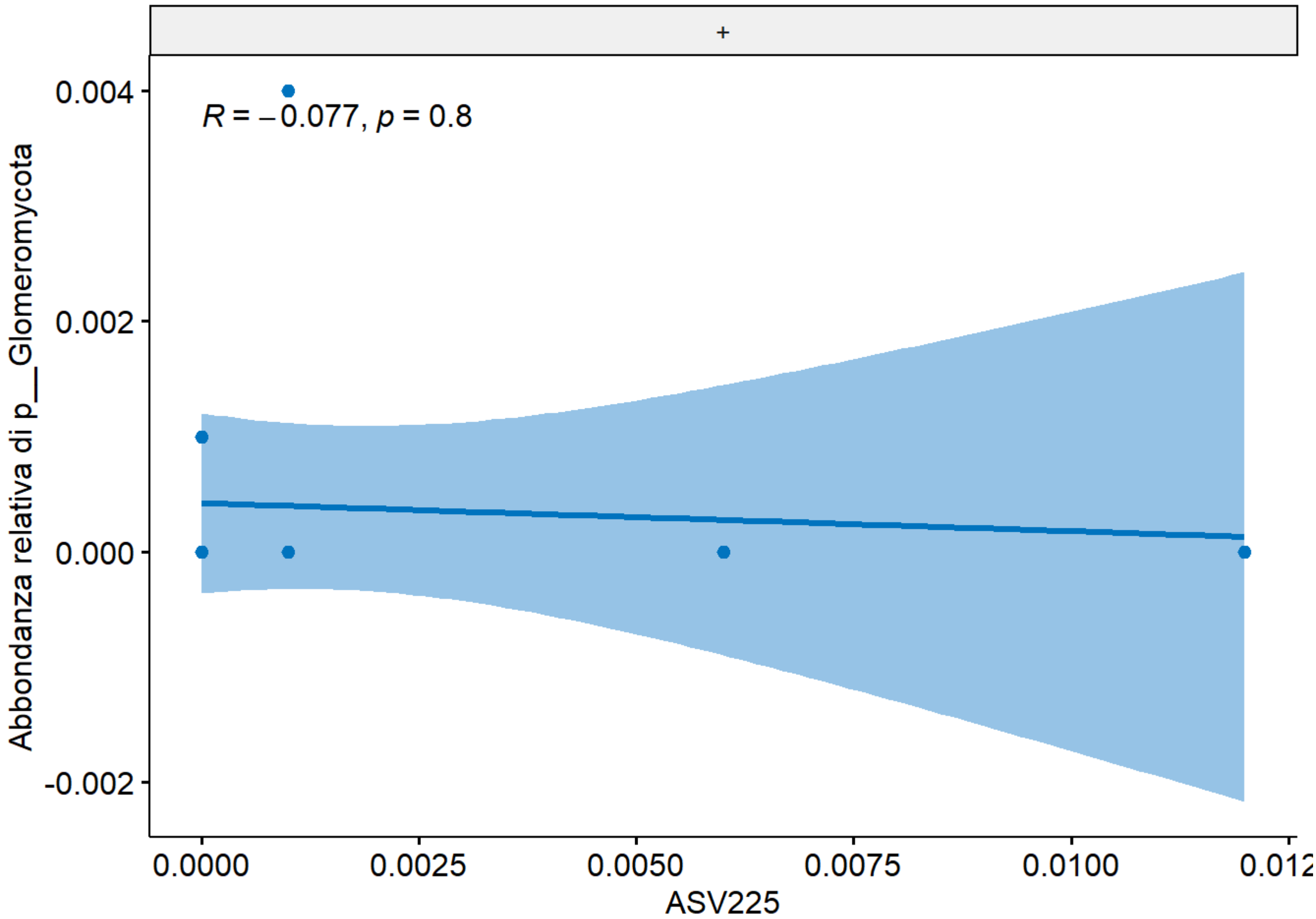

#####18.7 Figure S12C

```
# Step 1: Convert interval_ratioroot to a factor and filter for interval_ratioroot ==
"-"
final_matrix_selected$interval_ratioroot <- as.factor(final_matrix_selected$interval_
ratioroot)

# Filter the data for interval_ratioroot == "-"
final_matrix_Pi_selected <- final_matrix_selected %>%
  filter(interval_ratioroot == "-") %>%
  droplevels()  # Remove unused levels from the factor

# Step 2: Scatter plot with regression line, correlation coefficient, and faceting by
interval_ratioroot
ggscatter(final_matrix_Pi_selected, x = "ASV166", y = "p__Glomeromycota",
          add = "reg.line", conf.int = TRUE,
          cor.coef = TRUE, cor.method = "pearson",
          xlab = "ASV166", ylab = "Abbondanza relativa di p__Glomeromycota",
          color = "interval_ratioroot",   # Color points based on interval_ratioroot
          palette = "jco",               # Choose a color palette
          size = 6) +                    # Adjust point size
  facet_wrap(~ interval_ratioroot) +  # Create a separate plot for each level of inte
rval_ratioroot
  theme(legend.position = "none")    # Remove the legend if it's not necessary
```

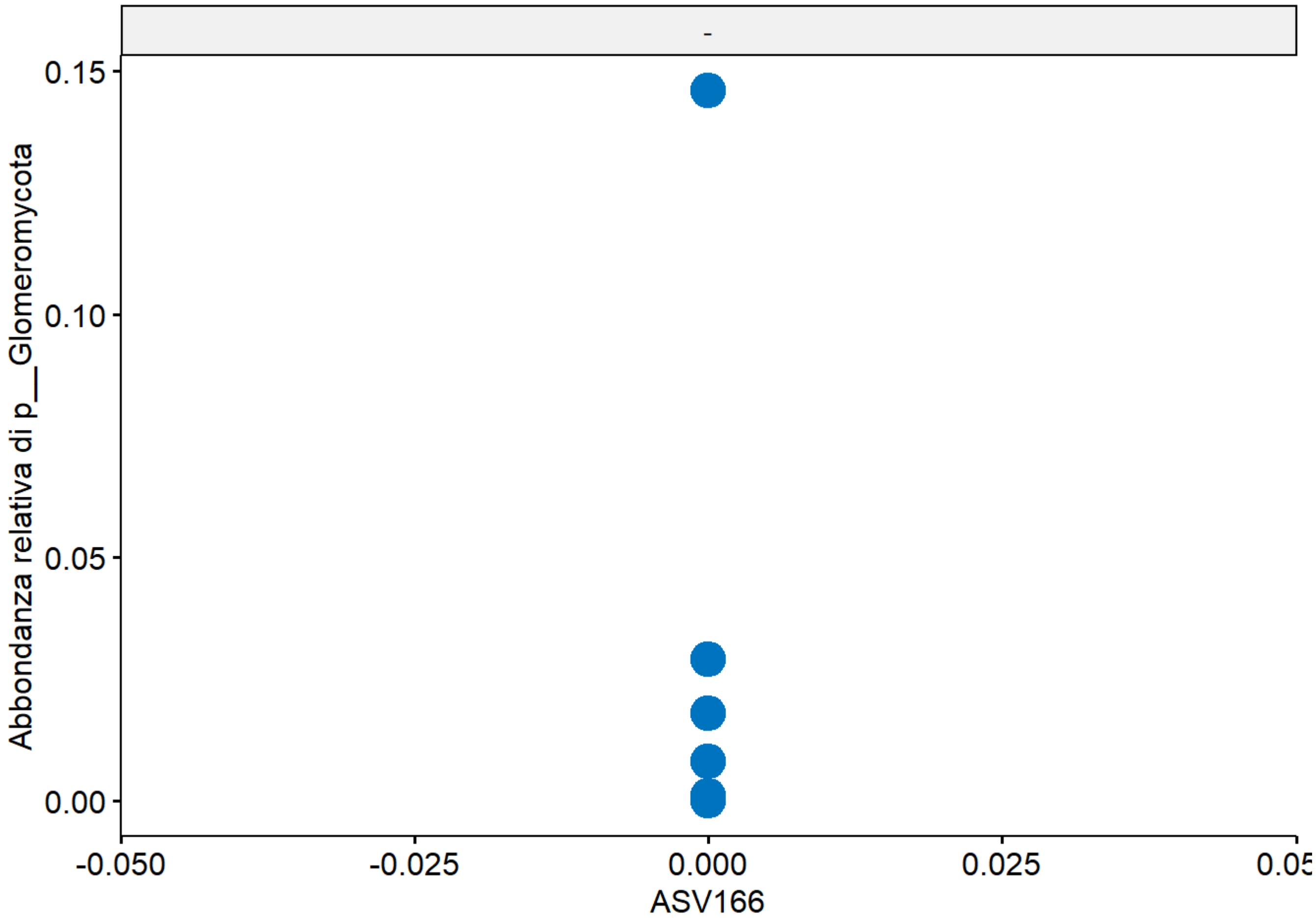

#19 - Figure S1 - Trouvelot analysis

```
###1-  libraries

library(ggExtra)
library(tidyverse)
library(multcomp)
library(ggpubr)
library(rstatix)
library(patchwork)
library(dplyr)
library(agricolae)
library(car)
```

```
## Caricamento del pacchetto richiesto: carData
```

```
##
## Caricamento pacchetto: 'car'
```

```
## Il seguente oggetto è mascherato da 'package:psych':
##
##     logit
```

```
## Il seguente oggetto è mascherato da 'package:dplyr':
##
##     recode
```

```
## Il seguente oggetto è mascherato da 'package:purrr':
##
##     some
```

```
###2- Import Suppelementary Table 13


data_Trouvelot <- read.csv("C:/Users/Alber/Desktop/DOTTORATO/LATTUGA/LANDLAB/Trouvelo
t/Trouvelot.csv", #replace with your path
                 header=T,sep = ";", na.string=c("na", "NA", "null"), check.names = F
ALSE)


###3- transform into factor

data_Trouvelot$position <- as.factor(data_Trouvelot$position)
data_Trouvelot$genotype <- as.factor(data_Trouvelot$genotype)
data_Trouvelot$treatment <- as.factor(data_Trouvelot$treatment)
data_Trouvelot$accession <-as.factor(data_Trouvelot$accession)
data_Trouvelot$accession_2<-as.factor(data_Trouvelot$accession_2)

###4 - Boxplot - Figure S1

library(tidyr)
library(ggplot2)
library(rstatix)
library(ggpubr)

###5 - Tranform with pivot_longer
dati_long <- data_Trouvelot %>%
  pivot_longer(
    cols = c(`F%`, `M%`, `m%`, `a%`, `A%`),
    names_to = "variable",
    values_to = "value"
  )

###6- Define variables
variabili <- c("F%", "M%", "m%", "a%", "A%")

###7- Boxplot for every variable - Figure S1

for (var in variabili) {
  p <- ggplot(dati_long %>% filter(variable == var),
            aes(x = genotype, y = value, fill = treatment)) +
    geom_boxplot(position = position_dodge(0.8)) +
    geom_jitter(position = position_jitterdodge(jitter.width = 0.2, dodge.width = 0.
8), size = 3) +
    labs(title = var, x = "Genotype", y = "Mychorization", fill = "Treatment") +
    scale_fill_manual(values = c("gray90", "#cc742c")) +
    theme_minimal() +
    theme(axis.text.x = element_text(angle = 45, hjust = 1))

  print(p)
}
```

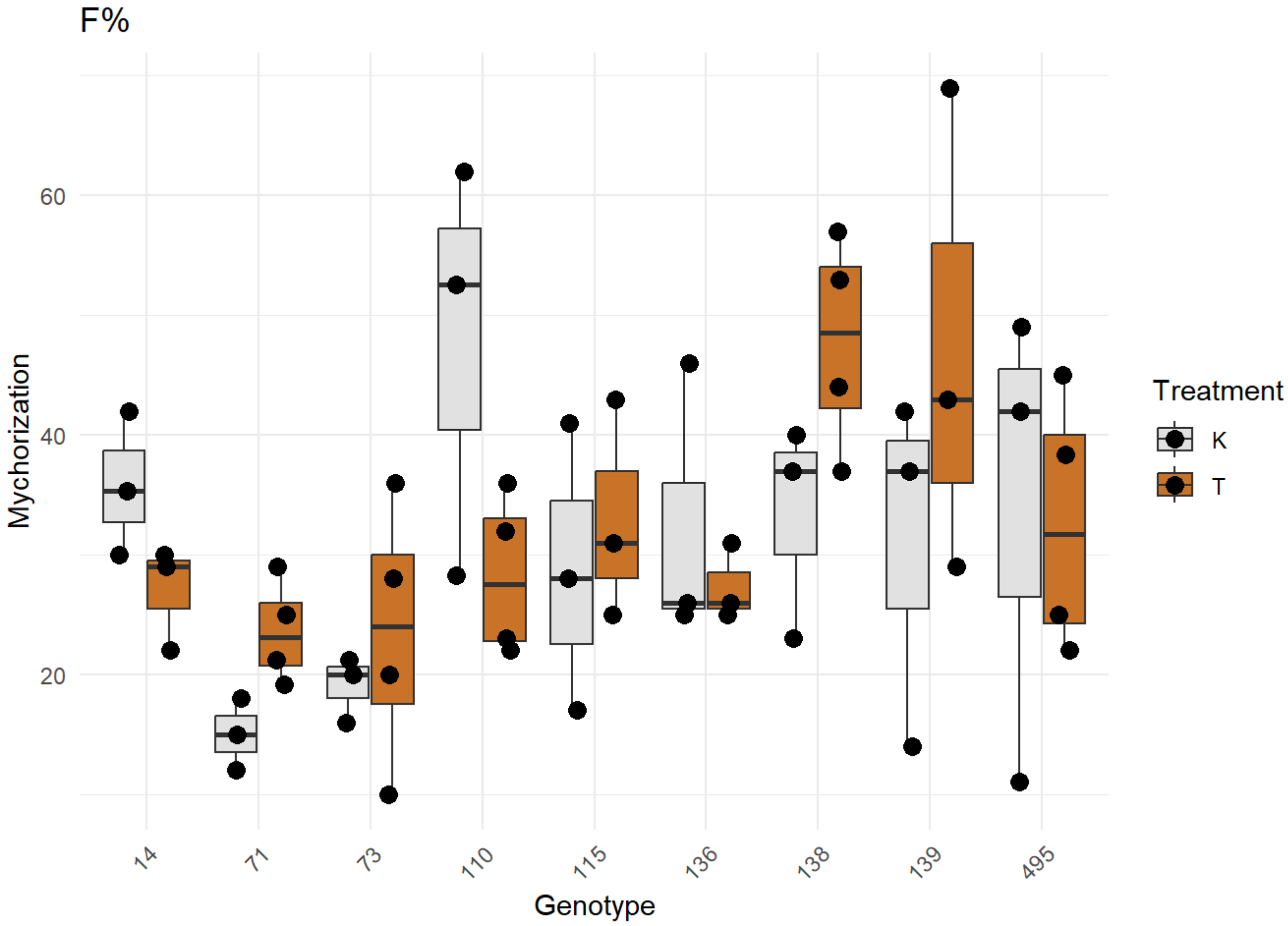
F%
60
40
20
Mychorization
14
71
73
110
115
136
138
139
495
Genotype
Treatment
K
T

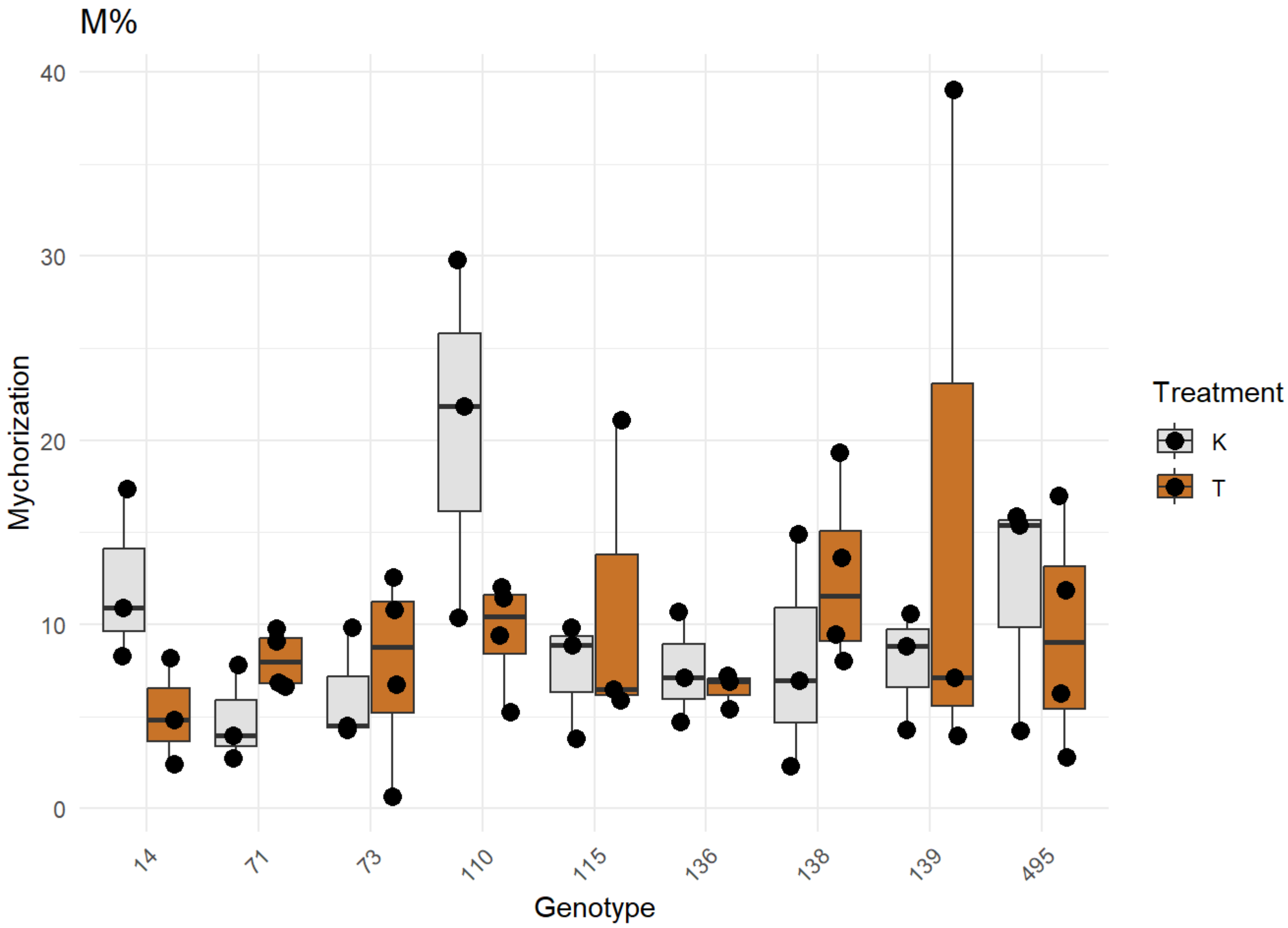
M%
40
30
20
10
0
Mychorization
14
71
73
110
115
136
138
139
495
Genotype
Treatment
K
T

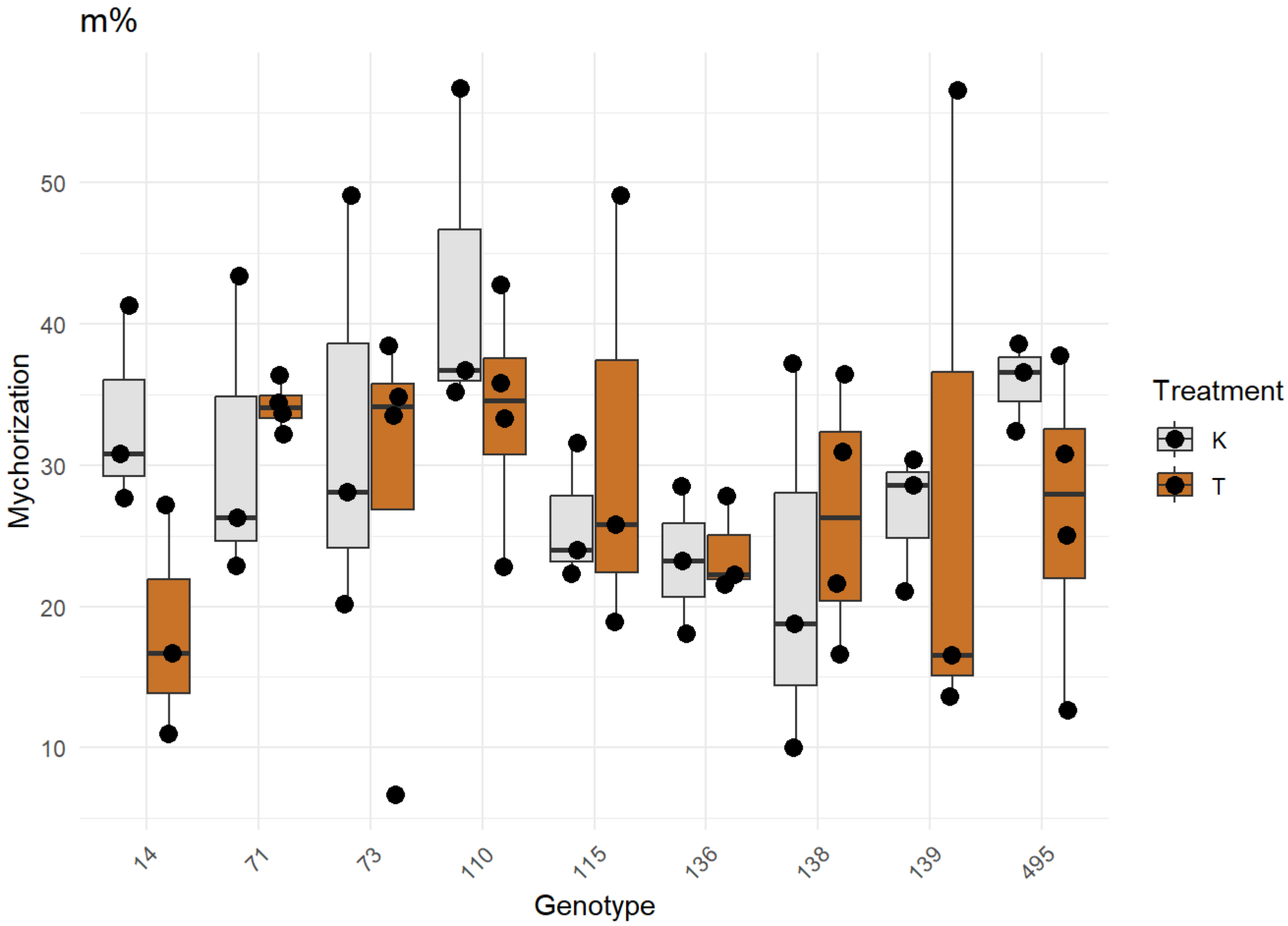
m%
Mychorization
10
20
30
40
50
14
71
73
110
115
136
138
139
495
Genotype
Treatment
K
T

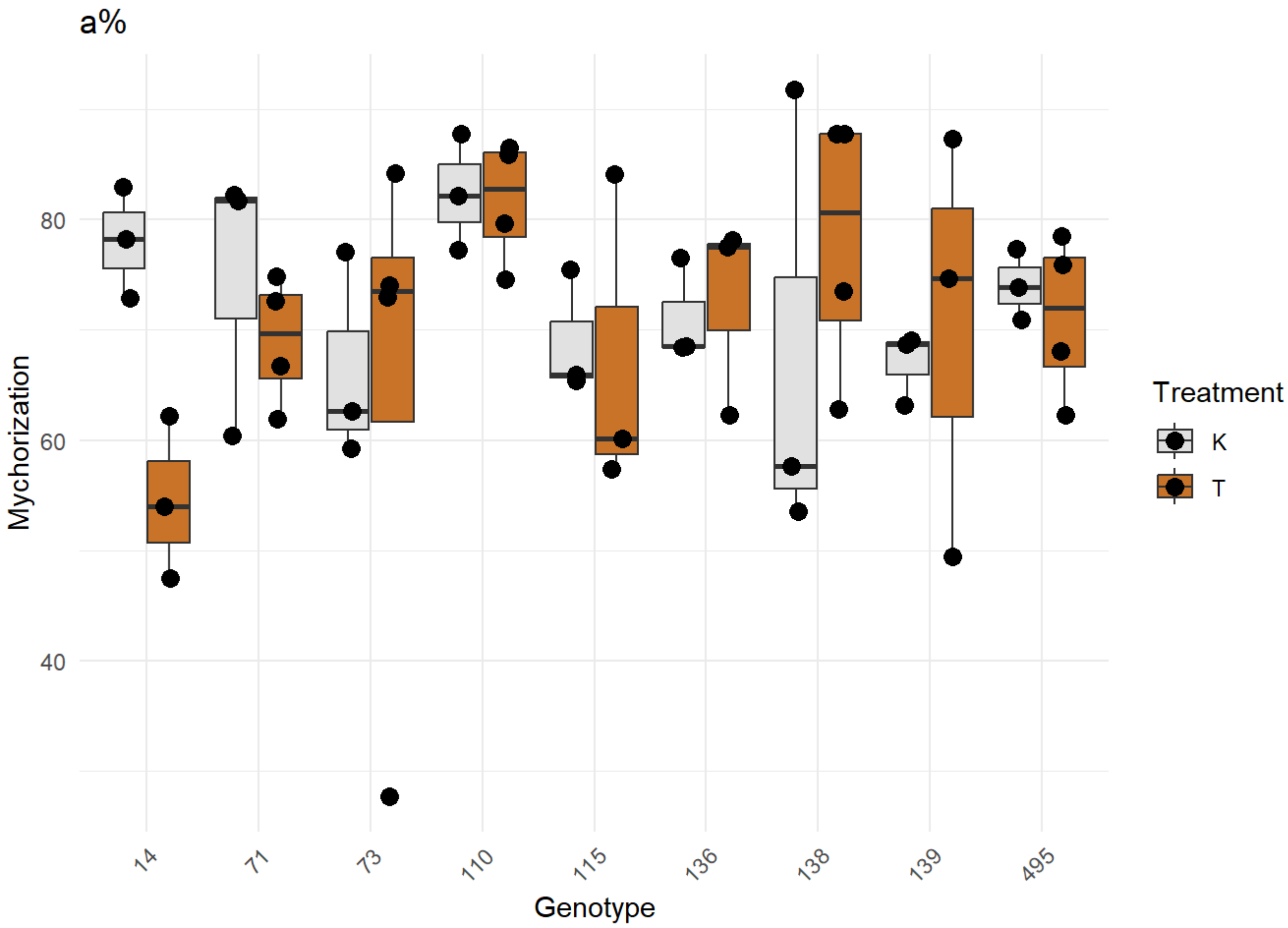
a%
Mychorization
40
60
80
14
71
73
110
115
136
138
139
495
Genotype
Treatment
K
T

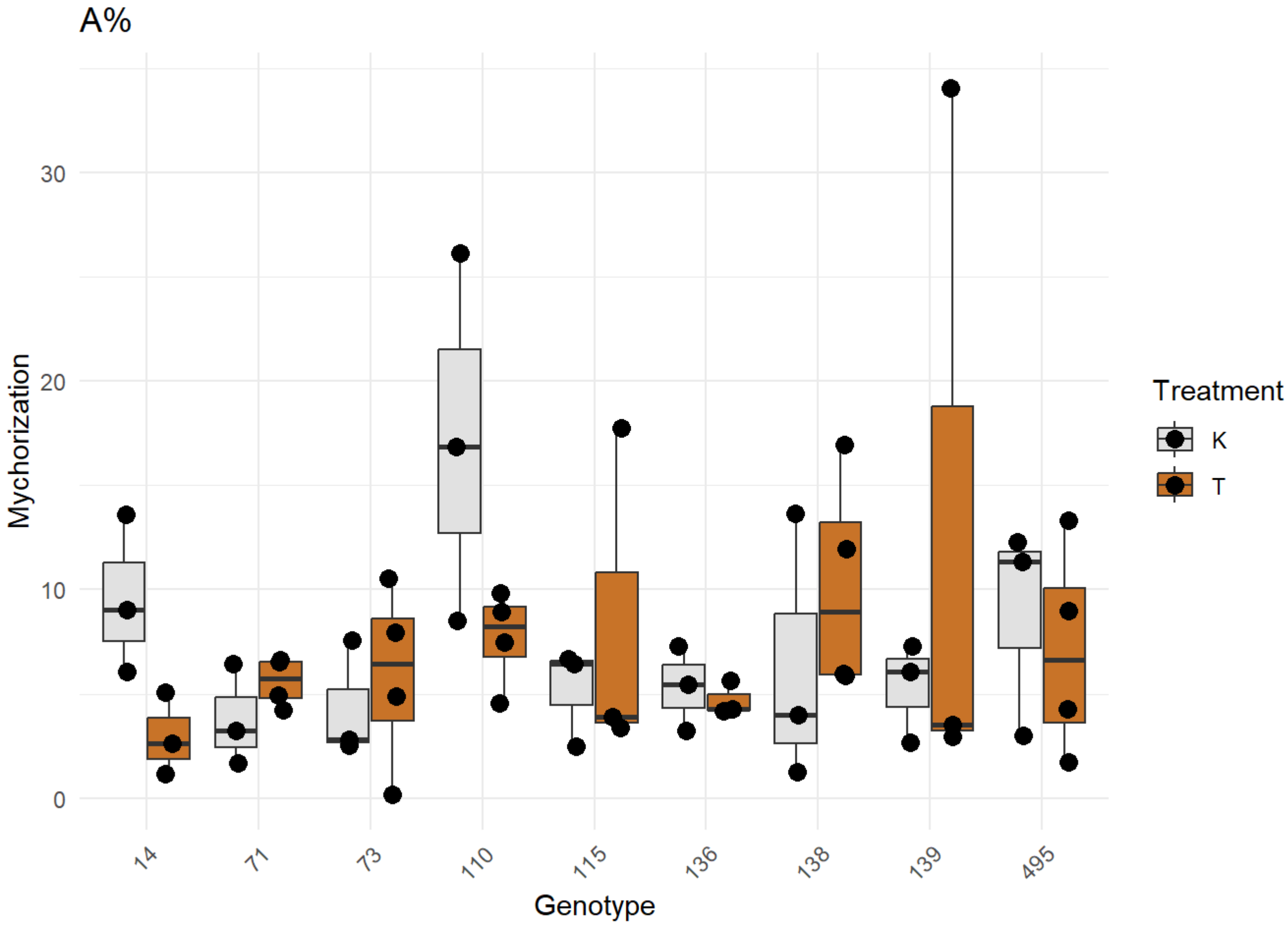


```
###8- Statistical analysis - Dunn test between treatments in each genotype

Dunn_test <- dati_long %>%
  group_by(genotype, variable) %>%
  dunn_test(value ~ treatment, p.adjust.method = "fdr")
```
